# Structural control of dynamic covalent cages: kinetic *vs.* thermodynamic assembly and PFAS removal from water†

**DOI:** 10.1039/d5sc02247a

**Published:** 2025-06-18

**Authors:** Tobias Pausch, Pablo Martínez Mestre, Fabiola Zapata, Andreas Mix, Bernd M. Schmidt

**Affiliations:** a Institut für Organische Chemie und Makromolekulare Chemie, Heinrich-Heine-Universität Düsseldorf Universitätsstraße 1 Düsseldorf 40225 Germany bernd.schmidt@hhu.de; b Departamento de Química Orgánica, Universidad de Murcia Edificio 19 Murcia 30100 Spain; c Institut für Anorganische Chemie und Strukturchemie, Universität, Bielefeld Universitätsstr. 25 Bielefeld 33615 Germany

## Abstract

Dynamic covalent chemistry is a powerful tool to synthesise complex structures from simple building blocks. However, even minor variations in the numerous parameters governing self-assembly can drastically influence the size and structure of the resulting assemblies. Herein, we report the selective formation of three cages belonging to the low-symmetry Tri^2^_2_Tri^2^ cage topology for the first time, using highly symmetric tritopic building blocks, confirmed by single-crystal X-ray (SC-XRD) analysis. Fluorinated and non-fluorinated aldehydes were combined with two amines differing in their degree of structural flexibility. Applying either kinetic or thermodynamic control through solvent selection allowed for the selective synthesis of either the low-symmetry Tri^2^_2_Tri^2^ or the larger, highly symmetric Tri^4^Tri^4^ assemblies. While the fluorinated linker strongly preferred the formation of the Tri^2^_2_Tri^2^ cage topology under thermodynamic control, the non-fluorinated linker selectively formed the Tri^4^Tri^4^ species. Kinetic control, using methanol as a poor solvent, allowed for the selective precipitation of the Tri^2^_2_Tri^2^ intermediate. Reduction of the Janus-like fluorinated Tri^2^_2_Tri^2^ cages yielded the cages Et^2^F^2^_red_ and TREN^2^F^2^_red_, which showed high potential for removing perfluorooctanoic acid (PFOA) from water, with Et^2^F^2^_red_ exhibiting structural rearrangements in organic solvents to accommodate PFOA, as observed by ^1^H and ^19^F NMR titrations in combination with ^19^F DOSY measurements.

## Abstract 2

Selective formation of low-symmetry Tri^2^_2_Tri^2^*via* dynamic covalent chemistry was achieved. Solvent choice enabled kinetic or thermodynamic control, directing assembly towards Tri^2^_2_Tri^2^ or high-symmetry Tri^4^Tri^4^ cages.

## Graphical abstract

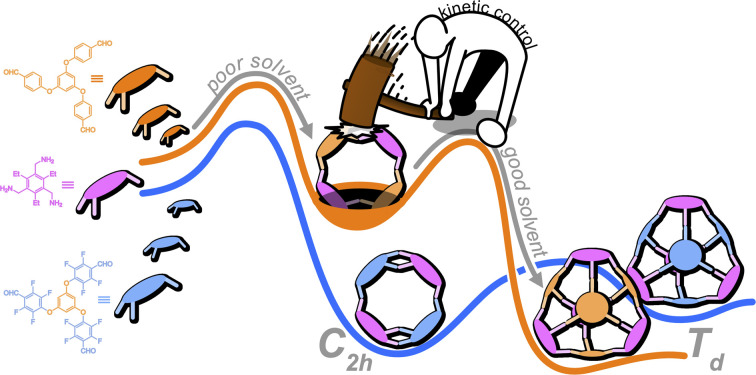

## Introduction

Molecular self-assembly using organic building blocks, in combination with metals or in their absence, gives access to a variety of nanostructures through the organisation of tailor-made molecular building blocks that exhibit suitable intra- and intermolecular interactions.^1^ Organic chemistry-rooted dynamic covalent chemistry (DCC) is therefore an efficient synthetic strategy that employs multitopic precursors to form reversible covalent bonds, combining the advantages of error correction during self-assembly with stability, allowing for in-solution analysis and application of the assembled molecule.^2^ It has enabled the synthesis of a variety of supramolecular architectures, from macrocycles^3^ to cages,^4^ polymers,^5^ and covalent organic frameworks (COFs),^6^ with potential energy surfaces being the central concept in understanding the assembly outcome.^4*a*,7^

Building blocks of similar topology, through changes in geometry,^1*c*,8^ size,^1,4,8^ rigidity of linkers,^1,9^ or even changes in substituents^10^ can build a multitude of accessible cage topologies.^4,11^ Recently, Jelfs and co-workers rationalised expected topologies (connection patterns) by analysing the geometry and topology of building blocks through calculations combined with experimentally observed structures.^11*b*^ They also introduced a systematic nomenclature for describing cage topologies, denoted as **X**^*m*^_p_**Y**^*n*^, providing clarity and avoiding confusion with bracket notation, such as [4 + 2], which is more commonly associated with pericyclic reactions in organic chemistry.

**Fig. 1:**
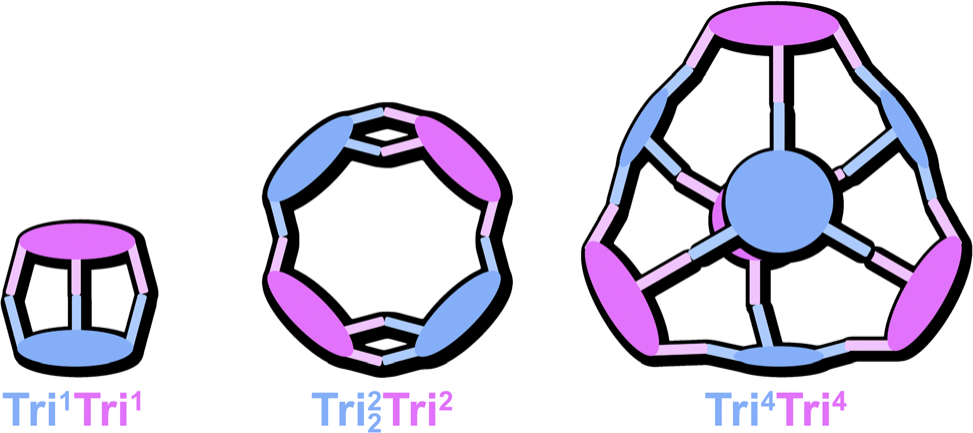
Schematic representation of the cage geometries obtained when reacting two tritopic building blocks with each other, giving different topologies.

By combining ditopic (Di), tritopic (Tri), or tetratopic (Tet) building blocks, a diverse library of cage geometries can be envisioned. Although several gaps have been filled in recent years, some predicted geometries remain unobserved, with no corresponding crystal structures reported. Within the Tri^*n*^Tri^*n*^ family (Fig. 1), Tri^4^Tri^4^ is the only geometry for which crystal structures have been commonly obtained.^12^ In contrast, Tri^1^Tri^1^ has mostly only been observed in solution,^13^ while the lower-symmetry Tri^2^_2_Tri^2^ topology has yet to be reported. The formation of such complex, lower-symmetry structures is relatively rare compared to highly symmetric Platonic or Archimedean solid-based topologies, such as cubes^14^ or tetrahedra.^15^ Computational studies have shown that the higher symmetry of such assemblies is generally preferred from an entropic point of view,^16*a*^ however, it cannot be fully disregarded that the formation of multiple smaller structures, disregarding their symmetry, should be overall more beneficial for the entropic term.^11*b*,16*b*^

Especially in recent years, the interest in these intricate assemblies has grown due to their unique and selective host–guest chemistry.^17,18^ For metal–organic cages (MOCs), various strategies have been explored to achieve complex assemblies.^17^ One common approach involves employing multiple linkers to form heteroleptic cages, effectively disrupting the symmetry of the final structure.^19^ In DCC-based systems this approach is of great interest, however, the desired social self-sorting^4*c*,20,21^ is rare, and narcissistic self-sorting^10,22^ or statistical mixtures^23^ are dominating this space. Thus, lower-symmetry assemblies are typically achieved by employing less symmetric building blocks, often exhibiting an inherent chirality.^18,22*a*,*b*,24^ For example, He and Zhang *et al.* demonstrated that the use of *C*_2_ and *C*_2v_ building blocks leads to the formation of a *C*_2_-symmetric imine cage of the unusual Tet^4^_4_Di^8^ topology.^24*b*^

Beyond linker design, reaction conditions can significantly influence the assembly process and resulting topology. Even if using the same starting materials, solvent choice^25^ and/or the concentration^26^ used can drastically shift the equilibrium towards different topologies by either enhancing or suppressing inter-/intramolecular interactions, respectively.^26*a*^

These examples, however, predominantly focus on the formation of the thermodynamic product. While many examples support the thermodynamically controlled formation of imine cages, some observations suggest that they may instead be kinetically controlled products, especially when precipitating from the reaction mixture.^12*d*,25*b*,27,28^ This can be rationalised by considering the potential energy surface of the system, where the cage structure may correspond to a kinetically trapped state rather than the global thermodynamic minimum, and cage formation can be driven by precipitation, preventing further equilibration towards more stable assemblies, highlighting the complexity of controlling self-assembly.

## Results and discussion

### Synthesis and characterisation of the cages

**Fig. 2:**
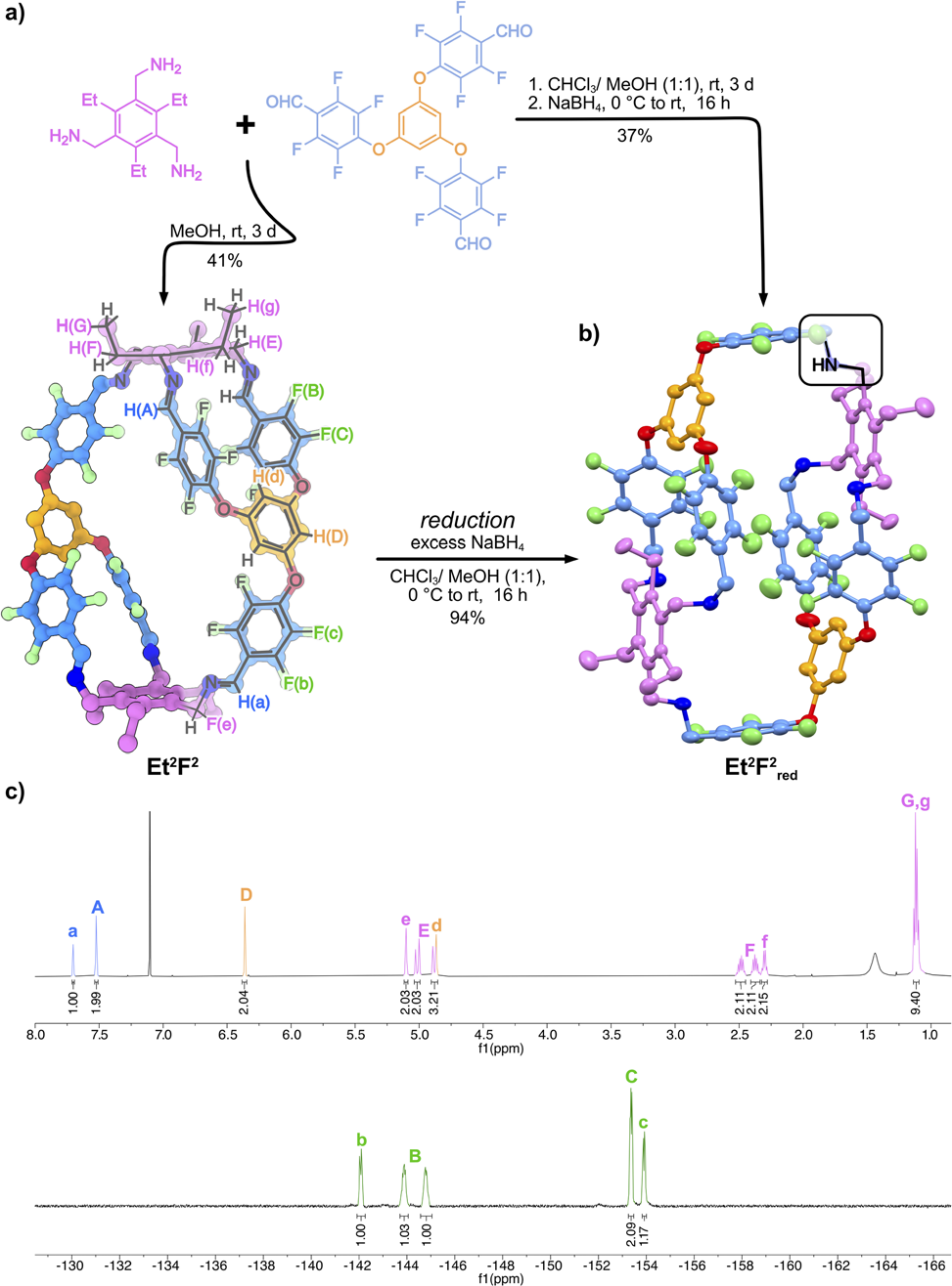
(a) Synthetic route towards imine cage Et^2^F^2^ and the respective amine cage Et^2^F^2^_red_; (b) the SC-XRD structure of Et^2^F^2^_red_ with thermal ellipsoids set at 50% probability, hydrogens and solvents were omitted for clarity; (c) ^1^H and ^19^F NMR (600 MHz/282 MHz, CDCl_3_, 298 K) spectra of the imine cage Et^2^F^2^ showing the splitting of all signals due to the reduced symmetry.

Herein, we report the synthesis of three novel cages of the unique Tri^2^_2_Tri^2^ geometry, including topological assembly control and the complexation of per- and polyfluoroalkyl substances (PFAS) in organic media and their removal from aqueous solutions. For that purpose, we chose the flexible aldehyde F, offering the combination of an electron-rich core and electron-deficient fluorinated panels, aiming for a Janus-type nanocavity to facilitate the intermolecular host–guest interactions.^12*c*^ Stirring (2,4,6-triethylbenzene-1,3,5-triyl)trimethanamine (Et) with the flexible F in an equimolar ratio for 3 days in methanol at room temperature resulted in the precipitation of a colourless solid. To our surprise, both the ^1^H and ^19^F NMR of a redissolved sample showed a complex set of signals (Fig. 2c), whereas, on the contrary, MALDI-MS only showed a singular signal belonging to a condensation of two F with two Et building blocks. The lower *C*_2h_ symmetry of the obtained cage Et^2^F^2^ results in a splitting of the observed resonance signals, showing a 2 : 1 ratio for all signals, which is in good accordance with the ratio expected for a cage of Tri^2^_2_Tri^2^ geometry. This unrepresented cage topology distinguishes itself through its lowered symmetry as a result of the two double connections found within its structure (Fig. 2a).^11*b*^ The resulting inherent strain and rigidity of the assembly cause a diastereotopic splitting of the protons H_E_ and H_F_ belonging to the double-connected amine motif (Fig. 2c).

Intrigued by these results, we investigated different conditions for the cage formation, aiming for either thermodynamic or kinetic control over the assemblies. For that purpose, we chose chloroform as solvent, heating to 60 °C to allow all intermediates to remain in solution, over time reaching the thermodynamic equilibrium. For experiments under kinetic conditions, we investigated the two most common “poor solvents” (lower solubility of intermediates and products), acetonitrile and methanol, to induce precipitation of possible intermediate structures. The respective mixtures were stirred at room temperature upon dropwise addition of a solution of the respective amine.^27^

Using acetonitrile as the solvent, stirring at room temperature for 3 days again selectively led to the formation of the Tri^2^_2_Tri^2^ species Et^2^F^2^ precipitating from the reaction solution. Stirring Et and F in chloroform at 60 °C for three days resulted in the formation of a second, more symmetric species, without any precipitation occurring. MALDI-MS of the obtained mixture revealed the formation of the highly symmetric (*T*_d_) Tri^4^Tri^4^ topology alongside Et^2^F^2^ (see Fig. S1 and S2†). In an attempt to isolate the observed different cage topologies, the dynamic covalent imines were reduced by *in situ* reaction with sodium borohydride.^29^ The respective amine cage Et^2^F^2^_red_ could be isolated in 37% yield from the one-pot two-step reaction of Et and F or in 94% yield from Et^2^F^2^. Again, HRMS (ESI†) and NMR analysis confirmed the formation of Et^2^F^2^_red_, whereas the larger Et^4^F^4^_red_ structure could not be isolated. Et^2^F^2^_red_ readily crystallised from a chloroform solution, and the obtained crystals were subjected to single-crystal X-ray analysis (SC-XRD), unambiguously confirming the anticipated Tri^2^_2_Tri^2^ structure (Fig. 2b). As a result of the two double connections between two singular F and Et motifs, the amine cage is flattened overall, resembling a double-walled macrocycle of *C*_2h_ symmetry, with two different angles for Ar–O–Ar_F_ bonds to accommodate the inherent strain expected with less flexible building blocks.^11*b*^ One ethyl group of the Et motif is located between the fluorinated panels of each double connection. The same is true for the analogous hydrogen of the phloroglucinol motif, explaining the strong upfield shifts observed in ^1^H NMR (Fig. S123,† and 4b, H_f_, H_g_). Meanwhile, all free electron pairs of the amine nitrogen are pointing inwards, forming two main cavities where residual chloroform solvent molecules are located. Overall, even with the increased flexibility of the amine bonds compared to the more rigid imine bonds, the structure still appears potentially strained.

Thus, in addition to Et, the flexible tris(2-aminoethyl)amine (TREN) was investigated in combination with aldehyde F, anticipating that under thermodynamic control, enrichment of the geometry encoded in the linker would be observed, as the rigidity is reduced.

Interestingly, again the Tri^2^_2_Tri^2^ topology was favoured over the larger Tri^4^Tri^4^ species (see Table S1†). Heating the reaction mixture in chloroform led to the almost exclusive formation of TREN^2^F^2^ over TREN^4^F^4^ (96 : 4). In contrast, precipitation from methanol or acetonitrile reaction mixtures favoured the formation of the Tri^4^Tri^4^ species, likely due to the increased solubility of the cages and their intermediates compared to the Et-based counterparts.^27^ This is supported by the observation that in acetonitrile solution, only TREN^4^F^4^ was found in the precipitate, whereas the filtrate contains a mixture of cage topologies similar to those observed in chloroform (acetonitrile filtrate TREN^2^F^2^ : TREN^4^F^4^ ratio 94 : 6). Overall, these findings indicate that aldehyde F preferentially directs the formation of the Tri^2^_2_Tri^2^ cage topology. Under thermodynamic control, this is less pronounced when employing the more rigid amine Et in comparison to TREN, with kinetic conditions exclusively leading to the formation of Et^2^F^2^ alongside insoluble oligomeric species typical for these conditions.^12*d*^ The inverse behaviour of the TREN-based cages is likely a result of two factors; (a) their increased solubility, allowing for a higher proportion of Tri^4^Tri^4^ species to be formed before precipitation occurs, and (b) the distance between the fluorobenzene motifs, which is influenced by the rigidity of the amine building block.

We rationalised that the Tri^2^_2_Tri^2^ topology is directed through small intramolecular interactions between the fluorinated benzene motifs leading to a preorganisation,^30^ where two panels are near to each other in solution leading to a preferred formation of a double connection between one Et molecule and two aldehyde groups of a singular F molecule. This is in line with previous observations where we found that using fluorinated aldehydes can result in the formation of the unusual Tri^6^Di^9^ topology alongside the otherwise strongly favoured, well-known Tri^4^Di^6^ topology.^31^ Additionally, the preferred formation of TREN^2^F^2^ over TREN^4^F^4^ suggests that these interactions are (a) lessened with the more preorganised and less flexible amine Et and (b) play a significant role in the stabilisation of the newly obtained Tri^2^_2_Tri^2^ cage topology.

**Fig. 3:**
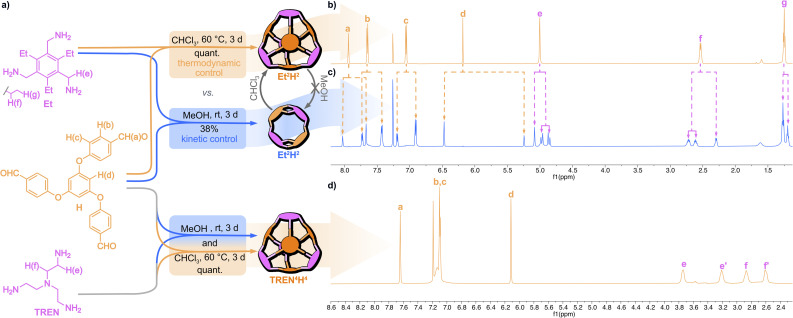
(a) Synthesis of the non-fluorinated cages under either thermodynamic or kinetic control with a schematic representation of the Tri^2^_2_Tri^2^ and Tri^4^Tri^4^ cage geometries and the respective ^1^H NMR (600 MHz, CDCl_3_, 298 K) spectra of (b) Et^4^H^4^, (c) Et^2^H^2^, and (d) TREN^4^H^4^. The signal assignments are shown in (a).

Therefore, we also investigated the non-fluorinated aldehyde derivative H, expecting no significant interactions between its panels. Aldehyde H was readily prepared in a two-step procedure starting from phloroglucinol (see the ESI†). To our delight, the initial screenings under either thermodynamic (in chloroform at 60 °C) or kinetic control (acetonitrile or methanol at room temperature) revealed that the highly soluble and flexible amine TREN exclusively formed the Tri^4^Tri^4^ geometry (Fig. 3). The respective imine cage TREN^4^H^4^ could be isolated from chloroform in quantitative yield (Fig. 3a). As expected for reactions under kinetic control, TREN^4^H^4^ precipitated from the reaction mixture along with insoluble by-products. Similarly, the Et-based cage (Et^4^H^4^) formed quantitatively under thermodynamic control. In contrast, NMR and MALDI-MS analyses of the precipitate from methanol revealed the clean formation of the lower symmetry cage Et^2^H^2^ of Tri^2^_2_Tri^2^ topology (Fig. 3c). Upon extraction from the insoluble by-products, the lower symmetry cage could be isolated in 38% yield.

Monitoring a redissolved sample of Et^2^H^2^ in CDCl_3_ (2.3 mM, consistent with the concentration used for cage synthesis) at either 60 °C or at room temperature resulted in the appearance of a new set of signals. After one day, noticeable amounts of Et^4^H^4^ had formed, whereas at room temperature only marginal amounts of the Tri^4^Tri^4^ species Et^4^H^4^ were observed. Over the course of seven days, the Tri^4^Tri^4^ species became increasingly enriched, and after 24 days, the complete conversion to Et^4^H^4^ was observed under both conditions. At 60 °C, this initial transformation proceeded notably quicker, reaching a 1 : 1 cage ratio already after five days, while at room temperature this was reached only after about nine days (Fig. S14–S16†). Similarly, when stirring Et^2^H^2^ in CDCl_3_ for 3 days at 60 °C, an almost complete cage-to-cage transformation towards Et^4^H^4^ was observed. At room temperature, only small amounts (∼34%) of Et^4^H^4^ were formed. Suspending Et^4^H^4^ in methanol and stirring for three days, even at 60 °C, did not result in any observable interconversion (Fig. S13†).

This underlines the bias towards the larger, highly symmetric, and less internally strained Tri^4^Tri^4^ topology over the low-symmetry Tri^2^_2_Tri^2^ topology in solution,^11*b*^ strongly suggesting that Et^2^H^2^ is an intermediate structure formed on the pathway towards Et^4^H^4^. To rule out that reaction temperature plays a role in this observation, we reacted Et with H in chloroform at room temperature, again selectively forming Et^4^H^4^, while stirring in methanol, even at 60 °C, leading to Et^2^H^2^ as the singular species, strongly suggesting that the solubility of the intermediates is the discriminating factor.

To test this, during cage formation studies, the chloroform content was fixed at 10% to fully dissolve the starting materials, while the acetonitrile concentration was varied. For a methanol/chloroform mixture (90 : 10), Et^2^H^2^ almost exclusively precipitated from the solution. As the acetonitrile content increased, the proportion of Et^4^H^4^ in the precipitate also increased, until at 40% acetonitrile content, only Et^4^H^4^ precipitated from the reaction solution (see Fig. S10 and S11†). This trend aligns with the observed solubility differences of aldehyde H, which is poorly soluble in methanol but highly soluble in acetonitrile. Additionally, the faster precipitation observed in mixtures with lower acetonitrile content (Fig. S12†) suggests that (a) the solubility of intermediates in the respective solvents can be roughly estimated from the solubility of the employed building blocks and (b) the primary factor driving the formation of Et^2^H^2^ is precipitation.^25*b*^ These findings highlight that, under kinetic control, a mixture of species might precipitate, but adjusting the solvent composition can dramatically shift the equilibrium. Thus, to achieve a desired outcome, the solubility of the building blocks should be carefully considered when selecting the solvent for self-assembly reactions.

### PFOA removal

With the understanding of the dynamic covalent chemistry behind the formation of the Tri^2^_2_Tri^2^ cage topology, we shifted our focus back to the fluorinated Tri^2^_2_Tri^2^ cages. As previously shown, the imine cages could be easily reduced *in situ* to the respective amine cages Et^2^F^2^_red_ and TREN^2^F^2^_red_ with sodium borohydride. Additionally, Et^2^F^2^_red_ with the electron-rich cores with fluorinated, electron-deficient panels, featuring exposed amine groups, was investigated for the uptake of perfluorinated octanoic acid (PFOA) and trifluoroacetic acid (TFA). Per- and polyfluoroalkyl substances (PFAS), or “forever chemicals,” are synthetic compounds used in industry for their thermal stability and resistance to degradation. Found in coatings, foams, and textiles, they persist in the environment, accumulate in organisms, and pose serious health risks.^32^ Despite restrictions, PFAS contamination remains a major global challenge due to their removal complexity.^33^ Supramolecular chemistry approaches have been employed to tackle the complex challenge of sieving PFAS molecules, which exhibit both hydrophilic and lipophilic behaviour, such as leveraging the rich host–guest chemistry of supramolecular compounds^34^ including cyclodextrin derivatives,^35*d*^ water-soluble iron-^35*e*^ or palladium-based,^35*a*^ and insoluble Zr-based^35*b*^ MOCs. Recently, Sessler and Chi *et al.* used a water-insoluble fluorinated amine-based cage feasible for the removal of PFOA from aqueous solutions through the reduction of the respective dynamic covalent imine cage.^35*c*^ Contrary to the binding of neutral compounds, *e.g.*, perfluorocarbons (PFCs), which is primarily governed by hydrophobic effects and π-stacking within nonpolar, shape-complementary cavities,^36^ the binding of PFAS typically relies on an interplay of electrostatic and fluorophilic interactions by using the polar headgroup as an anchor.^35*c*–*e*^

First, we performed NMR titrations in organic media (chloroform/methanol mixture 95 : 5) at a concentration of 2.5 mM, investigating the cage's ability to capture PFOA and to study present interactions. Additionally, the symmetric “open-cavity” model compound Bn^3^F^1^_red_ was prepared for the comparison with the “closed-cavity” cages. Bn^3^F^1^_red_ was synthesised by condensation of aldehyde F with benzylamine, followed by *in situ* reduction with sodium borohydride.

**Fig. 4:**
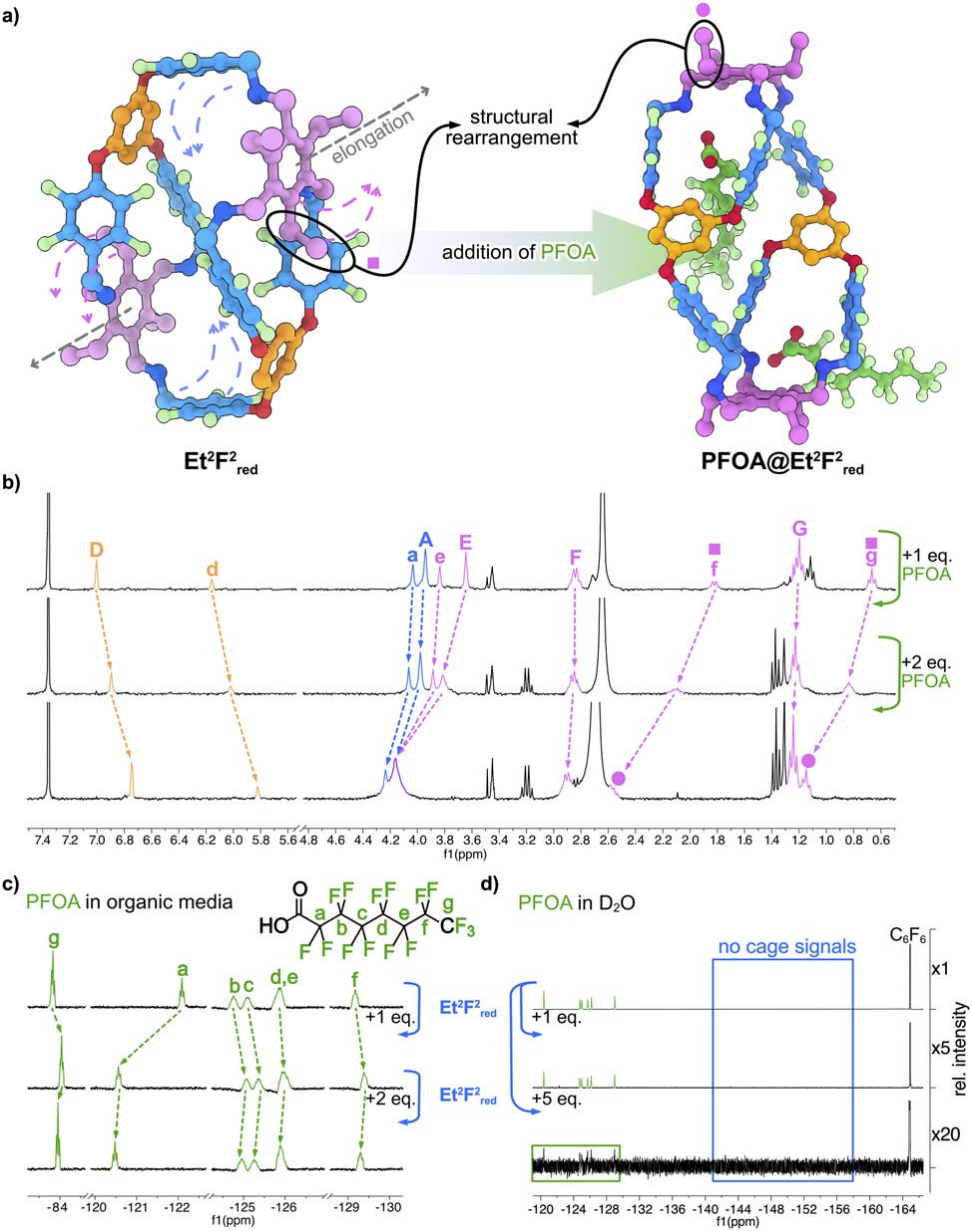
(a) Schematic illustration of the PFOA-induced structural rearrangement in Et^2^F^2^_red_, where one ethyl group (pink square) of the Et motif is pushed away from the two neighbouring fluorobenzenes of the F motif; (b) stacked ^1^H NMR spectra (300 MHz, CDCl_3_/MeOD, 95 : 5, 2.5 mM, 298 K), highlighting the chemical shifts in Et^2^F^2^_red_ upon the addition of PFOA, signal assignment analogous to Fig. 2a; (c) stacked ^19^F NMR spectra (282 MHz, CDCl_3_/MeOD, 95 : 5, 2.5 mM, 298 K), highlighting the chemical shifts of PFOA upon the addition of Et^2^F^2^_red_; (d) stacked ^19^F NMR spectra (565 MHz, 128 scans, D_2_O, 298 K) showing the removal of PFOA from an aqueous solution using Et^2^F^2^_red_ as a heterogenous adsorbent.

Upon addition of the cages to a PFOA solution, shifts for all PFOA signals could be observed in ^19^F NMR spectra. The CF_2_-group (F_a_) neighbouring the carboxylic acid exhibited the most significant shift of 1.6–1.8 ppm, while the other signals, even including the terminal CF_3_-group (F_g_), were roughly shifted by −0.2 ppm (see ESI†). For the symmetric model compound Bn^3^F^1^_red_, however, only marginal shifts of (<0.02 ppm) for all signals besides F_a_ were noticeable. The addition of PFOA to the respective hosts led to a shifting of all host signals in ^1^H and ^19^F NMR, with the CH_2_–NH–CH_2_ motif expressing the strongest shifts in ^1^H NMR, while other signals were only slightly shifted (<0.05 ppm) in the case of TREN^2^F^2^_red_ and Bn^3^F^1^_red_. Et^2^F^2^_red_ showed noticeable downfield shifts for almost all signals. Most interestingly, the protons H_f_ and H_g_ were strongly downfield shifted by 0.78 ppm and 0.48 ppm, respectively, until almost overlaying with the other ethyl groups (H_F_ and H_G_) of the Et motif. Simultaneously, both phloroglucinol protons H_D_ and H_d_ shifted upfield, indicating that Et^2^F^2^_red_ undergoes a structural rearrangement. Upon addition of PFOA, the singular ethyl group of the Et located between the double-connected fluorinated benzenes of F is supposedly pushed outwards, away from the fluorinated panels, being deshielded in the process. This assumption is supported by ^1^H–^1^H NOESY NMR data, which reveal that upon the addition of PFOA, the previously isolated ethyl group exhibits a new correlation pattern similar to that observed for the other ethyl groups (Fig. S75†). A similar structural change can be observed in the solid-state. When comparing the crystal structures of PFOA@Et^2^F^2^_red_ (grown from a dichloromethane/acetonitrile solution) and Et^2^F^2^_red_ (Fig. S17–S19†). PFOA@Et^2^F^2^_red_ shows a more elongated cage structure, where the ethyl groups are all pointing away from the fluorobenzenes and two PFOA molecules are located outside of the cage (Fig. 4a). The addition of a strong acid like trifluoroacetic acid did not result in a significant shifting of these signals, instead, a broadening of the signals is observed (Fig. S50 and S51†). When titrating octanoic acid, of comparable structure and with a p*K*_a_ of 3.8 ± 0.1 (*vs.* 2.2 ± 0.2)^37^ no shifting of the cage signals can be observed (Fig. S45 and S46†), rendering the observed structural rearrangement of Et^2^F^2^_red_ to be selective towards PFOA, not being a result of simple protonation. ^19^F DOSY experiments unambiguously confirmed the formation of a complex between PFOA and Et^2^F^2^_red_ in organic media, showing almost identical diffusion coefficients (3.68 × 10^−10^ m^2^ s^−1^ for PFOA and 3.81 × 10^−10^ m^2^ s^−1^ for Et^2^F^2^_red_) corresponding well to the one observed for pure cage (3.64 × 10^−10^ m^2^ s^−1^, see Table S3†).

The observed ^19^F NMR shifts suggest that PFOA binding in organic media arises from a combination of electrostatic and fluorophilic interactions. Binding is primarily driven by electrostatic attraction between the carboxylic acid group of PFOA and the protonated, quaternary amines of the hosts. However, only the cages show additional interactions, as indicated by pronounced shifts of the PFOA signals, particularly of the terminal CF_3_ group. This points to a binding mode in which the perfluoroalkyl chain remains largely outside the cage, while the CF_3_ group partially inserts into the cage windows and interacts with the fluorinated panels. This interpretation is in good accordance with the preliminary SC-XRD data. Overall, this fluorophilic interaction appears to be weak and of dynamic nature, with the apparent encapsulation resulting mainly from spatial proximity rather than strong inclusion (see the ESI† for a detailed discussion). These findings are consistent with studies on COFs,^38^ porous polymers^39^ and macrocycle-based hydrogels,^40^ where quaternisation of amine groups enhances PFAS uptake from aqueous solutions through an interplay of electrostatic and hydrophobic effects.

Encouraged by these promising observations, we investigated the ability of these highly hydrophobic cages to remove PFOA from aqueous solution. A 1 mg mL^−1^ solution of PFOA in deionised water was prepared, to which 1 equivalent of the completely insoluble cages was added. After stirring the colourless suspension for one hour, the mixture was filtered through a syringe filter, and the clear filtrate was analysed by ^19^F NMR. To our delight, 1 equivalent of the cages already removed approximately 80% of the initial PFOA amount. Upon addition of 5 equivalents, no clear signals corresponding to PFOA or the cage were detected, indicating an almost complete removal (Fig. 4d). These results highlight the potential of these cages as heterogeneous, low-molecular-weight adsorbent materials for PFOA removal.

## Conclusions

In conclusion, we were able to gain access to an unrepresented cage geometry of the Tri^2^_2_Tri^2^ topology using only highly symmetric building blocks. This was accomplished by leveraging either thermodynamic or kinetic control over the self-assembly process. As building blocks, a fluorinated and a non-fluorinated aldehyde alongside one of two amines with differing degrees of preorganisation, flexibility, and solubility were used.

Investigations revealed that the fluorinated linker favoured the Tri^2^_2_Tri^2^ cage topology under thermodynamic control, whereas the non-fluorinated linker H exclusively formed the larger symmetric Tri^4^Tri^4^ derivatives under these conditions. Applying kinetic control allowed for the selective formation of the low-symmetry Tri^2^_2_Tri^2^ cages that precipitated from the reaction mixtures. Studies conducted strongly suggest that the flexibility of the building blocks plays a crucial role, enabling the formation of the Tri^2^_2_Tri^2^ species as an intermediate towards larger structures. This understanding allowed us to use solvent selection to direct the assembly pathway, enabling the formation of either the intermediate low-symmetry Tri^2^_2_Tri^2^ cages under kinetic control or the larger high-symmetry Tri^4^Tri^4^ structures under thermodynamic control.

Additionally, the reduction of the fluorinated Tri^2^_2_Tri^2^ cages led to the low-symmetry Janus-like cages Et^2^F^2^_red_ and TREN^2^F^2^_red_, which demonstrated promising potential for the removal of PFOA from aqueous solutions. Even in organic solvents, Et^2^F^2^_red_ indicated interactions selectively with PFOA, undergoing a structural rearrangement to accommodate PFOA.

Our findings highlight the delicate aspects of self-assembly pathways in directing cage assembly and provide new insights into the use of fluorinated and non-fluorinated linkers to tailor structural outcomes. This approach expands the toolbox of supramolecular chemists, offering a new route to design cage-like compounds with enhanced structural complexity, paving the way for enzyme-like complex host–guest structures and advanced lightweight functional materials.

## Author contributions

The study was conceptualised by T. P. and B. M. S. The majority of experiments were conducted by T. P., including the synthesis and characterisation, assisted by P. M. M. P. M. M. and F. Z. performed the titration experiments. A. M. was responsible for ^19^F DOSY NMR measurements and data analysis, B. M. S. conducted SC-XRD data acquisition and refinement. The first draft of the manuscript, review, editing, and data presentation was executed by T. P. and B. M. S. All authors approved the final submission.

## Conflicts of interest

There are no conflicts to declare.

## Supplementary Material

SC-OLF-D5SC02247A-s001

SC-OLF-D5SC02247A-s002

## Data Availability

The data supporting this article have been included as part of the ESI.† Crystallographic data for Et^2^F^2^_red_ (2430764) has been deposited in the joint Cambridge Crystallographic Data Centre (CCDC) and Fachinformations-zentrum Karlsruhe Access Structures service, available free of charge.
