# Supplementary material for: Structural control of dynamic covalent cages: kinetic vs. thermodynamic assembly and PFAS removal from water
Source: Chem Sci. 2025 Jun 18;16(29):13221–8. doi: 10.1039/d5sc02247a (PMC12188467; doi:10.1039/d5sc02247a)
Supplement: SC-016-D5SC02247A-s001 [file SC-016-D5SC02247A-s001.pdf]

Supplementary Information (SI) for Chemical Science.
This journal is © The Royal Society of Chemistry 2025

Contents

I. General 2

III. Experimental Details 3

- **I.a. Synthesis of building blocks** 4
- **III.b. Synthesis of the fluorinated cages** 6
- **III.c. Synthesis of the non-fluorinated cages** 12

IV. Cage formation studies 15

- **IV.a. Cage formation studies under thermodynamic and kinetic control** 15
- **IV.b. Extended cage formation studies of Et^XH^X** 21
- **IV.c. Extended formation studies of Et^XH^X in solvent mixtures:** 22

V. Cage-to-Cage transformation of Et^2H^2 and Et^4H^4 24

VI. SC-XRD 27

VII. DOSY NMR 33

- **VII.a. ^{1}H-DOSY NMR** 33
- **VII.b. ^{19}F-DOSY NMR** 38

VIII. NMR Titrations 44

- **VIII.a Titrations of $Et^2F^2_{red}$** 47
- **VIII.b. Titrations of $TREN^2F^2_{red}$** 54
- **VIII.c. Titrations of $Bn^3F^1_{red}$** 60
- **VIII.d. 2D-NMR studies of $Et^2F^2_{red}$ with PFOA** 66

IX. PFOA removal 69

X. Spectra 72

XI. References 106

I. General

Chemicals and solvents were purchased from Sigma-Aldrich, VWR/Merck, and Tokyo Chemical Industry and were used without further purification. Nitrile **2** and amine **Et** were synthesised according to literature procedures,[S1-2] full synthetic scheme for **Et** is shown in Scheme S1. HPLC-grade acetonitrile was used as received. Dry dichloromethane, tetrahydrofuran, and dioxane were obtained by a solvent purification system from MBraun (SPS-800). Reactions were monitored by thin layer chromatography (TLC), using silica gel plates from Macherey Nagel (ALUGRAM® Xtra SIL G/UV254). Column chromatography was done with silica gel from Macherey Nagel (Silica 60 M, 0.04–0.063 mm). The solvents were removed under reduced pressure by using a rotary evaporator at 50 °C, if not stated otherwise.

Reactions which had to be done under complete exclusion of water were prepared by drying the laboratory glassware and the stirring bar at 80 °C for several hours. The respective reactions were run using Schlenk techniques for working under an inert atmosphere of nitrogen or argon.

II. Analytics

NMR measurements

The measurements of ^{1}H NMR, ^{19}F NMR, ^{13}C{^{1}H} NMR spectra,^{1}H DOSY and 2D-spectra were recorded on a Bruker Avance III – 300 (^{1}H NMR: 300 MHz, ^{19}F NMR: 282 MHz, ^{13}C{^{1}H} NMR: 75 MHz), Bruker Avance III – 600 and Bruker Avance NEO 600 (^{1}H NMR: 600 MHz, ^{19}F NMR: 564 MHz, ^{13}C{^{1}H} NMR: 150 MHz) NMR-spectrometers. ^{19}F DOSY spectra were recorded on a Bruker Avance NEO 600 spectrometer at 20 °C. ^{1}H-^{1}H NOESY spectra were measured on a Bruker Avance NEO 600 spectrometer (^{1}H NMR: 600 MHz). The ^{1}H-^{19}F HOESY and ^{19}F-^{19}F NOESY experiments were performed on a Bruker Avance 400 spectrometer (^{1}H NMR: 400 MHz, ^{19}F {^{1}H } NMR: 376 MHz) at 298 K. All samples were dissolved in deuterated solvents.

IR

Infrared spectra were measured with an FT/IR-6200 of the company JASCO and FT/IR IRAffinity-1 with ATR attachment of the company Shimadzu.

Mass spectrometry

Matrix-assisted Laser Desorption/Ionisation mass spectrometry was performed on a MALDI-TOF/TOF UltrafleXtreme (Bruker Daltonics) using dithranol as matrix. High-resolution electrospray ionisation mass spectrometry was performed on a UHR-QTOF maXis 4G (Bruker Daltonics) instrument.

III. Experimental Details

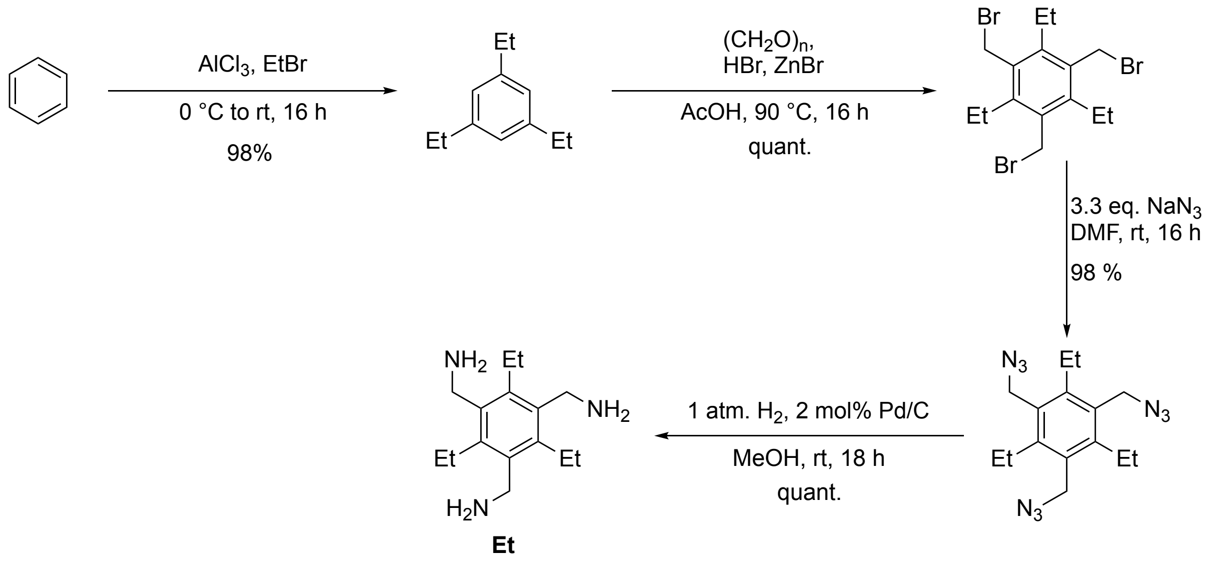

Scheme S1: 1,3,5-Triethylbenzene, 1,3,5-tri(bromomethyl)-2,4,6-triethylbenzene, and 1,3,5-tris(azido-methyl)-2,4,6-triethylbenzene were obtained following literature procedures. (2,4,6-Triethylbenzene-1,3,5-triyl)-trimethanamine (**Et**) was obtained by a slightly modified literature procedure, exchanging the solvent (ethanol) for methanol. All the analytical data obtained were in accordance with the literature.[S2]

I.a. Synthesis of building blocks

Synthesis of the fluorinated linker (F)

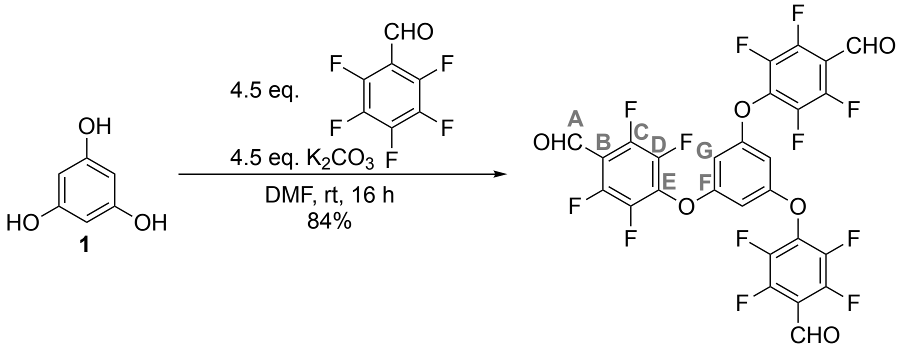

F

A 100 mL Schlenk flask was charged with phloroglucinol **1** (631 mg, 5.00 mmol), potassium carbonate (3.11 g, 22.5 mmol), and 50 mL of dry DMF. The resulting suspension was stirred for 30 minutes under N_2 atmosphere. Afterwards, pentafluorobenzaldehyde (2.7 mL, 22.5 mmol) was added, turning the solution into a dark mixture. The reaction was let to stir additionally for 16 h under an inert atmosphere at room temperature. After that time, the reaction mixture was diluted with ethyl acetate and filtered to remove the remaining potassium carbonate, and the solvent was removed *in vacuo*. The crude product was purified by silica gel column chromatography, with cyclohexane/ethyl acetate (3:1 v/v) as eluent, to afford the desired compound **F** as a pale-yellow powder (2.76 g, 4.22 mmol, 84%).

^{1}H NMR (600 MHz, $CDCl_3$): δ = 10.30 (s, 3H, **H_A**), 6.49 (s, 3H, **H_B**); **^{19}F NMR** (282 MHz, $CDCl_3$): δ = - 143.75 – -143.94 (m, 6F, **F_C**), -151.97 – -152.31 (m, 6F, **F_D**); **^{13}C{^{1}H} NMR** (151 MHz, $CDCl_3$): δ = 181.77 (**C_A**), 158.32 (**C_F**), 150.45 – 145.56 (m, **C_C**), 141.20 (ddd, *J* = 256.1, 14.3, 3.4 Hz, **C_D**), 137.68 (t, *J* = 12.6 Hz, **C_E**), 112.38 (t, *J* = 9.6 Hz, **C_B**), 100.87 (s, **C_G**); **IR** (cm^{-1}): 2974.23 (w), 288.58 (w), 1705.07 (m), 1649.14 (w), 1604.77 (m), 1487.12 (s), 1465.90 (m), 1396.46 (m), 1305.81 (w), 1151.50 (s), 1018.41 (m), 981.41 (m), 947.05 (w), 927.76 (w), 831.32 (w), 808.17 (w), 752.24 (w), 659.66 (w), 628.79 (m); **HRMS (ESI)**: m/z calculated for $[C_{27}H_6F_{12}O_6+Na]^+$ = 676.9870, found m/z = 676.9869.

Synthesis of the non-fluorinated linker (H)

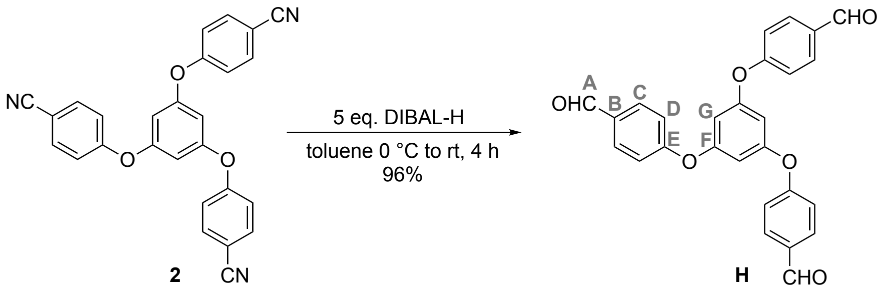

Nitrile **2** (283 mg, 660 µmol, 1.00 eq.) was dissolved in 40 mL dry toluene, and a 1.5 M (25 wt%) solution of DIBAL-H in toluene (2.2 mL, 3.3 mmol, 5.0 eq.) was added dropwise over a period of 10 minutes at 0 °C. After complete addition, the resulting mixture was allowed to reach room temperature and was stirred for an additional 4 hours (or until a full conversion could be observed by TLC). The reaction was quenched by the addition of 10 mL of ethyl acetate and 20 mL of 2 M hydrochloric acid. The organic phase was separated, and the aqueous phase was extracted with dichloromethane (2 x 100 mL). The combined organic phases were dried over Na_2SO_4, and the solvent was evaporated under reduced pressure to yield **H** (276 mg, 630 µmol, 96%) as a pale-yellow wax-like solid, which completely solidified over two days.

^{1}H NMR (600 MHz, $CDCl_3$): δ = 9.93 (s, 1H, **H_A**), 7.88 (d, *J* = 8.1 Hz, 2H, **H_C**), 7.14 (d, *J* = 8.1 Hz, 2H, **H_D**), 6.60 (s, 1H, **C_G**); **$^{13}C\{^1H\}$ NMR** (151 MHz, $CDCl_3$): δ = 190.67 (**C_A**), 161.57 (**C_F**), 158.18 (**C_E**), 132.40(**C_B**), 132.15(**C_C**), 118.68(**C_D**), 107.26 (**C_G**); **IR** (cm^{-1}): 2229.71 (w), 1618.28 (w), 1589.34 (s), 1494.83 (s), 1448.54 (m), 1411.89 (w), 1219.01 (s), 1165.00 (s), 1132.21 (m), 1116.78 (m), 1002.98 (s), 956.69 (w), 850.61 (m), 823.60 (m), 731.02 (w), 692.44 (w), 677.01 (w); **HRMS (ESI)**: m/z calculated for $[C_{27}H_{18}O_6+H]^+$= 439.1176, found m/z = 439.1175.

Note: If necessary, the product can be purified further by column chromatography using cyclohexane/ ethyl acetate (4:1) as eluent.

III.b. Synthesis of the fluorinated cages

Synthesis of imine cage Et^2F^2

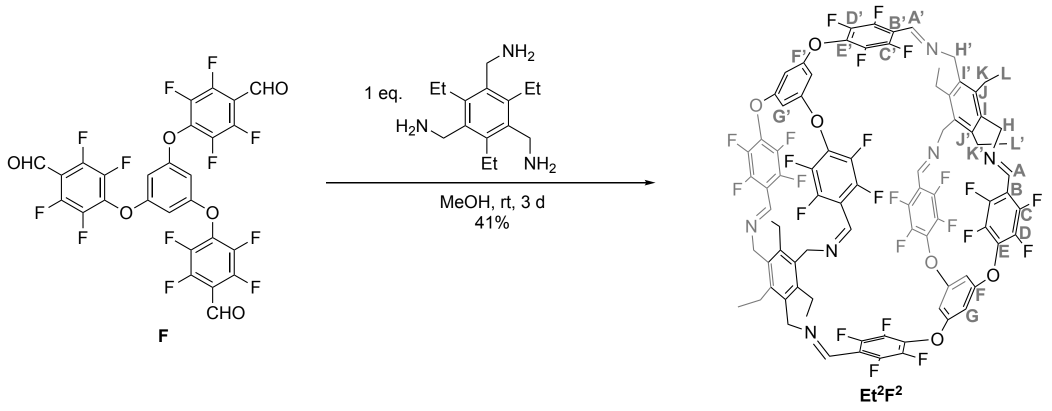

Aldehyde **F** (131 mg, 250 µmol, 1.00 eq.) was suspended in 25 mL of methanol, and a solution of **Et** (50 mg, 250 µmol, 1.00 eq.) in 25 mL methanol was added dropwise over the course of 4 h. During the addition, the suspension transformed into a pale-yellow solution and then back to a colourless suspension. The mixture was stirred for 3 days at room temperature. Afterwards, the precipitate was filtered off, washed with 2 x 5 mL methanol, and the resulting solid was extracted with 100 mL dichloromethane by sonication. Filtration followed by the addition of 200 mL *n*-hexane and the removal of the solvent yielded the pure imine cage **Et^2F^2** as a colourless powder (70.0 mg, 41.2 µmol, 41%).

When using acetonitrile as solvent, the cage was obtained in 20% yield.

^{1}H NMR (600 MHz, $CDCl_3$): δ = 7.86 (s, 1H, **$H_{A'}$**), 7.68 (s, 2H, **H_A**), 6.52 (d, *J* = 2.0 Hz, 2H, **H_G**), 5.26 (s, 2H, **$H_{H'}$**), 5.17 (d, *J* = 16.4 Hz, 2H, **H_H**), 5.08 – 5.00 (m, 3H, **$H_{H,G'}$**), 2.64 (dq, *J* = 14.8, 7.4 Hz, 2H, **H_K**), 2.53 (dq, *J* = 14.7, 7.4 Hz, 2H, **H_K**), 2.46 (q, *J* = 7.4 Hz, 2H, **$H_{K'}$**), 1.30 – 1.23 (m, 9H, **$H_{L,L'}$**); **^{19}F NMR** (282 MHz, $CDCl_3$): δ = -142.08 (dd, *J* = 20.6, 9.8 Hz, 1F, **$F_{C'}$**), -143.88 (dd, *J* = 20.3, 9.9 Hz, 1F, **F_C**), -144.52 – -145.02 (m, 1F, **F_C**), -153.39 (dd, *J* = 20.3, 8.6 Hz, **F_D**), -153.91 (dd, *J* = 20.0, 9.6 Hz, **$F_{D'}$**); **^{13}C{^{1}H} NMR** (151 MHz, $CDCl_3$): δ = 159.14 (**$C_{F'}$**), 158.76 (**C_F**), 149.24 (**$C_{A'}$**), 149.03 (**C_A**), 147.50 – 144.98 (m, **C_C**), 146.05 – 143.94 (m, **$C_{C'}$**), 144.44 (**C_J**), 143.87 (**$C_{J'}$**), 142.61 – 140.50 (m, **C_D**), 141.81 – 139.70 (m, **$C_{D'}$**), 133.34 (**C_I**), 133.17 (t, *J* = 13.7 Hz, **C_E**), 113.17 (t, *J* = 12.2 Hz, **C_B**), 112.89 (t, *J* = 11.1 Hz, **$C_{B'}$**), 99.42(**C_G**), 92.20 (**$C_{G'}$**), 56.64 (**$C_{H'}$**), 56.27 (**C_H**), 23.63 (**C_K**), 23.38 (**$C_{K'}$**), 16.40 (**C_L**), 16.36 (**$C_{L'}$**); **IR** (cm^{-1}): 2964.59 (w), 2929.87 (w), 2904.80 (w), 1645.28 (w), 1606.70 (m), 1487.12 (s), 1460.11 (m), 1415.75 (w), 1379.10 (w), 1300.02 (w), 1261.45 (w), 1232.51 (w), 1170.79 (m), 1159.22 (m), 1101.35 (w), 1002.98 (m), 979.84 (m), 918.12 (w), 837.11 (w), 802.39 (m), 719.45 (w), 665.44 (w); **HRMS (ESI)**: m/z calculated for $[C_{84}H_{54}F_{24}N_6O_6+H]^+$ = 1699.3794, found m/z = 1699.3779.

Synthesis of fluorinated imine cage TREN^{2}F^2

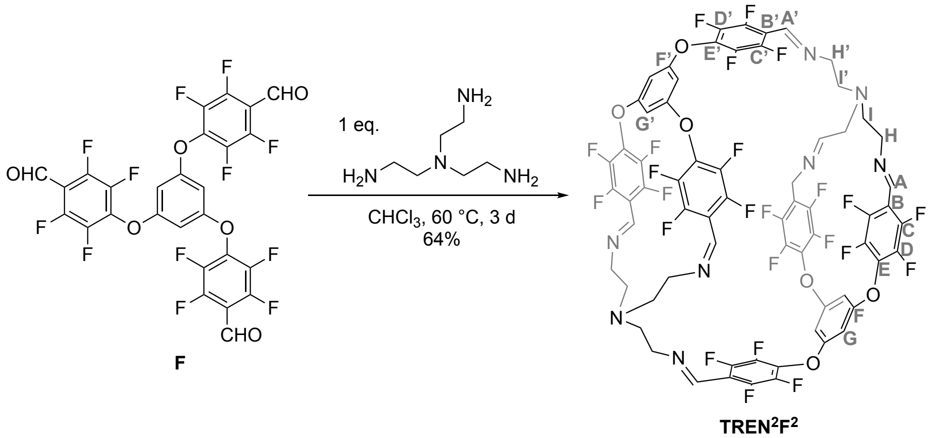

Aldehyde **F** (33 mg, 50 µmol, 1.0 eq.) was dissolved in 10 mL chloroform, and amine **TREN** (7.5 µL, 50 mmol, 1.0 eq.) was added. The reaction flask was capped, and the mixture was stirred at 60 °C for 3 days. Afterwards, the clear solution was filtered, the flask and filter paper were rinsed with 5 mL chloroform, and 20 mL *n*-hexane were added to the resulting filtrate. The resulting clear solution was then concentrated to roughly 10 mL, forming a pale-yellow precipitate. After an additional addition of 20 mL *n*-hexane, the suspension was filtered, yielding the pure imine cage **TREN^{2}F^2** as a pale-yellow powder (24 mg,16 µmol, 64%.).

*The cage **TREN^{2}F^2** was isolated with small amounts of **TREN^{4}F^4** as a side product, which could not be separated due to the dynamic nature of these compounds. In ^{19}F NMR, the signals belonging to **TREN^{2}F^2** appeared very broad in CDCl$_3$. Using MeCN-d$_3$ as the solvent slightly sharpened these signals. Contrary, the signals corresponding to **TREN^{4}F^4** were observed to be sharp in both cases and are marked with an asterisk. Due to the low solubility of the cage in MeCN-d$_3$, the spectral resolution is low, leading to imperfect integrals, as seen for the sharp signals of **TREN^{4}F^4**.*

^{1}H NMR (600 MHz, CDCl$_3$): δ = 8.45 (s, 1H, **H$_{A'}$**), 8.34 (s, 2H, **H$_A$**) 6.60 (s, 2H, **H$_G$**), 5.22 (s, 1H, **H$_{G'}$**), 3.87 (s, 6H, **H$_{I,I'}$**), 2.88 (s, 4H, **H$_H$**), 2.83 (s, 2H, **H$_{H'}$**); **^{19}F NMR** (565 MHz, CD$_3$CN): δ = -144.66 (bs, 2F, **F$_C$**), -144.89 (bs, 1F, **F$_{C'}$**), -145.31 – -145.51 (m, 1.2F, **F$_{C*}$**), -155.72 – -156.45 (m, 2F, **F$_{D,D'}$**), -156.83 (dd, *J* = 20.0, 8.2 Hz, 0.7F, **F$_{D*}$**); **^{13}C{^{1}H} NMR** (151 MHz, CDCl$_3$): δ = 158.70 (**C$_{F'}$**), 158.61(**C$_F$**), 150.94 (**C$_{A'}$**), 150.67 (**C$_A$**), 147.18 – 145.27 (m, **C$_C$**), 145.76 (d, *J* = 252.2 Hz, **C$_{C'}$**), 141.24 (d, *J* = 254.0 Hz, **C$_{D,D'}$**), 133.93 – 133.58 (m, **C$_E$**), 133.26 – 132.78 (m, **C$_{E'}$**), 113.28 (t, *J* = 13.0 Hz, **C$_{B'}$**), 112.66 (t, *J* = 10.7 Hz, **C$_B$**), 99.58 (**C$_G$**), 93.78(**C$_{G'}$**), 60.11 (**C$_H$**), 58.81 (**C$_{H'}$**), 53.84 (**C$_I$**), 53.67 (**C$_{I'}$**); **IR** (cm^{-1}): 1645.28 (w), 1606.70 (m), 1487.12 (s), 1460.11 (m), 1417.68 (w), 1381.03 (w), 1303.88 (w), 1155.36 (s), 1020.34 (m), 983.70 (m), 840.96 (w), 185.03 (w), 669.30 (m); **HRMS (ESI)**: m/z calculated for [$C_{66}H_{36}F_{24}N_8O_6$+2H]$^{2+}$ = 747.1260, found m/z = 747.1266.

Synthesis of the cage $Et^2F^2_{red}$

Method A:

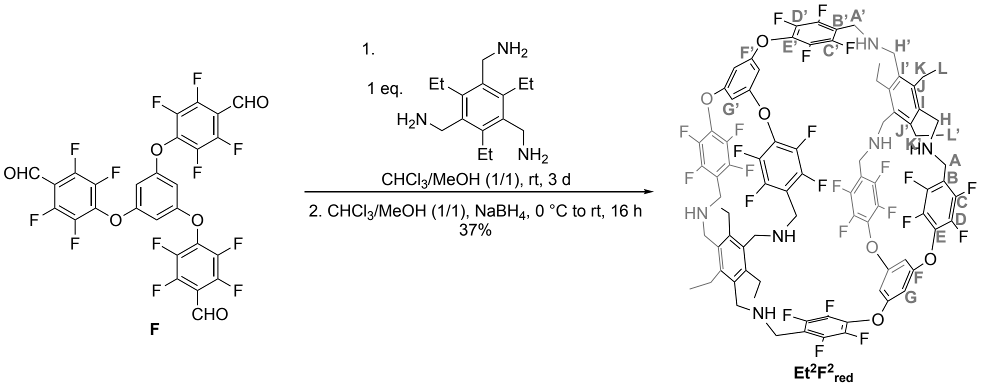

Aldehyde **F** (654 mg, 1.00 mmol, 1.00 eq.) was dissolved in 125 mL of chloroform/ methanol (1:1 v/v), to which a solution of **Et** (249 mg, 1.00 mmol, 1.00 eq.) in 125 mL of chloroform/methanol (1:1 v/v) was added dropwise over the course of 4 h. The resulting solution was stirred for three days at room temperature, during which the reaction was monitored by ^{19}F NMR. Upon completion, the mixture was cooled to 0 ºC using an ice bath, and $NaBH_4$ (1.89 g, 50.0 mmol, 50.0 eq.) was added in small portions over the course of an hour. After the addition, the mixture was allowed to slowly warm up to room temperature and left to react overnight. After that time, the solvent was removed *in vacuo*. To the resulting white solid, 200 mL of a saturated solution of sodium carbonate were added and let to stir for one hour. The aqueous layer was extracted with 3 x 30 mL of chloroform. The resulting organic phase was washed with 100 mL of water, dried over Na_2SO_4, and solvent was removed *in vacuo*. The resulting crude was purified by silica gel column chromatography with dichloromethane/ methanol (95:5 v/v, 1% Et_3N v/v) as eluent to afford the amine cage $\mathbf{Et^2F^2_{red}}$ as a pale-yellow powder (317 mg, 185 µmol, 37%).

Method B:

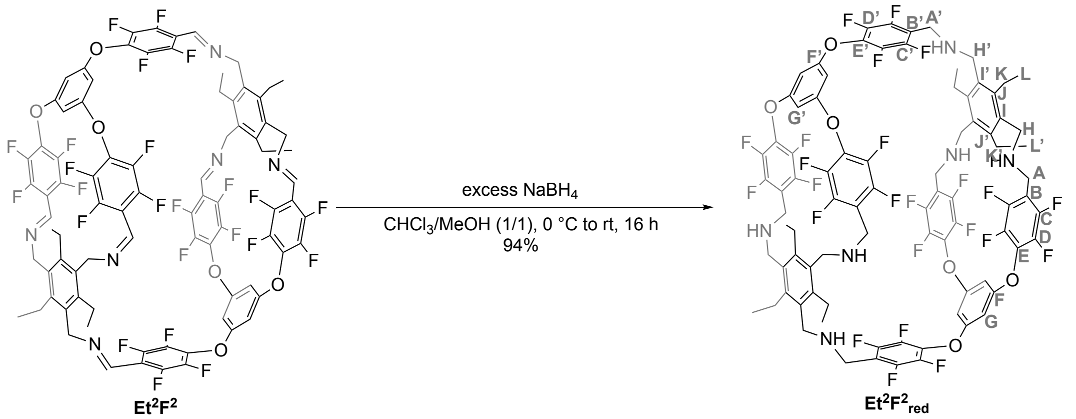

Et^2F^2 **$Et^2F^2_{red}$**

Imine cage **Et^2F^2** (70.0 mg, 41.2 µmol, 1.00 eq.) was dissolved in 100 mL of chloroform/ methanol (1:1 v/v), and the mixture was cooled to 0 ºC using an ice bath. Then $NaBH_4$ (770 mg, 20.4 mmol, 495 eq.) was added in small portions over the course of an hour. After the addition, the mixture was allowed to slowly warm up to room temperature and left to react overnight. The solvent was removed *in vacuo*, the resulting colourless solid was redissolved with 100 mL of 2M hydrochloric acid and stirred for 30 minutes at room temperature. The solution was transferred into a separatory funnel, and the aqueous phase was washed with 2 x 40 mL of chloroform and discarded. Afterwards, the aqueous phase was brought to a pH of 7–8 by the addition of a concentrated aq. KOH solution (8M). The resulting colourless suspension was extracted with 3 x 100 mL of chloroform. The collected organic phases were dried over Na_2SO_4, and solvent was removed *in vacuo* to yield the amine cage **$Et^2F^2_{red}$** as a pale-yellow powder (66.2 mg, 38.7 µmol, 94%).

^{1}H NMR (600 MHz, $CDCl_3$): δ = 6.99 (s, 2H, **H_G**), 6.13 (s, 1H, **$H_{G'}$**), 3.98 (s, 2H, **$H_{A'}$**), 3.91 (s, 4H, **H_A**), 3.79 (s, 2H, HH'), 3.57 (s, 4H, **H_H**), 2.81 (q, *J* = 6.4 Hz, 4H, **H_K**), 1.68 (q, *J* = 7.7 Hz, 2H, **$H_{K'}$**), 1.16 (t, *J* = 7.3 Hz, 6H, **H_L**), 0.57 (t, *J* = 7.2 Hz, 3H, **$H_{L'}$**). ^{19}F NMR (565 MHz, $CDCl_3$) δ -143.89 (dd, *J* = 21.0, 7.6 Hz, 2F, **$F_{C'}$**), -144.31 (dd, *J* = 22.6, 9.4 Hz, 1F, **F_C**), -154.14 (bs, 2F, **F_D**), -154.35 (dd, *J* = 22.6, 9.4 Hz, 1F, **$F_{D'}$**); **^{13}C{^{1}H}** NMR (151 MHz, $CDCl_3$) δ 159.78 (**$C_{F'}$**), 158.42 (**C_F**), 145.47 (d, *J* = 254.0 Hz, **$C_{C,C'}$**), 142.76 (**C_J**), 141.82 (**$C_{J'}$**), 141.72 – 139.79 (m, **$C_{D,D'}$**), 133.92 (**$C_{I'}$**), 133.56 (**C_I**), 115.90 (**$C_{B'}$**), 115.22 (d, *J* = 19.4 Hz, **C_B**), 107.24 (**C_G**), 99.89 (**$C_{G'}$**), 47.27 (**$C_{H'}$**), 45.70 (**C_H**), 41.16 (**$C_{A'}$**), 40.19 (**C_A**), 22.62 (**C_K**), 21.37 (**$C_{K'}$**), 17.01 (**C_L**), 16.02 (**$C_{L'}$**); **IR** (cm^{-1}): 1604.77 (w), 1489.05 (s), 1458.18 (m), 1371.39 (w), 1292.31 (w), 1141.86 (m), 1089.78 (w), 1062.78 (w), 993.34 (m), 974.05 (m), 925.83 (w), 837.11 (w), 740.67 (w), 669.30 (w); **HRMS (ESI)**: m/z calculated for $[C_{84}H_{66}F_{24}N_6O_6+2H]^{2+}$= 856.2403, found m/z = 856.2406.

Synthesis of the cage TREN^{2}F$^2_{red}$

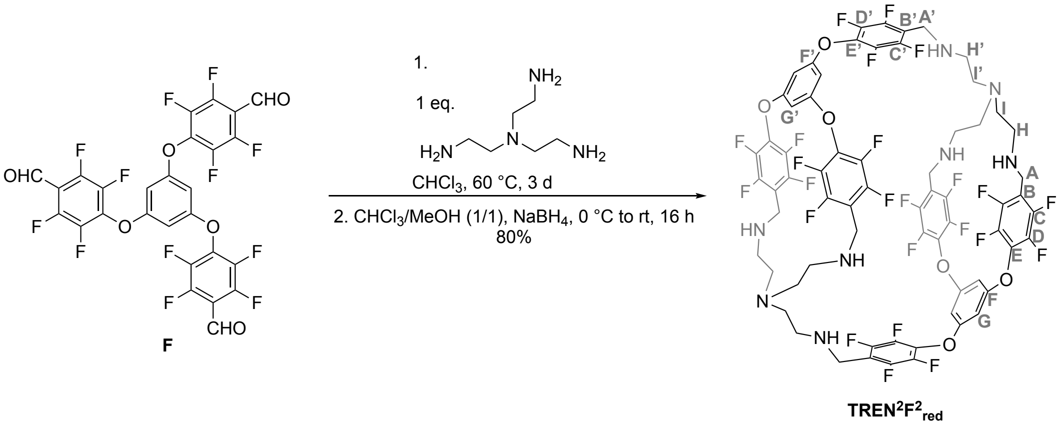

Aldehyde **F** (131 mg, 200 µmol, 1.00 eq.) was dissolved in 40 mL of chloroform to which **TREN** (30 µL, 200 µmol, 1.00 eq.) was added. The solution was heated to 60 °C and stirred for three days. Upon completion, indicated by ^{19}F NMR, 40 mL methanol were added, and the mixture was cooled to 0 ºC using an ice bath. Afterwards, $NaBH_4$ (756 g, 20.0 mmol, 100 eq.) was added in small portions over the course of an hour. After complete addition, the mixture was allowed to slowly warm up to room temperature and left to react overnight. The solvent was removed *in vacuo*, the resulting white solid was redissolved with 100 mL of 2 M hydrochloric acid and stirred for 30 minutes at room temperature. The solution was transferred into a separatory funnel, and the aqueous phase was washed with 2 x 40 mL of chloroform and discarded. Afterwards, the aqueous phase was brought to a pH of 7–8 by the addition of a concentrated aq. KOH solution (8M). The resulting colourless suspension was extracted with 3 x 100 mL of chloroform. The collected organic phases were dried over Na_2SO_4, and solvent was removed *in vacuo* to yield the amine cage **TREN^{2}F$^2_{red}$** as a colourless powder (120 mg, 79.7 µmol, 80%) and was used as obtained.

^{1}H NMR (600 MHz, $CDCl_3$): δ = 6.53 (s, 2H, **H$_G$**), 5.61 (s, 1H, **H$_{G'}$**), 3.97 (s, 2H, **H$_{A'}$**), 3.81 (s, 4H, **H$_A$**), 2.87 – 2.84 (m, 2H, **H$_{H'}$**), 2.72 (t, *J* = 5.3 Hz, 4H, **H$_H$**), 2.68 – 2.64 (m, 2H, **H$_{I'}$**), 2.63 – 2.59 (m, 4H, **H$_I$**), observed integrals are larger due to underlying oligomeric byproducts; **^{19}F NMR** (282 MHz, $CDCl_3$): δ = -144.89 – -145.44 (m, 3F, **F$_C$** and **F$_{C'}$** are partially overlaying), -153.48 (d, *J* = 13.2 Hz, 2F, **F$_D$**), -153.71 (dd, *J* = 21.9, 9.2 Hz, 1F, **F$_{D'}$**); **^{13}C{^{1}H} NMR** (151 MHz, $CDCl_3$): δ = 158.83 (**C$_{F'}$**), 158.21 (**C$_F$**), 141.37 (dd, *J* = 250.8, 13.7 Hz, **C$_{C,C'}$**), 140.71 (dd, *J* = 253.6, 15.3 Hz, **C$_D$**), 131.45 – 131.14 (m, **C$_{D'}$**), 130.52 – 130.23 (m, **C$_E$**), 115.48 (t, *J* = 18.6 Hz, **C$_{B'}$**), 114.86 (t, *J* = 18.6 Hz, **C$_B$**), 99.72 (**C$_G$**), 93.53 (**C$_{G'}$**), 54.91 (**C$_I$**), 54.65 (**C$_{I'}$**), 48.08 (**C$_A$**), 48.04 (**C$_{A'}$**) 41.14 (**C$_H$**), 41.05 (**C$_{H'}$**); **IR** (cm^{-1}): 2829.85 (w), 1606.70 (m), 1490.97 (s), 1460.11 8m), 1336.67 (w), 1294.24 (w), 1217.08 (w), 1145.72 (s), 1004.91 (m), 935.48 (m), 835.18 (w), 744.52 (w), 669.30 (w); **HRMS (ESI)**: m/z calculated for $[C_{66}H_{48}F_{24}N_6O_6+H]^+$= 1505.3386, found m/z = 1505.3376.

Synthesis of the model compound (MC)

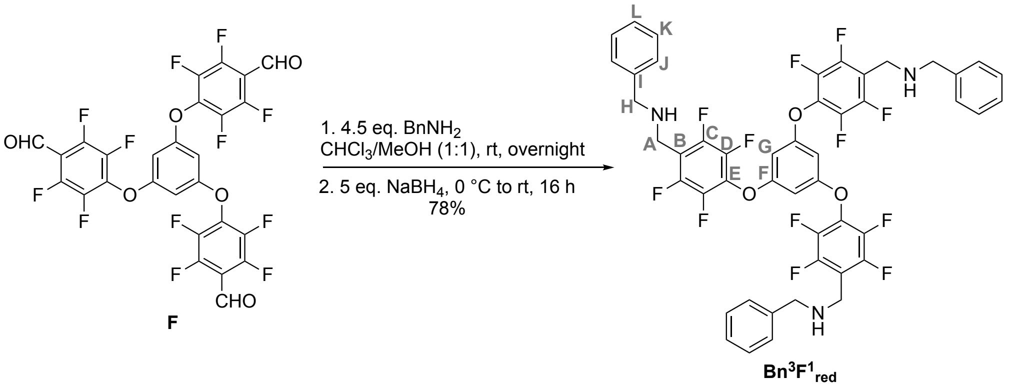

Aldehyde **F** (261 mg, 0.40 mmol, 1.0 eq.) was dissolved in 50 mL of chloroform/ methanol (1:1 v/v), to which a solution of benzylamine (0.21 mL, 1.8 mmol, 4.5 eq.) in 25 mL of chloroform/ methanol (1:1 v/v) was added dropwise over the course of 1 h. The solution was left to react for 24 h at room temperature with heavy stirring monitored by ^{19}F NMR. After the completion of the reaction, the mixture was cooled to 0 ºC using an ice bath, and $NaBH_4$ (190 mg, 5.0 mmol, 5.0 eq.) was added in small portions over the course of an hour. After the addition, the mixture was allowed to slowly warm up to room temperature and left to react overnight. After that time, deionised water was added (200 mL), the organic phase was separated, and the aqueous phase was extracted with dichloromethane (3 x 25 mL). The organic phase was collected and washed with brine (3 x 50 mL), dried over Na_2SO_4, and the solvent was removed *in vacuo*, affording the model compound **$Bn^3F^1_{red}$** as a yellow oil (290 mg, 0.31 mmol, 78%) and was used as obtained.

^{1}H NMR (600 MHz, $CDCl_3$): δ = 7.27 – 7.24 (m, 5H, **$H_{J,K,L}$**), 6.25 (s, 1H, **$H_{J,K,L}$**), 3.89 (s, 2H, **H_A**), 3.75 (s, 2H, **H_H**); **^{19}F NMR** (565 MHz, $CDCl_3$) δ -136.30 – -143.90 (m, 2F, **F_C**), -150.24 (dd, *J* = 21.4, 8.3 Hz, 2F, **F_D**); **^{13}C{^{1}H} NMR** (151 MHz, $CDCl_3$) δ 158.87 (**C_F**), 145.53 (d, *J* = 246.6 Hz, **C_C**), 141.07 (dd, *J* = 252.7, 15.5 Hz, **C_D**), 138.92 (d, *J* = 2.4 Hz, **C_C**), 131.67 (t, *J* = 12.5 Hz, **C_E**), 128.48 (**C_K**), 128.03 (**C_J**), 127.36 (**C_L**), 115.16 (t, *J* = 18.5 Hz, **C_B**), 98.79 (**C_G**), 52.93 (**C_H**), 39.98 (**C_A**); **IR** (cm^{-1}):1606.70 (m), 1490.97 (s), 1460.11 (m), 1384.89 (w), 1363.67 (w), 1338.60 (w), 1294.24 (w), 1199.72 (w), 1143.79 (s), 1089.78 (w), 1058.92 (w), 1002.98 (m), 983.70 (m), 925.83 (m), 831.32 (w), 734.88 (m), 696.30 (m); **HRMS (ESI)**: m/z calculated for $[C_{48}H_{33}F_{12}N_3O_3+H]^+$ = 928.2403, found m/z = 928.2396.

III.c. Synthesis of the non-fluorinated cages

Synthesis of Et^2H^2

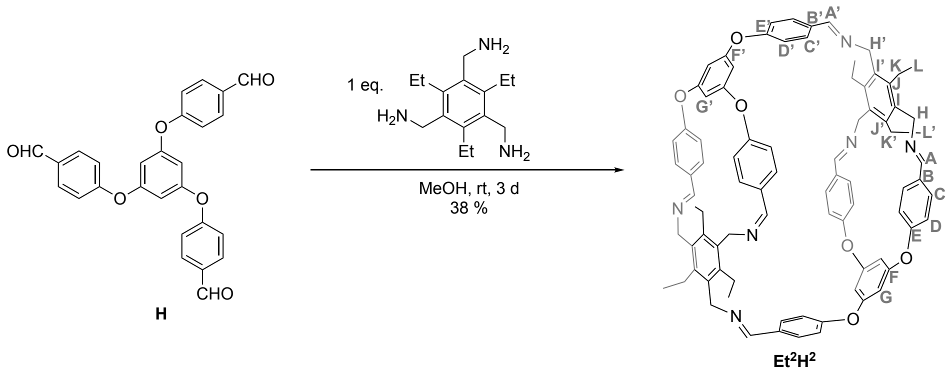

Aldehyde **H** (87.9 mg, 200 µmol, 1.00 eq.) was suspended in 20 mL of methanol, and a solution of **Et** (30.0 mg, 200 mmol, 1.00 eq.) in 30 mL methanol was added dropwise over the course of 2 h. During the addition, the suspension turned into a pale-yellow solution and then back to a suspension. The mixture was stirred for 3 days at room temperature. Afterwards, the precipitate was filtered off, washed with 5 mL acetonitrile, and the resulting solid was extracted with 100 mL dichloromethane using sonication. Filtration, followed by the addition of 20 mL *n*-hexane and the removal of the solvent, yielded the pure imine cage **Et^2H^2** as a colourless powder (40 mg, 38 µmol, 38%).

Note: If necessary, residual aldehyde can be removed by washing the obtained solid with acetonitrile one additional time as the cage is insoluble in acetonitrile while **H** shows a high solubility.

^{1}H NMR (600 MHz, $CDCl_3$): δ = 8.03 (s, 1H, **$H_{A'}$**), 7.73 (d, *J* = 8.0 Hz, 2H, **$H_{C'}$**), 7.67 (s, 2H, **H_A**), 7.43 (d, *J* = 7.8 Hz, 4H, **H_C**), 7.19 (d, *J* = 8.0 Hz, 2H, **$H_{D'}$**), 6.91 (d, *J* = 7.9 Hz, 4H, **H_D**), 6.47 (s, 2H, **H_G**), 5.24 (s, 1H, **$H_{G'}$**), 5.08 (s, 2H, **$H_{H'}$**), 4.97 (d, *J* = 15.9 Hz, 2H, **H_H**), 4.86 (d, *J* = 15.8 Hz, 2H, **H_H**), 2.72 (dq, *J* = 13.9, 6.3 Hz, 2H, **H_K**), 2.61 (dq, *J* = 14.6, 7.3 Hz, 2H, **H_K**), 2.29 (q, *J* = 6.8 Hz, 2H, **$H_{K'}$**), 1.27 (t, *J* = 7.2 Hz, 6H, **H_L**), 1.20 (t, *J* = 7.2 Hz, 3H, **$H_{L'}$**); **^{13}C{^{1}H} NMR** (151 MHz, $CDCl_3$): δ = 160.56 (**C_F**), 159.86 (**$C_{F'}$**), 159.43 (**C_A**), 158.51 (**$C_{A'}$**), 158.24 (**$C_{E'}$**), 157.14 (**C_E**), 143.78 (**$C_{J'}$**), 143.66 (**C_J**), 133.88 (**C_I**), 133.15 (**C_B**), 132.74 (**$C_{B'}$**), 130.82 (**$C_{I'}$**), 129.82 (**$C_{C'}$**), 129.49 (**C_C**), 121.38 (**C_D**), 120.15 (**$C_{D'}$**), 102.10 (**C_G**), 99.10 (**$C_{G'}$**), 55.86 (**$C_{H'}$**), 55.32 (**C_H**), 23.68 (**C_K**), 22.88 (**$C_{K'}$**), 16.36 (**C_L**); **IR** (cm^{-1}): 2962.66 (w), 2927.94 (w), 2900.94 (w), 2870.08 (w), 1699.29 (w), 1637.56 (w), 1591.27 (s), 1502.55 (s), 1454.33 (m), 1417.68 (w), 1375.25 (w), 1307.74 (w), 1298.09 (w), 1222.87 (s), 1161.15 (m), 1114.86 (m), 1074.35 (w), 1043.49 (w), 1004.91 (m), 966.34 (w), 867.97 (m), 833.25 (m), 765.74 (w); **HRMS (ESI)**: m/z calculated for $[C_{84}H_{78}N_6O_6+H]^+$ = 1267.6057, found m/z = 1267.6049.

Synthesis of Et⁴H⁴

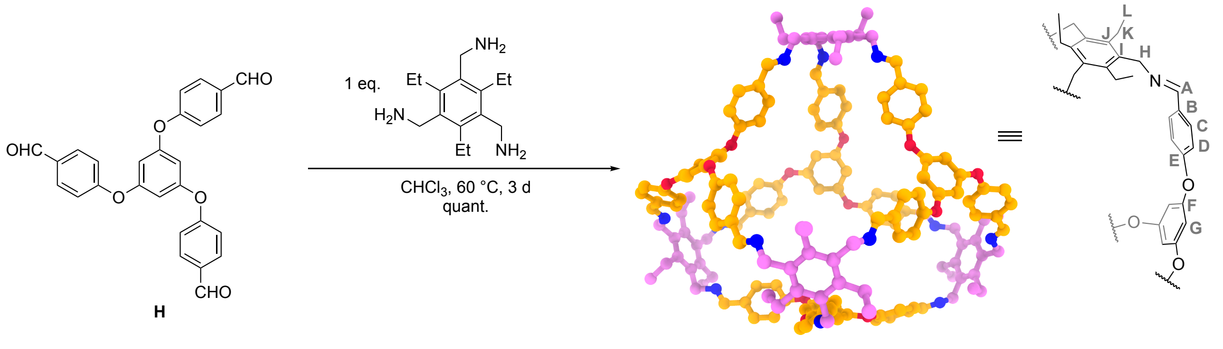

Aldehyde **H** (23.0 mg, 52.5 μmol, 1.00 eq.) was dissolved in 10 mL chloroform, and amine **Et** (13.2 mg, 52.9 mmol, 1.01 eq.) was added. The reaction flask was capped, and the mixture was stirred at 60 °C for 3 days. Afterwards, the clear solution was filtered, the flask and filter paper were rinsed with 5 mL chloroform, and 20 mL *n*-hexane were added to the resulting filtrate. Removal of the solvent by rotary evaporation yielded the pure imine cage **Et²H²** as a colourless powder (34 mg, 26 μmol, quant.).

Note: If necessary, an excess of one or both starting materials can be removed by washing the obtained solid with acetonitrile.

¹H NMR (600 MHz, $CDCl_3$): δ = 7.94 (s, 1H, **H_A**), 7.65 (d, *J* = 8.2 Hz, 2H, **H_C**), 7.06 (d, *J* = 8.2 Hz, 2H, **H_D**), 6.19 (s, 1H, **H_G**), 5.00 (s, 2H, **H_H**), 2.54 (q, J = 6.4 Hz, 2H, **H_K**), 1.25 (t, *J* = 7.3 Hz, 3H, **H_L**); **¹³C{¹H} NMR** (151 MHz, $CDCl_3$): δ = 160.02 (**C_F**), 158.50 (**C_A**), 157.30 (**C_E**), 144.02 (**C_J**), 133.11 (**C_I**), 132.04 (**C_B**), 129.88 (**C_C**), 120.71 (**C_D**), 101.03 (**C_G**), 55.86 (**C_H**), 23.32 (**C_K**), 16.07 (**C_L**); **IR** (cm^{-1}): 2358.94 (w), 2330.01 (w), 1641.42 (w), 1593.20 (m), 1502.55 (m), 1456.26 (m), 1315.45 (w), 1224.80 (s), 1159.22 (m), 1118.71 (m), 1004.91 (m), 966.34 (w), 873.75 (w), 833.25 (m); **MS (MALDI)**: m/z calculated for $[C_{168}H_{156}N_{12}O_{12}+H]^+$ = 2534.20, found m/z = 2534.24.

Synthesis of TREN^{4}H^4

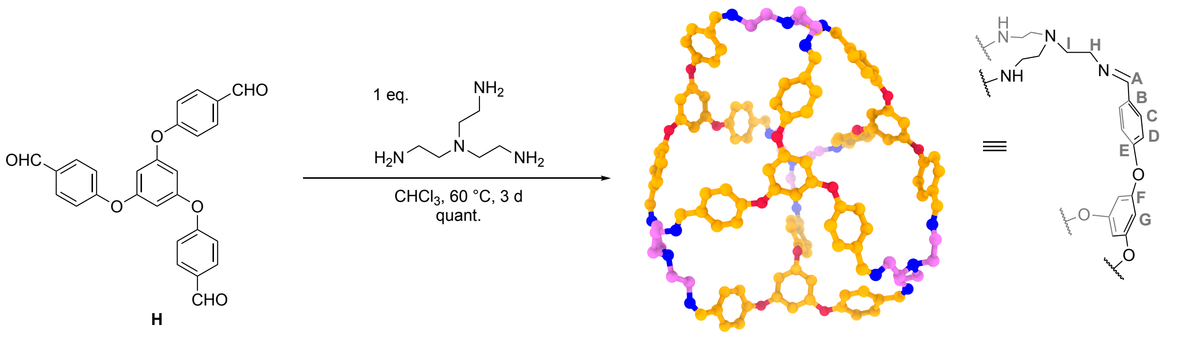

TREN^{4}H^4

Aldehyde **H** (22 mg, 50 µmol, 1.0 eq.) was dissolved in 10 mL chloroform, and amine **TREN** (7.5 µL, 50 mmol, 1.0 eq.) was added. The reaction flask was capped, and the mixture was stirred at 60 °C for 3 days. Afterwards, the clear solution was filtered, and the flask and filter paper were rinsed with 5 mL chloroform. and 20 mL *n*-hexane were added to the resulting filtrate. Removal of the solvent by rotary evaporation yielded the pure imine cage **TREN^{4}H^4** as a colourless powder (27 mg, 25 µmol, quant.).

Note: If necessary, an excess of amine can be removed by washing the obtained solid with methanol.

^{1}H NMR (600 MHz, $CDCl_3$): δ = 7.71 (s, 1H, **H_A**), 7.24 – 7.09 (m, 4H, **$H_{C,D}$**), 6.19 (s, 1H, **H_G**), 3.81 (s, 1H, **H_H**), 3.28 (bs, 1H, **H_H**), 2.94 (bs, 1H, **H_I**), 2.68 (bs, 1H, **H_I**); **^{13}C{^{1}H} NMR** (151 MHz, $CDCl_3$): δ = 161.66 (**C_F**), 160.49 (**C_A**), 156.09 (**C_E**), 134.13 (**C_C**), 130.12 (**C_B**), 122.70 (**C_D**), 97.31 (**C_G**), 60.11 (**C_I**), 56.28 (**C_H**); **IR** (cm^{-1}): 1645.28 (w), 1591.27 (m), 1502.55 (m), 1454.33 (m), 1415.75 (w), 1377.17 (w), 1336.67 (w), 1296.16 (w), 1222.87 (s), 1159.22 (m), 1118.71 (m), 1064.71 (w), 1033.85 (w), 1004.91 (m), 920.05 (w), 831.32 (m), 815.89 (m), 756.10 (m); **HRMS (ESI)**: m/z calculated for $[C_{132}H_{120}N_{16}O_{12}+2H]^{2+}$ = 1061.4709, found m/z = 1061.4697.

IV. Cage formation studies

IV.a. Cage formation studies under thermodynamic and kinetic control

General procedure A, thermodynamic control, using $CHCl_3$ at 60 °C:

Aldehyde **F** or **H** (50 µmol, 1.0 eq.) was dissolved in 10 mL chloroform, and the respective amine (50 µmol, 1.0 eq.) was added. The reaction flask was capped, and the mixture was stirred at 60 °C for 3 days. Afterwards, the clear solution was filtered, the flask and filter paper were rinsed with 5 mL chloroform, and a sample of 0.1 mL of the filtrate was submitted to MALDI-MS analysis. 20 mL *n*-hexane were added to the resulting filtrate. The resulting clear solution was then concentrated to roughly 10 mL (rotary evaporation, bath temp. 50 °C), giving a pale-yellow precipitate. In the case of fluorinated cages, 20 mL of *n*-hexane were again added to the suspension, which was filtered, resulting in a completely dissolvable pale-yellow powder. This was done to prevent the formation of insoluble polymers, as it was observed that especially **TREN^{2}F^2** was likely to form insoluble polymers during the solvent removal. For the non-fluorinated cages, the solvent was completely removed, yielding a colourless, completely dissolvable solid. The obtained solids were investigated by NMR and MALDI-MS analysis. All analysis was performed in less than 4 h after dissolving/ isolating the samples.

General procedure B, kinetic control, using MeCN or MeOH at room temperature:

Aldehyde **F** or **H** (50 µmol, 1.0 eq.) was suspended in 4 mL of solvent, and a solution of the respective amine (50 µmol, 1.0 eq.) in 6 mL solvent was added dropwise over the course of 30 min. The mixture was stirred for 3 days at room temperature. Afterwards, the precipitate was filtered off, washed with 2 mL of the respective solvent, and a 0.1 mL sample of the resulting filtrate was taken for MALDI-MS analysis. The solvent was removed by rotary evaporation (bath temp. 50 °C), and the crude reaction mixture was redissolved with $CDCl_3$ for NMR analysis. The obtained precipitates were only partly dissolvable in many cases, thus, roughly 12 mg of the obtained precipitates were sonicated in 0.9 mL $CDCl_3$ for 3 minutes and filtered through a syringe filter. The obtained clear solutions were used for NMR and MALDI-MS analysis. All analysis was performed in less than 4 h after dissolving/ isolating the samples.

The results are listed in Table S1, and the respective NMR spectra are shown in Fig. S1-S8.

Table S1: Summary of the results for the reactions between aldehydes **F** and **H** with the amines **Et** and **TREN**. For the observed mixtures, the relative compositions were calculated from the observed integrals in the ^{1}H NMR and are listed in the parentheses.

Aldehyde	Conditions		Amine	
			**Et**	**TREN**
**F**	MeOH (RT)	precip.	$\mathbf{Tri^2_2Tri^2}$	$\mathbf{Tri^2_2Tri^2}$ (74%)<br>$\mathbf{Tri^4Tri^4}$ (26%)
		filtrate	$\mathbf{Tri^2_2Tri^2}$	/
	MeCN (RT)	precip.	$\mathbf{Tri^2_2Tri^2}$	$\mathbf{Tri^4Tri^4}$
		filtrate	$\mathbf{Tri^2_2Tri^2}$ (91%)<br>$\mathbf{Tri^4Tri^4}$ (9%)	$\mathbf{Tri^2_2Tri^2}$ (94%)<br>$\mathbf{Tri^4Tri^4}$ (6%)
	$CHCl_3$ (60°C)	precip.	/	/
		filtrate	$\mathbf{Tri^2_2Tri^2}$ (76%)<br>$\mathbf{Tri^4Tri^4}$ (24%)	$\mathbf{Tri^2_2Tri^2}$ (96%)<br>$\mathbf{Tri^4Tri^4}$ (4%)
**H**	MeOH (RT)	precip.	$\mathbf{Tri^2_2Tri^2}$	$\mathbf{Tri^4Tri^4}$
		filtrate	Residual starting materials	Residual starting materials
	MeCN (RT)	precip.	Undefined oligomeric species	$\mathbf{Tri^4Tri^4}$
		filtrate	Undefined oligomeric species	Undefined oligomeric species
	$CHCl_3$ (60°C)	precip.	/	/
		filtrate	$\mathbf{Tri^4Tri^4}$	$\mathbf{Tri^4Tri^4}$

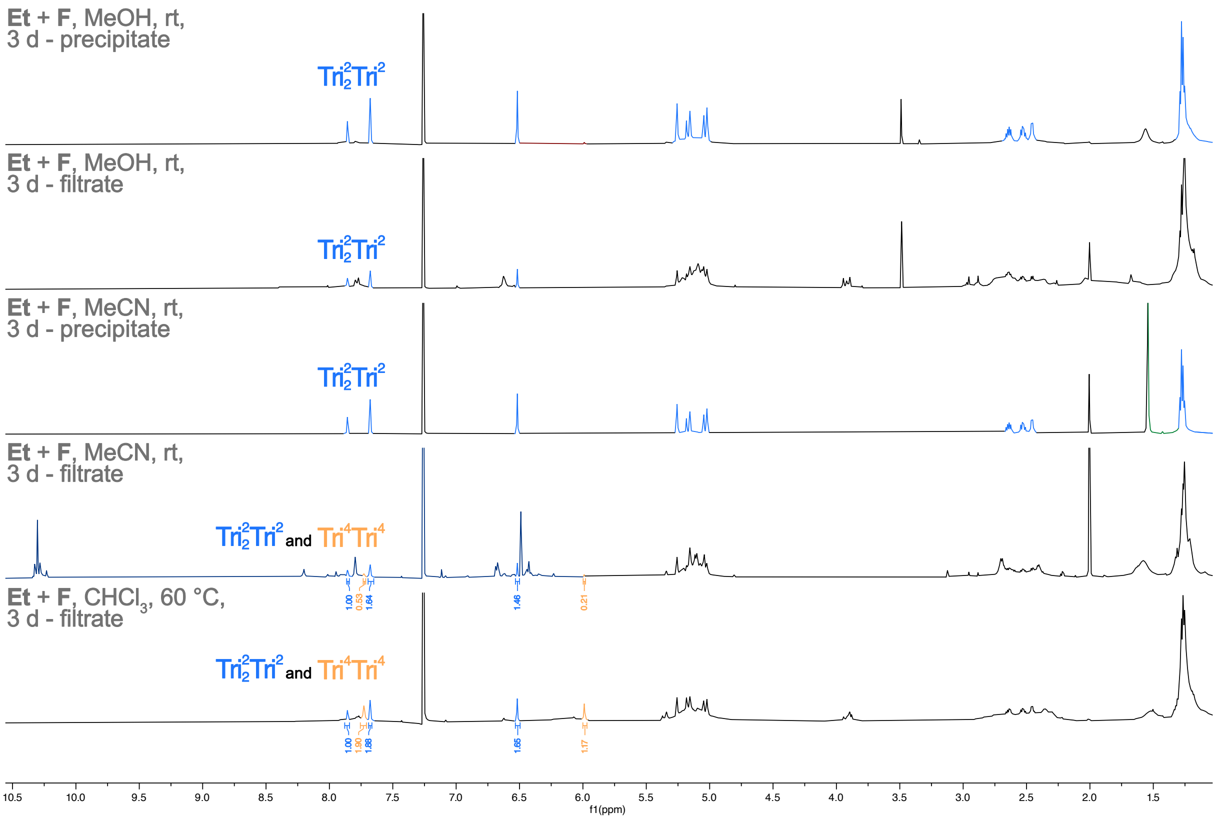

Figure S1: Stacked ^{1}H NMR (300 MHz, $CDCl_3$) spectra of the cage formation experiments between **Et** and **F**.

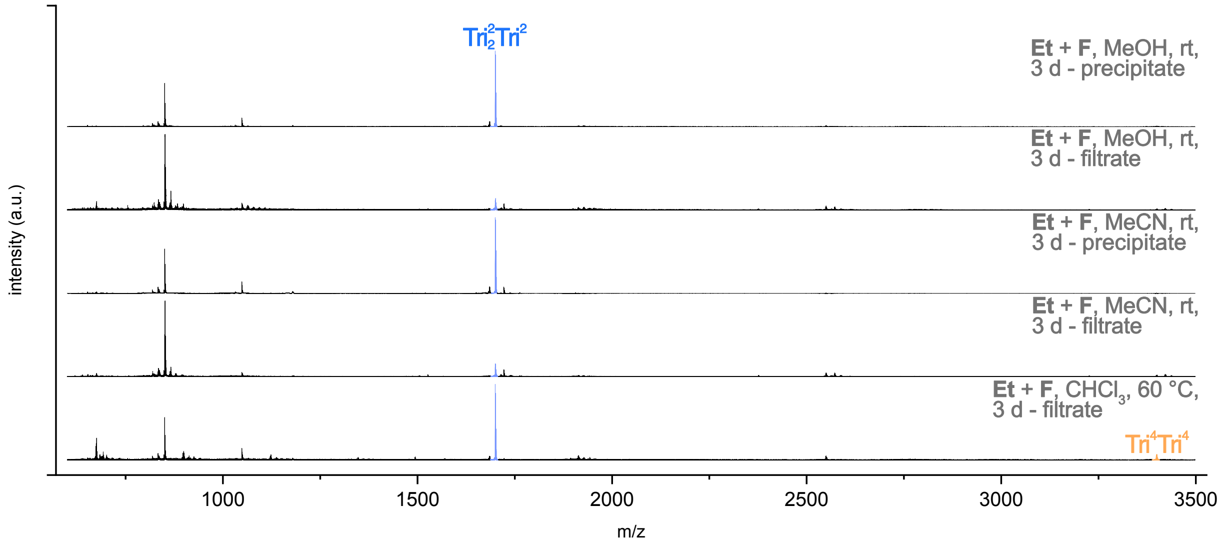

Figure S2: Stacked MALDI-MS spectra of the cage formation experiments between **Et** and **F**.

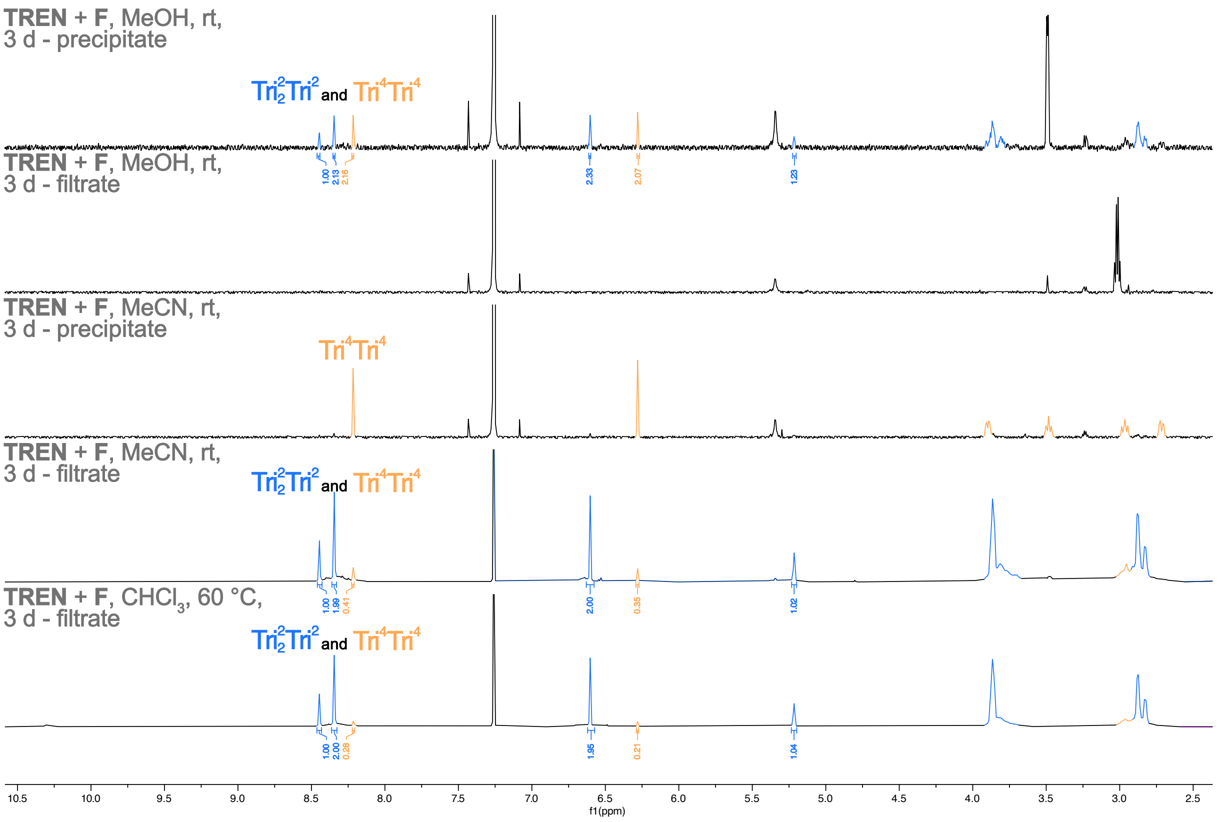

Figure S3: Stacked ^{1}H NMR (300 MHz, $CDCl_3$) spectra of the cage formation experiments between **TREN** and **F**.

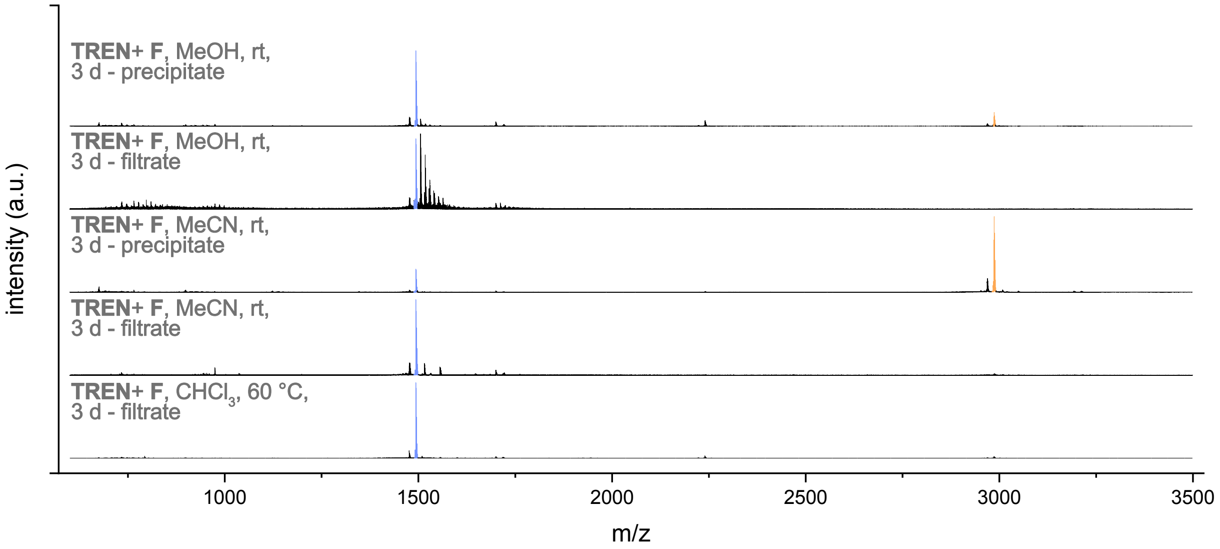

Figure S4: Stacked MALDI-MS spectra of the cage formation experiments between **TREN** and **F**.

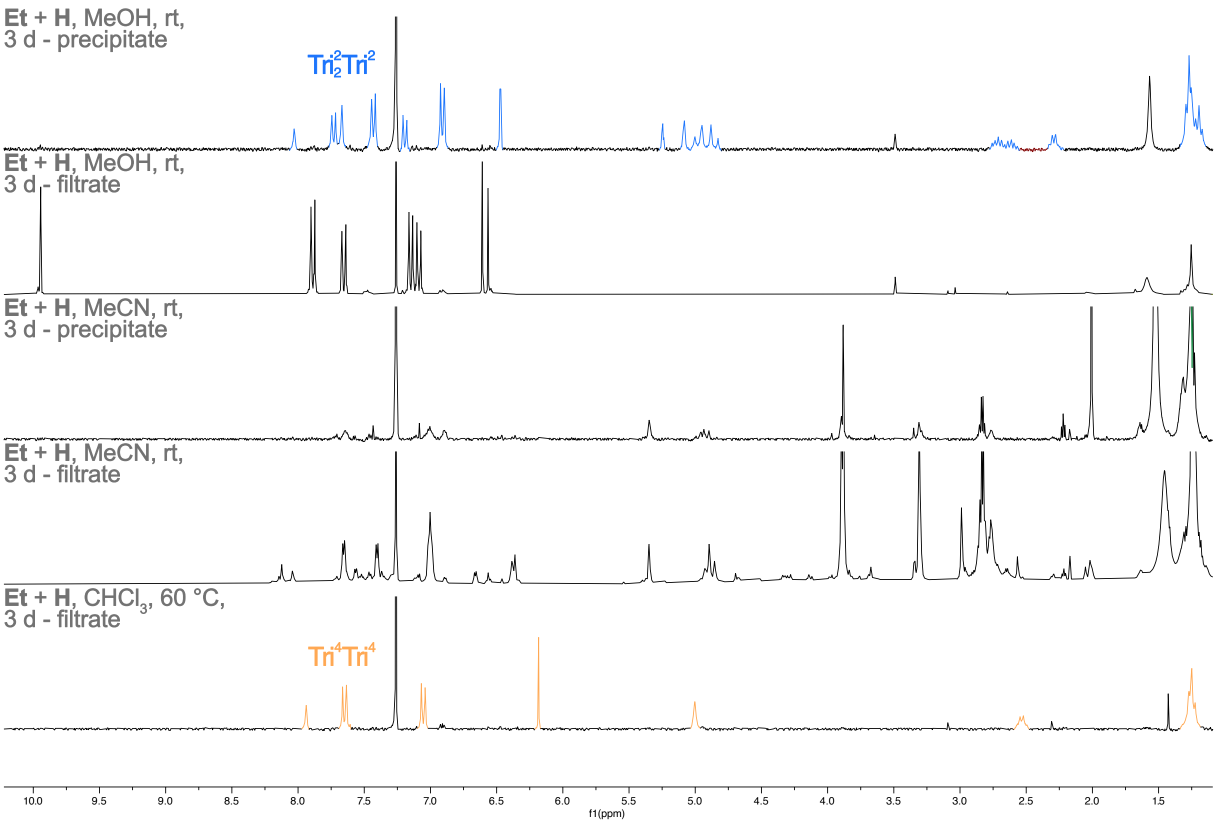

Figure S5: Stacked ^{1}H NMR (300 MHz, $CDCl_3$) spectra of the cage formation experiments between **Et** and **H**.

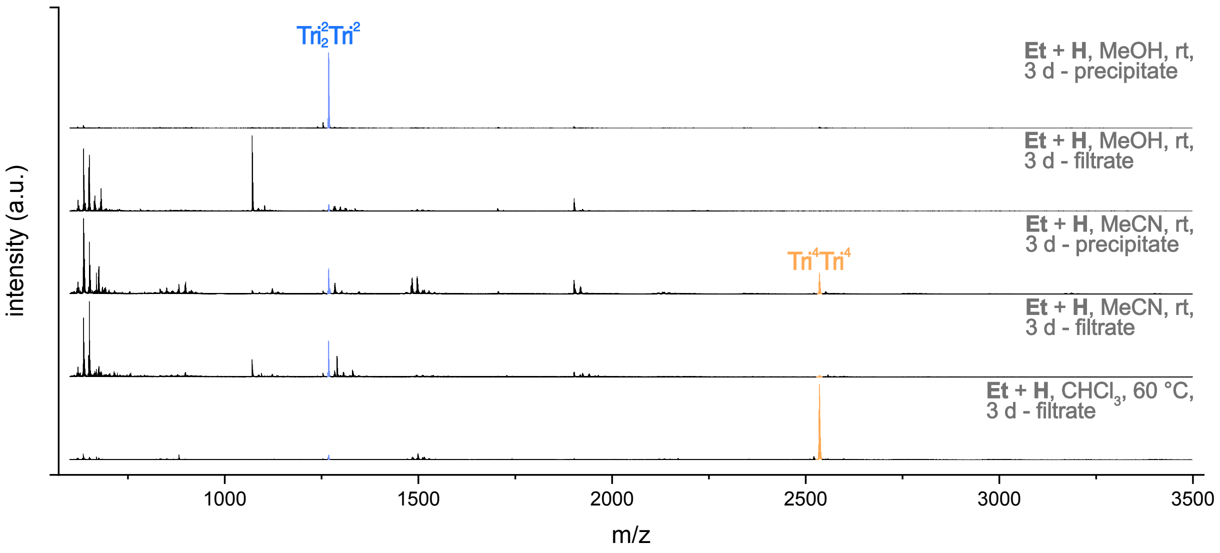

Figure S6: Stacked MALDI-MS spectra of the cage formation experiments between **Et** and **H**.

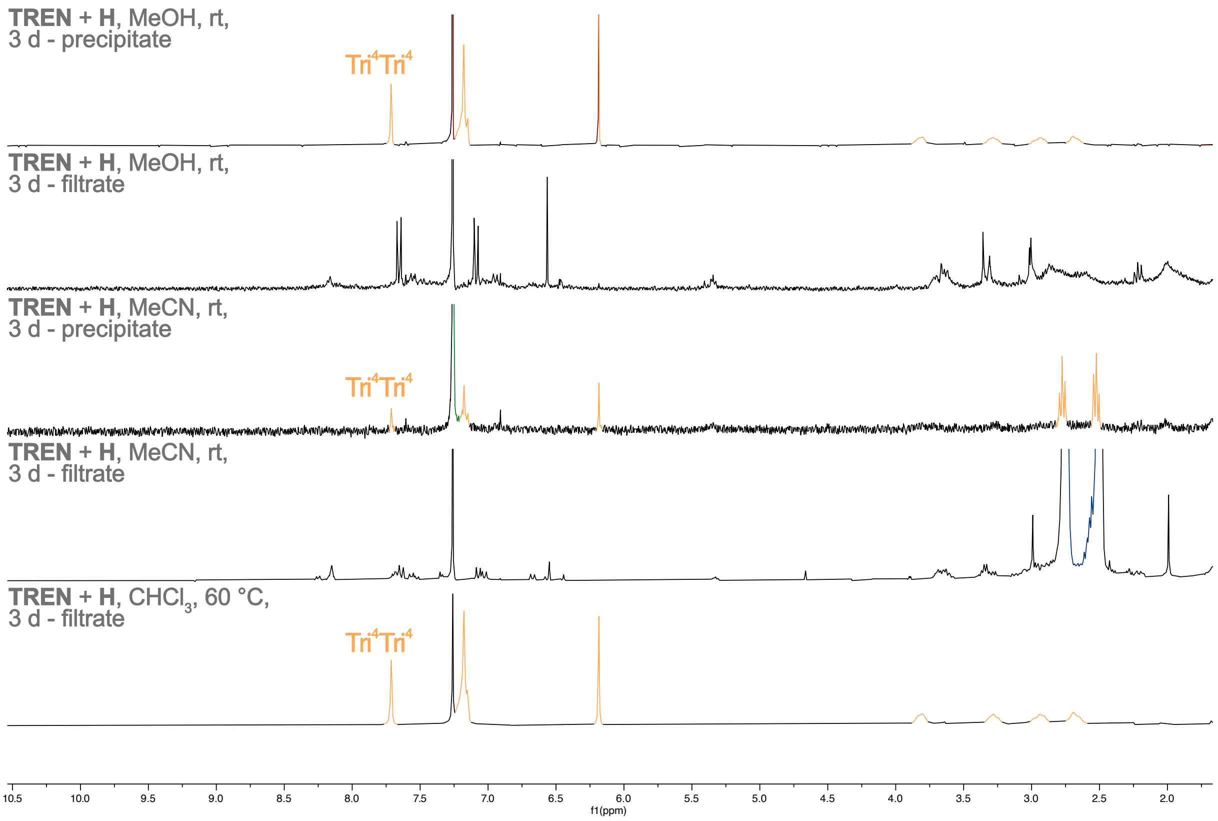

Figure S7: Stacked ^{1}H NMR (300 MHz, $CDCl_3$) spectra of the cage formation experiments between **TREN** and **H**.

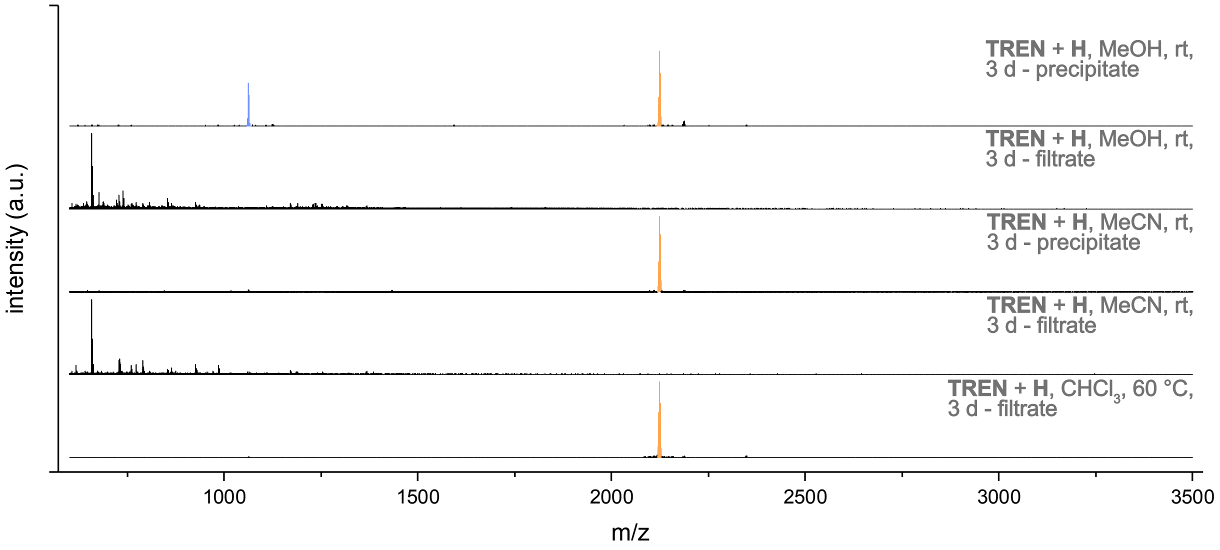

Figure S8: Stacked MALDI-MS spectra of the cage formation experiments between **TREN** and **H**.

IV.b. Extended cage formation studies of Et^XH^X

Aldehyde **H** (11 mg, 25 µmol, 1.0 eq.) was dissolved/ suspended in 5 mL of either chloroform or methanol, respectively. Afterwards, amine **Et** (6.2 mg, 25 µmol, 1.0 eq.) was added, and the mixture was either stirred at room temperature (chloroform) or 60 °C (methanol) for 3 days. The obtained mixtures were filtered, and the flask and filter paper were rinsed with 1 mL of the respective solvent. Afterwards, in the case of chloroform, 10 mL of *n*-hexane were added to the resulting filtrate. In all cases the solvent was removed by rotary evaporation (bath temp. 50 °C). The obtained solids were investigated by NMR and MALDI-MS analysis. All analysis was performed in less than 4 h after dissolving/ isolating the samples.

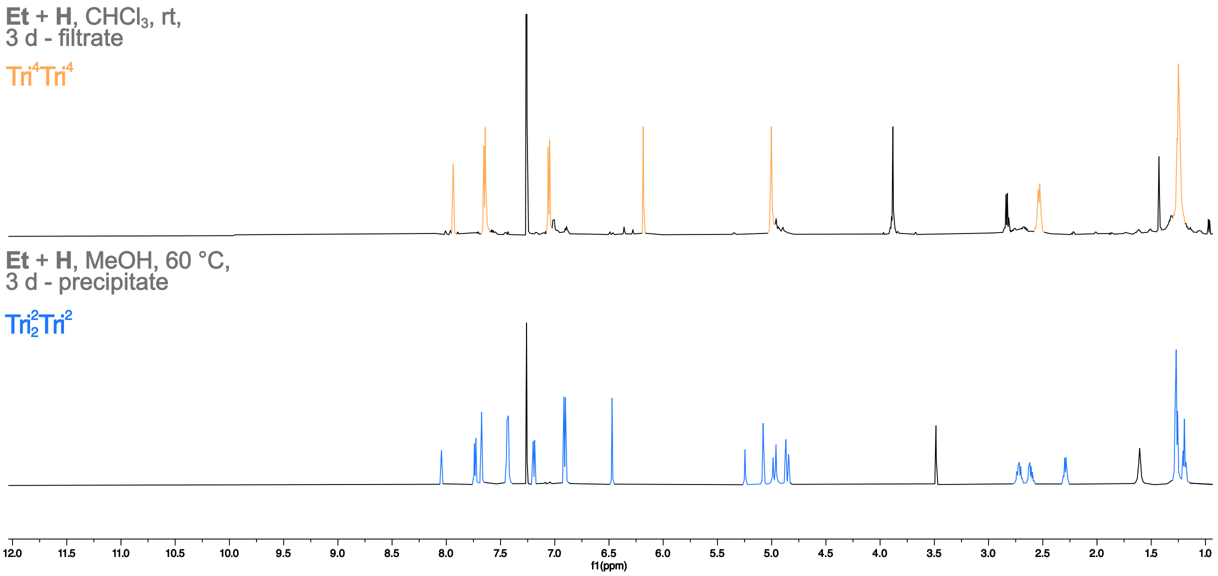

Figure S9: Stacked ^1H NMR (600 MHz, $CDCl_3$) spectra of the cage formation experiments between **Et** and **H.**

IV.c. Extended formation studies of Et^XH^X in solvent mixtures:

Aldehyde **H** (11 mg, 25 µmol, 1.0 eq.) was dissolved/ suspended in 2.5 mL of the respective solvent mixture. Afterwards, 2.5 mL (1.0 eq.) of a stock solution of amine **Et** in methanol (2.5 mg/mL) was added dropwise over the course of 30 min, and the mixture was stirred at room temperature for 3 days. The suspensions were filtered, and the obtained solid was left in the fume hood to dry for one hour. The solvent was removed from the filtrate by rotary evaporation (bath temp. 50 °C), and all obtained solids were investigated by NMR and MALDI-MS analysis. All analysis was performed in less than 4 h after dissolving/ isolating the samples.

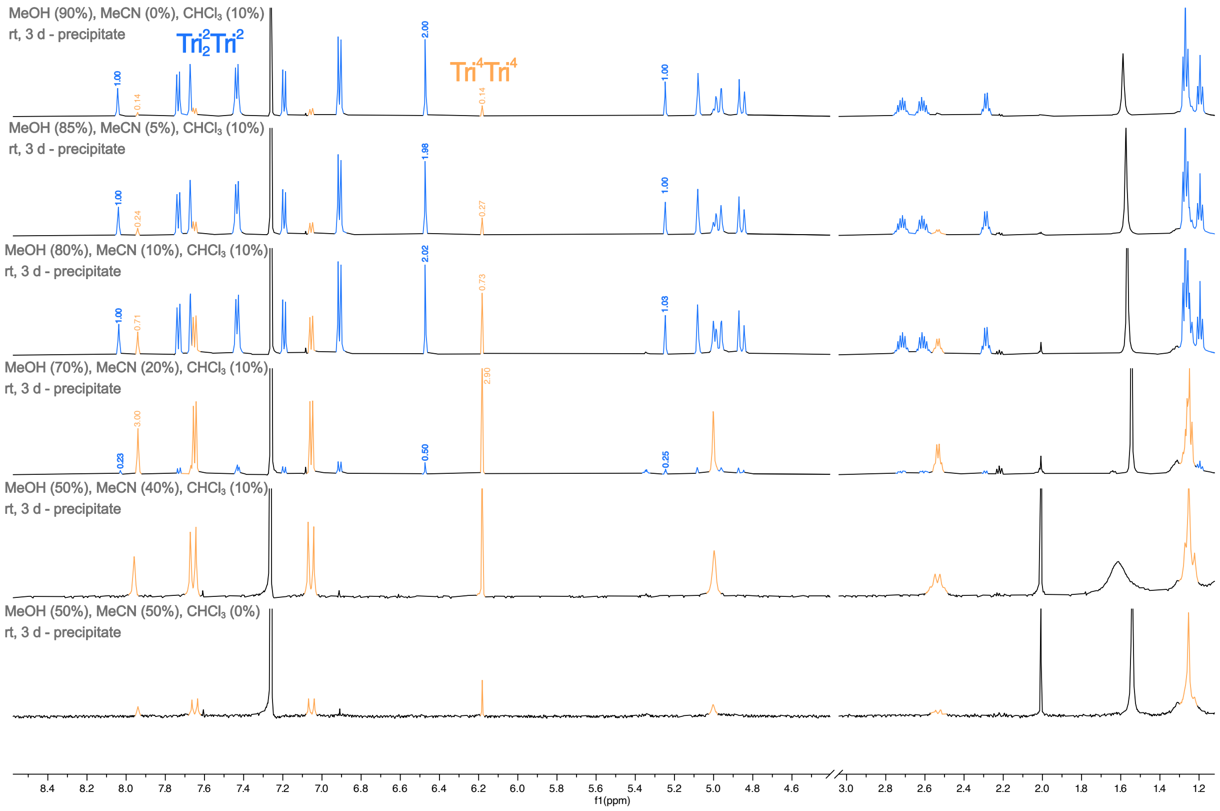

Figure S10: Stacked ^1H NMR (300 MHz, $CDCl_3$) spectra of the cage formation experiments between **Et** and **H** using different solvent mixtures, varying the amount of acetonitrile used, observed integrals are denoted above the respective signals.

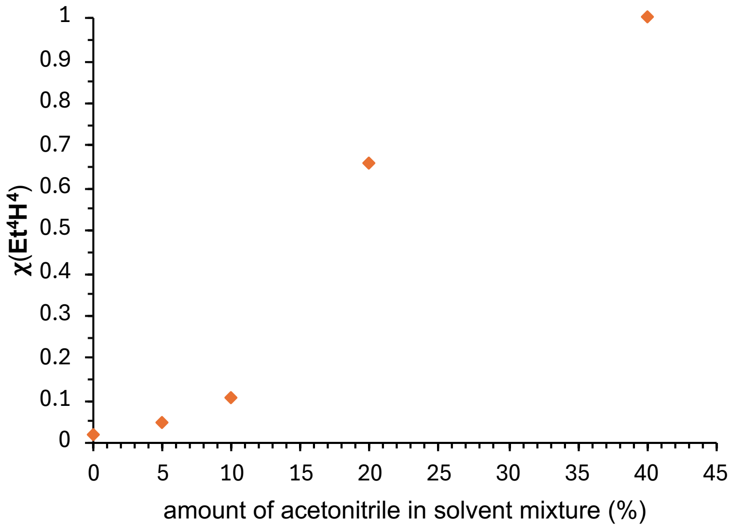

Figure S11: Plot of the molar fraction X of **Et⁴H⁴** in dependency of the amount of acetonitrile used, calculated from the H_G and $H_{G'}$ integrals observed in ^{1}H NMR.

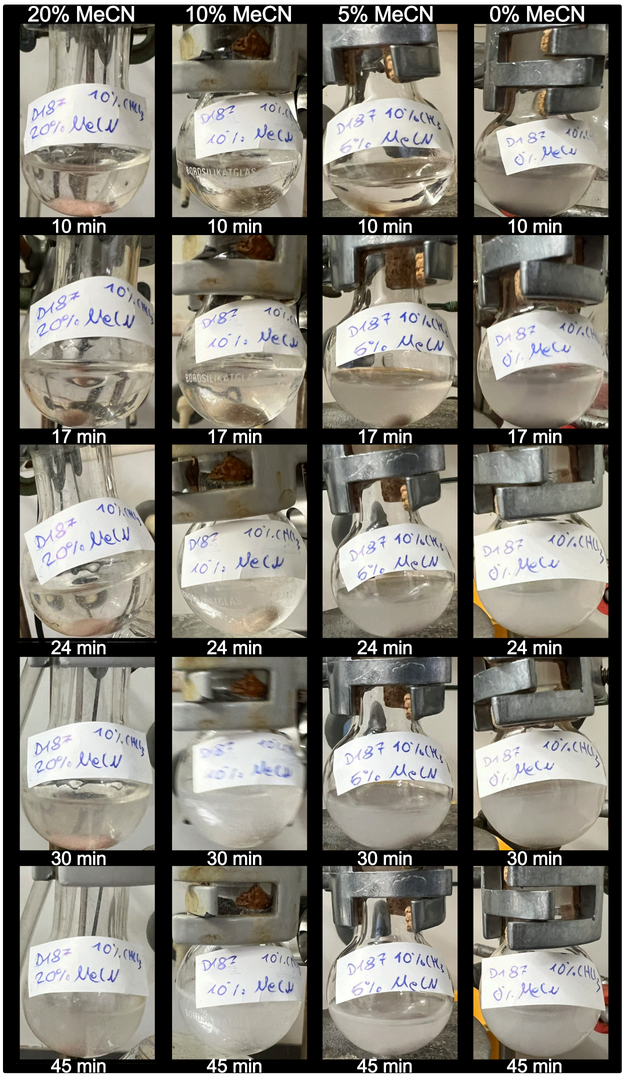

Figure S12: Photographs of the reaction flask during the mixed solvent screenings indicating, at which time precipitation occurred after the addition of the amine was completed. All reactions started from a clear solution.

V. Cage-to-Cage transformation of Et^2H^2 and Et^4H^4

Experiments performed while stirring:

3.0 mg of either cage $\mathbf{Et^2H^2}$ or $\mathbf{Et^4H^4}$ were dissolved/ suspended in the respective solvent (1 mL), and stirred for 3 days at either room temperature or 60°C in a capped vial. In the case of $\mathbf{Et^2H^2}$, $CDCl_3$ was used, and the obtained sample was directly submitted. Experiments conducted with $\mathbf{Et^4H^4}$ were performed in methanol. After 3 days, the colourless solids were filtered off and investigated by NMR and MALDI-MS. In chloroform, all species remained in solution without precipitation occurring, while in methanol, the only change observed was a colour change from a colourless to a pale-yellow suspension if heated to 60 °C for three days.

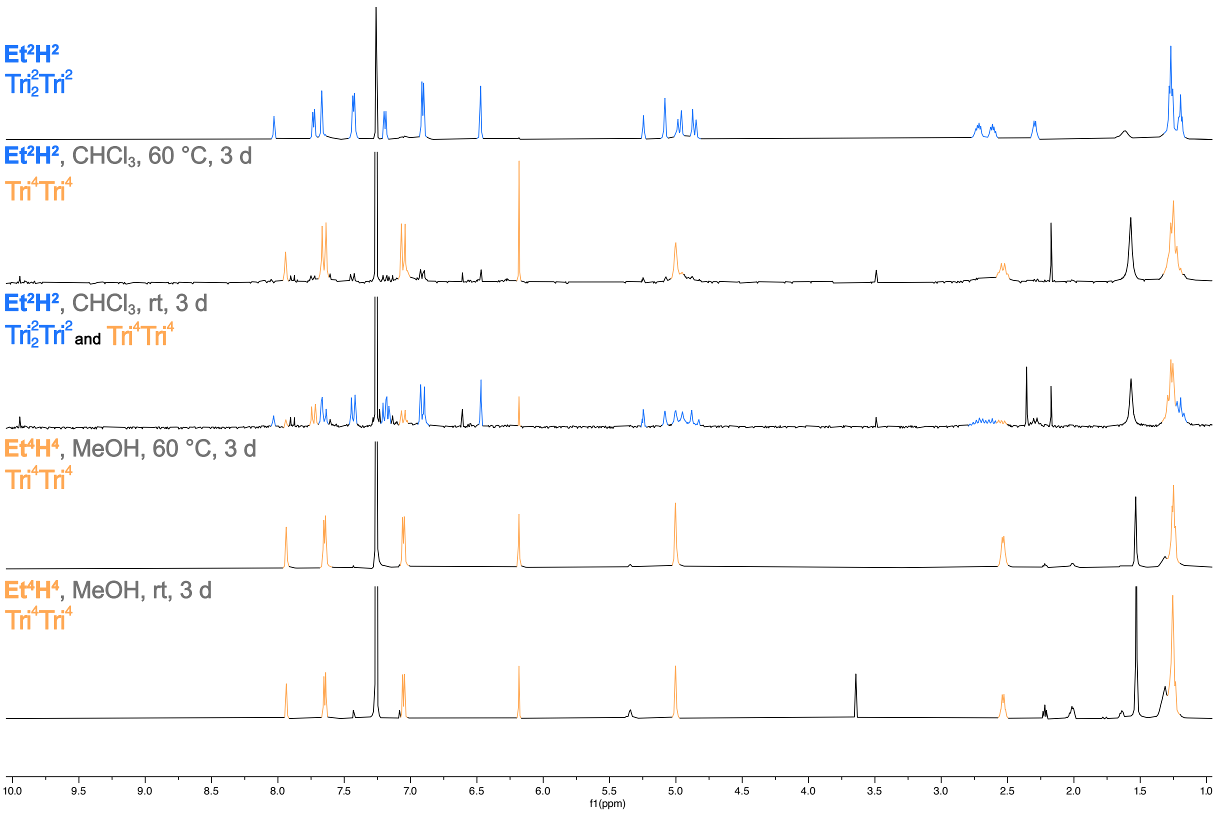

Figure S13: Stacked ^1H NMR (600 MHz, $CDCl_3$) spectra of the cage-to-cage transformation experiments between of $\mathbf{Et^2H^2}$ and $\mathbf{Et^4H^4}$.

NMR experiments:

6.0 mg of cage **Et²H²** were dissolved in 2 mL chloroform-d. The obtained solution was split, and 1 mL each was placed into two NMR tubes. The tubes were capped and closed with parafilm. Afterwards, one of the tubes was placed into an oil bath (60°C) while the other was placed in the fume hood at room temperature. Every 24 hours, the samples were remeasured, keeping them no longer than 2 hours out of the oil bath.

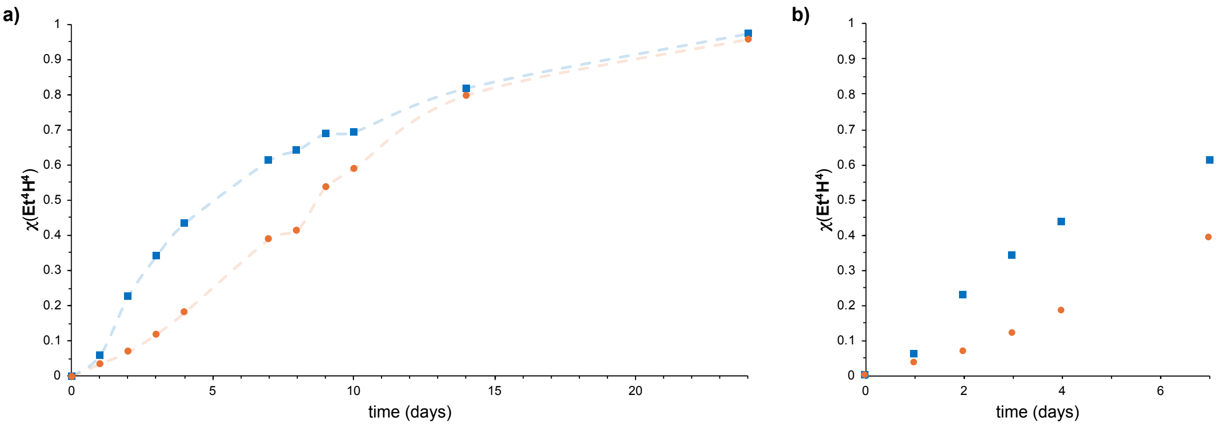

Figure S14: Plot of the cage-to-cage transformation experiments showing the molar fraction X of **Et⁴H⁴** against the time, calculated from the H_G and $H_{G'}$ integrals observed in ^{1}H NMR. a) Shows the complete timeframe until full conversion was reached and b) the initial timeframe of the first 7 days when **Et²H²** was left standing in $CDCl_3$ at either 60 °C (blue square) or room temperature (orange circle).

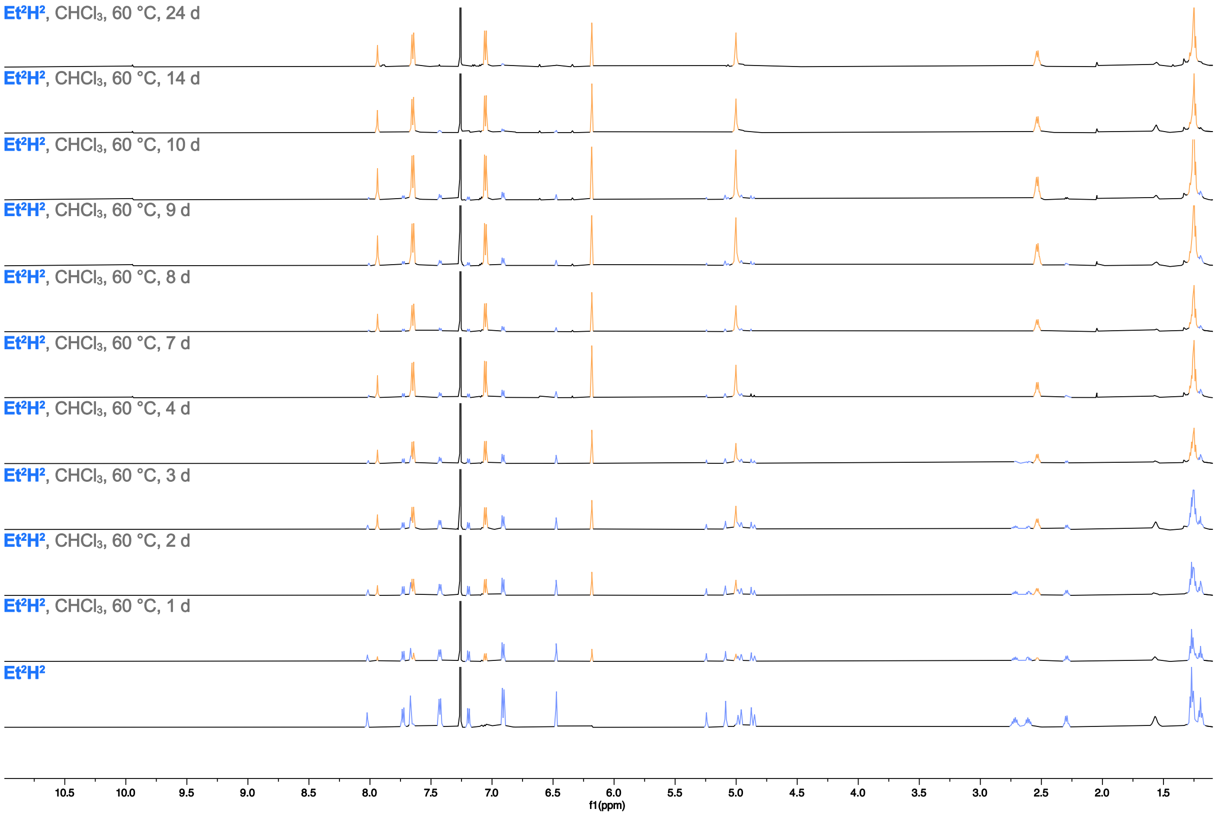

Figure S15: Stacked ^{1}H NMR (600 MHz, $CDCl_3$) spectra of the cage-to-cage transformation experiments from **Et^2H^2** to **Et^4H^4** performed without stirring in an NMR tube.

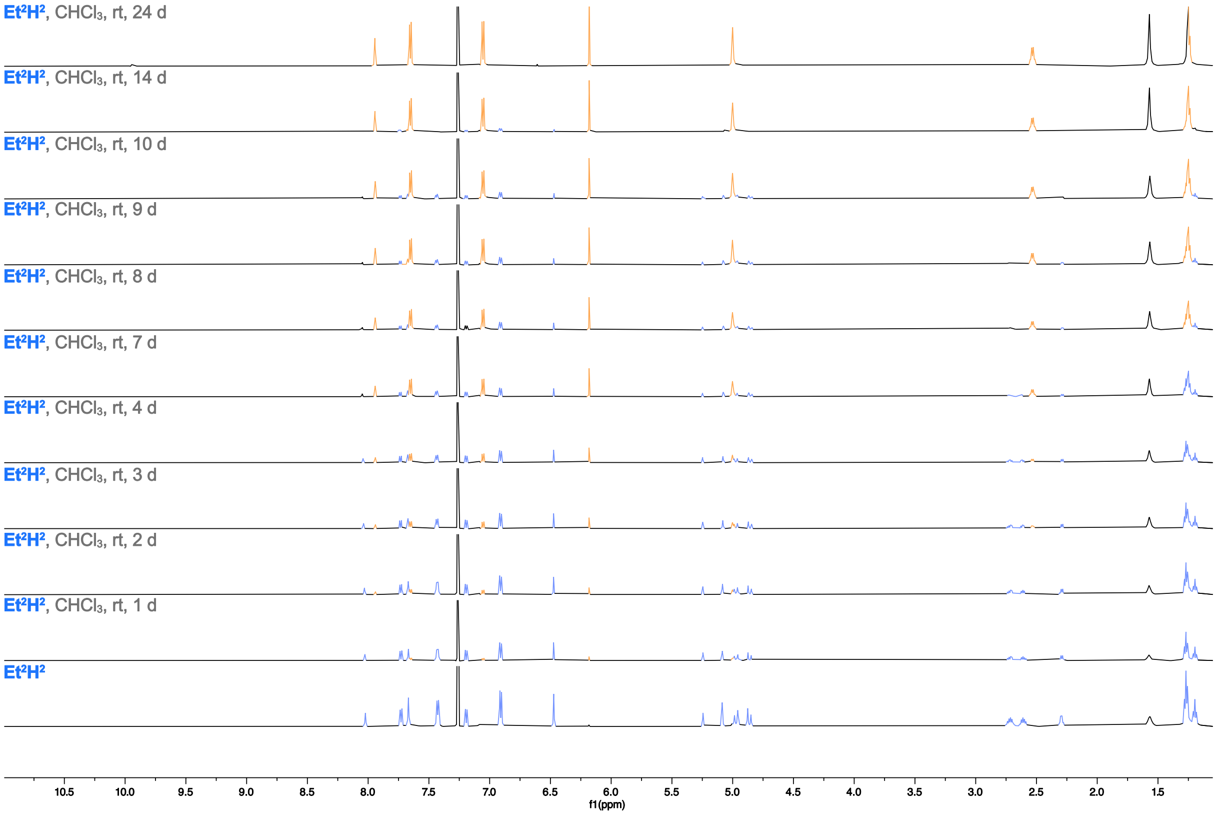

Figure S16: Stacked ^{1}H NMR (600 MHz, $CDCl_3$) spectra of the cage-to-cage transformation experiments from **Et^2H^2** to **Et^4H^4** performed without stirring in an NMR tube.

VI. SC-XRD

For all measurements carried out on a Rigaku XtaLAB Synergy Dualflex (**$Et^2F^2_{red}$** and **PFOA@$Et^2F^2_{red}$**), Cu-K_α (λ = 1.54178 Å) radiation was used for data collection at the temperature stated for each compound. Single-crystals were mounted using a microfabricated polymer film crystal-mounting tool (dual-thickness MicroMount, MiTeGen) using low-viscosity oil (perfluoropolyalkylether; viscosity 1800 cSt, ABCR) to reduce X-ray absorption and scattering. The structures were solved by intrinsic phasing (SHELXT-2013) and refined by full-matrix least-squares methods on F^2 (SHELXL-2014).[S3] The hydrogen atoms were placed in calculated positions and refined using a riding model. CCDC 2430764 (**$Et^2F^2_{red}$**) contains the supplementary crystallographic data for this paper. These data can be obtained free of charge from The Cambridge Crystallographic Data Centre.

$Et^2F^2_{red}$

Needle-like crystals of **$Et^2F^2_{red}$** were grown from a chloroform solution by slow evaporation at room temperature. A needle was mounted, and the structure was obtained at 170 K using Cu-K_α radiation. One chloroform molecule was successfully modelled, whereas another one showed heavy disorder and was ultimately removed using SQUEEZE.[S4]

PFOA@$Et^2F^2_{red}$

Crystals of **PFOA@$Et^2F^2_{red}$** were grown from a dichloromethane/acetonitrile solution by slow evaporation at room temperature in the presence of an excess of perfluorooctanoic acid. A colourless fragment was mounted, and the structure was obtained at 150 K using Cu-K_α radiation. A complete dataset was obtained, however, due to the large structure bearing no heavy atoms and the disorder of solvent molecules, as well as three independent perfluorooctanoic acid molecules (two modelled), a suitable model could be generated using restraints. The crystal data was ultimately not deemed good enough for deposition in the CCDC. Therefore, the structure is only used to discuss the general uptake of perfluorooctanoic acid within the cage, without discussing bond lengths and other structural features.

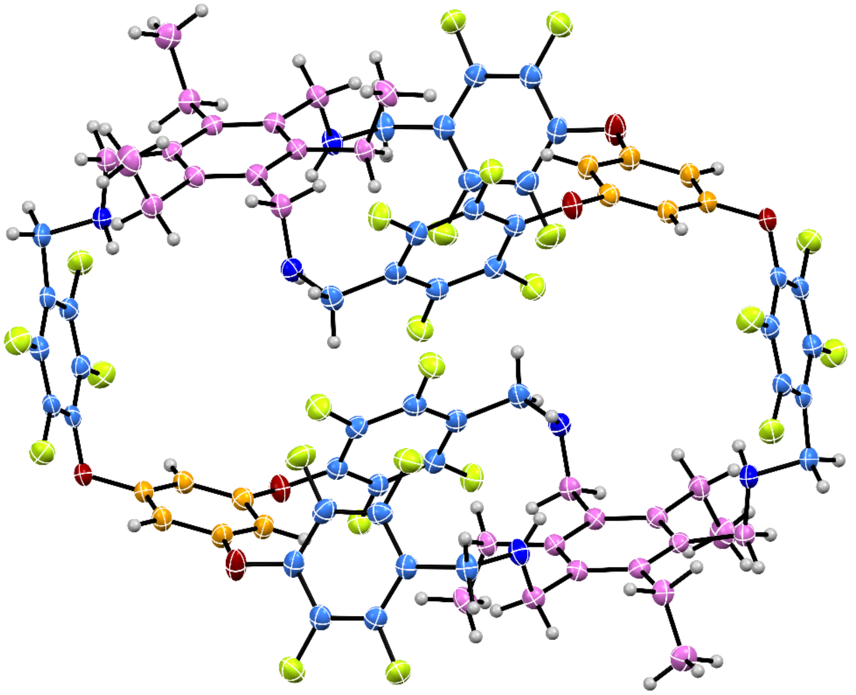

Figure S17: Data set **Et^{2}F$^{2}_{red}$** showing the asymmetric unit (one whole cage), with thermal ellipsoids set at 30% probability and solvents omitted for clarity. The structure was measured at 170 K and solved in triclinic space group *P*-1 with *R*int = 0.092, *R*1 = 0.091, and *wR*2 = 0.26.

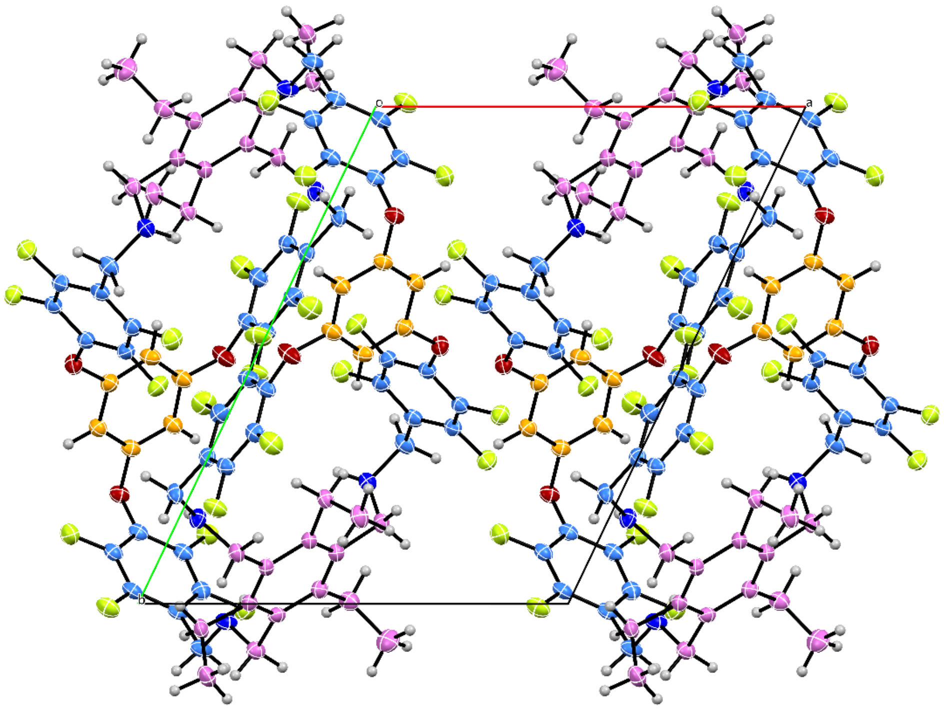

Figure S18: View of the unit cell of **Et^{2}F$^{2}_{red}$** along the crystallographic *a* axis, with solvents omitted for clarity.

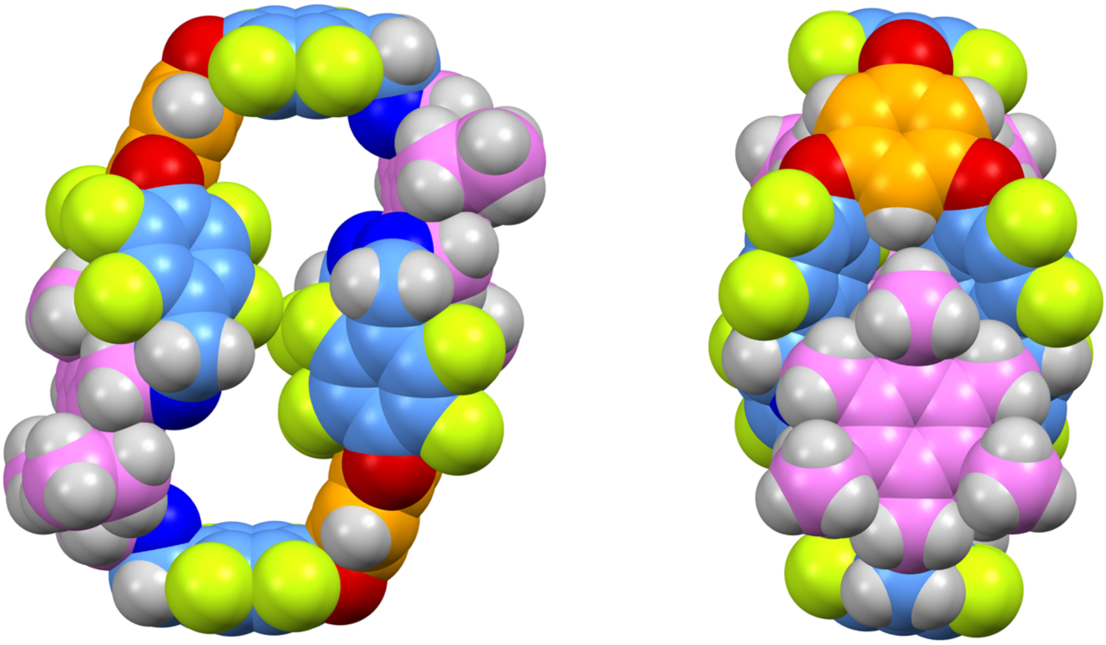

Figure S19: CPK representation of cage $\mathbf{Et^2F^2_{red}}$, showing the macrocycle-like structure of the cage, solvents omitted for clarity.

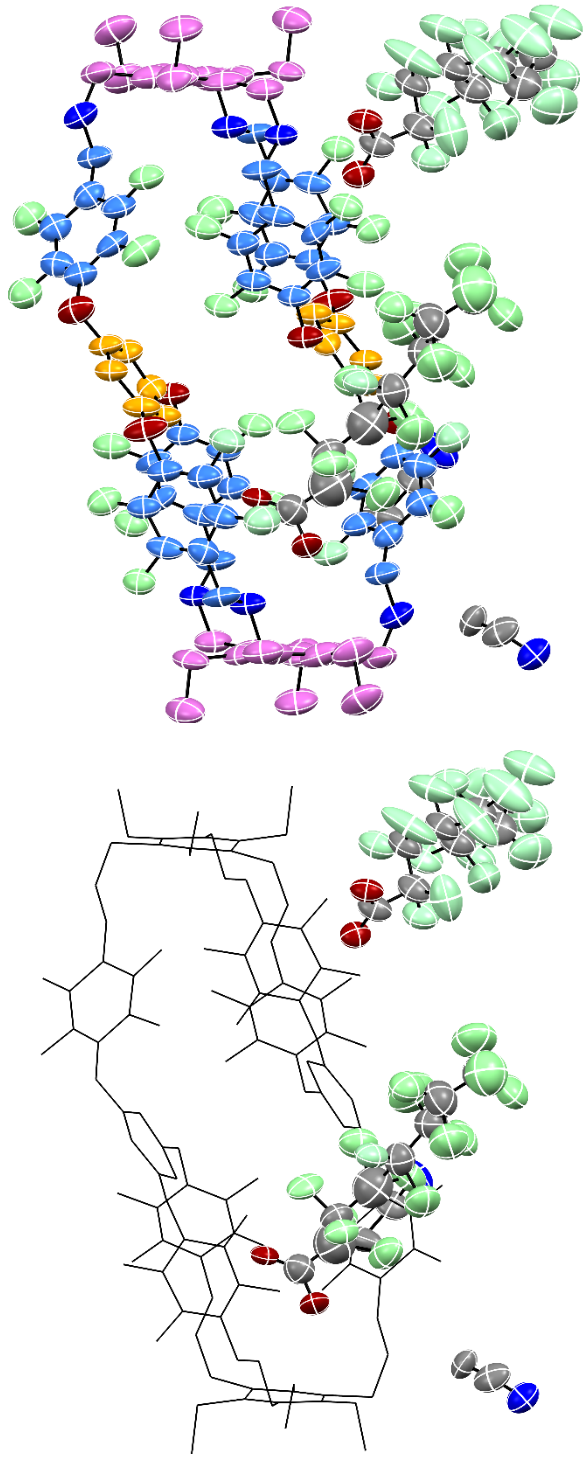

Figure S20: Preliminary data set of **PFOA@Et²F²$_{\mathbf{red}}$** showing the asymmetric unit, with thermal ellipsoids set at 30%. The structure was measured at 150 K and refined in the triclinic space group *P*-1 with *R*int = 0.113, *R*1 = 0.24, and *wR*2 = 0.55.

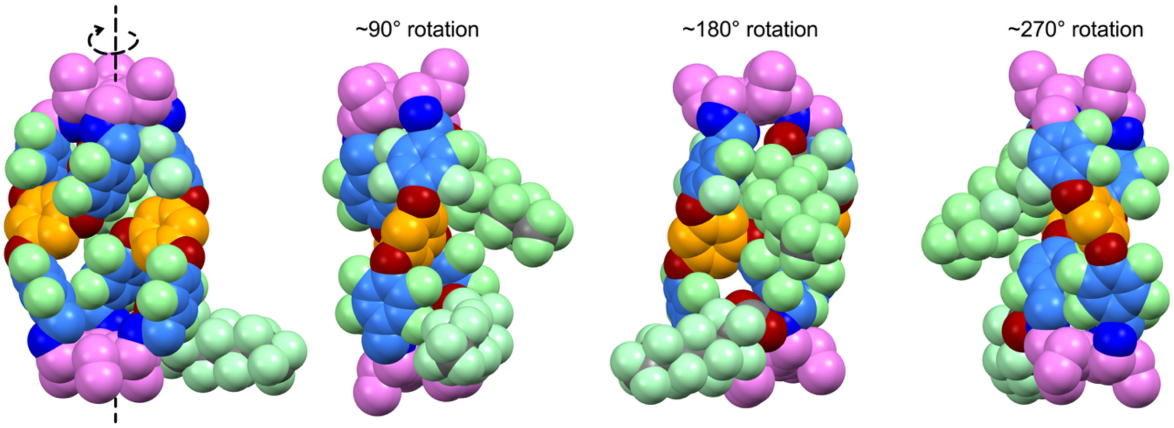

Figure S21: CPK representation of the structure of **PFOA@Et^{2}F$^2_{red}$**, as obtained from the preliminary SC-XRD dataset. From left to right, snapshots of the rotation around the indicated axes are shown to highlight the location of the PFOA molecules in the asymmetric unit.

Table S2: Experimental SC-XRD details.

	**$Et^2F^2_{red}$** (CCDC 2430764)
**Chemical formula**	$C_{84}H_{66}F_{24}N_6O_6\cdot4(CHCl_3)$[+solvent]
***M*$_r$**	2188.90
**Crystal system, space group**	Triclinic, $P\bar{}1$
**Temperature (K)**	170
***a* (Å)**	11.9496 (4), 15.3777 (7), 15.9938 (3)
***V* (Å^3)**	101.316 (3), 99.956 (2), 112.498 (4)
Z	2560.83 (17)
**Radiation type**	1
**m (mm^{-1})**	Cu *K*α
**Crystal size (mm)**	3.81
**Diffractometer**	XtaLAB Synergy, Dualflex, HyPix
**Absorption correction**	Multi-scan *CrysAlis PRO* 1.171.43.98a (Rigaku Oxford Diffraction, 2023) Empirical absorption correction using spherical harmonics, implemented in SCALE3 ABSPACK scaling algorithm
**T_{min}, T_{max}**	0.540, 1.000
**No. of measured, independent and observed [*I* > 2s(*I*)] reflections**	46767, 10699, 8494
**R_{int}**	0.092
**(sin q/l)$_{max}$ (Å^{-1})**	0.640
**$R[F^2 > 2s(F^2)]$, $wR(F^2)$, *S***	0.091, 0.263, 1.06
**No. of reflections**	10699
**No. of parameters**	650
**No. of restraints**	66
**H-atom treatment**	H atoms treated by a mixture of independent and constrained refinement
**Dρ$_{max}$, Dρ$_{min}$ (e Å^{-3})**	1.04, -0.90

VII. DOSY NMR

DOSY NMR experiments were recorded at either 298 K (^{1}H) or 293 K (^{19}F) and calibrated using known self-diffusion values for the solvents used (D_{solv}).[S5] The hydrodynamic radii were estimated using the unmodified Stokes-Einstein equation. This equation was solved for r_H using values for η from the literature.[S6]

$$D = \frac{k_B T}{6\pi\eta r_H} \Rightarrow r_H = \frac{k_B T}{6\pi\eta D}$$

D is the measured diffusion coefficient ($m^2 s^{-1}$)

k_B is the Boltzmann constant ($1.3806485 \cdot 10^{-23}$ $m^2 kg\ s^{-2}\ K^{-1}$)

T is the temperature (K)

r_H is the hydrodynamic radius of the analyte (m)

η is the viscosity of the solvent at temperature T ($kg\ m^{-1}\ s^{-1}$)

VII.a. ^{1}H-DOSY NMR

^{1}H-DOSY NMR experiments were recorded on a Bruker Avance III 600 spectrometer at 25 °C.

Compound	log(D) [log($m^2\ s^{-1}$)]	D [x10^{-10} $m^2\ s^{-1}$]	r_H [Å]
**Et^{2}H^2**	-9.24	5.71	7.08
**Et^{4}H^4**	-9.39	4.05	10.0
**TREN^{4}H^4**	-9.30	5.01	8.07
**Et^{2}F^2**	-9.28	5.27	7.67
**Et^{2}F$^2_{red}$**	-9.31	4.93	8.20
**TREN^{2}F^2**	-9.24	5.75	7.03
**TREN^{2}F$^2_{red}$**	-9.28	5.24	7.72
**Bn^{3}F$^1_{red}$**	-9.22	6.10	6.63

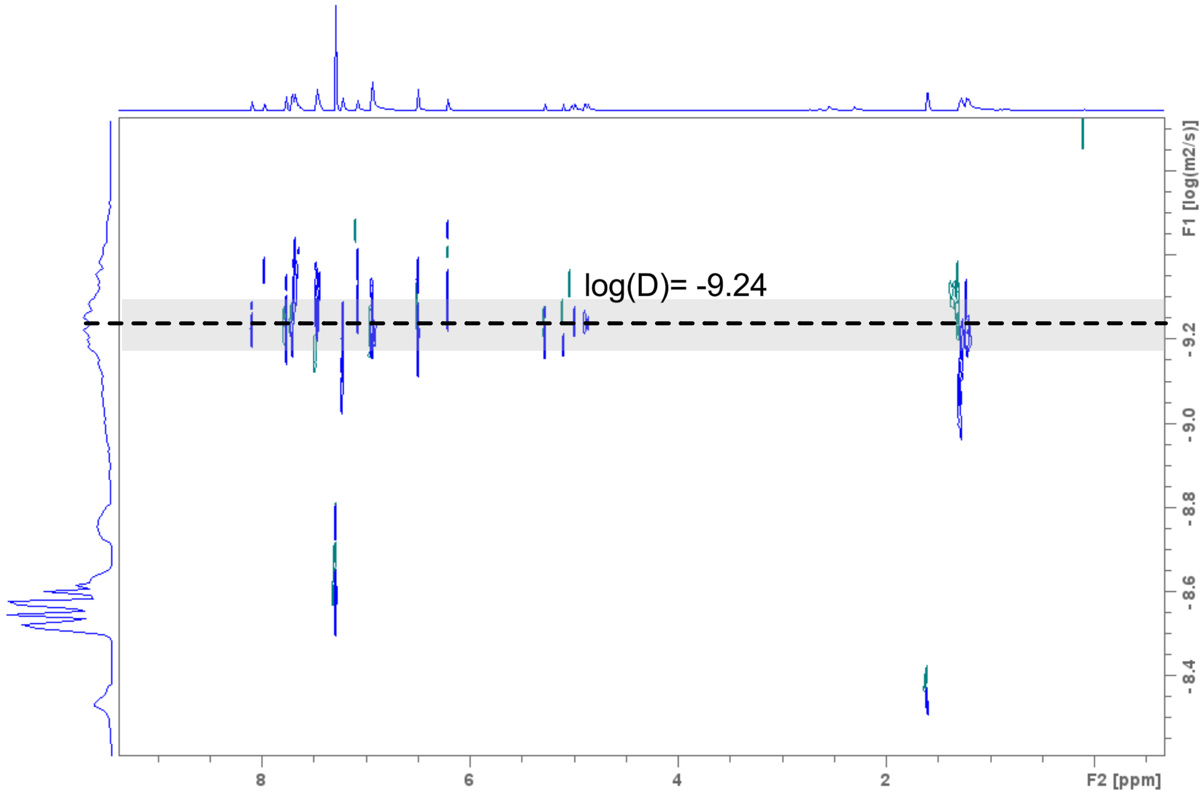

Figure S22: ^{1}H-DOSY NMR (600 MHz, $CDCl_3$, 298 K) of fluorinated imine cage **Et^{2}F^2**.

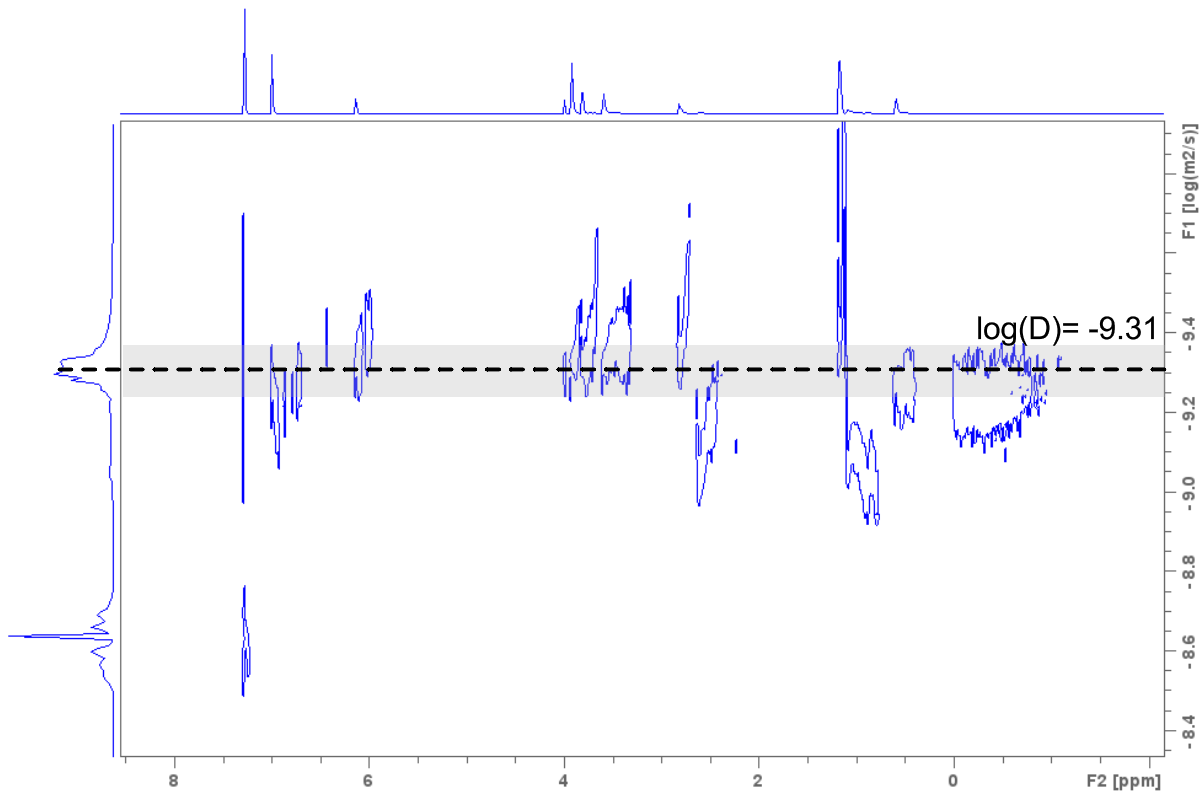

Figure S23: ^{1}H-DOSY NMR (600 MHz, $CDCl_3$, 298 K) of fluorinated amine cage **Et^{2}F^2$_{red}$**.

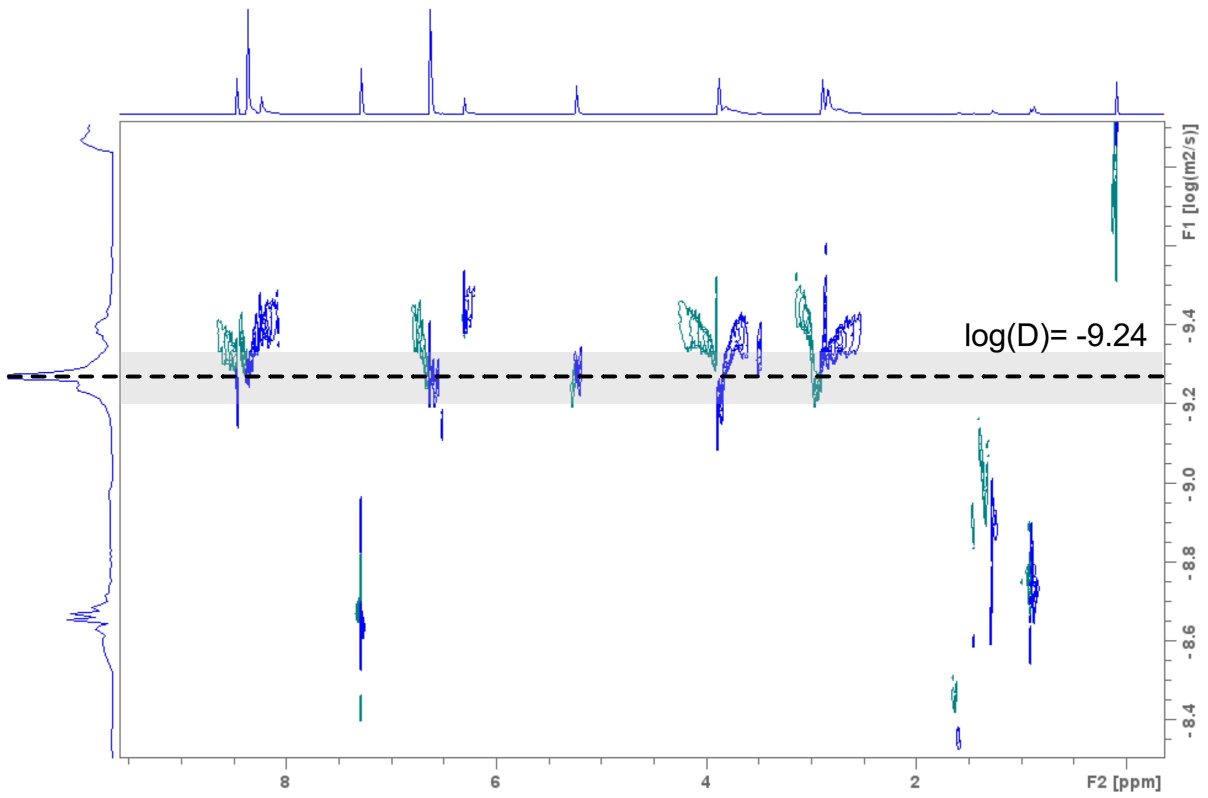

Figure S24: ^{1}H-DOSY NMR (600 MHz, $CDCl_3$, 298 K) of fluorinated imine cage **TREN^{2}F^2**.

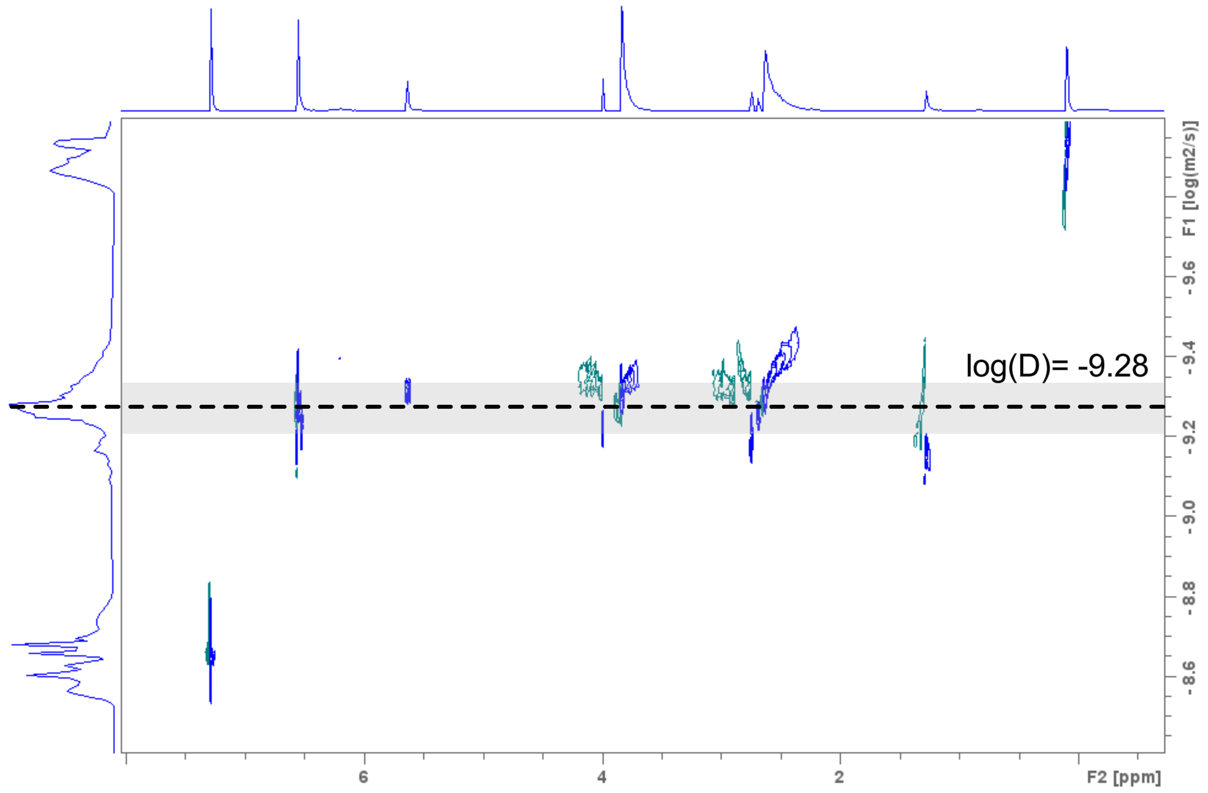

Figure S25: ^{1}H-DOSY NMR (600 MHz, $CDCl_3$, 298 K) of fluorinated amine cage **TREN^{2}F^2$_{red}$**.

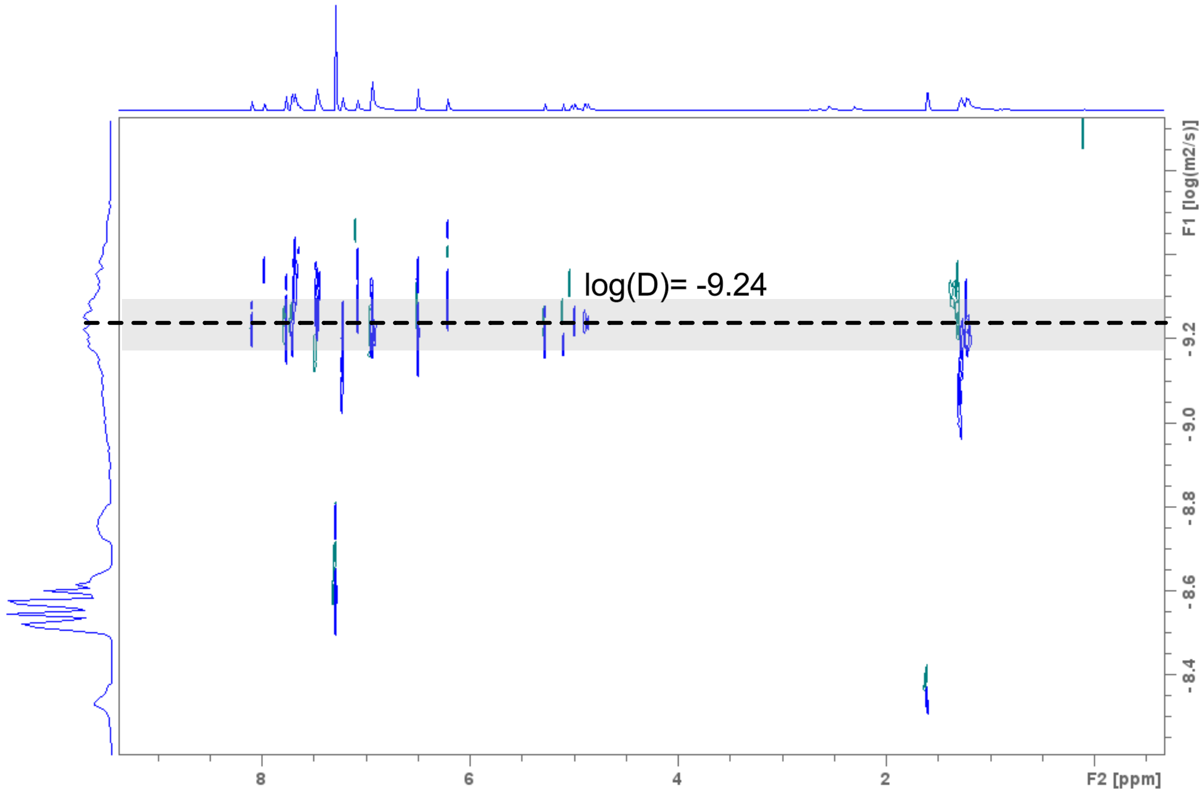

Figure S26: ^{1}H-DOSY NMR (600 MHz, $CDCl_3$, 298 K) of fluorinated imine cage **Et²H²**.

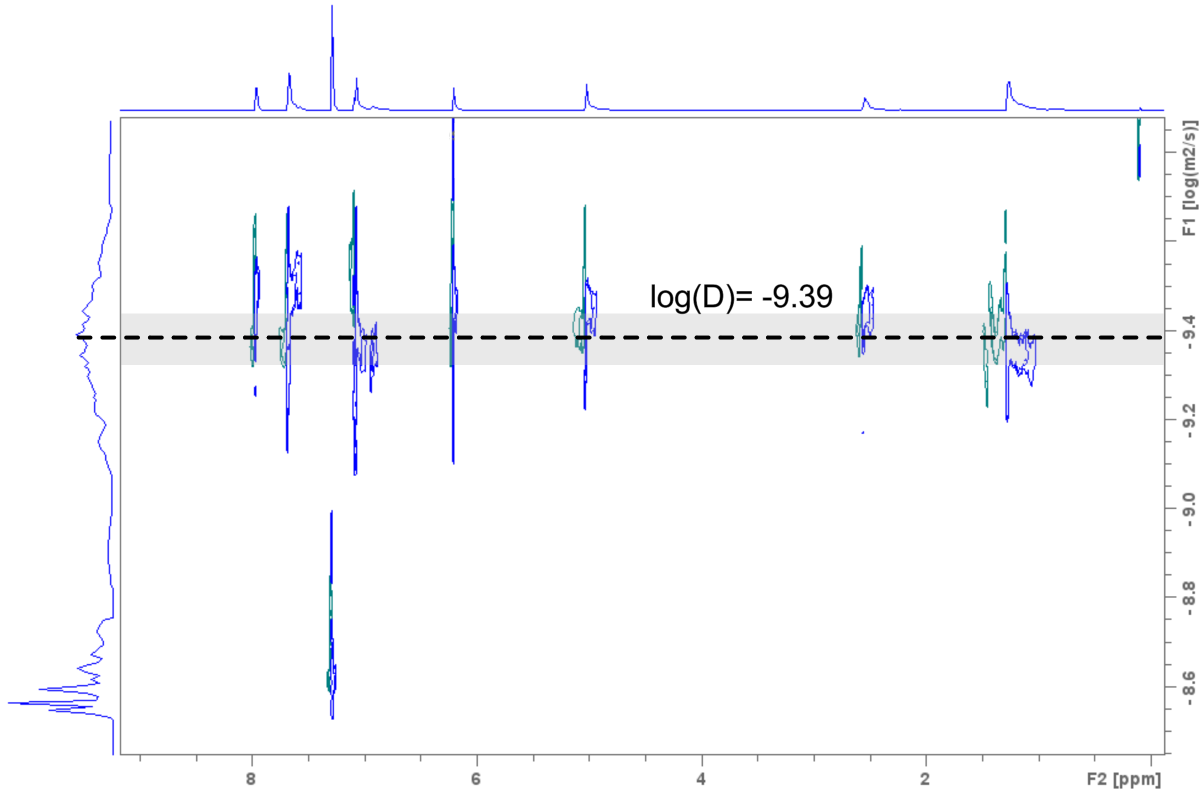

Figure S27: ^{1}H-DOSY NMR (600 MHz, $CDCl_3$, 298 K) of fluorinated imine cage **Et⁴H⁴**.

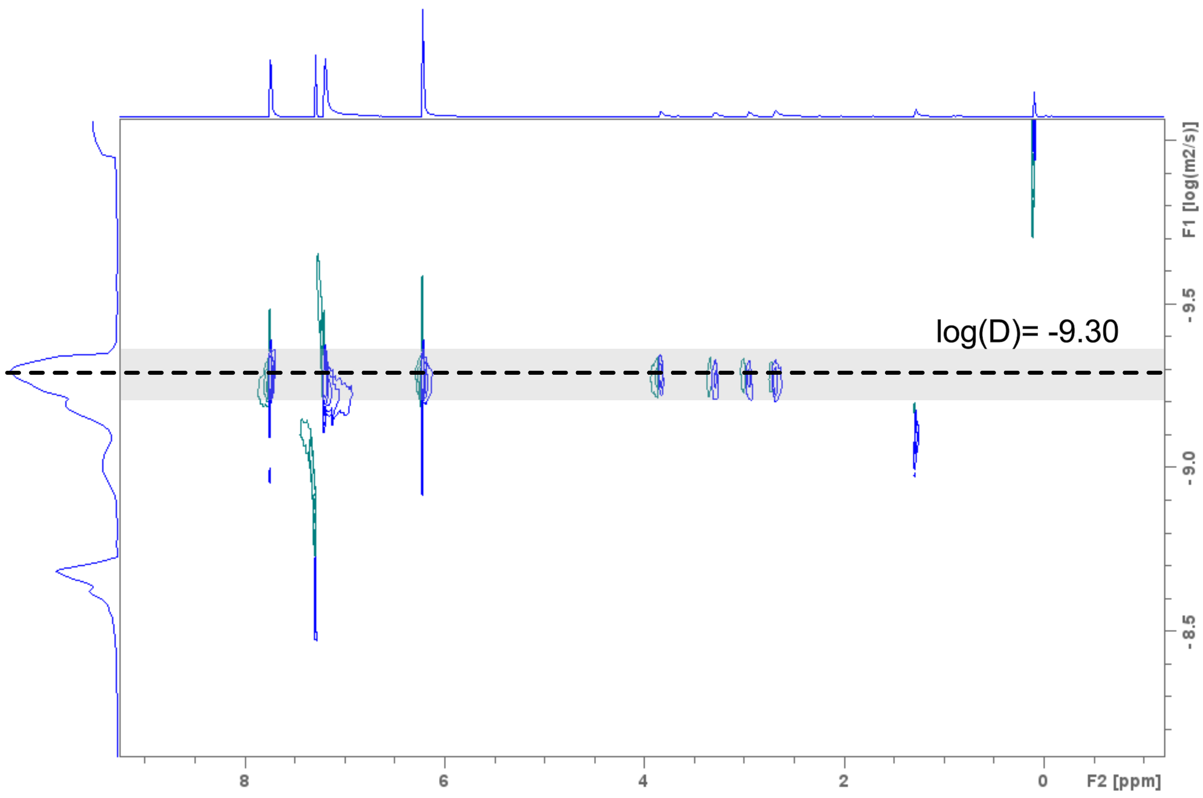

Figure S28: ^{1}H-DOSY NMR (600 MHz, $CDCl_3$, 298 K) of fluorinated imine cage **TREN⁴H⁴**.

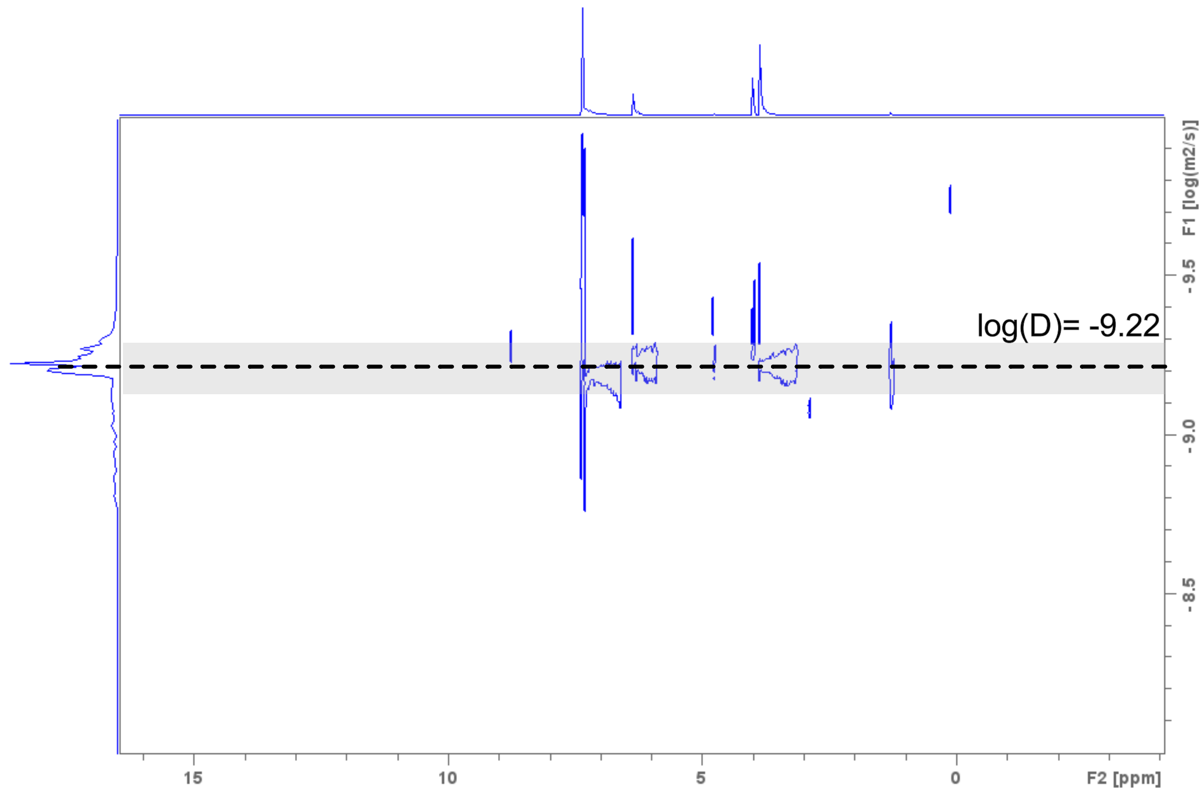

Figure S29: ^{1}H-DOSY NMR (600 MHz, $CDCl_3$, 298 K) of model compound $\mathbf{Bn^3F^1_{red}}$.

VII.b. ^{19}F-DOSY NMR

All samples were measured at a 10 mM concentration. First, each compound was measured individually. After completing these measurements, 2 equivalents of either TFA or PFOA were added while maintaining a constant host concentration. The ^{19}F DOSY NMR was then remeasured. The calculated diffusion coefficients are summarised in Table S3.

Table S3: Summary of ^{19}F DOSY NMR results.

host	guest	D(host) [x10^{-10}m^2 s^{-1}]	D(guest) [x10^{-10}m^2 s^{-1}]
–	PFOA	–	0.105
–	TFA	–	0.146
**Et^{2}F$^2_{red}$**	–	3.64	–
**Et^{2}F$^2_{red}$**	PFOA	3.62	3.81
**Et^{2}F$^2_{red}$**	TFA	3.63	4.17
**TREN^{2}F$^2_{red}$**	–	3.89	–
**TREN^{2}F$^2_{red}$**	PFOA	3.21	3.41
**TREN^{2}F$^2_{red}$**	TFA	3.35	3.85
**Bn^{3}F$^1_{red}$**	–	5.72	–
**Bn^{3}F$^1_{red}$**	PFOA	4.75[a]	5.36
**Bn^{3}F$^1_{red}$**	TFA	5.52	7.94

[a] host-signals in ^{19}F DOSY NMR were observed to be comparatively broad.

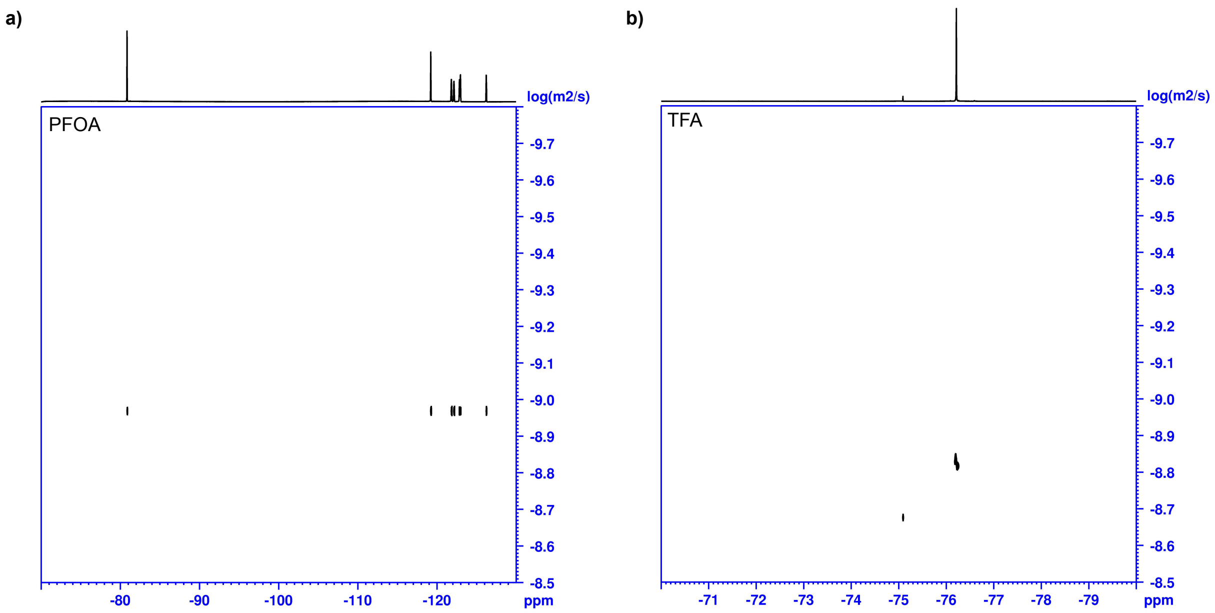

Figure S30: ^{19}F DOSY NMR (565 MHz, $CDCl_3$, 293 K) of a) PFOA and b) TFA at a concentration of 10 mM in $CDCl_3$:MeOD (95:5).

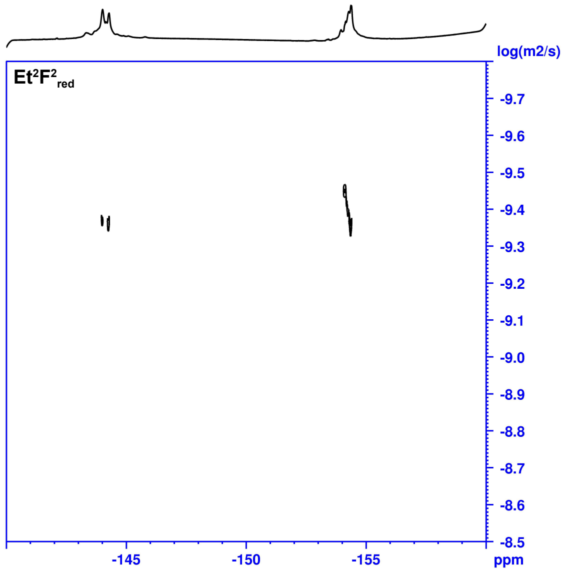

Figure S31: ^{19}F DOSY NMR (565 MHz, $CDCl_3$, 293 K) of $\mathbf{Et^2F^2_{red}}$ at a concentration of 10 mM in $CDCl_3$:MeOD (95:5).

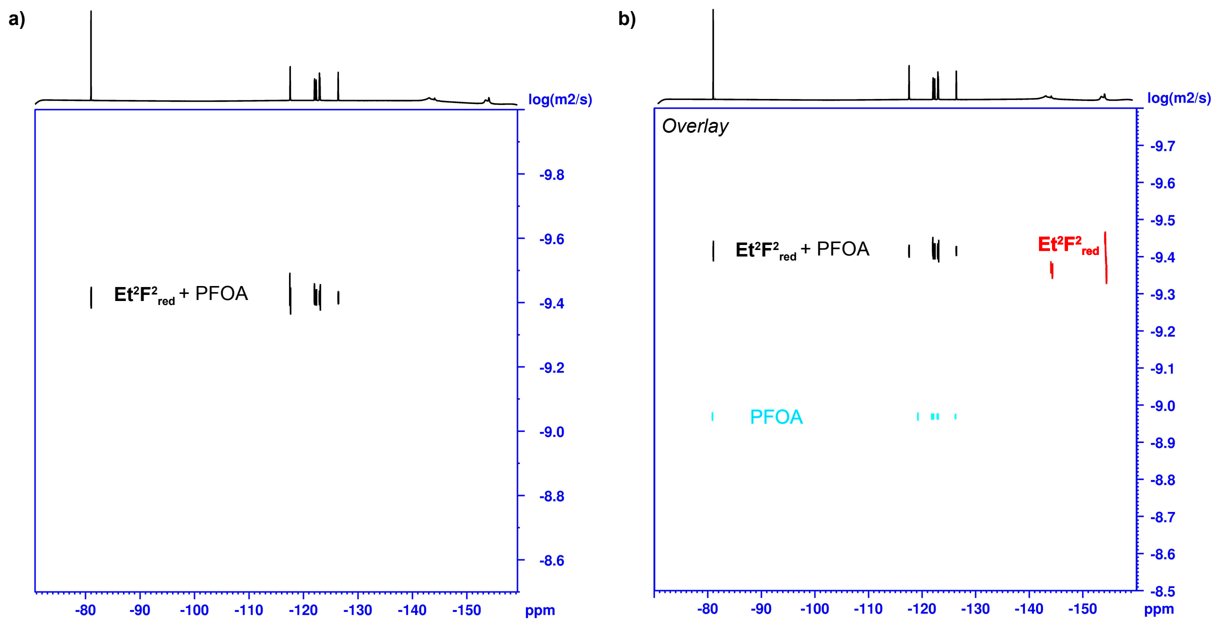

Figure S32: ^{19}F DOSY NMR (565 MHz, $CDCl_3$, 293 K) of a) **$Et^2F^2_{red}$** after addition of PFOA at a concentration of 10 mM in $CDCl_3$:MeOD (95:5) and b) overlay of the host (red), guest (turquoise) and host-guest (black) ^{19}F DOSY NMR spectra.

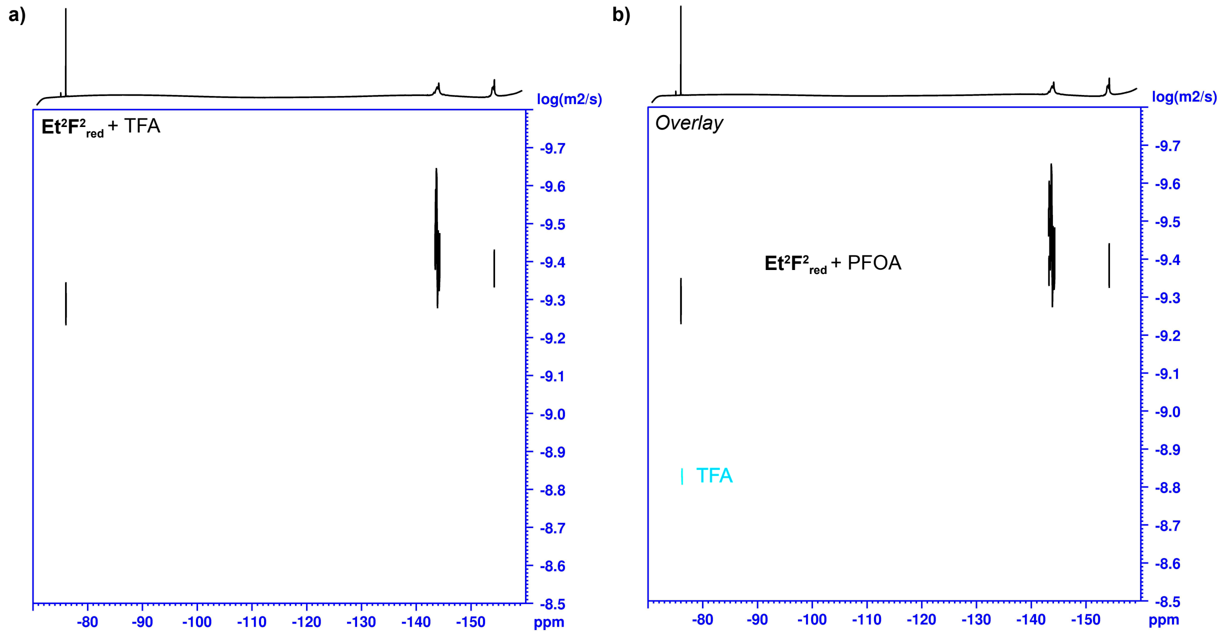

Figure S33: ^{19}F DOSY NMR (565 MHz, $CDCl_3$, 293 K) of a) **$Et^2F^2_{red}$** after addition of TFA at a concentration of 10 mM in $CDCl_3$:MeOD (95:5) and b) overlay of guest (turquoise) and host-guest (black) ^{19}F DOSY NMR spectra.

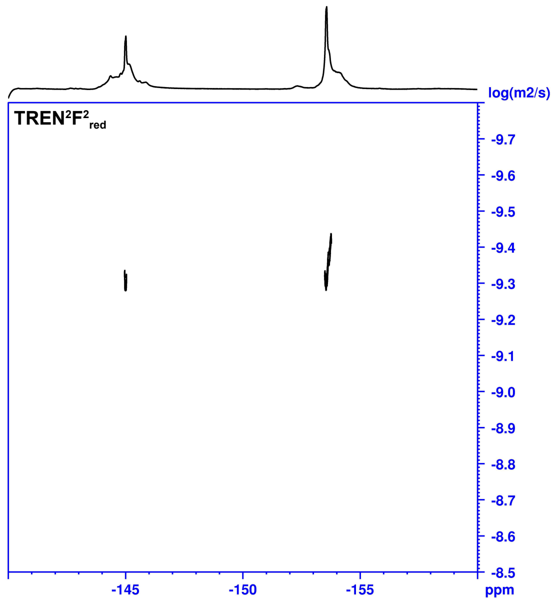

Figure S34: ^{19}F DOSY NMR (565 MHz, $CDCl_3$, 293 K) of **TREN^{2}F^2$_{red}$** at a concentration of 10 mM in $CDCl_3$:MeOD (95:5).

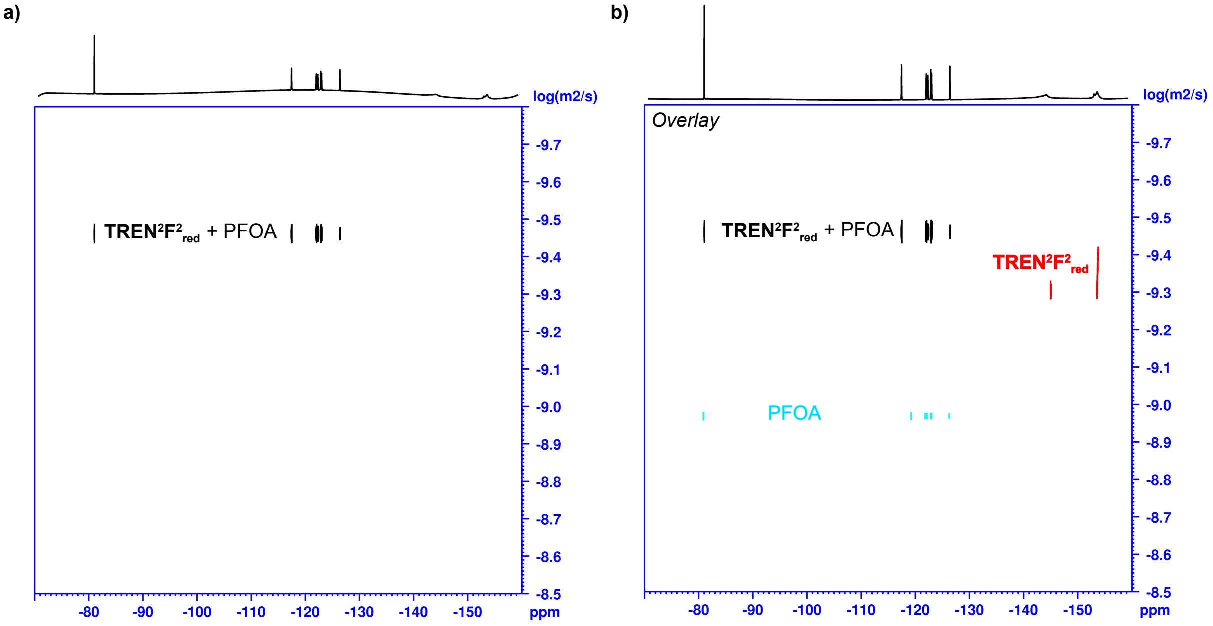

Figure S35: ^{19}F DOSY NMR (565 MHz, $CDCl_3$, 293 K) of a) **TREN^{2}F^2$_{red}$** after addition of PFOA at a concentration of 10 mM in $CDCl_3$:MeOD (95:5) and b) overlay of the host (red), guest (turquoise) and host-guest (black) ^{19}F DOSY NMR spectra.

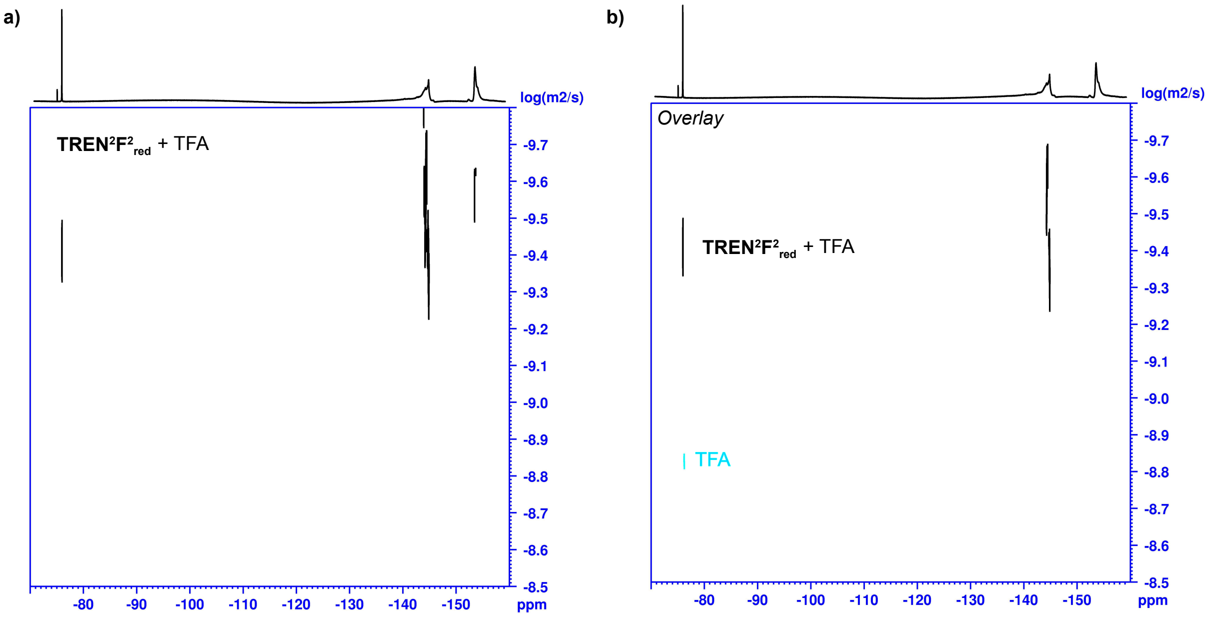

Figure S36: ^{19}F DOSY NMR (565 MHz, $CDCl_3$, 293 K) of a) **TREN^{2}F^2$_{red}$** after addition of TFA at a concentration of 10 mM in $CDCl_3$:MeOD (95:5) and b) overlay of the guest (turquoise) and host-guest (black) ^{19}F DOSY NMR spectra.

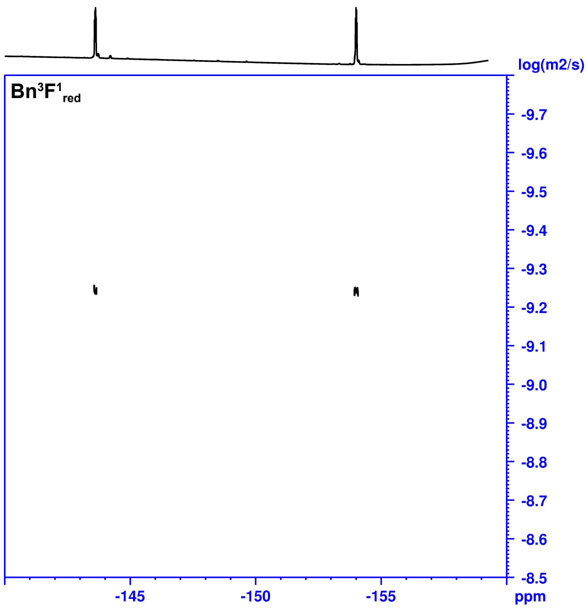

Figure S37: ^{19}F DOSY NMR (565 MHz, $CDCl_3$, 293 K) of **Bn^{3}F^1$_{red}$** at a concentration of 10 mM in $CDCl_3$:MeOD (95:5).

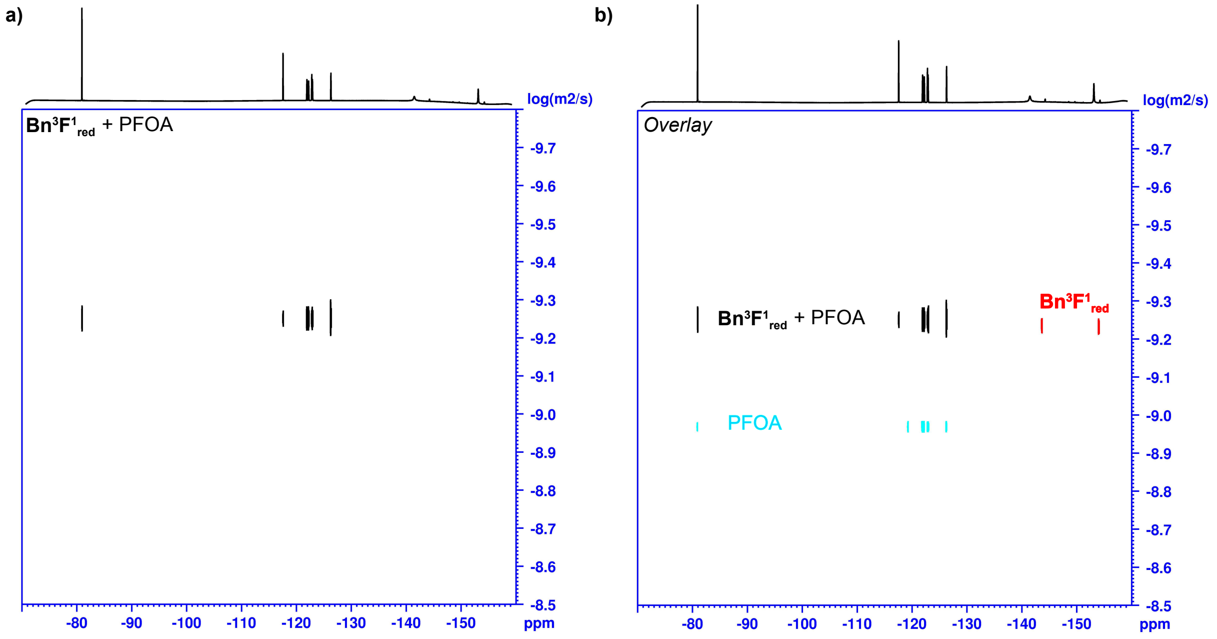

Figure S38: ^{19}F DOSY NMR (565 MHz, $CDCl_3$, 293 K) of a) $\mathbf{Bn^3F^1_{red}}$ after addition of PFOA at a concentration of 10 mM in $CDCl_3$:MeOD (95:5) and b) overlay of the host (red), guest (turquoise) and host-guest (black) ^{19}F DOSY NMR spectra.

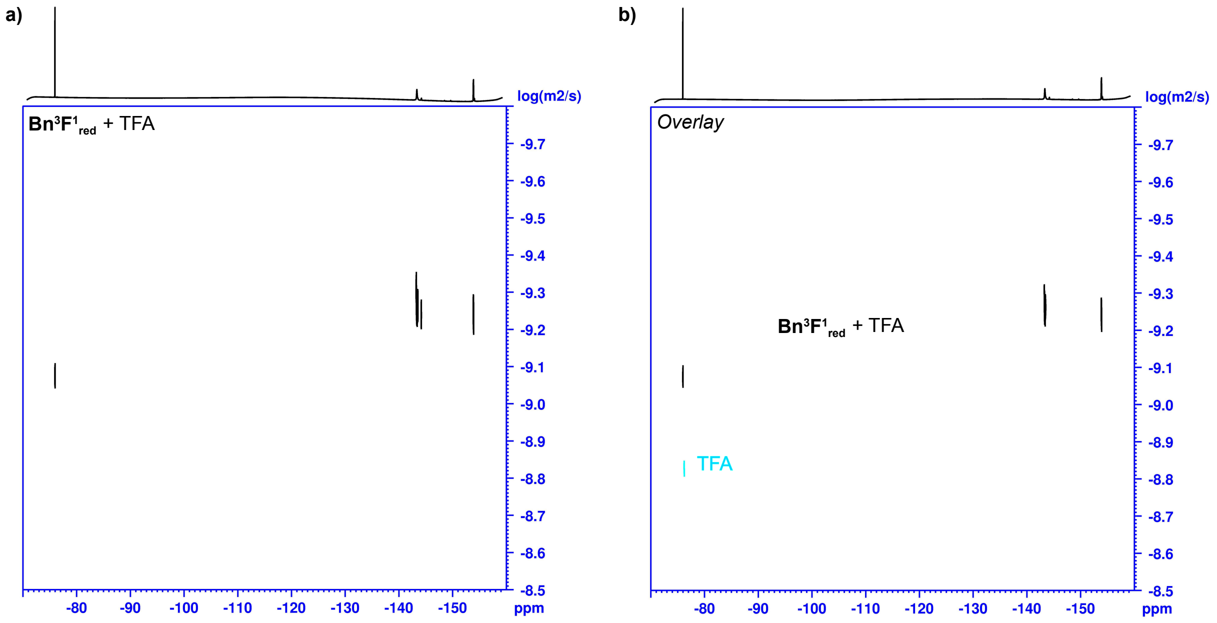

Figure S39: ^{19}F DOSY NMR (565 MHz, $CDCl_3$, 293 K) of a) $\mathbf{Bn^3F^1_{red}}$ after addition of TFA at a concentration of 10 mM in $CDCl_3$:MeOD (95:5) and b) overlay of the guest (turquoise) and host-guest (black) ^{19}F DOSY NMR spectra.

VIII. NMR Titrations

The samples were dissolved in a mixture of $CDCl_3/CD_3OD$ 95:5 (v/v), 2.5 mM, employing C_6F_6 as an external standard for ^{19}F NMR, chemical shift referenced to -164.9 ppm. Solutions were prepared freshly before each measurement. The samples were measured on a Bruker Avance III – 300 (^{1}H NMR: 300 MHz, $^{19}F\{^{1}H\}$ NMR: 282 MHz at 298 K). Each titration profile was plotted following the proton signal of the Ar_F-CH_2 bridge towards the NH (proton H_A). A Job's plot was performed on each titration to get the stoichiometry of complexes.

Discussion of the results:

Titrations with PFOA were performed from two viewpoints, one being the respective supramolecular compound, and the second being PFOA. This was done as, aside from host-guest interactions, protonation is an expected side reaction occurring. Therefore, multiple control experiments were conducted. Overall, cage **$TREN^2F^2_{red}$** showed interactions corresponding in part to an acid-base reaction. Titrations with **$TREN^2F^2_{red}$** and PFOA showed noticeable shifts in the ^{1}H and ^{19}F NMR. The addition of PFOA to the cage mainly led to the CH_2-groups near the nitrogen shifting downfield during the addition, furthermore, small shifts for the cage signals as well as a broadening were observed in the ^{19}F NMR. However, no clear maximum could be observed in the corresponding Job's plot, supposedly due to the protonation of the cage happening at the same time. If the cage was added to PFOA, however, a clear shift for C-2 and even the terminal C-8 of PFOA was observed, while a stoichiometry of 1:1 for PFOA:**$TREN^2F^2_{red}$** was indicated from the obtained Job's plot. Meanwhile, adding the stronger acid TFA to a solution of the cage only resulted in marginal shifts of the cage signals in ^{1}H and ^{19}F NMR when added to the cage, rendering this observation to predominantly be a result of protonation, while PFOA shows additional interactions with **$TREN^2F^2_{red}$**.

This interaction was also observed with the model compound **$Bn^3F^1_{red}$**. Upon adding PFOA, clear shifts in the fluorine signal of C-2 were observed, while the other fluorine signals remained largely unchanged. This suggests that deprotonation is the primary process occurring, rather than "classical" host-guest interactions. A similar effect was observed when PFOA was added to a solution of **$Bn^3F^1_{red}$**. The CH_2-N-CH_2 motif exhibited noticeable shifts, whereas the phloroglucinol proton remained unchanged. However, both the benzyl group and the perfluorobenzene motif experienced downfield shifts. A comparable, though less pronounced, effect was observed upon the addition of TFA to a **$Bn^3F^1_{red}$** solution, indicating that, unlike in the case of the cages, the observed shifts are primarily due to protonation of the NH group.

This contrasting behaviour is exemplified by cage **$Et^2F^2_{red}$**. Upon adding PFOA to a solution of the cage, nearly all signals in both ^1H and ^{19}F NMR exhibited noticeable shifts. The protons associated with the phloroglucinol motif shifted upfield, while the CH_2 groups adjacent to nitrogen shifted downfield, as did all ^{19}F cage signals. Interestingly, the isolated, strongly upfield-shifted ethyl group (H_g and H_f, see Fig. 2 and Fig. 4b) within the **Et** motif experienced a downfield shift upon PFOA addition, eventually merging with the signals of the other two ethyl groups present in the desymmetrised **Et** motif (H_G and H_F). This is strongly suggesting a structural change upon PFOA addition, where the ethyl group pointing towards the inner cavity is pushed out. These changes do not occur when titrating octanoic acid with a comparable pK_a (3.8 ± 0.1 and 2.2 ± 0.2, respectively)[S7] or the much stronger acid TFA, further supporting a PFOA-induced structural change (induced fit), rather than simple protonation, rendering the observed structural change to be a result of the interplay between electrostatic and fluorophilic interactions between PFOA and **Et^2F^2red**, selectively. Both series of titrations performed with PFOA as guest indicate a PFOA:**$Et^2F^2_{red}$** stoichiometry of 2:1.

^{19}F DOSY experiments were conducted by first measuring the host alone. Afterward, the guest was added, and the DOSY spectra of the resulting "host-guest" mixture were remeasured (for details see section VII.b.). The experiments supported the formation of host-guest assemblies between PFOA and the investigated hosts. In the host-guest mixture, the diffusion coefficient of PFOA was drastically lowered, very closely matching the diffusion coefficient of the host without the guest, overall showing comparatively sharp signals, indicating the formation of the respective complexes. TFA, on the other hand, showed diffusion coefficients noticeably lower than the ones of the host. Overall, the signals were broadened, thus indicating that at least some attractive interactions between TFA and the hosts are present, supposedly stemming from comparatively weaker attractive electrostatic effect. Surprisingly even **$Bn^3F^1_{red}$**, although no significant shifting apart from the C-2 fluorine atoms of PFOA during the titrations could be observed, showed the formation of a "host-guest" complex.

To understand PFOA binding, titrations with a neutral TEA^+PFOA^- mixture were performed, showing only negligible shifts with all compounds, strongly hinting that the $R_2NH_2^+$ species formed upon the addition of an acid is a strong contributing factor, especially in organic media. This is similar to effects observed by other groups, that either the introduction of NH_2-groups or the quaternisation of R_3N- or R_2NH-groups significantly boosted the PFAS removal abilities of COFs and polymer membranes, due to a proposed interplay of electrostatic and hydrophobic interactions.[S8–S11] As TFA and octanoic acid show no clear signs of adduct formation, this seems to be a likely explanation for the observed complex formation between the hosts and PFOA. When comparing the shifts of the PFOA ^{19}F NMR signals upon addition of the different hosts, only the cages cause noticeable changes beyond the C-2 fluorine atoms, with the terminal CF_3-group showing the most pronounced shift apart from C-2. This suggests that while

all hosts can interact with PFOA through electrostatic attraction to its anionic carboxylate group, only the cages induce significant shielding, pointing to a closer spatial association. Preliminary SC-XRD data indicates that the perfluoroalkyl chain of PFOA remains mostly outside the cage, but in solution, the terminal CF_3-group likely partially inserts into the cage window, interacting with the fluorinated panels, being shielded in the process, explaining the observed shifts. This interaction, however, appears to be relatively weak, with electrostatic attraction likely being the main contributing factor, while the observed 'encapsulation' seems to result primarily from the spatial proximity between the perfluoroalkyl chain and the fluorinated panels of the cage.

Lastly, ^{1}H-^{1}H NOESY, ^{19}F-^{19}F NOESY and ^{19}F-^{1}H HOESY experiments were performed to gain a clearer understanding of the binding mode and structural changes associated with **$Et^2F^2_{red}$** taking place. The ^{19}F correlation experiments, however, did not reveal any significant signs of NOEs between the perfluoroalkyl chain of PFOA and the cage signals, supporting the assumption that electrostatic interactions play the dominant role and interactions between the PFOA chain and the cage are rather loose and of dynamic nature. However, it should be noted here that due to the limitations in solubility, the general broadening of the cage signals upon addition of PFOA hindered the acquisition of good quality spectra. Thus, the absence of NOEs can also likely be a result of the low signal-to-noise ratio.

Analogous to the chemical shift changes observed upon PFOA addition, the ^{1}H-^{1}H NOESY spectra before and after addition indicate subtle structural changes in the cage. In the absence of PFOA, the desymmetrised ethyl group (H_f and H_g) shows NOEs only within itself, and no NOEs are observed for the phloroglucinol motif. Upon the addition of PFOA, new NOE cross peaks appear. Specifically, the symmetric protons of the phloroglucinol motif (H_D) and all ethyl group protons (H_f and H_g) exhibit NOEs with the CH_2–N–CH_2 motif. This suggests that all ethyl groups become more conformationally similar upon PFOA binding, which corresponds well with the 'elongated' cage structure observed by SC-XRD and can be explained by the less restricted motion of the ethyl groups in this conformation, allowing closer spatial proximity between previously distant regions.

VIII.a Titrations of $Et^2F^2_{red}$

$Et^2F^2_{red}$ with addition of PFOA

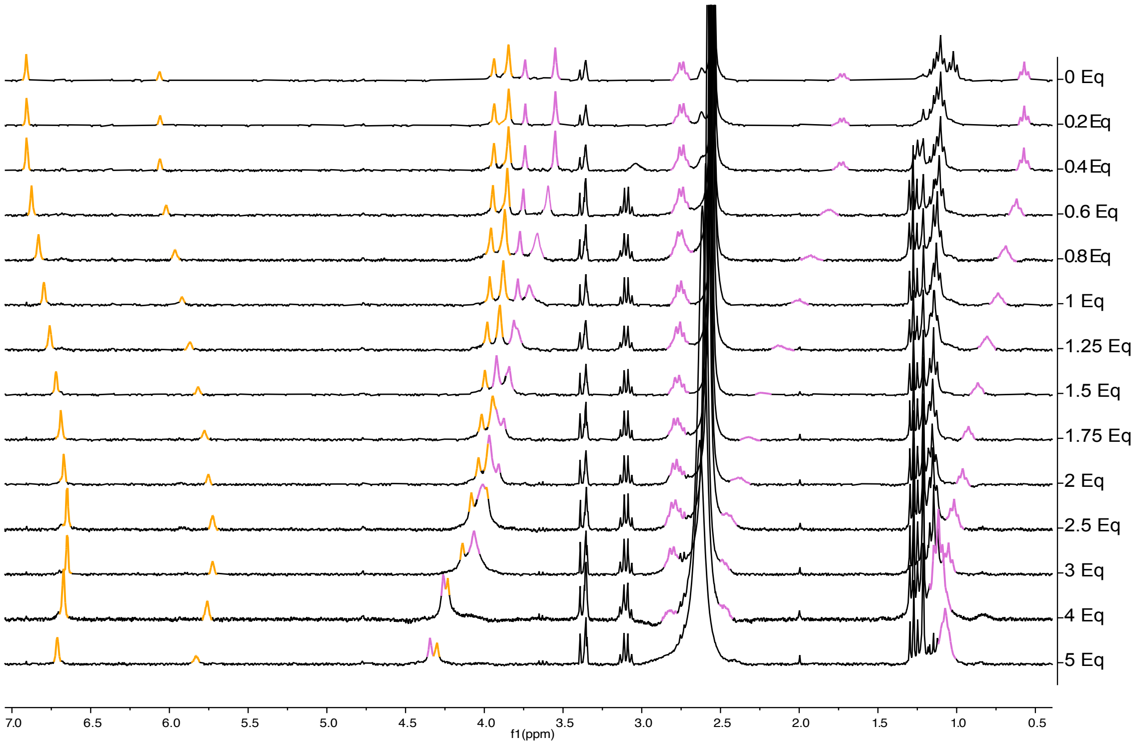

Figure S40: ^{1}H NMR spectral changes observed in **$Et^2F^2_{red}$** (c = 2.5 mM) in $CDCl_3/CD_3OD$ (95:5, v/v) with the addition of up to 5 equiv. of PFOA.

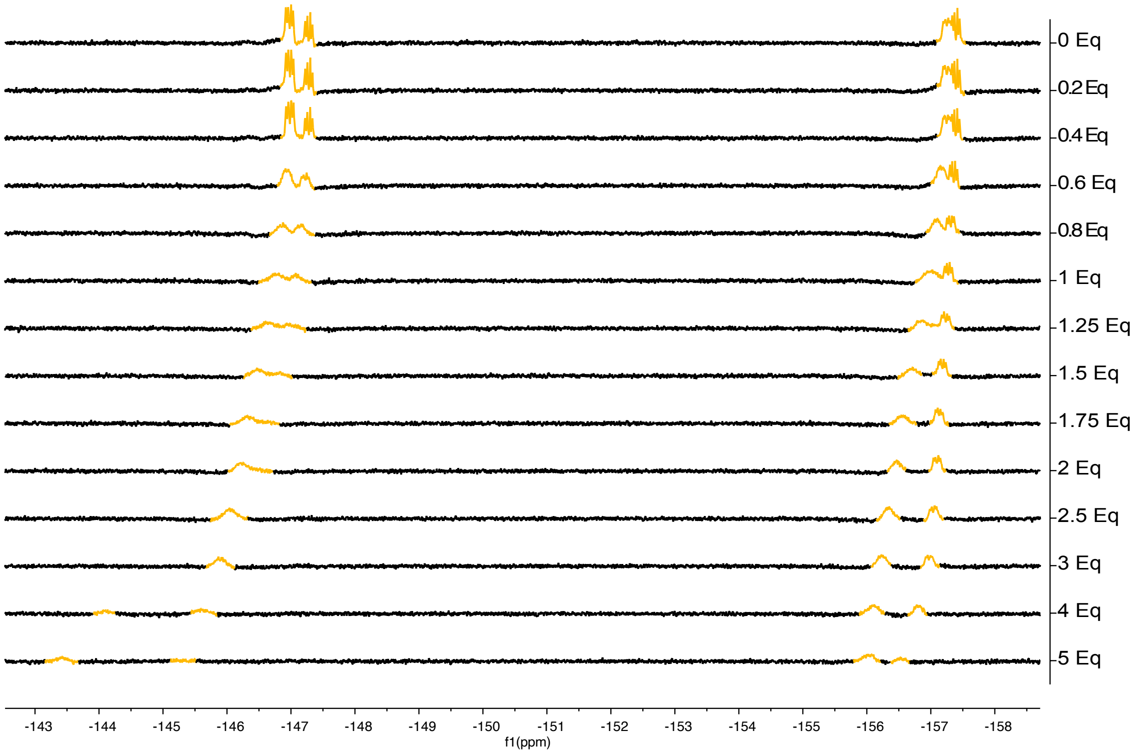

Figure S41: ^{19}F NMR spectral changes observed in **$Et^2F^2_{red}$** (c = 2.5 mM) in $CDCl_3/CD_3OD$ (95:5, v/v) with the addition of up to 5 equiv. of PFOA.

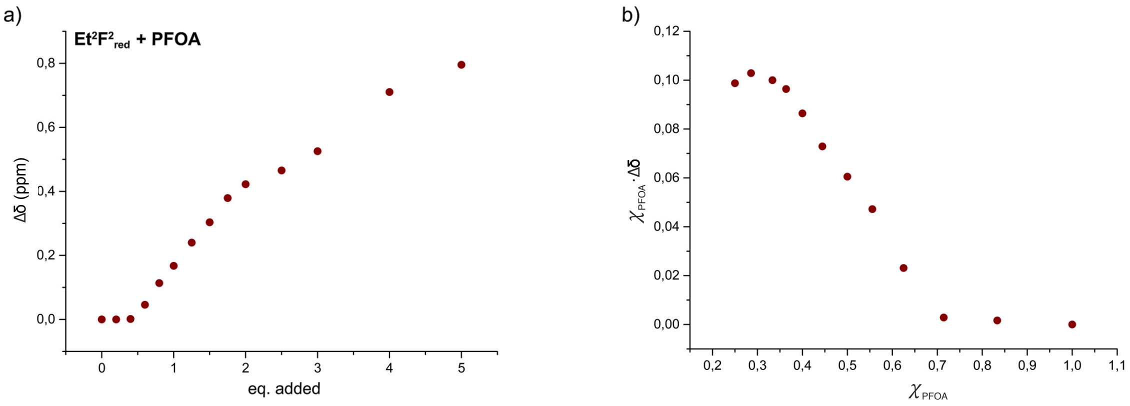

Figure S42: a) Titration profile of $\mathbf{Et^2F^2_{red}}$ upon PFOA addition, following H_A shifting and b) the resulting Job's plot of the titration, showing a maximum at 0.33, indicating a 2:1 stoichiometry between $\mathbf{Et^2F^2_{red}}$ and PFOA.

PFOA with addition of $Et^2F^2_{red}$

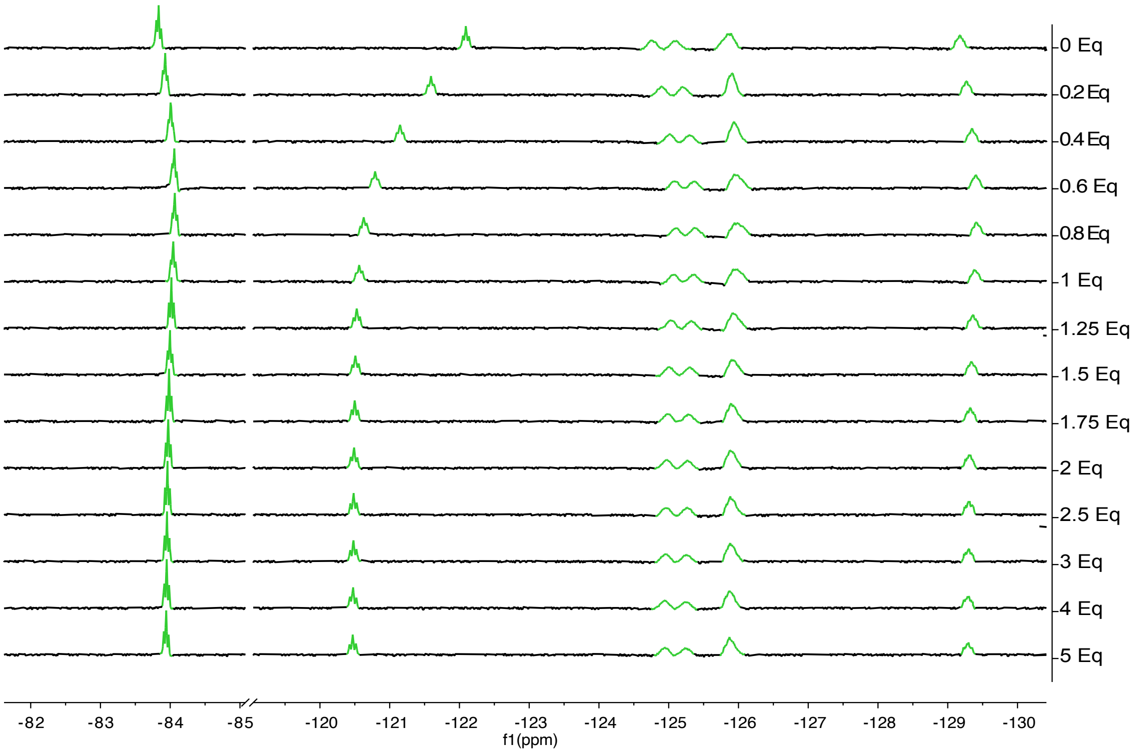

Figure S43: ^{19}F NMR spectral changes observed in PFOA (c = 2.5 mM) in $CDCl_3/CD_3OD$ (95:5, v/v) with the addition of up to 5 equiv. of $\mathbf{Et^2F^2_{red}}$.

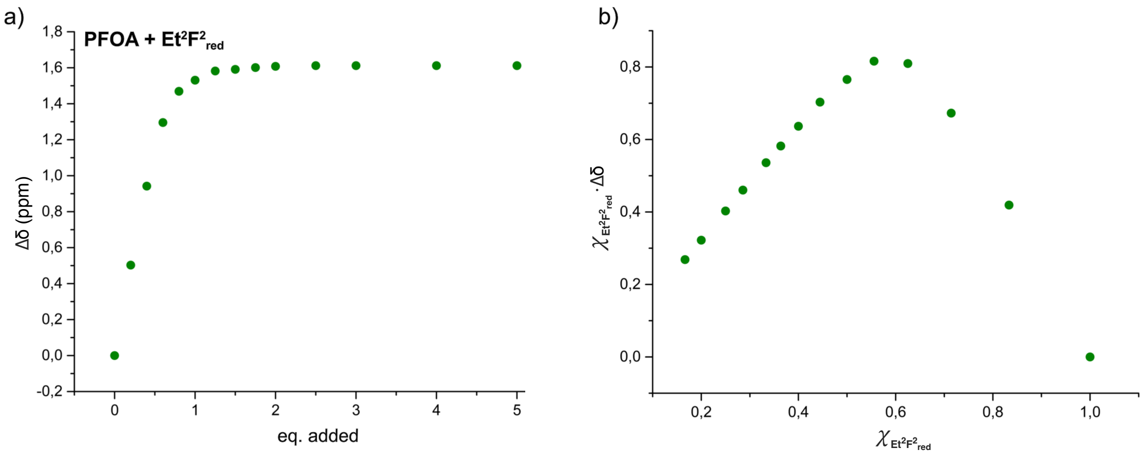

Figure S44: a) Titration profile of PFOA upon addition of $\mathbf{Et^2F^2_{red}}$ and b) Job's plot of titration, showing a maximum at 0.66, indicating a 2:1 stoichiometry between PFOA and $\mathbf{Et^2F^2_{red}}$.

$Et^2F^2_{red}$ with octanoic acid

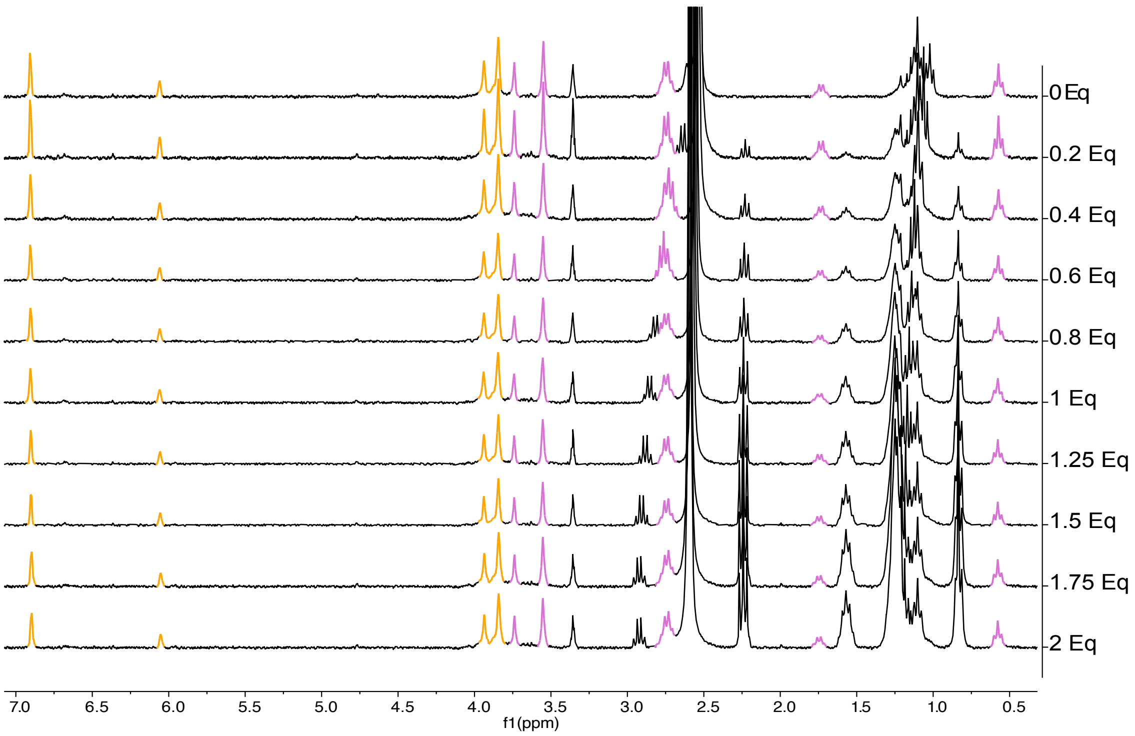

Figure S45: ^{1}H-NMR spectral changes observed in $\mathbf{Et^2F^2_{red}}$ (c = 2.5 mM) in $CDCl_3/CD_3OD$ (95:5, v/v) with the addition of up to 2 equiv. of octanoic acid.

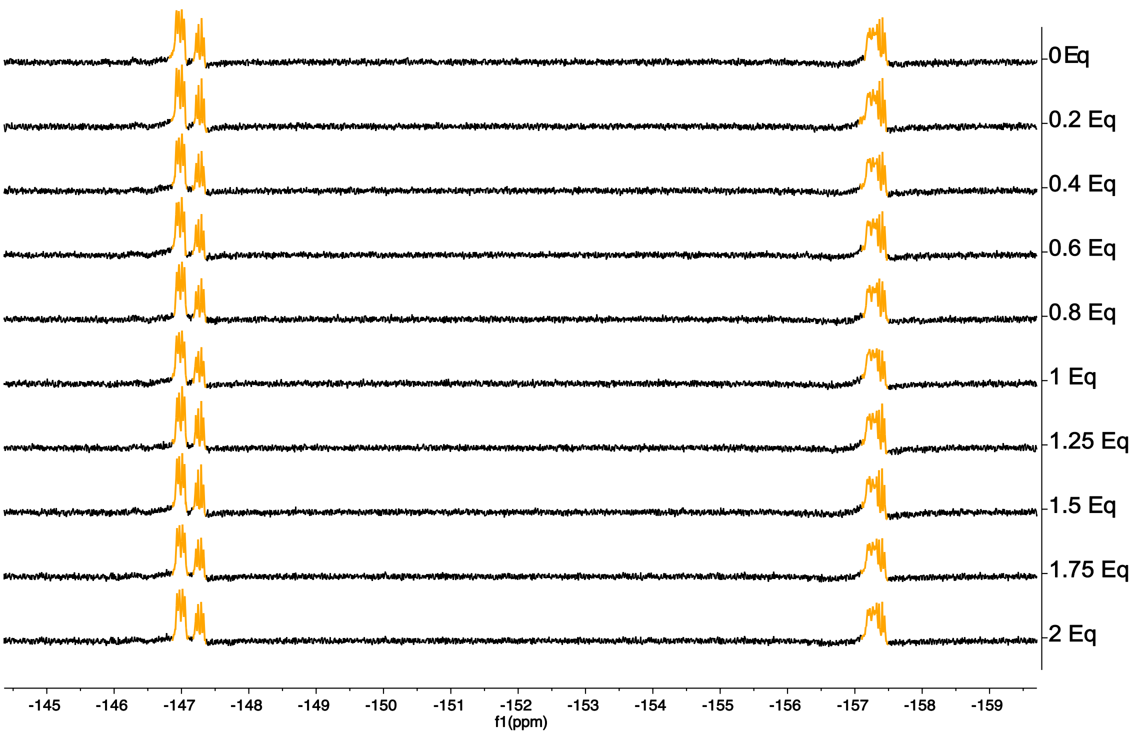

Figure S46: ^{19}F NMR spectral changes observed in **Et^{2}F^2$_{red}$** (c = 2.5 mM) in $CDCl_3/CD_3OD$ (95:5, v/v) with the addition of up to 2 equiv. of octanoic acid.

Et^{2}F^2$_{red}$ with addition of TEA$^+$PFOA$^-$

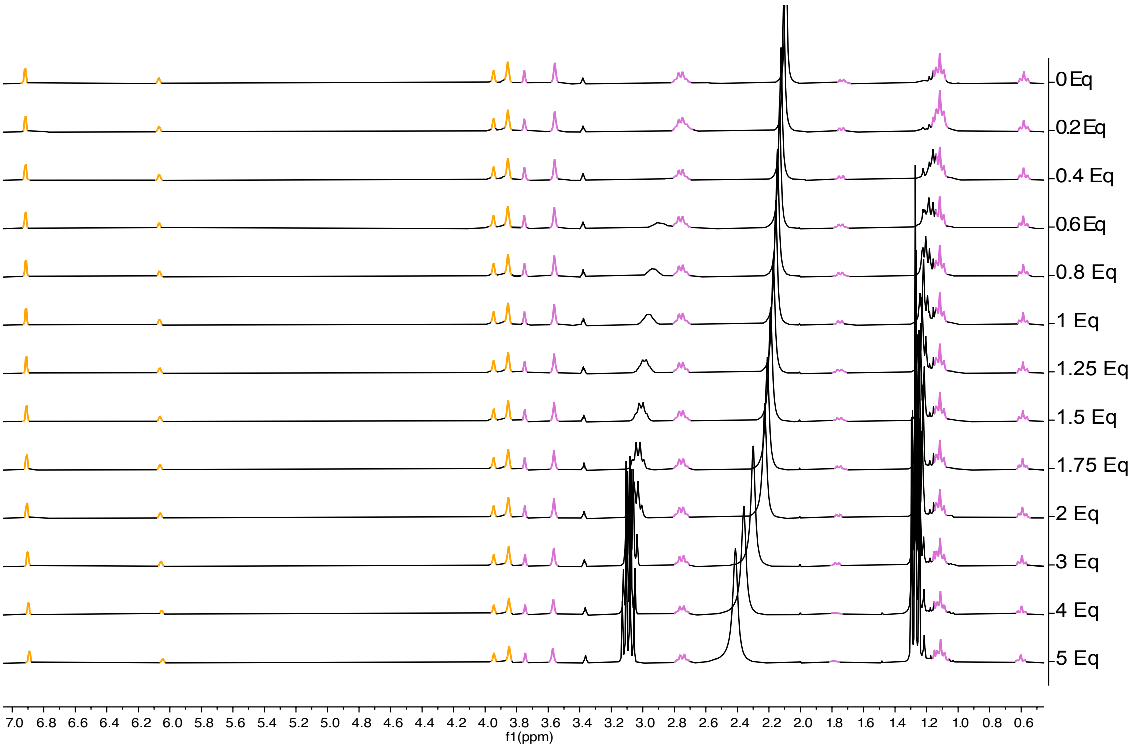

Figure S47: ^{1}H NMR spectral changes observed in **Et^{2}F^2$_{red}$** (c = 2.5 mM) in $CDCl_3/CD_3OD$ (95:5, v/v) with the addition of up to 5 equiv. of TEA$^+$PFOA$^-$.

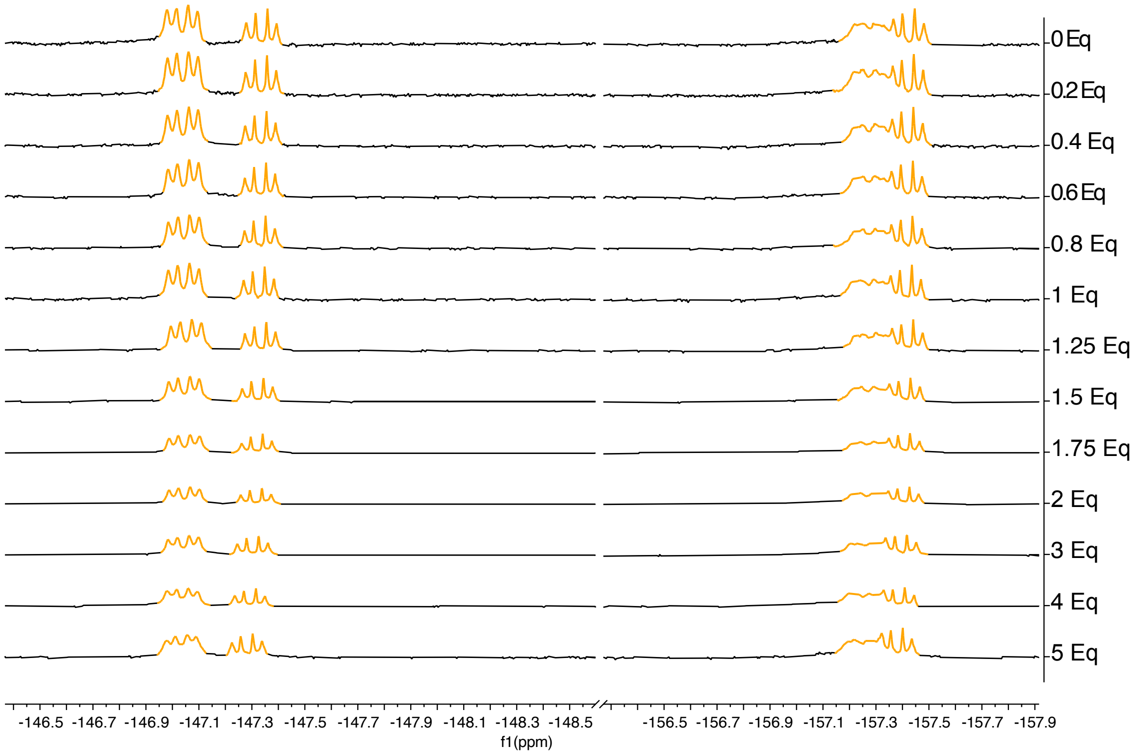

Figure S48: ^{19}F NMR spectral changes observed in **Et^{2}F^2$_{red}$** (c = 2.5 mM) in $CDCl_3/CD_3OD$ (95:5, v/v) with the addition of up to 5 equiv. of TEA^+PFOA^-.

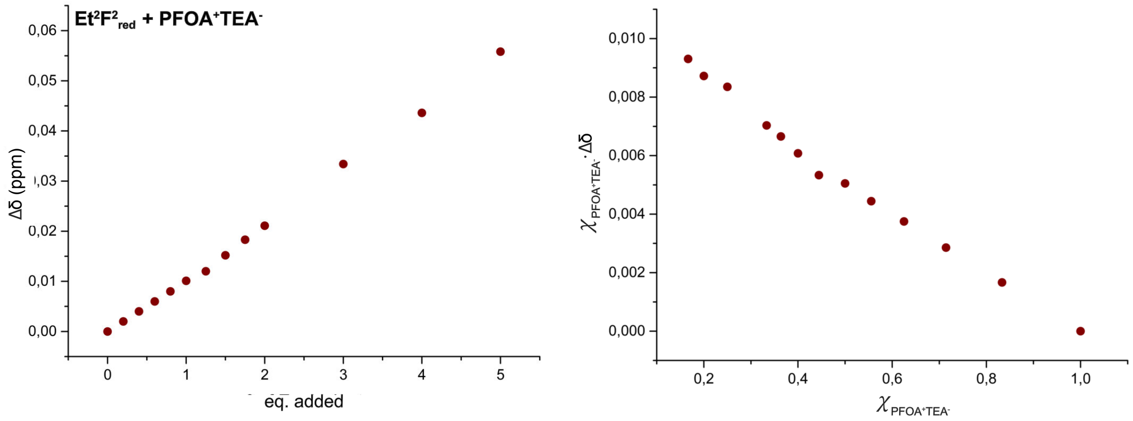

Figure S49: a) Titration profile of **Et^{2}F^2$_{red}$** upon TEA^+PFOA^- addition, following H_A shifting and b) the Job's plot of the titration, showing no maximum at any point.

$Et^2F^2_{red}$ with addition of TFA

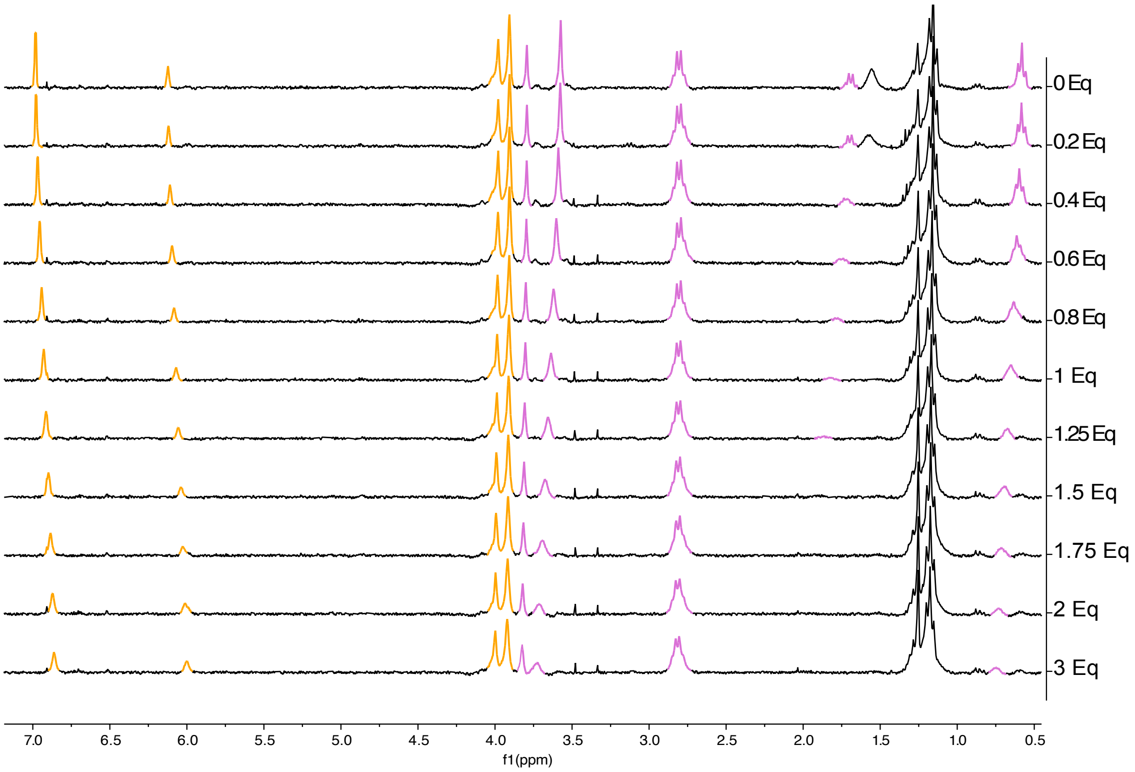

Figure S50: ^{1}H NMR spectral changes observed in $\mathbf{Et^2F^2_{red}}$ (c = 2.5 mM) in $CDCl_3$ with the addition of up to 3 equiv. of TFA.

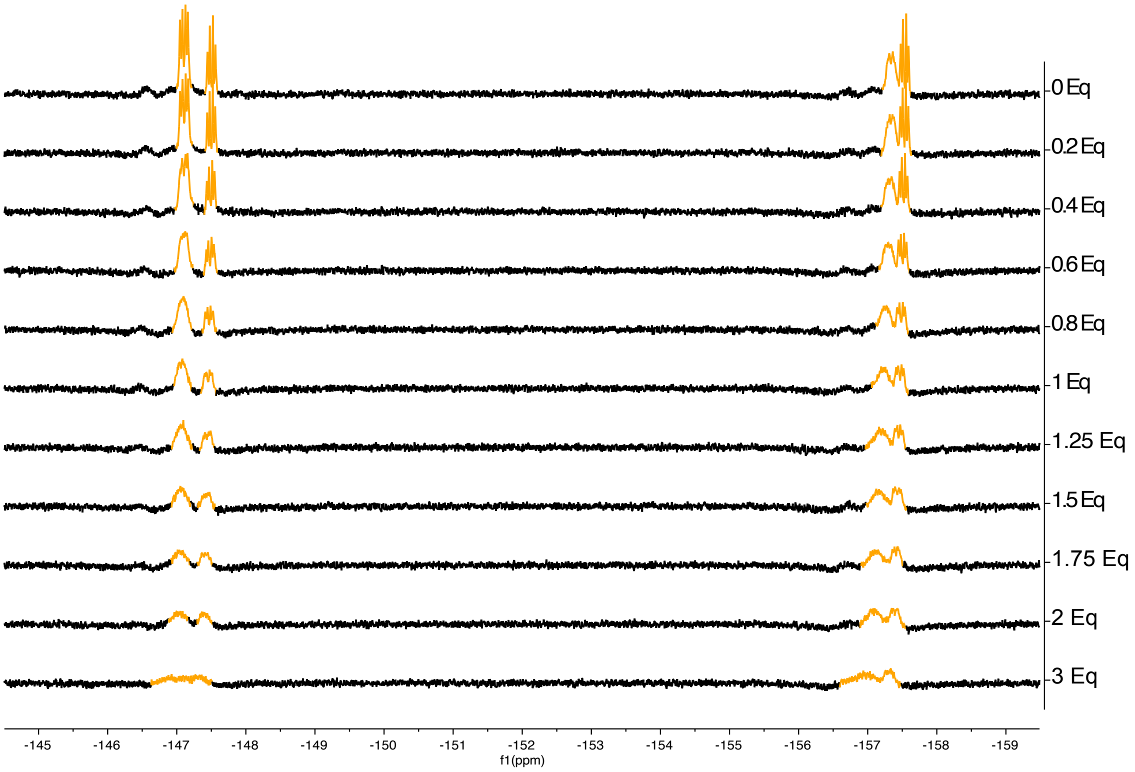

Figure S51: ^{19}F NMR spectral changes observed in $\mathbf{Et^2F^2_{red}}$ (c = 2.5 mM) in $CDCl_3$ during the addition of up to 3 equiv. of TFA.

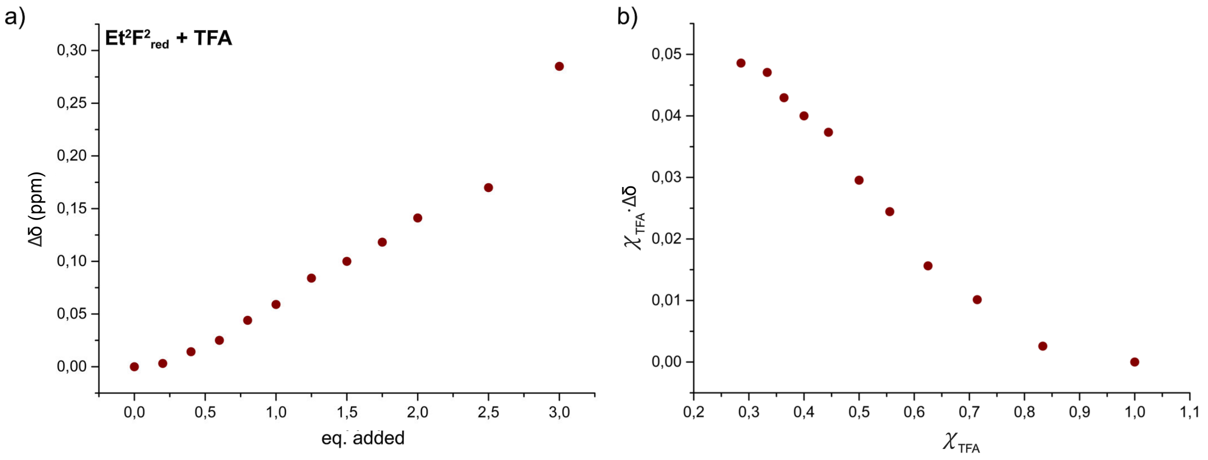

Figure S52: a) Titration profile of **Et^{2}F^2$_{red}$** upon TFA addition, following H_A shifting and b) Job's Plot of the titration, showing no clear maximum.

VIII.b. Titrations of $TREN^2F^2_{red}$

$TREN^2F^2_{red}$ with addition of PFOA

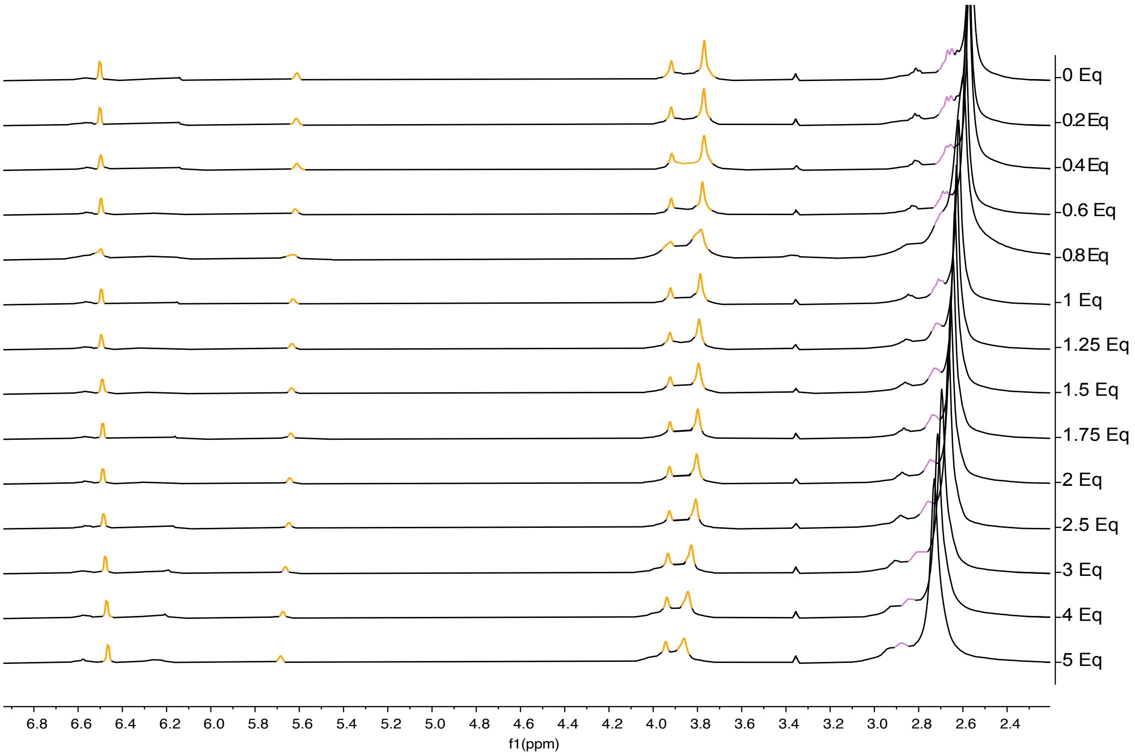

Figure S53: ^{1}H NMR spectral changes observed in $\mathbf{TREN^2F^2_{red}}$ (c = 2.5 mM) in $CDCl_3/CD_3OD$ (95:5, v/v) with the addition of up to 5 equiv. of PFOA.

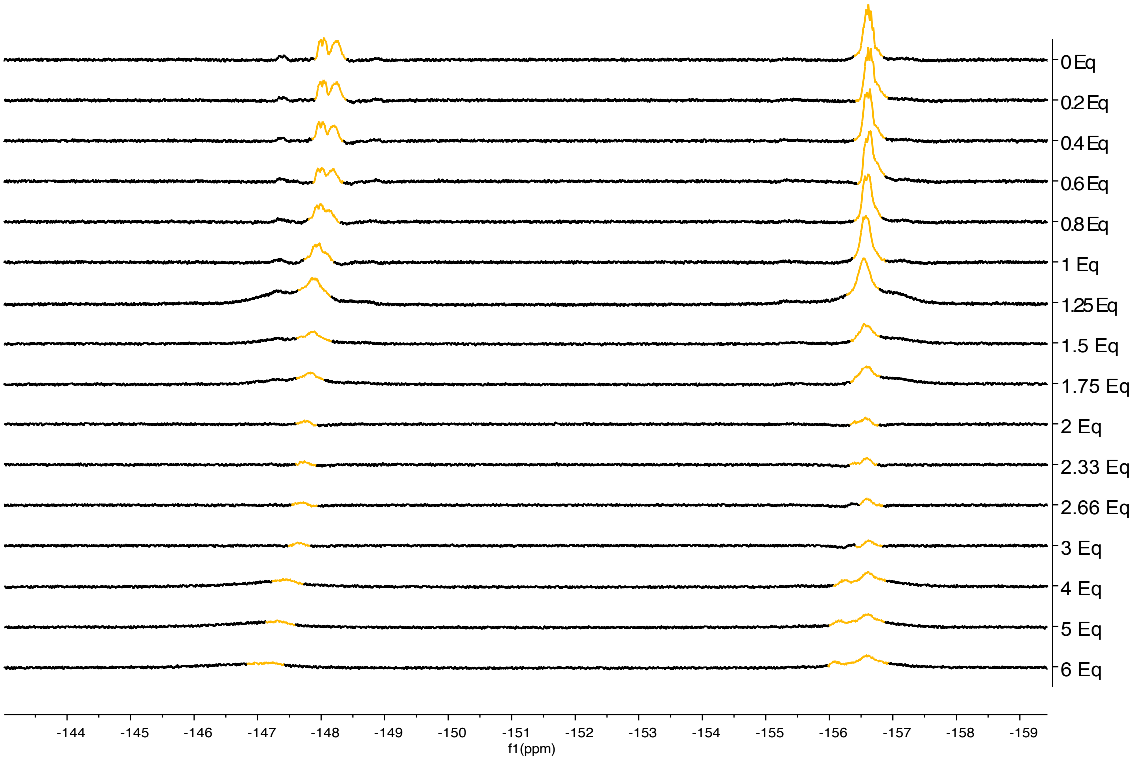

Figure S54: ^{19}F NMR spectral changes observed in $\mathbf{TREN^2F^2_{red}}$ (c = 2.5 mM) in $CDCl_3/CD_3OD$ (95:5, v/v) with the addition of up to 5 equiv. of PFOA.

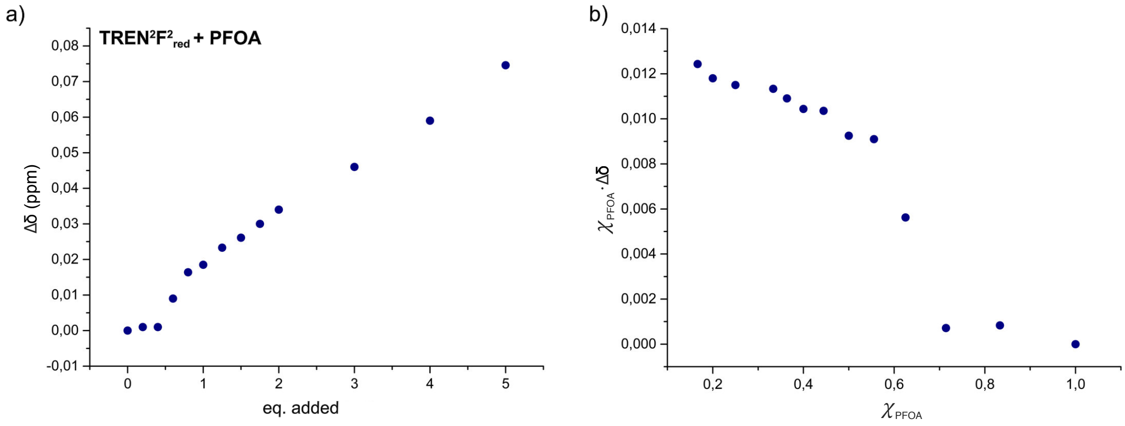

Figure S55: a) Titration profile of **TREN^{2}F^2$_{red}$** upon PFOA addition, following H_A shifting and b) Job's plot of the titration, showing no clear maximum.

PFOA with addition of TREN^{2}F^2$_{red}$

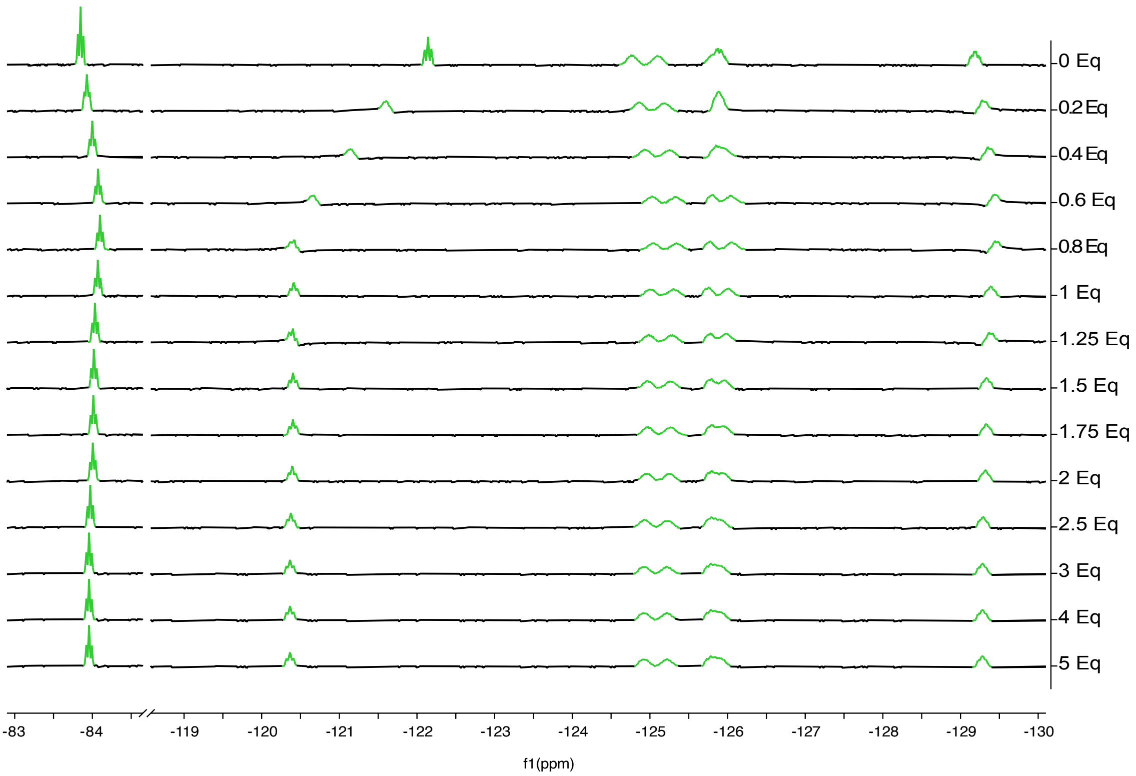

Figure S56: ^{19}F NMR spectral changes observed in PFOA (c = 2.5 mM) in $CDCl_3/CD_3OD$ (95:5, v/v) with the addition of up to 5 equiv. of **TREN^{2}F^2$_{red}$**.

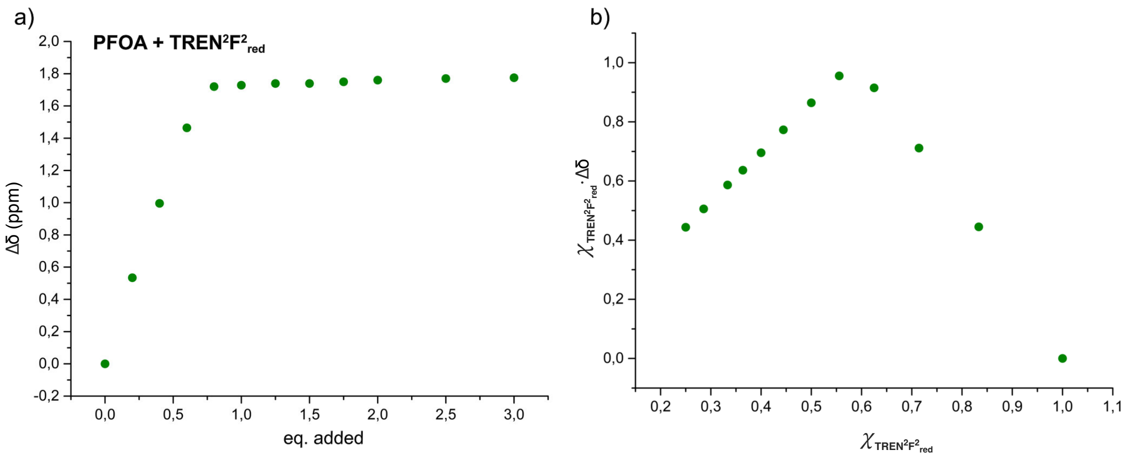

Figure S57: a) Titration profile of PFOA upon **TREN^{2}F$^2_{red}$** addition and b) the Job's plot of the titration, showing a maximum on 0.54, indicating a 1:1 stoichiometry between PFOA and **TREN^{2}F$^2_{red}$**.

TREN^{2}F$^2_{red}$ with addition of TEA$^+$PFOA$^-$

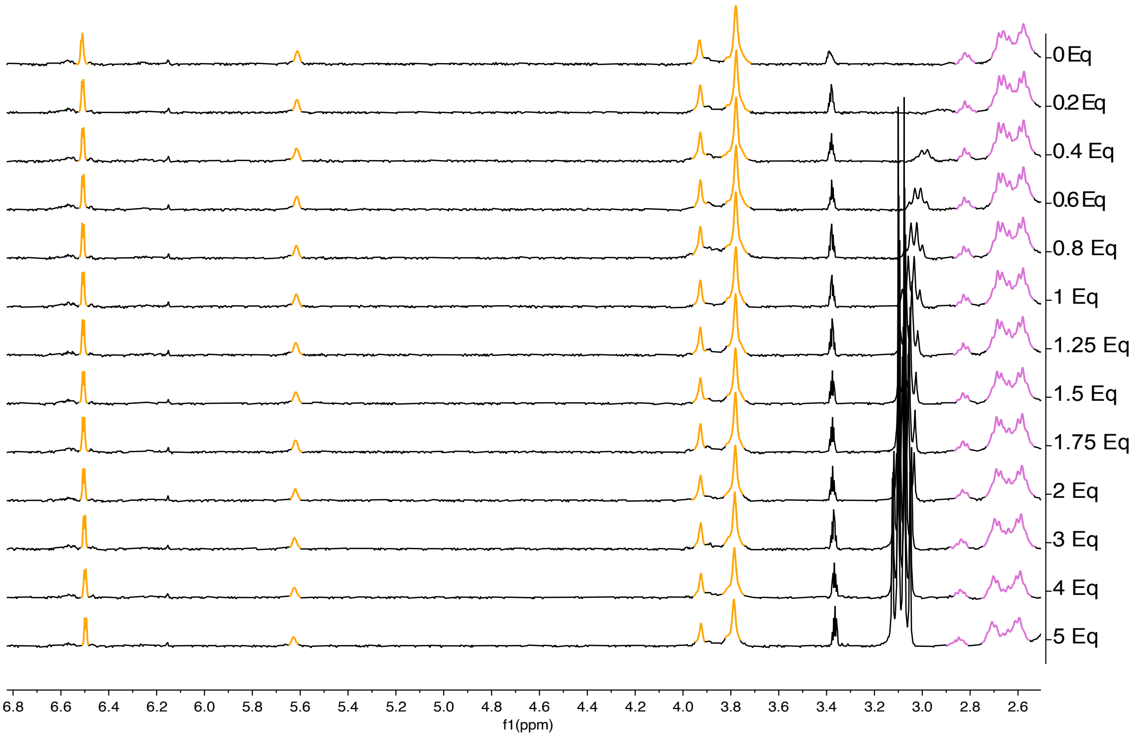

Figure S58: ^{1}H NMR spectral changes observed in **TREN^{2}F$^2_{red}$** (c = 2.5 mM) in $CDCl_3/CD_3OD$ (95:5, v/v) with the addition of up to 5 equiv. of TEA$^+$PFOA$^-$.

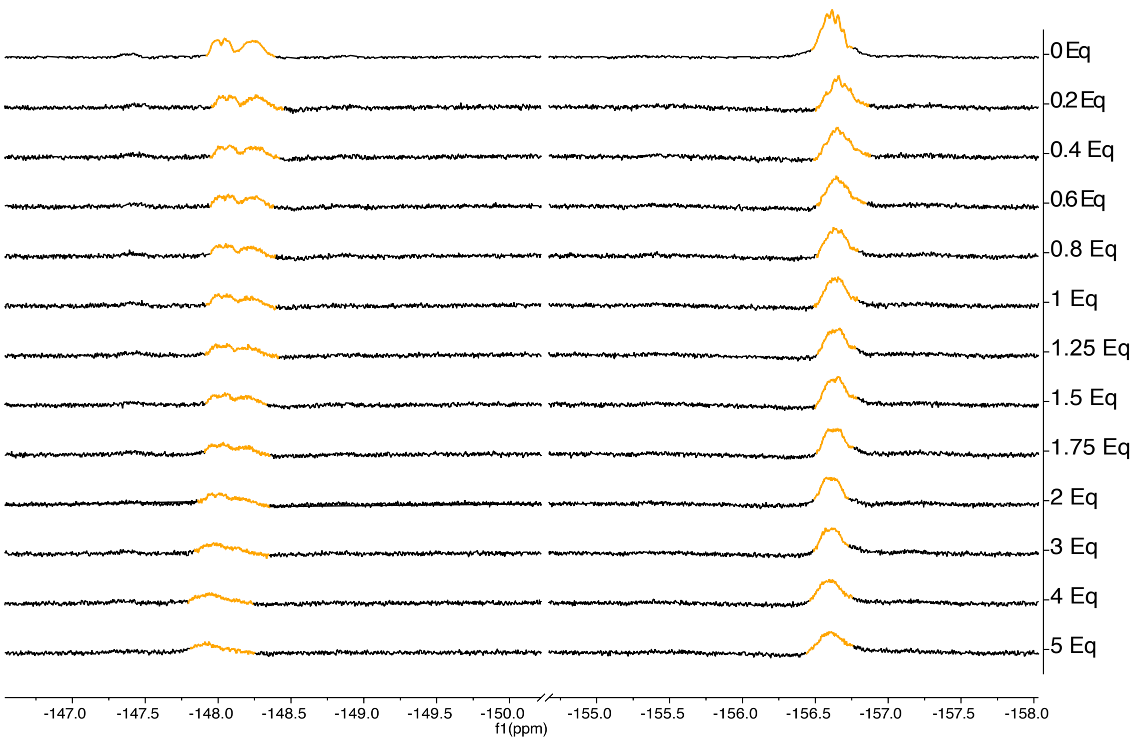

Figure S59: ^{19}F NMR spectral changes observed in **TREN^{2}F^2$_{red}$** (c = 2.5 mM) in $CDCl_3$/CD_3OD (95:5, v/v) with the addition of up to 5 equiv. of TEA^+PFOA^-.

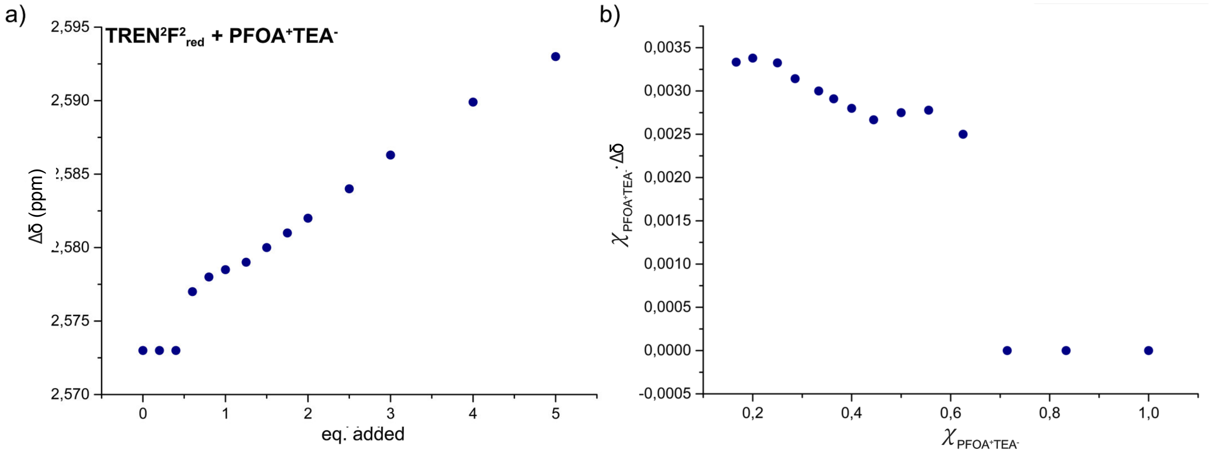

Figure S60: a) Titration profile of **TREN^{2}F^2$_{red}$** upon TEA^+PFOA^- addition, following H_A shifting and b) Job's Plot of the titration, showing a first maximum at 0.55 and a second one at 0.2. Indicating a different interaction between substances.

TREN^{2}F$^2_{red}$ with addition of TFA

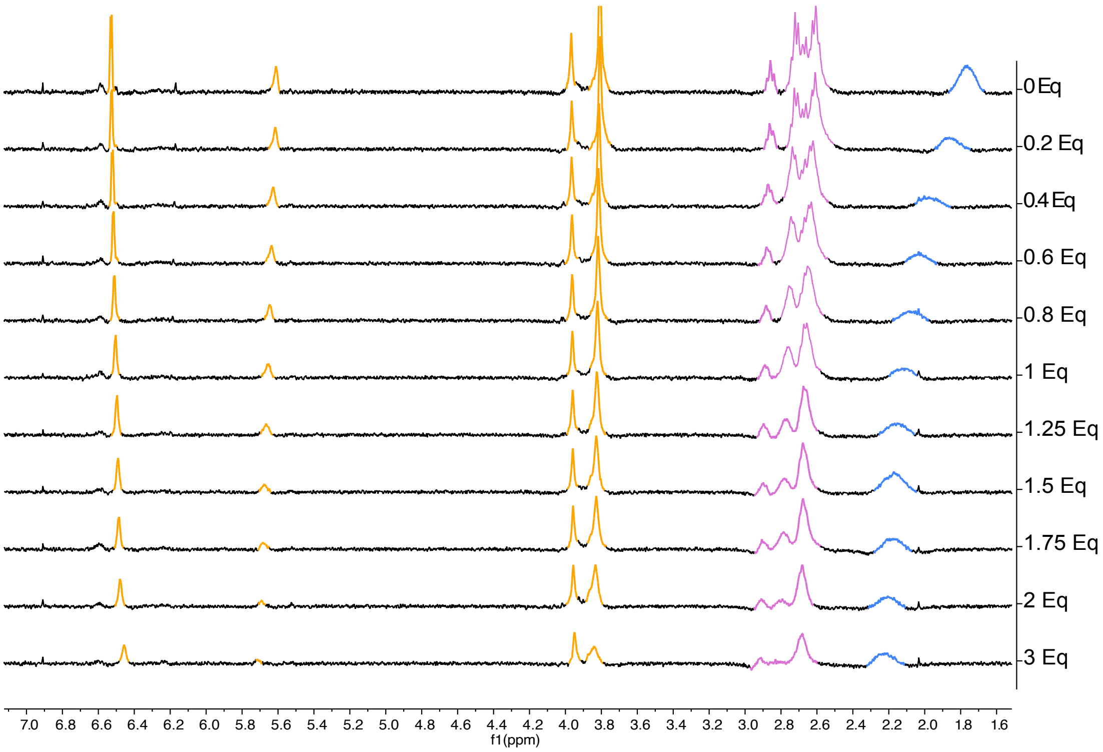

Figure S61: ^{1}H NMR spectral changes observed in **TREN^{2}F$^2_{red}$** (c = 2.5 mM) in $CDCl_3$ during the addition of up to 2 equiv. of TFA.

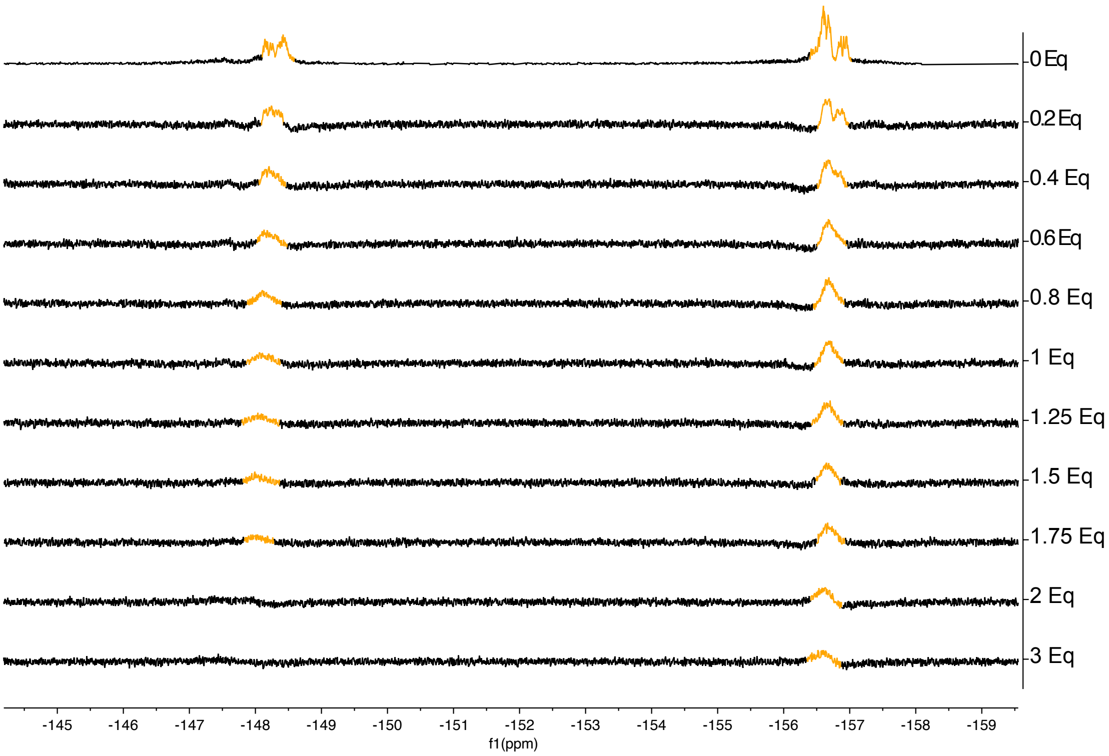

Figure S62: ^{19}F NMR spectral changes observed in **TREN^{2}F$^2_{red}$** (c = 2.5 mM) in $CDCl_3$ with the addition of up to 3 equiv. of TFA.

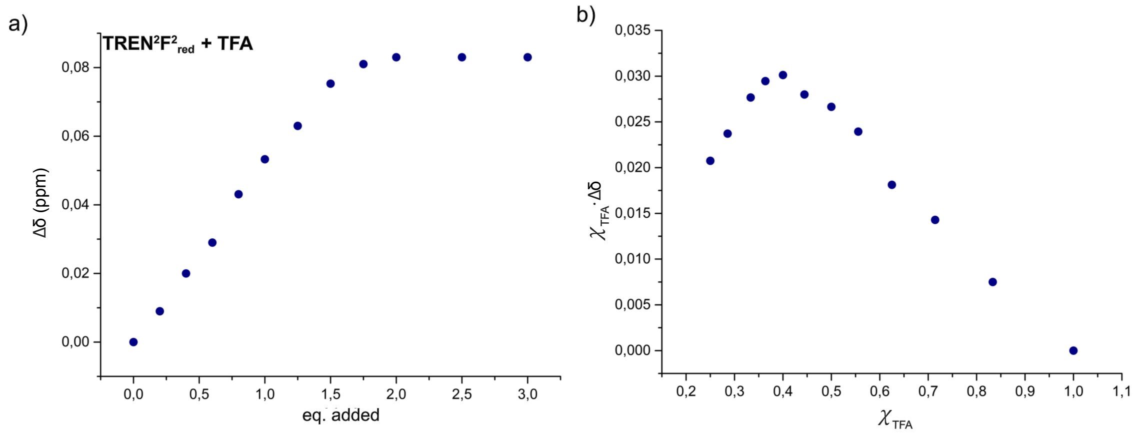

Figure S63: a) Titration profile of $\mathbf{TREN^2F^2_{red}}$ upon TFA addition, following H_A shifting and b) Job's Plot of the titration, showing a maximum at 0.33, indicating a 2:1 stoichiometry between $\mathbf{TREN^2F^2_{red}}$ and TFA.

VIII.c. Titrations of $Bn^3F^1_{red}$

$Bn^3F^1_{red}$ with addition of PFOA

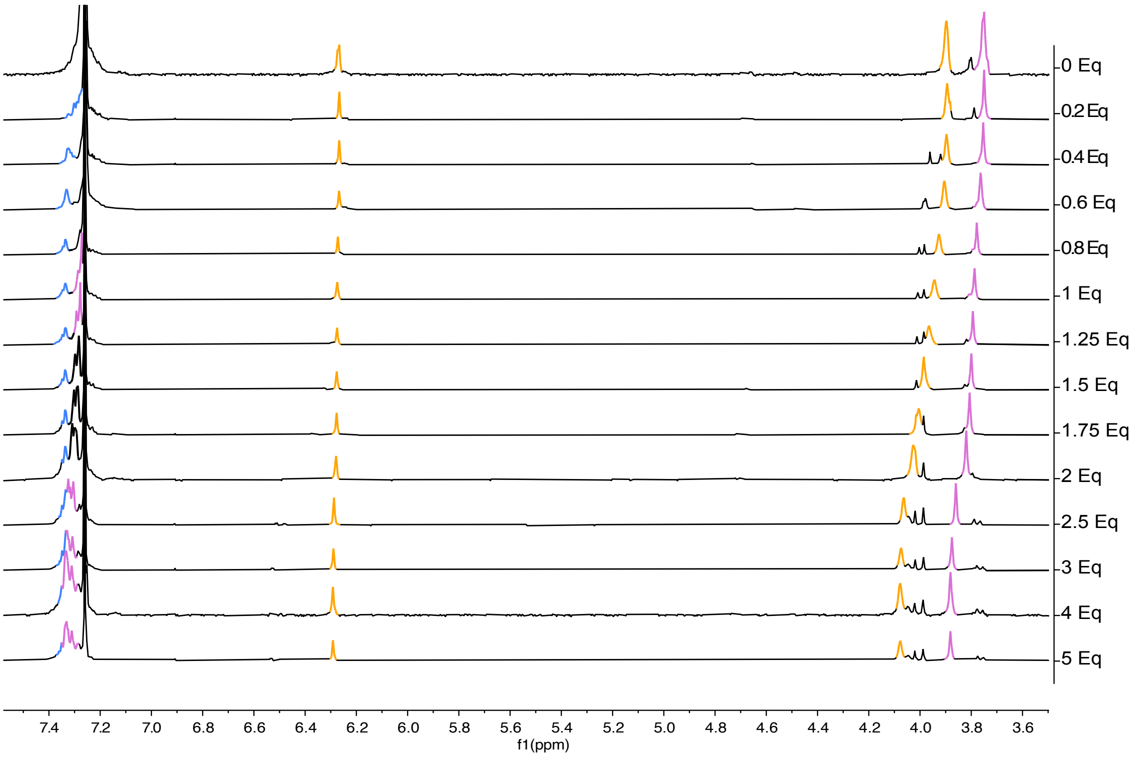

Figure S64: ^{1}H NMR spectral changes observed in $\mathbf{Bn^3F^1_{red}}$ (c = 2.5 mM) in $CDCl_3/CD_3OD$ (95:5, v/v) with the addition of up to 5 equiv. of PFOA.

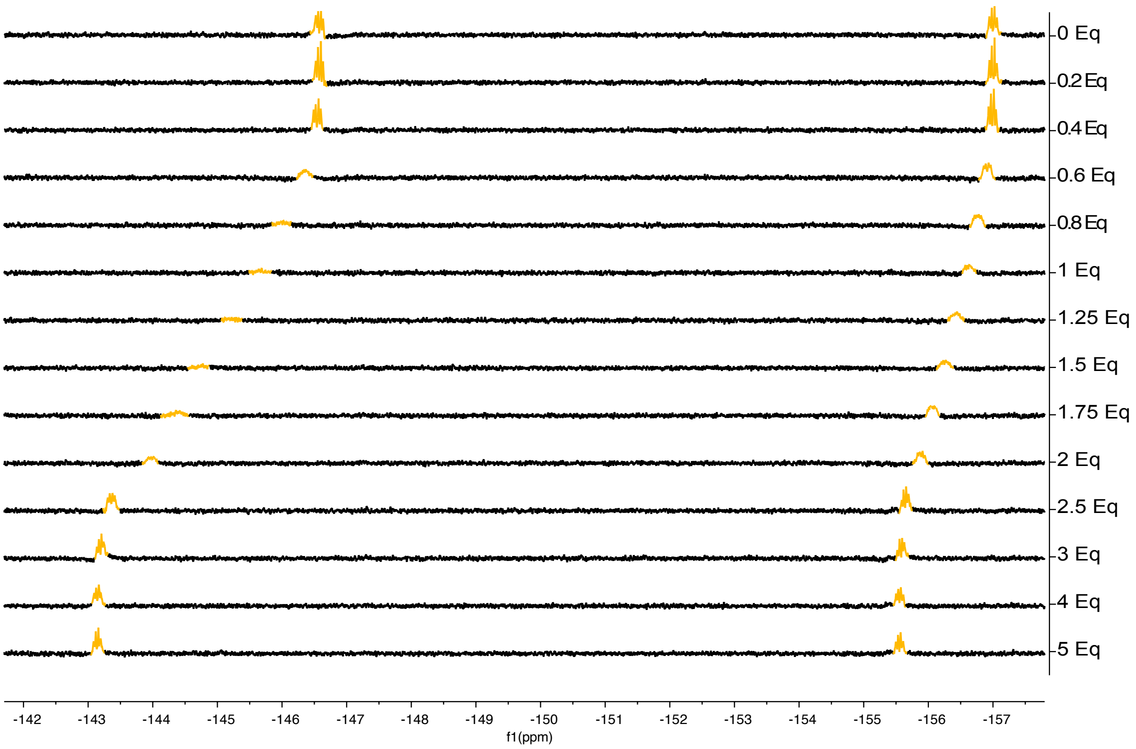

Figure S65: ^{19}F NMR spectral changes observed in $\mathbf{Bn^3F^1_{red}}$ (c = 2.5 mM) in $CDCl_3/CD_3OD$ (95:5, v/v) with the addition of up to 5 equiv. of PFOA.

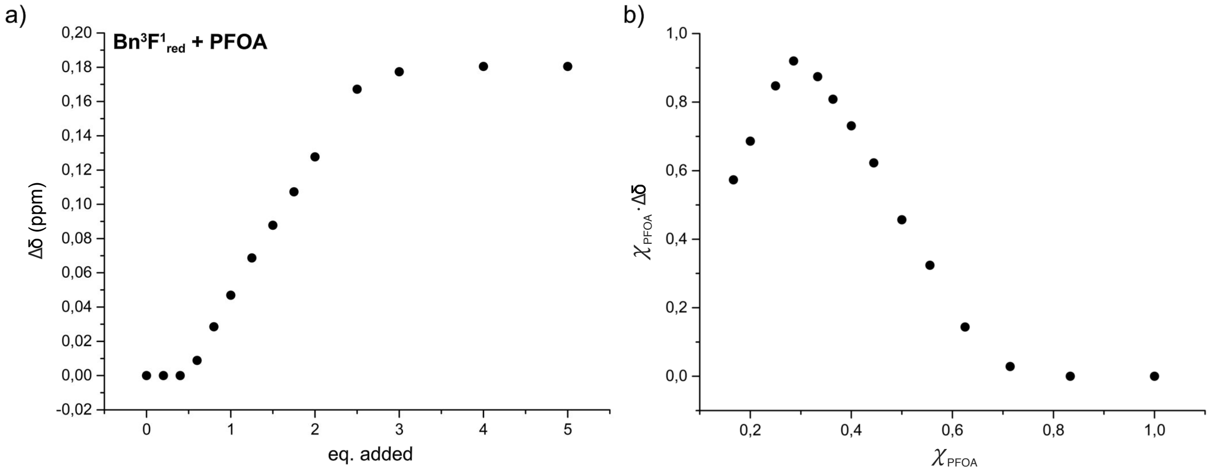

Figure S66: a) Titration profile of $\mathbf{Bn^3F^1_{red}}$ upon PFOA addition, following H_A shifting and b) the Job's plot of the titration, showing a maximum at 0.33, indicating a 2:1 stoichiometry between $\mathbf{Bn^3F^1_{red}}$ and PFOA.

PFOA with addition of $\mathbf{Bn^3F^1_{red}}$

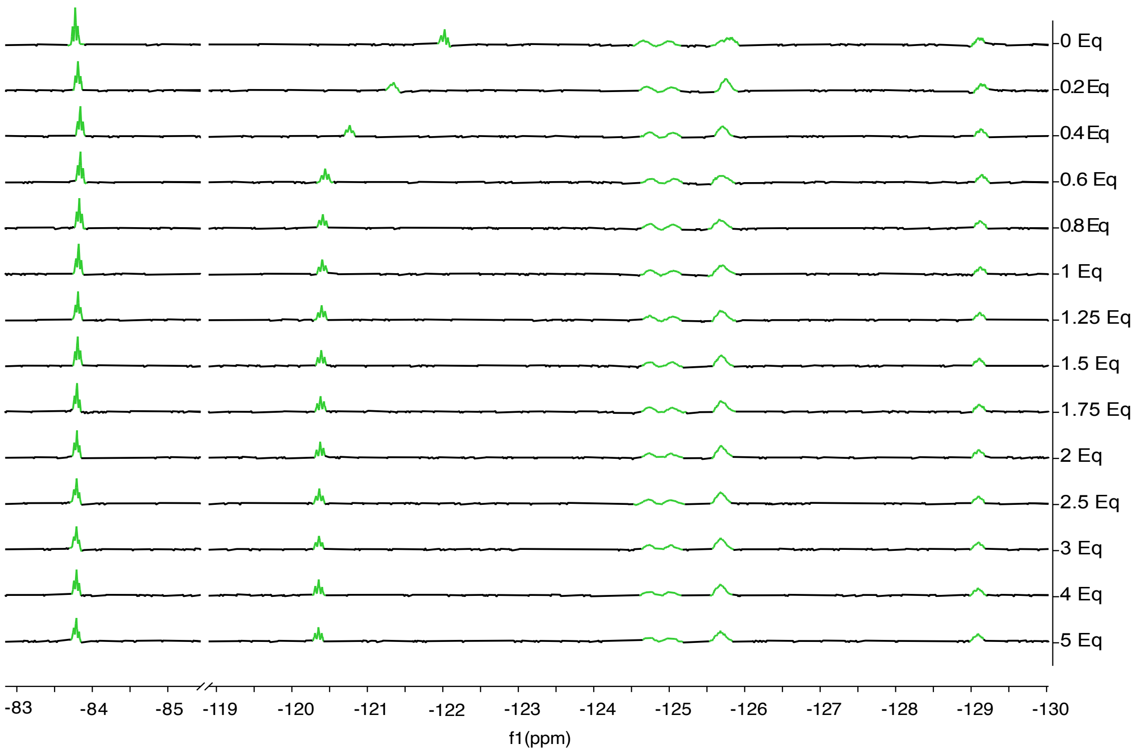

Figure S67: ^{19}F NMR spectral changes observed in PFOA (c = 2.5 mM) in $CDCl_3/CD_3OD$ (95:5, v/v) with the addition of up to 5 equiv. of $\mathbf{Bn^3F^1_{red}}$.

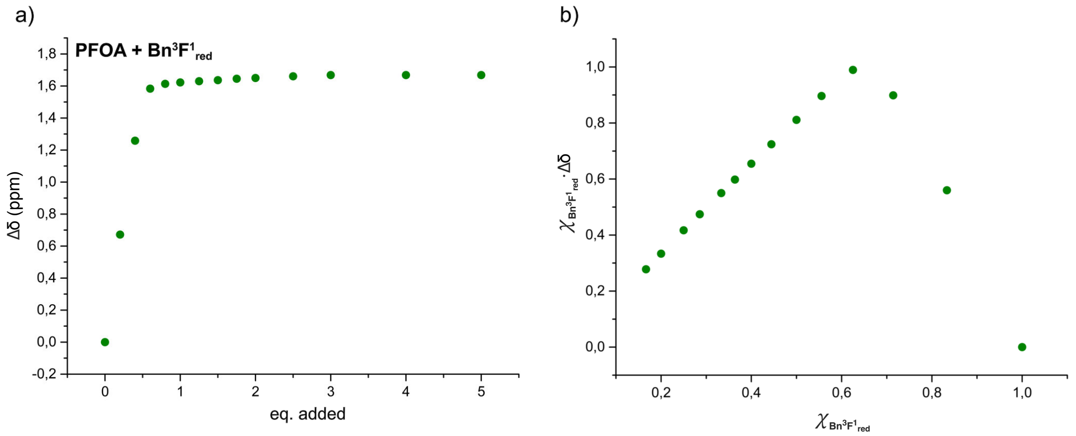

Figure S68: a) Titration profile of PFOA upon $\mathbf{Bn^3F^1_{red}}$ addition and b) the Job's Plot of the titration, showing a maximum at 0.66, indicating a 1:2 stoichiometry between PFOA and $\mathbf{Bn^3F^1_{red}}$.

$\mathbf{Bn^3F^1_{red}}$ with addition of $\mathbf{TEA^+PFOA^-}$

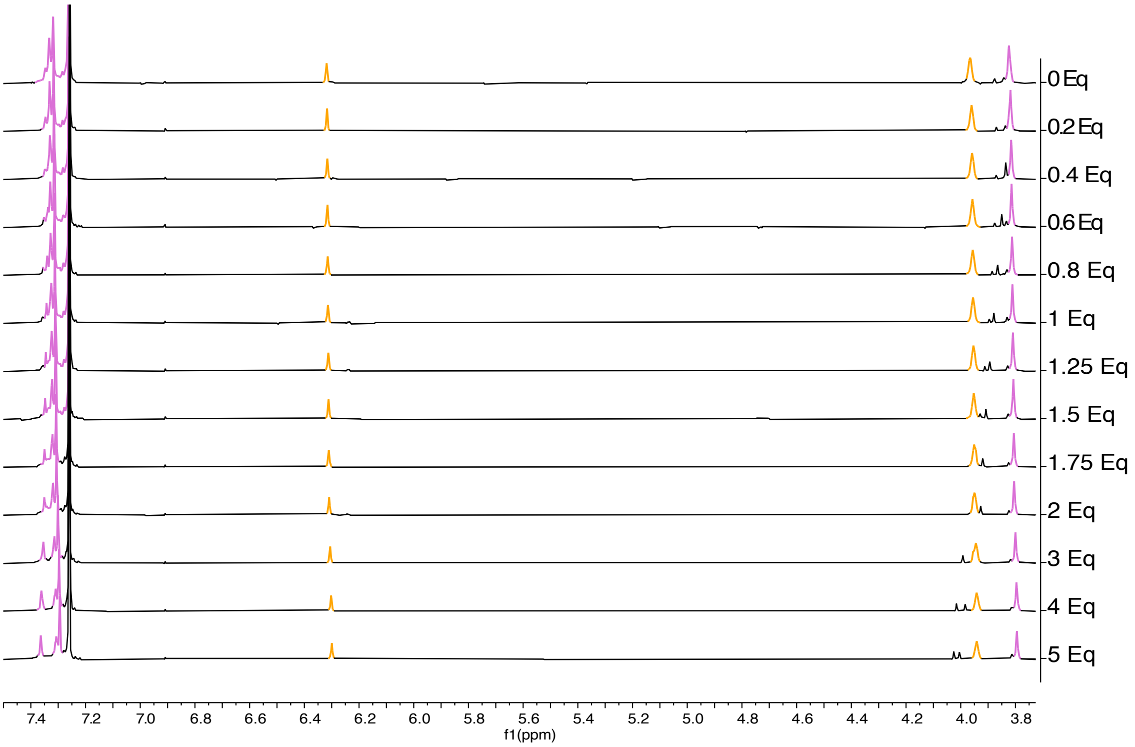

Figure S69: ^{1}H NMR spectral changes observed in $\mathbf{Bn^3F^1_{red}}$ (c = 2.5 mM) in $CDCl_3/CD_3OD$ (95:5, v/v) with the addition of up to 5 equiv. of TEA^+PFOA^-.

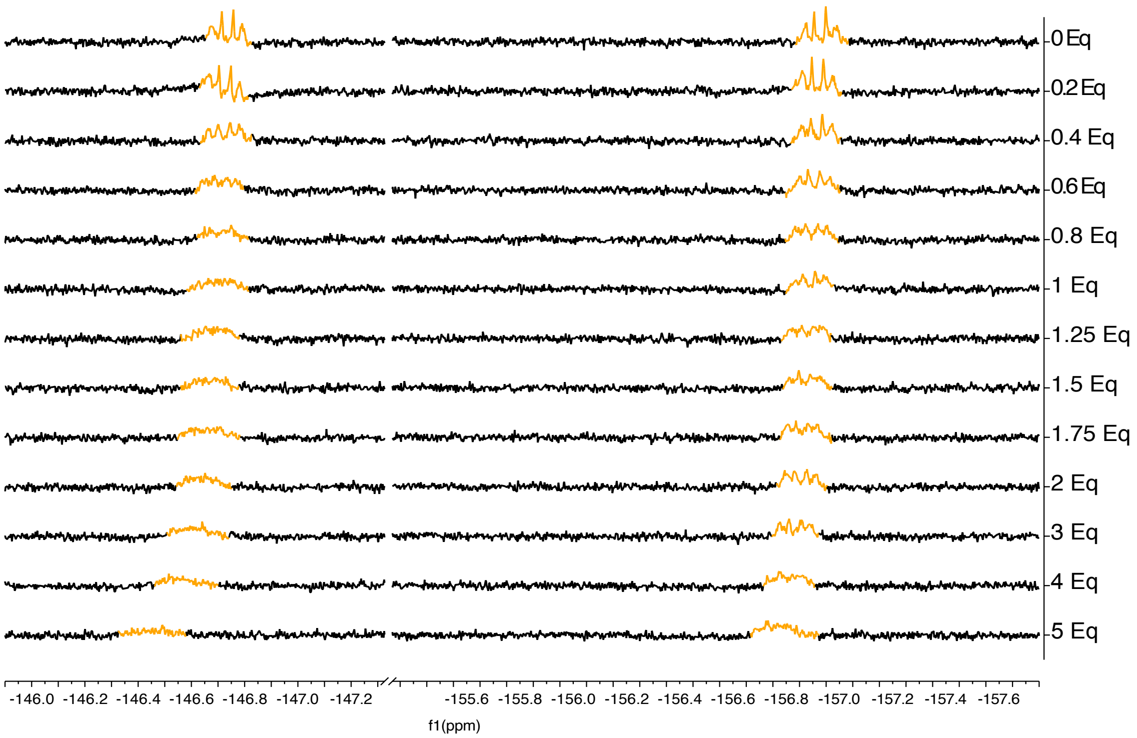

Figure S70: ^{19}F NMR spectral changes observed in **Bn^{3}F^{1}$_{red}$** (c = 2.5 mM) in $CDCl_3/CD_3OD$ (95:5, v/v) with the addition of up to 5 equiv. of $TEA^{+}PFOA^{-}$.

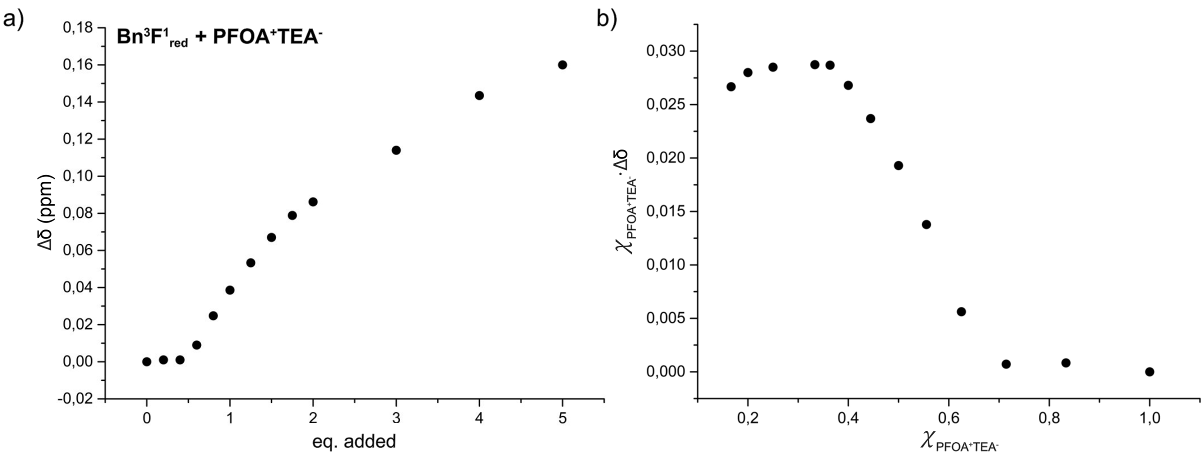

Figure S71: a) Titration profile of **Bn^{3}F^{1}$_{red}$** upon PFOA-TEA salt addition, following H_A shifting and b) Job's Plot of the titration, showing a maximum at 0.33, indicating a 2:1 stoichiometry of **Bn^{3}F^{1}$_{red}$** and $TEA^{+}PFOA^{-}$.

$Bn^3F^1_{red}$ with addition of TFA

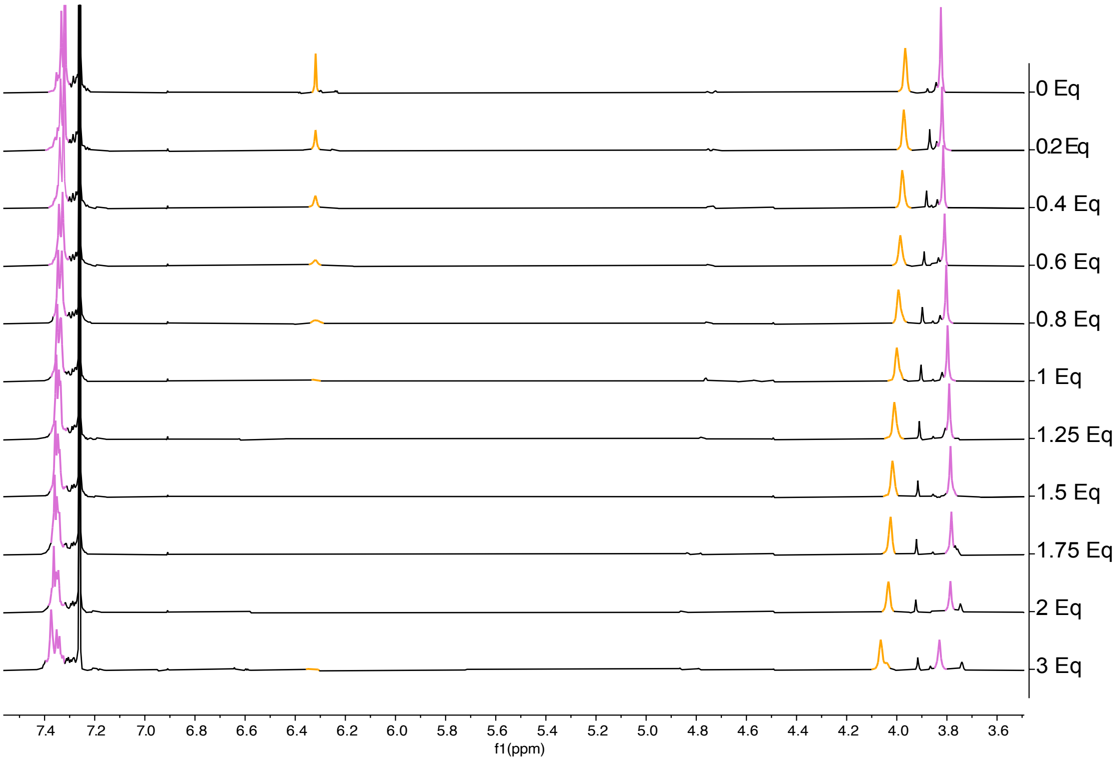

Figure S72: ^{1}H NMR spectral changes observed in **$Bn^3F^1_{red}$** (c = 2.5 mM) in $CDCl_3$ with the addition of up to 5 equiv. of TFA.

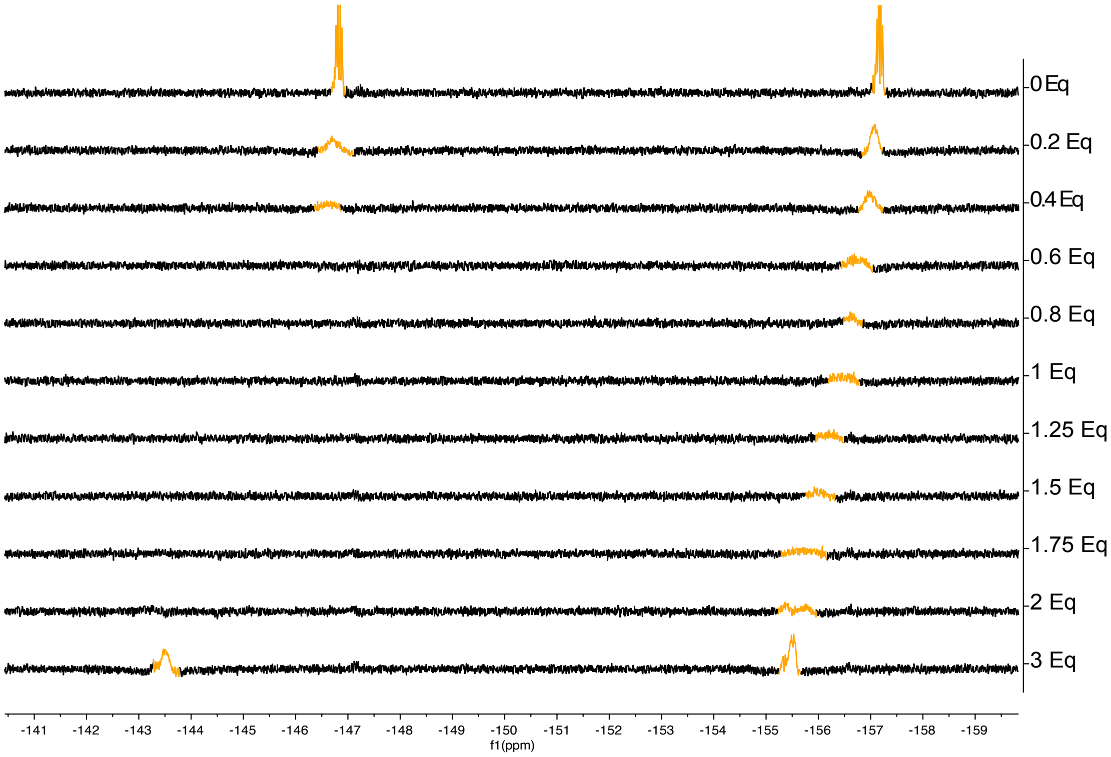

Figure S73: ^{19}F NMR spectral changes observed in **$Bn^3F^1_{red}$** (c = 2.5 mM) in $CDCl_3$ with the addition of up to 3 equiv. of TFA.

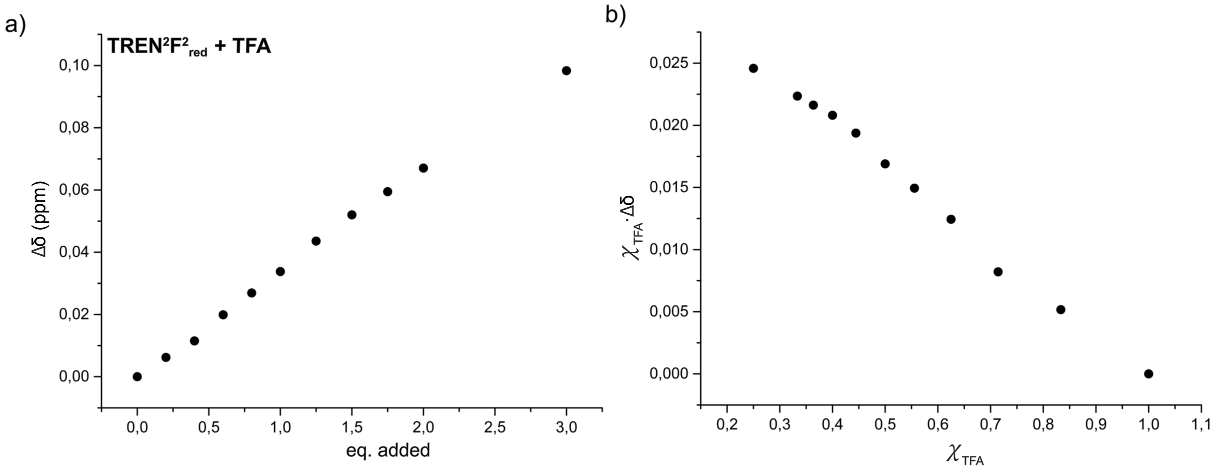

Figure S74: a) Titration profile of $\mathbf{Bn^3F^1_{red}}$ upon TFA addition, following H_A shifting and b) Job's Plot of the titration, showing no maximum at any point.

VIII.d. 2D-NMR studies of $Et^2F^2_{red}$ with PFOA

All samples were measured at a 10 mM concentration in a mixture of $CDCl_3/CD_3OD$ 95:5 (v/v). First, each $\mathbf{Et^2F^2_{red}}$ was measured individually. After completing these measurements, 2 equivalents PFOA were added while maintaining a constant host concentration. The correlation spectra were then remeasured. Solutions were prepared fresh before each measurement. The ^{19}F correlation spectra were measured on a Bruker Avance 400 (^1H NMR: 400 MHz, $^{19}F\{^1H\}$ NMR: 376 MHz at 298 K), and the ^1H-^1H NOESY experiments were performed on a Bruker Avance NEO 600 spectrometer (^1H NMR: 600 MHz).

Short summary of the results:

When ^1H-^{19}F HOESY experiment was performed on $\mathbf{Et^2F^2_{red}}$, the only observable cross peaks corresponded the contacts between the Ar_F–CH_2–N bridge. No additional signal was distinguishable between the S/N ratio.

After the addition of 2 equivalents of PFOA, the same NMR experiments were repeated. The ^1H-^{19}F HOESY spectrum showed no observable correlations, and the ^{19}F-^{19}F NOESY spectrum revealed only intramolecular contacts between adjacent fluorine atoms along the perfluorinated chain of PFOA. This absence of detectable intermolecular interactions may also result from the pronounced broadening of the ^1H and ^{19}F signals of $\mathbf{Et^2F^2_{red}}$, which hampers the observation of cross peaks. Due to restrictions in the solubility of the compounds and compound mixtures, higher concentrations could not be achieved.

In contrast, the ^1H-^1H NOESY spectrum displayed the emergence of new cross peaks upon PFOA addition. Notably, the previously isolated and upfield-shifted ethyl group in the pure cage sample now exhibited a correlation pattern akin to the other ethyl groups. This strongly suggests that the cage undergoes a structural rearrangement upon PFOA binding, effectively "freeing" the constrained ethyl group and rendering its environment more comparable to the rest.

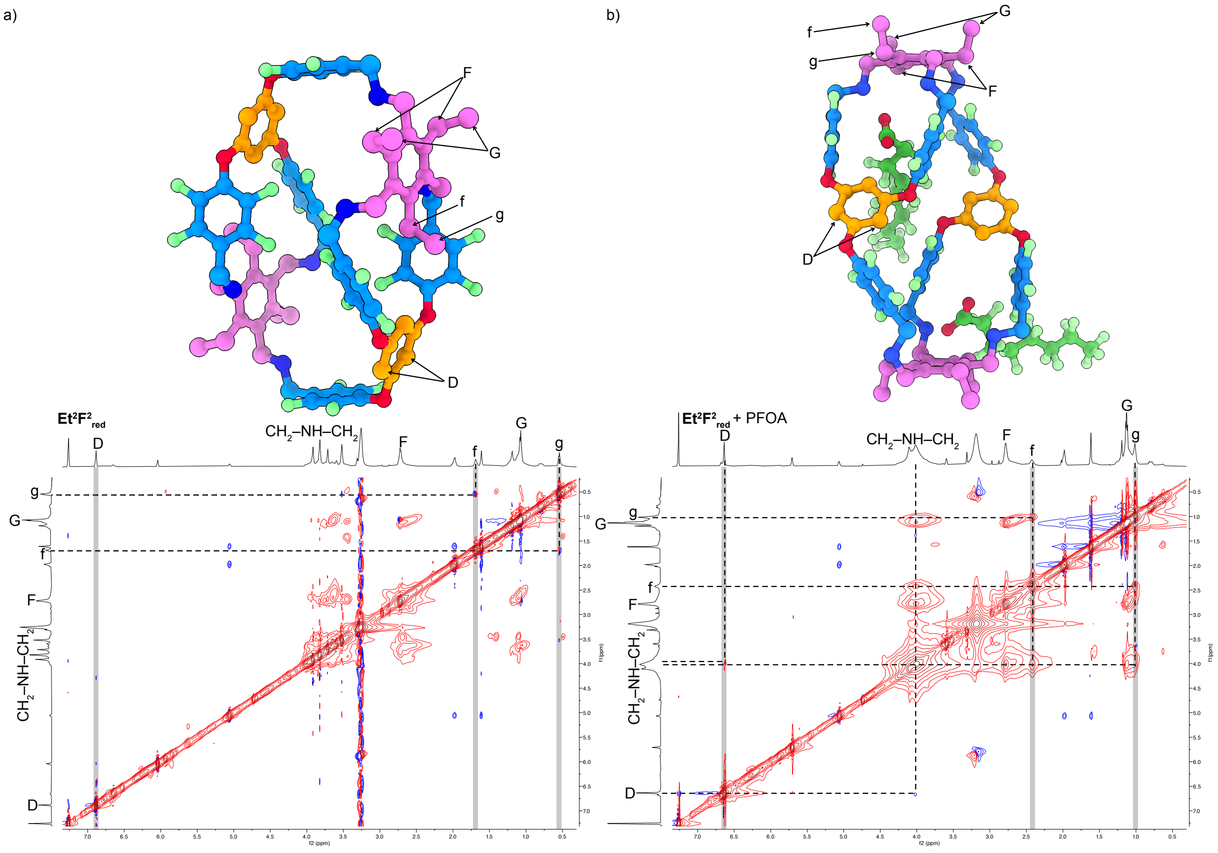

Figure S75: ^{1}H-^{1}H NOESY (600 MHz, 298 K) spectra of **$Et^2F^2_{red}$** a) before and b) after addition of 2 equivalents of PFOA. The corresponding solid-state structures obtained by SC-XRD are shown alongside for comparison, illustrating a possible structural rearrangement that accounts for the observed chemical shift changes and the appearance of new cross peaks upon PFOA addition. The black dotted lines indicate the observed cross peaks, while the grey bars only act as visual aids.

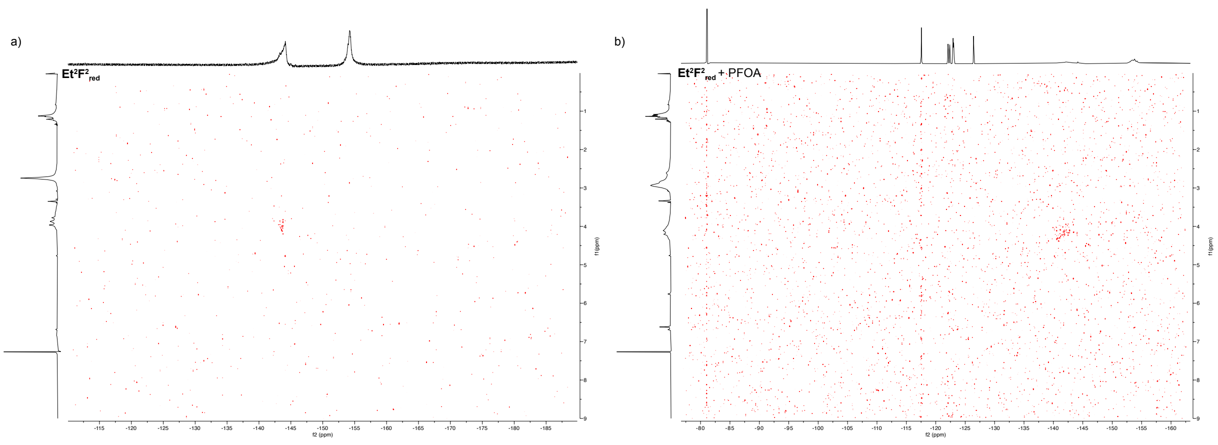

Figure S76: ^{19}F-^{1}H HOESY (400 MHz, 376 MHz, 298 K) spectra of **$Et^2F^2_{red}$** a) before and b) after addition of 2 equivalents of PFOA.

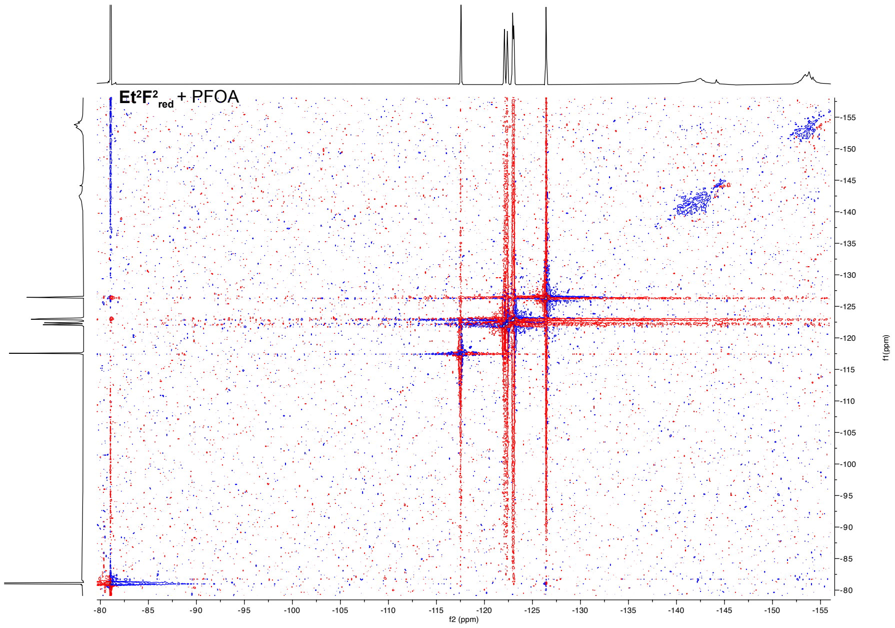

Figure S77: ^{19}F-^{19}F NOESY (376 MHz, 298 K) spectrum of **$Et^2F^2_{red}$** after addition of 2 equivalents of PFOA.

IX. PFOA removal

General procedure A:

In separate 2 mL vials, either 2.9 mg (1.0 eq.), 8.7 mg (3.0 eq.), or 14.5 mg (5 eq.) of **Et^{2}F^2$_{red}$**, or 2.5 (1.0 eq.), 7.6 (3.0 eq.), or 12.7 mg (5.0 eq.) of **TREN^{2}F^2$_{red}$** was added. To each vial, 0.7 mL of a 1.0 mg mL^{-1} solution of PFOA in deionised water was added. The resulting colourless suspensions were stirred for 1 hour at room temperature and then filtered using a syringe filter, yielding a clear solution in all cases. The obtained solutions were analysed by ^{19}F NMR using an external hexafluorobenzene standard for reference. The amount of PFOA removed was calculated by comparison of the integrals.

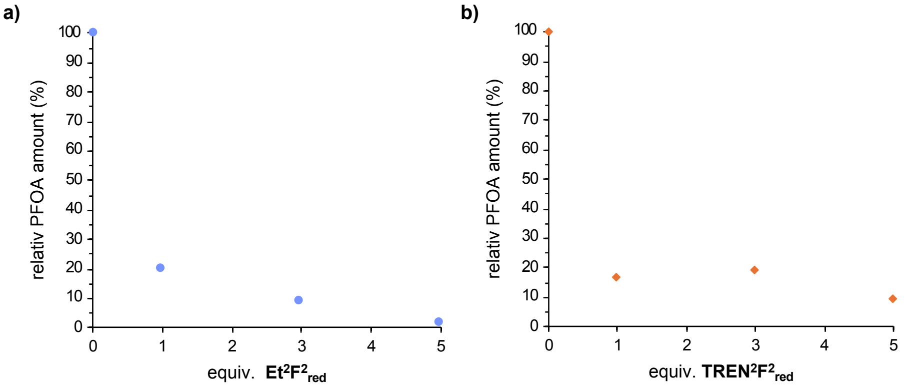

Figure S78: Plots of the relative amounts of PFOA left in the aqueous phase upon the addition of different equivalents of either a) **Et^{2}F^2$_{red}$** or b) **TREN^{2}F^2$_{red}$**.

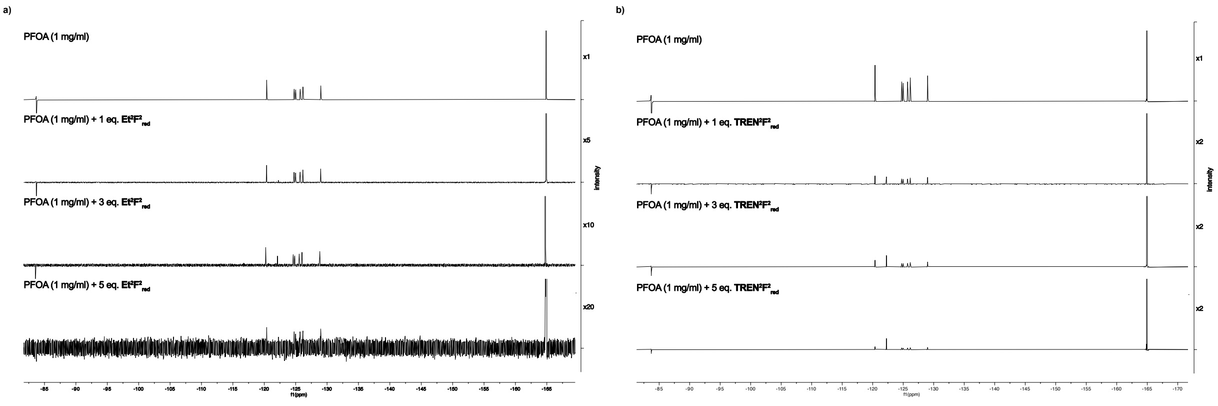

Figure S79: Stacked ^{19}F NMR (565 MHz, 128 scans) spectra of the PFOA removal experiments using the same amount of a) **Et^{2}F^2$_{red}$** or b) **TREN^{2}F^2$_{red}$**.

General procedure B:

In separate 2 mL vials, either 2.9 mg **Et^{2}F2$_{red}$** or 2.5 mg **TREN^{2}F$^2_{red}$** was placed. To each vial, a solution of PFOA in deionised water at varying concentrations (2 mg mL^{-1}, 1 mg mL^{-1}, 0.5 mg mL^{-1}, 0.25 mg mL^{-1}) was added. The resulting colourless suspensions were stirred for 1 hour at room temperature and then filtered using a syringe filter, yielding a clear solution in all cases. The obtained solutions were analysed by ^{19}F NMR using an external hexafluorobenzene standard for reference. The amount of PFOA removed was calculated by comparison of the integrals.

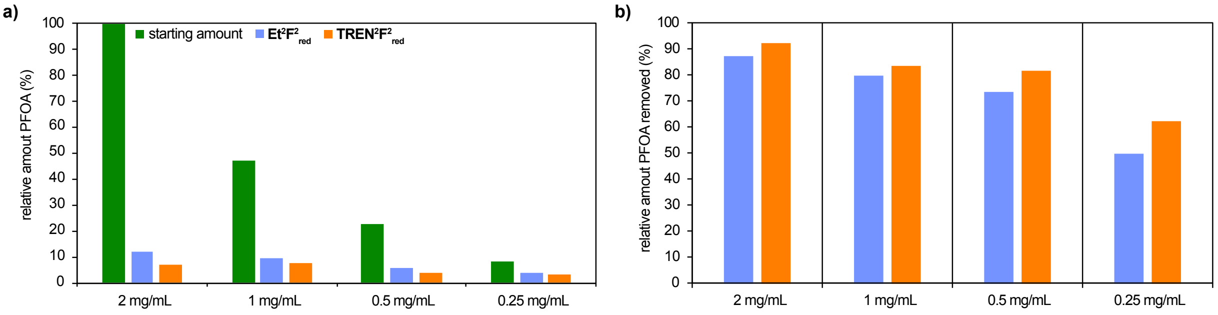

Figure S80: a) The relative amount of PFOA in the aqueous phase standardised to the 2 mg mL^{-1} starting solution, and b) the relative amount of PFOA removed from each solution of differing concentration, using the same amount of **Et^{2}F$^2_{red}$** (2.9 mg) and **TREN^{2}F$^2_{red}$** (2.5 mg) in each case, respectively.

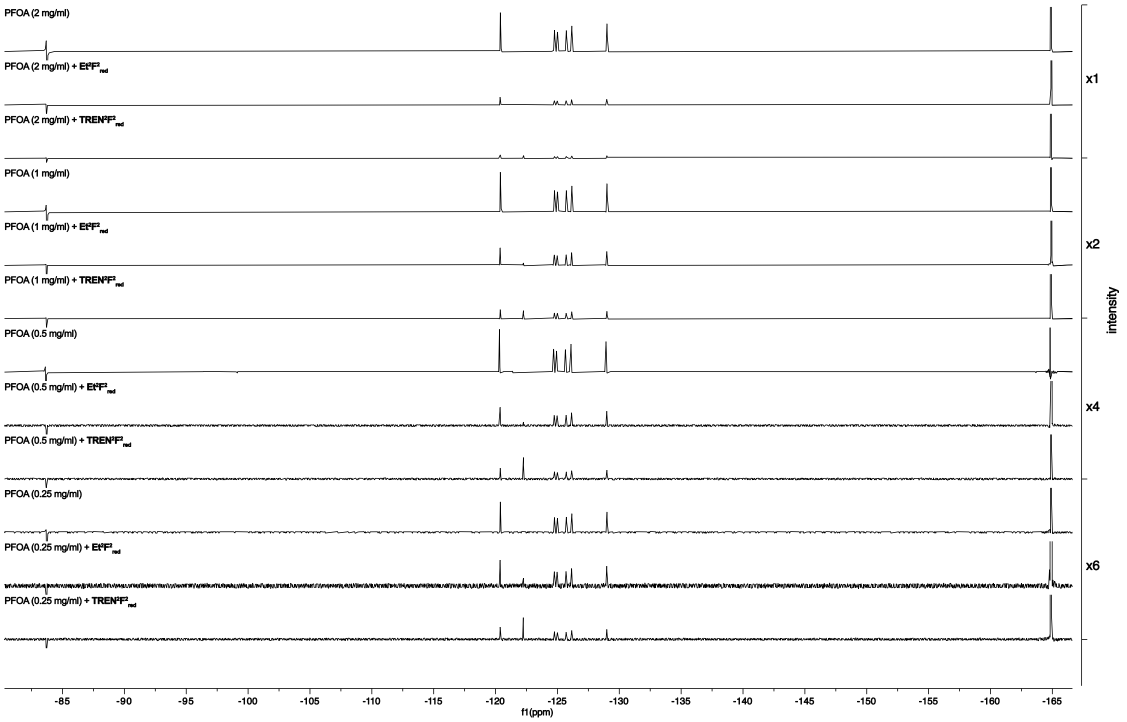

Figure S81: Stacked 19F NMR (565 MHz, 128 scans) spectra of the PFOA removal experiments using the same amount of the respective fluorinated cage.

X. Spectra

Aldehyde F

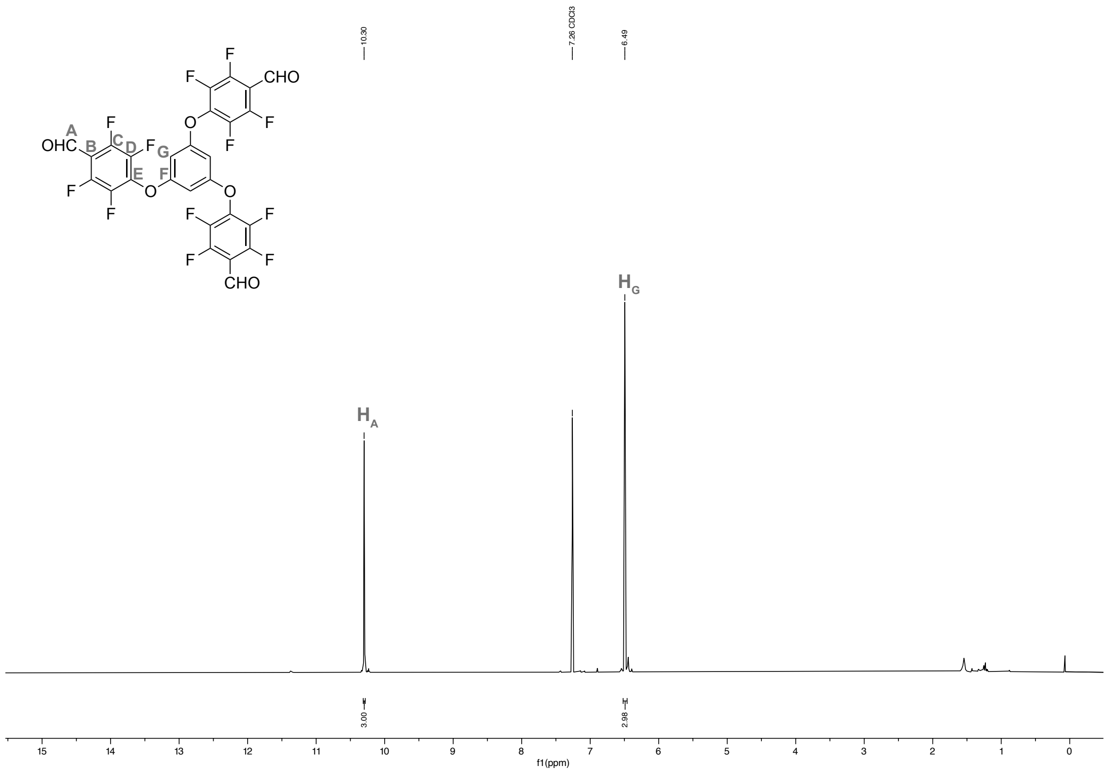

Figure S82: ^{1}H NMR (600 MHz, $CDCl_3$) of fluorinated aldehyde **F**.

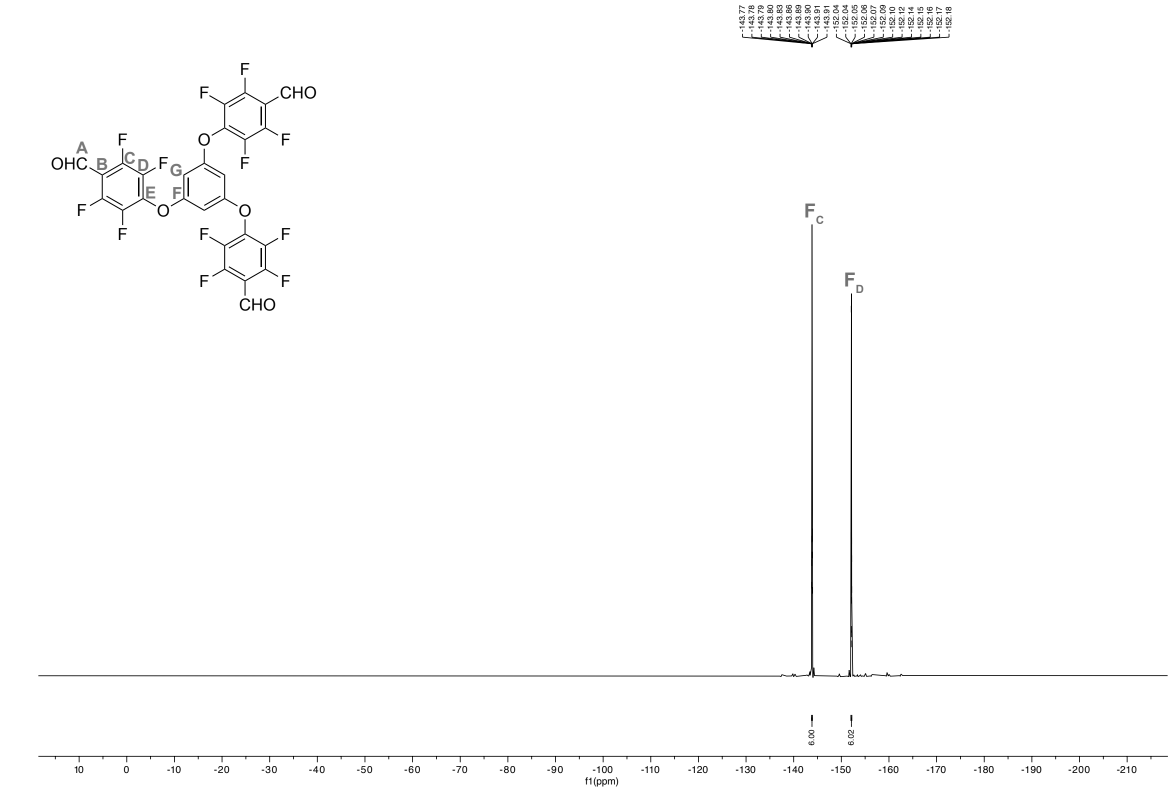

Figure S83: ^{19}F NMR (282 MHz, $CDCl_3$) of fluorinated aldehyde **F**.

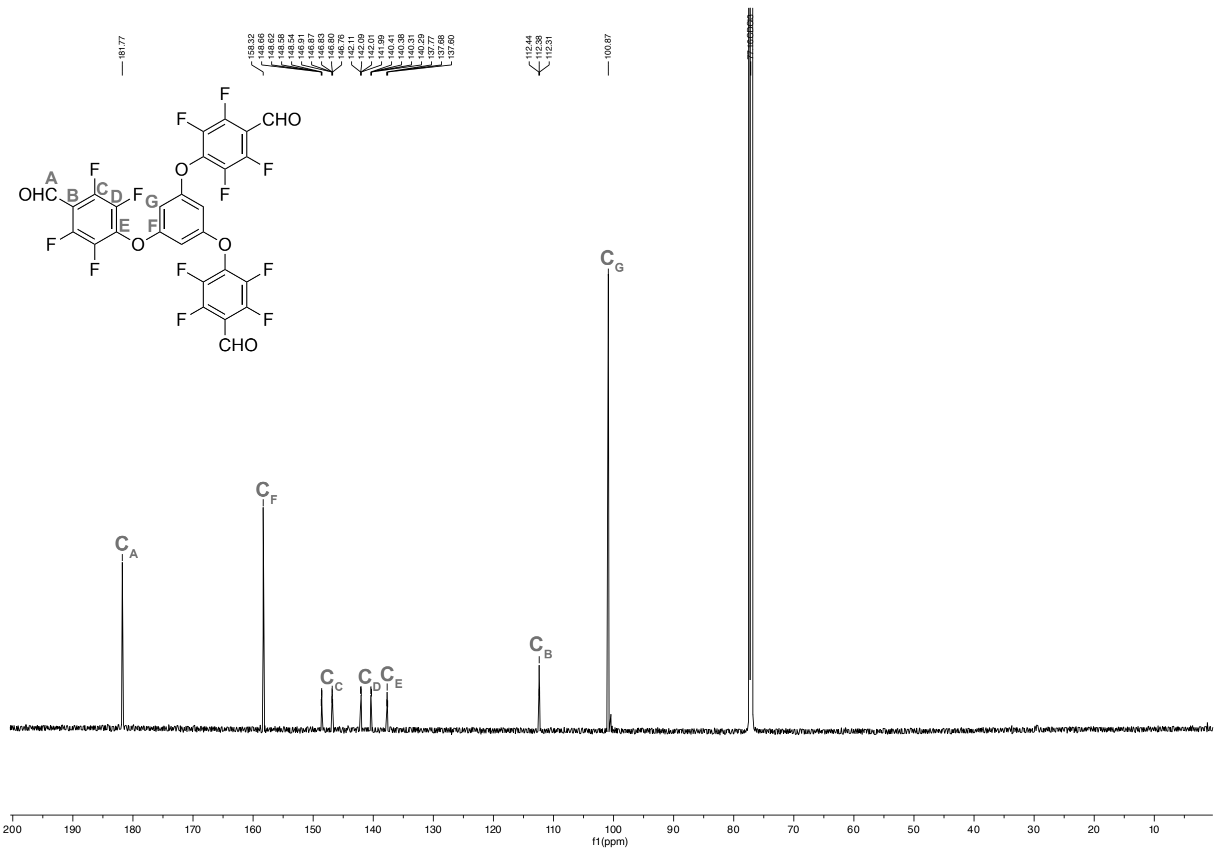

Figure S84: $^{13}C\{^1H\}$ NMR (151 MHz, $CDCl_3$) of fluorinated aldehyde **F**.

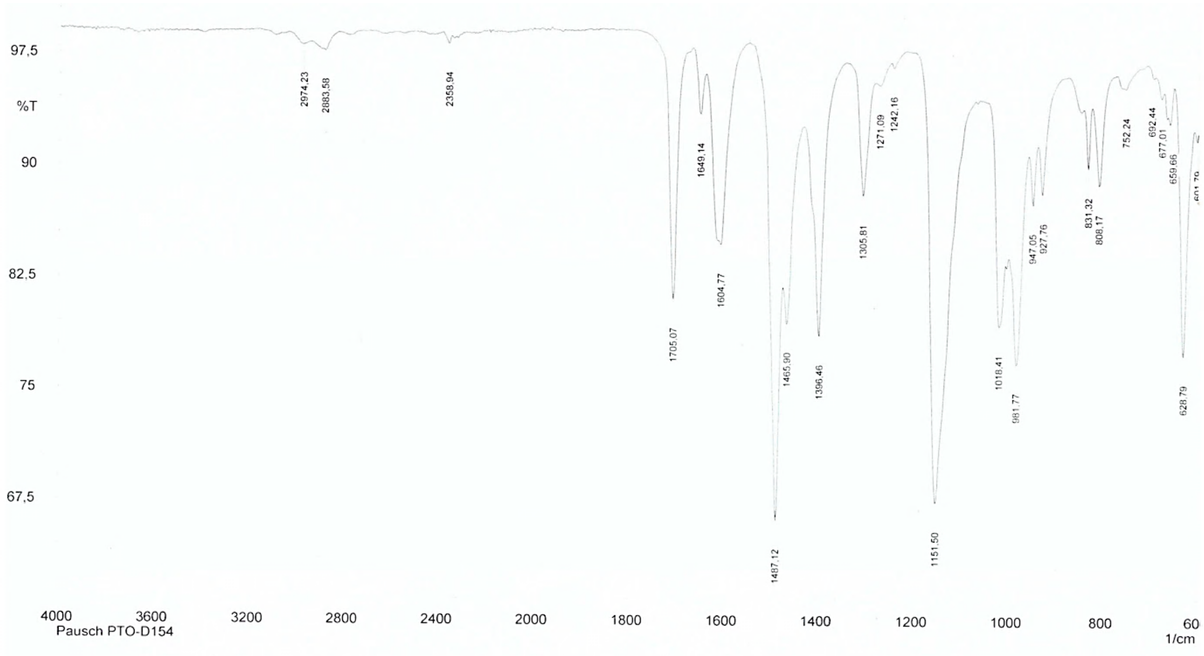

Figure S85: AT-IR spectrum of fluorinated aldehyde **F**.

Acquisition Parameter

Source Type	ESI	Ion Polarity	Positive	Set Nebulizer	0.3 Bar
Focus	Not active	Set Capillary	4000 V	Set Dry Heater	180 °C
Scan Begin	50 m/z	Set End Plate Offset	-500 V	Set Dry Gas	4.0 l/min
Scan End	1500 m/z	Set Collision Cell RF	600.0 Vpp	Set Divert Valve	Source

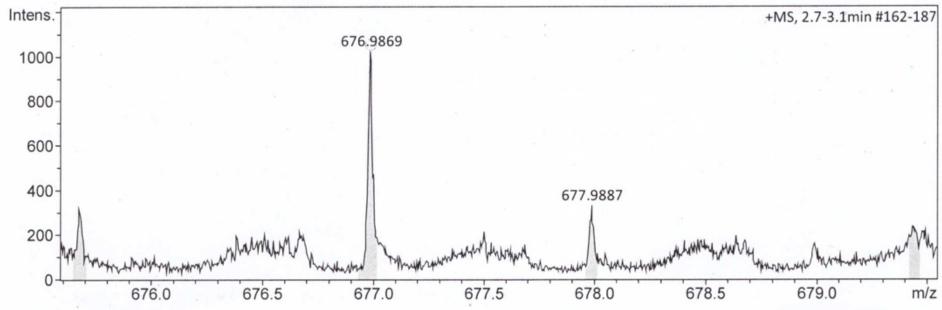

Meas. m/z	#	Ion Formula	m/z	err [ppm]	mSigma	# mSigma	Score	rdb	e⁻ Conf	N-Rule
676.9869	1	C28H2F12N4NaO2	676.9878	1.4	30.9	1	56.01	23.5	even	ok
	2	C27H6F12NaO6	676.9865	-0.6	31.0	2	100.00	18.5	even	ok

Figure S86: HR-MS (ESI) spectrum of fluorinated aldehyde **F**.

Aldehyde H

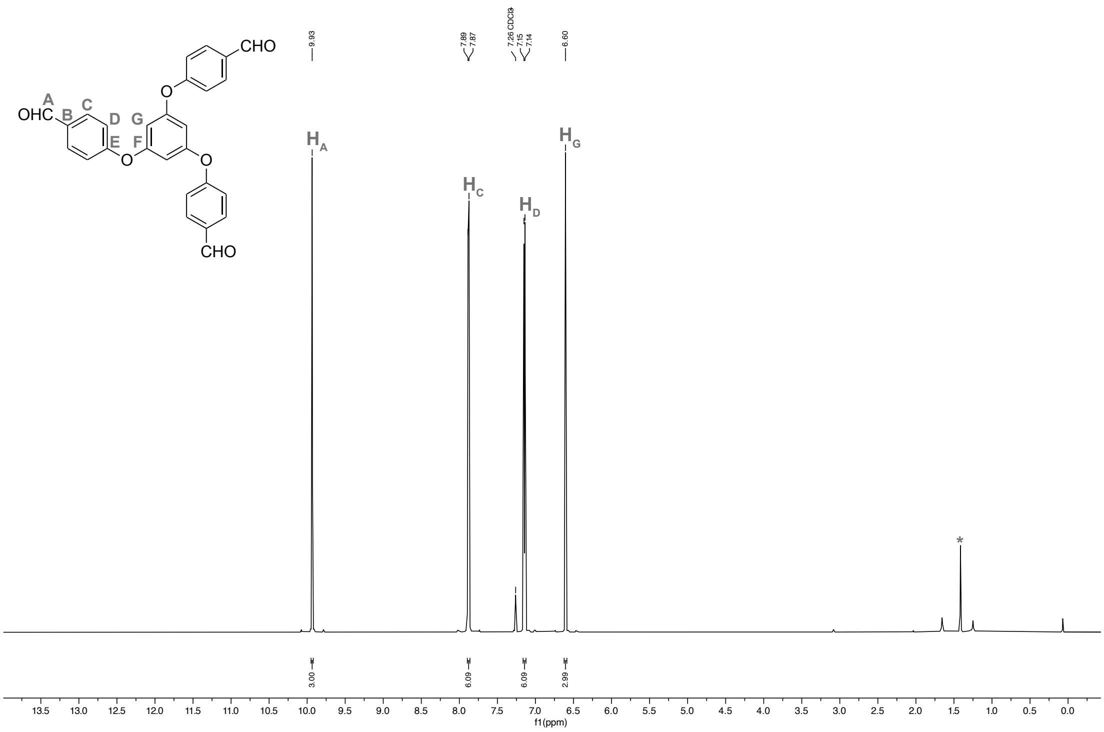

Figure S87: ^{1}H NMR (600 MHz, $CDCl_3$) of non-fluorinated aldehyde **H**. *Cyclohexane

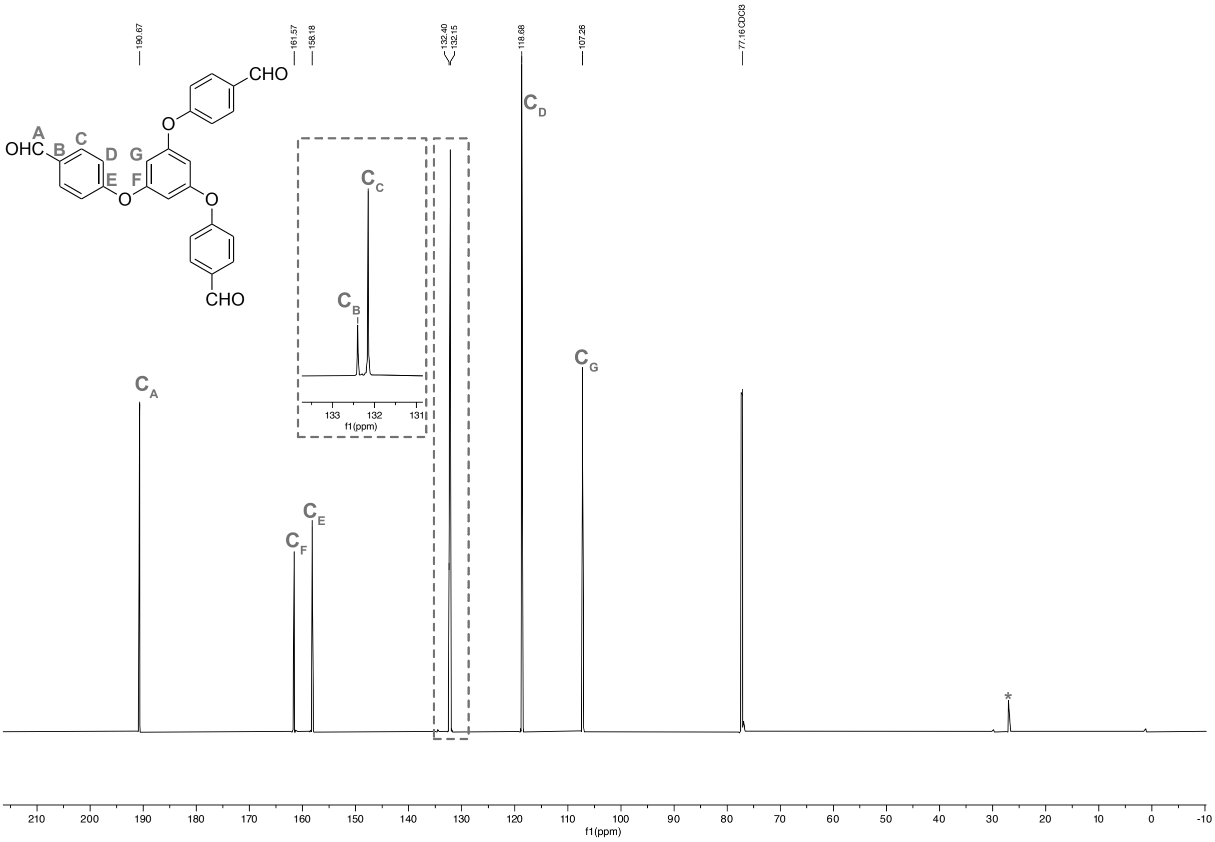

Figure S88: $^{13}C\{^1H\}$ NMR (151 MHz, $CDCl_3$) of non-fluorinated aldehyde **H**. *Cyclohexane

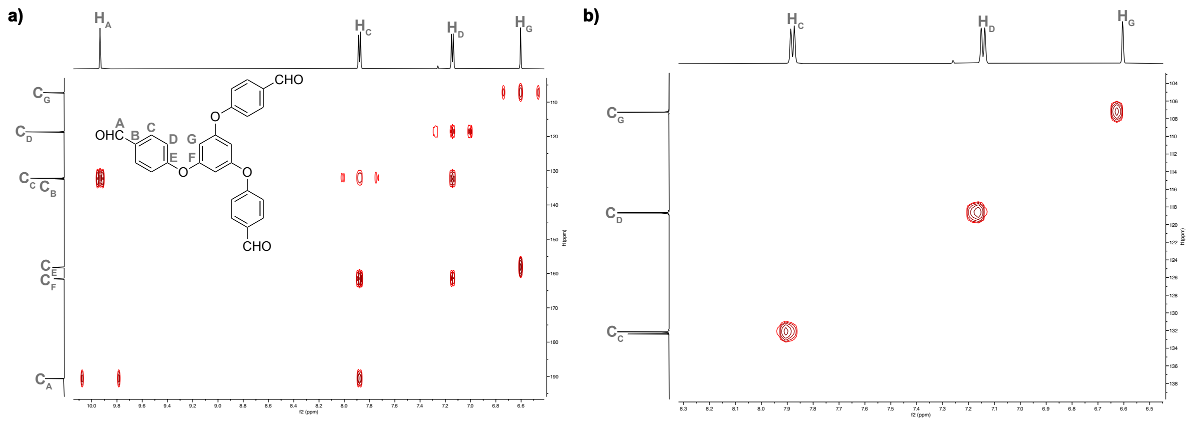

Figure S89: a) ^{1}H-^{13}C HSQC NMR (600 MHz, 151 MHz, $CDCl_3$) and b) ^{1}H-^{13}C HMBC NMR (600 MHz, 151 MHz, $CDCl_3$) of non-fluorinated aldehyde **H**.

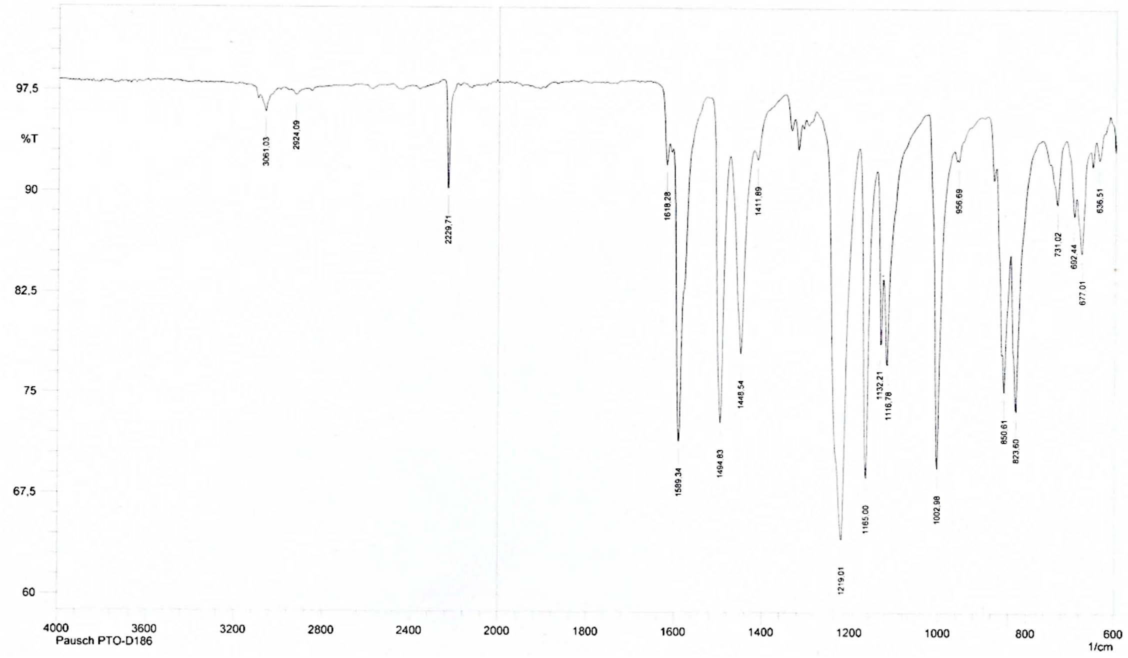

Figure S90: AT-IR spectrum of non-fluorinated aldehyde **H**.

Acquisition Parameter

Source Type	ESI	Ion Polarity	Positive	Set Nebulizer	0.3 Bar
Focus	Not active	Set Capillary	4000 V	Set Dry Heater	180 °C
Scan Begin	50 m/z	Set End Plate Offset	-500 V	Set Dry Gas	4.0 l/min
Scan End	1500 m/z	Set Collision Cell RF	600.0 Vpp	Set Divert Valve	Source

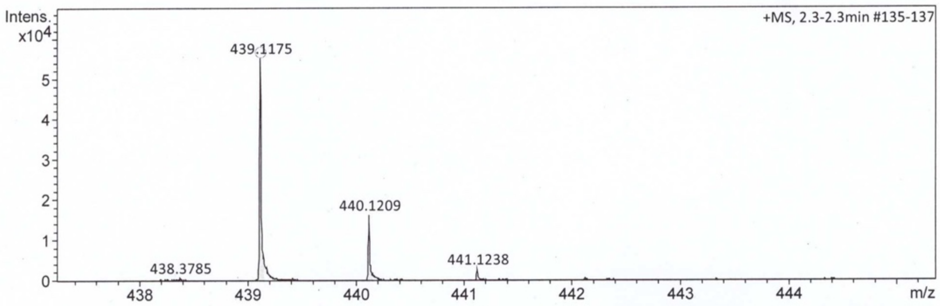

Meas. m/z	#	Ion Formula	m/z	err [ppm]	mSigma	# mSigma	Score	rdb	e⁻ Conf	N-Rule
439.1175	1	C27H19O6	439.1176	0.2	2.6	1	100.00	18.5	even	ok

Figure S91: HR-MS (ESI) spectrum of non-fluorinated aldehyde **F**.

Fluorinated Imine Cage Et^2F^2

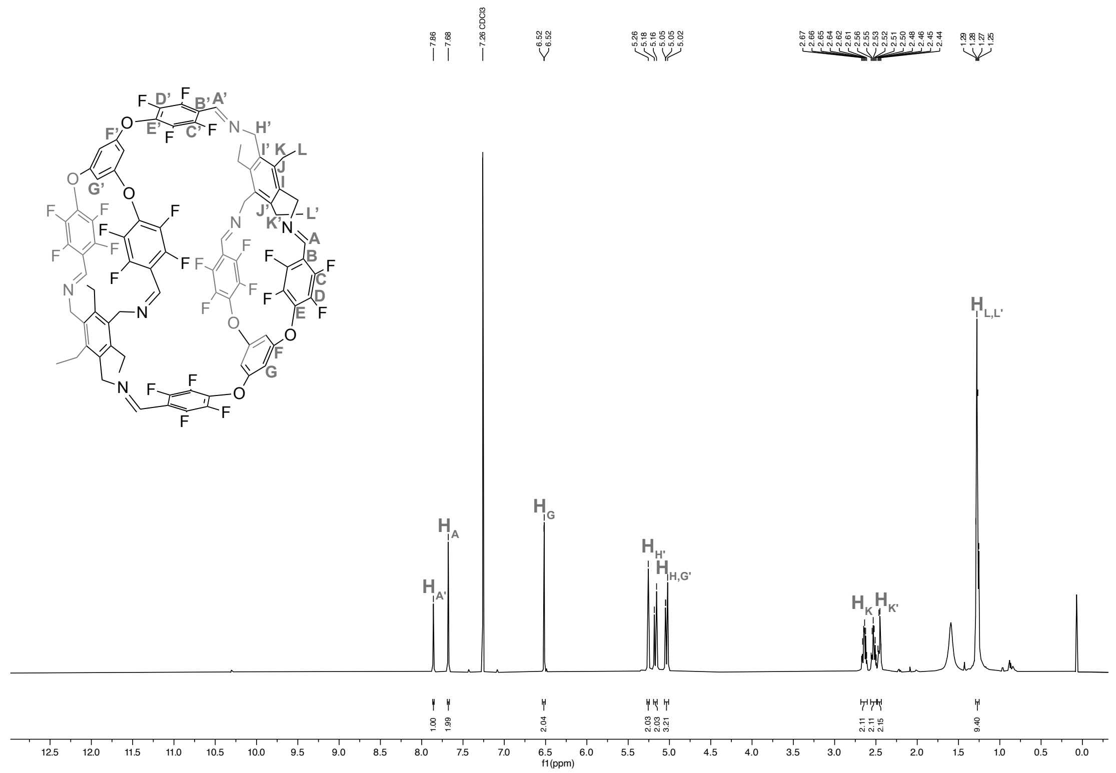

Figure S92: ^1H NMR (600 MHz, $CDCl_3$) of fluorinated imine cage $\mathbf{Et^2F^2}$.

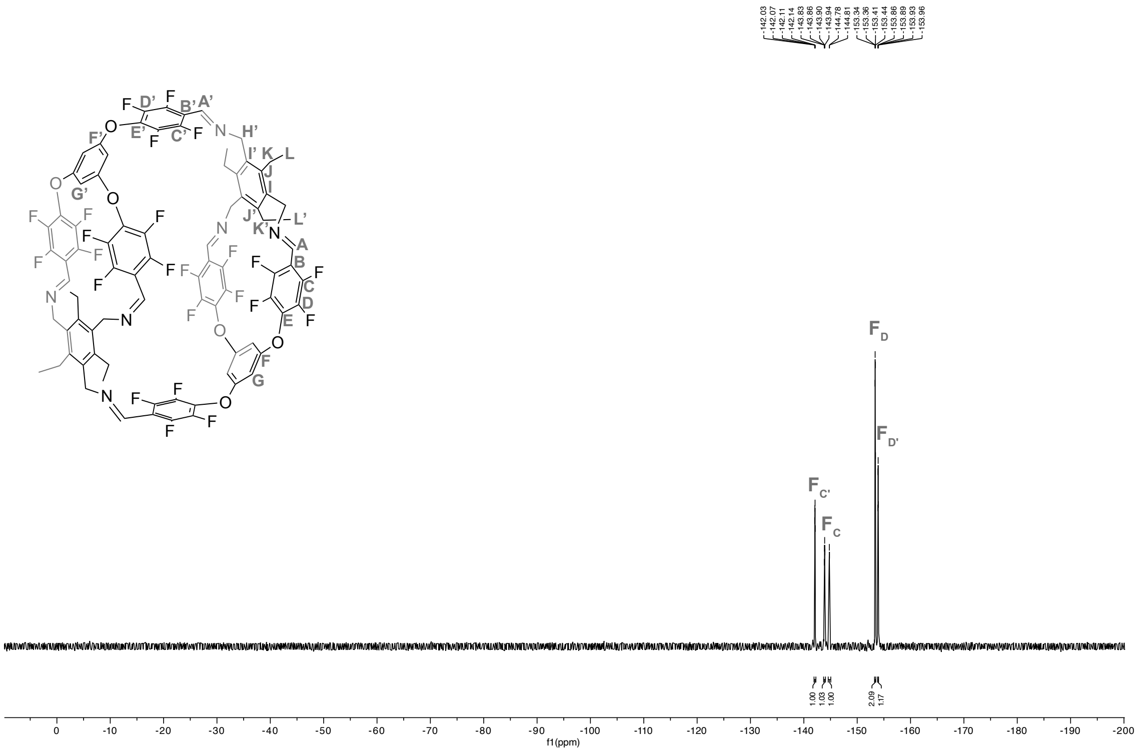

Figure S93: ^{19}F NMR (282 MHz, $CDCl_3$) of fluorinated imine cage $\mathbf{Et^2F^2}$.

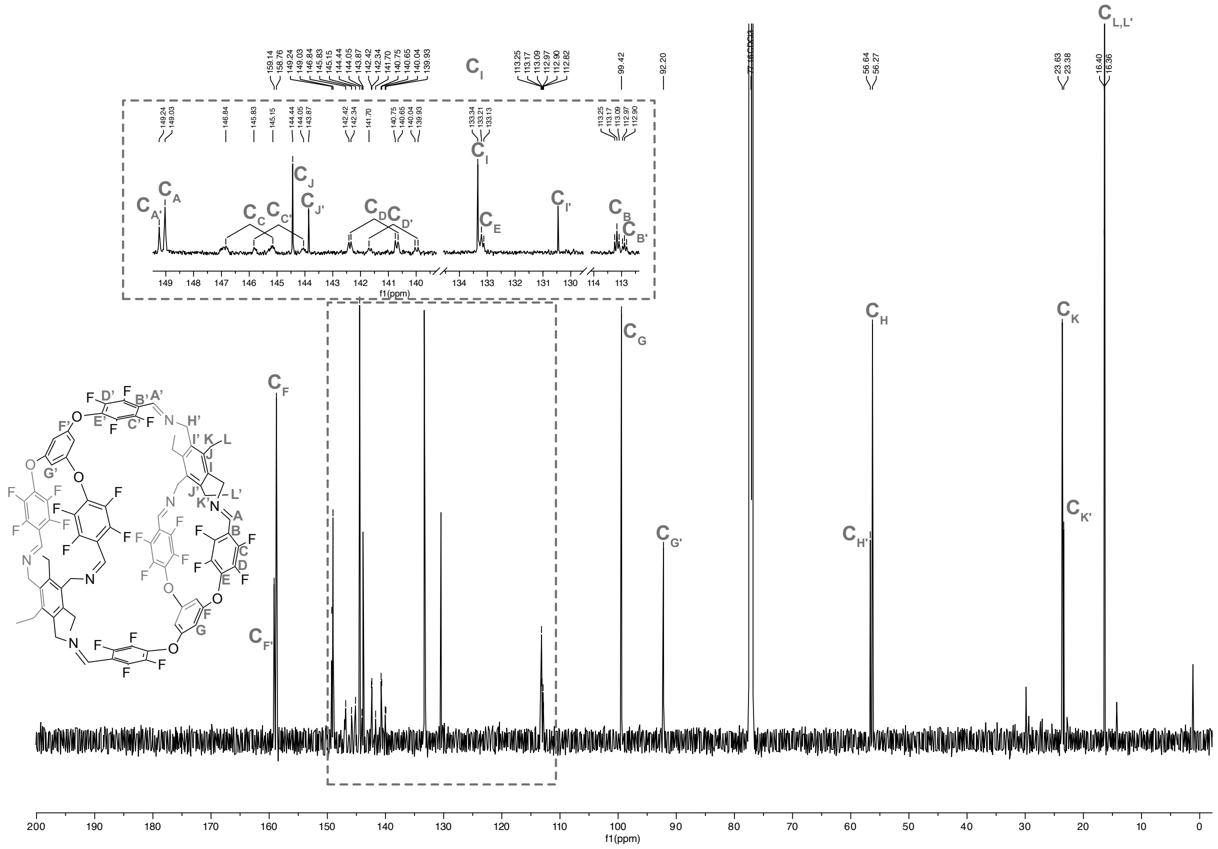

Figure S94: $^{13}C\{^{1}H\}$ NMR (151 MHz, $CDCl_3$) of fluorinated imine cage **Et^{2}F^2**.

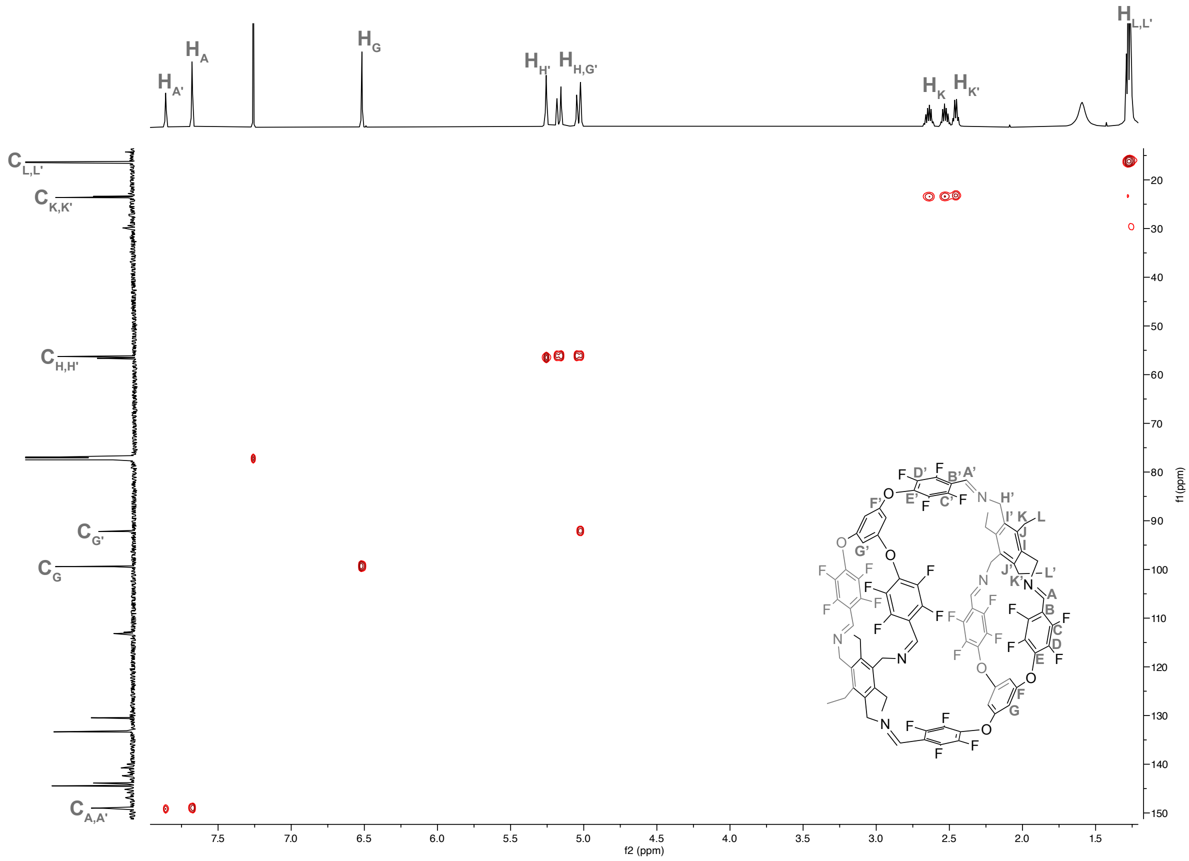

Figure S95: ^{1}H-^{13}C HSQC NMR (600 MHz, 151 MHz, $CDCl_3$) of fluorinated imine cage **Et^{2}F^2**.

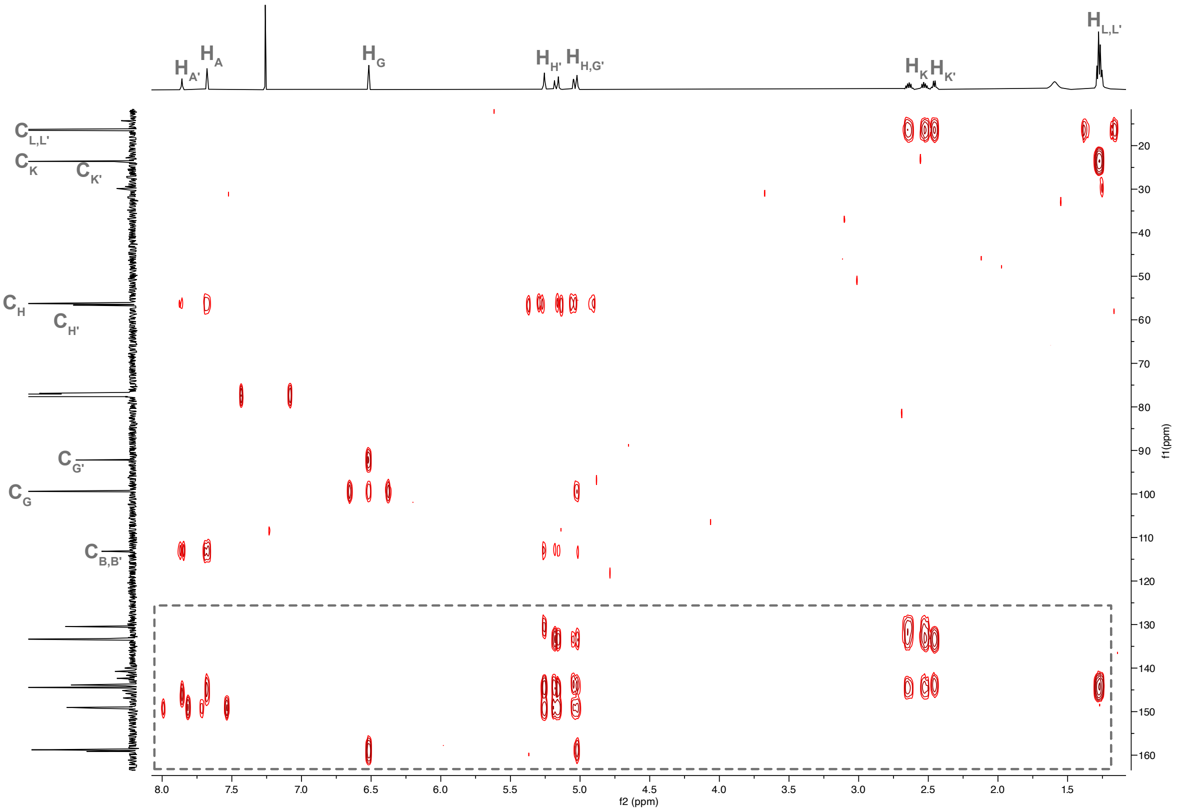

Figure S96: ^{1}H-^{13}C HMBC NMR (600 MHz, 151 MHz, $CDCl_3$) of fluorinated imine cage **Et^{2}F^{2}**.

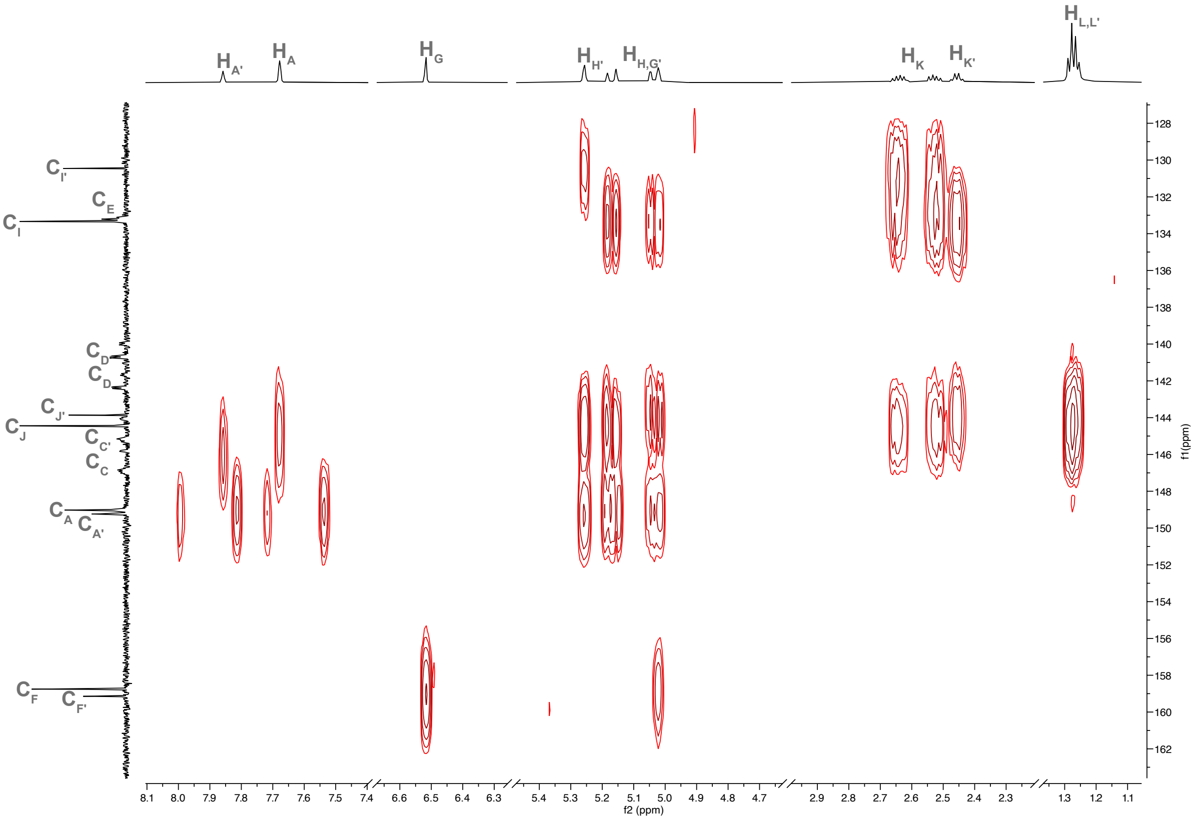

Figure S97: ^{1}H-^{13}C HMBC NMR (600 MHz, 151 MHz, $CDCl_3$) of fluorinated imine cage **Et^{2}F^{2}**, excerpt of the aromatic region.

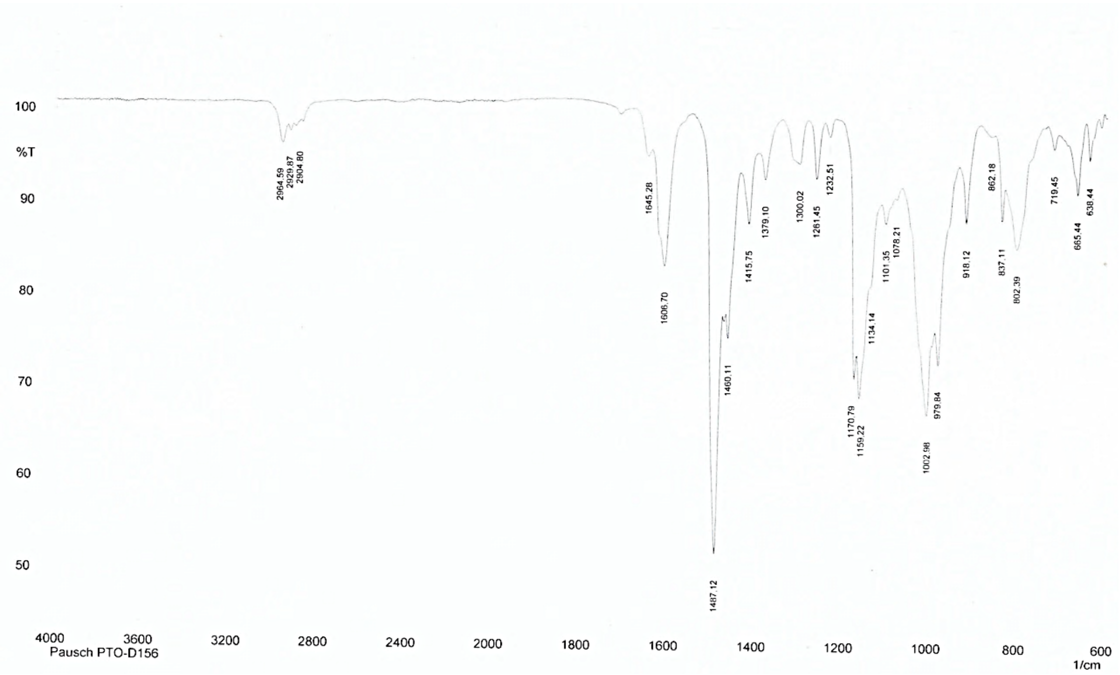

Figure S98: AT-IR spectrum of fluorinated imine cage **Et²F²**.

Acquisition Parameter

Source Type	ESI	Ion Polarity	Positive	Set Nebulizer	0.3 Bar
Focus	Active	Set Capillary	4000 V	Set Dry Heater	180 °C
Scan Begin	300 m/z	Set End Plate Offset	-500 V	Set Dry Gas	4.0 l/min
Scan End	2800 m/z	Set Collision Cell RF	2500.0 Vpp	Set Divert Valve	Source

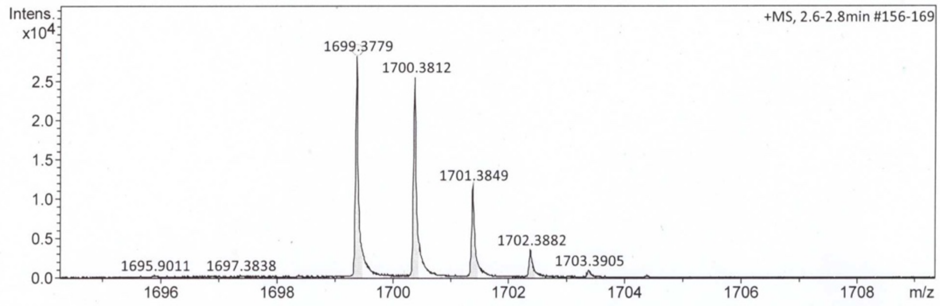

Meas. m/z	#	Ion Formula	m/z	err [ppm]	mSigma	# mSigma	Score	rdb	e⁻ Conf	N-Rule
1699.3779	1	C80H51F24N12O4	1699.3768	-0.7	7.1	1	66.75	49.5	even	ok
	2	C83H59F24N2O10	1699.3781	0.1	12.1	2	100.00	43.5	even	ok
	3	C81H47F24N16	1699.3781	0.1	18.9	3	87.58	54.5	even	ok
	4	C84H55F24N6O6	1699.3794	0.9	22.7	4	38.64	48.5	even	ok
	5	C85H51F24N10O2	1699.3808	1.7	34.5	5	9.95	53.5	even	ok

Figure S99: HR-MS (ESI) spectrum of fluorinated imine cage **Et²F²**.

Fluorinated Imine Cage TREN^{2}F^2

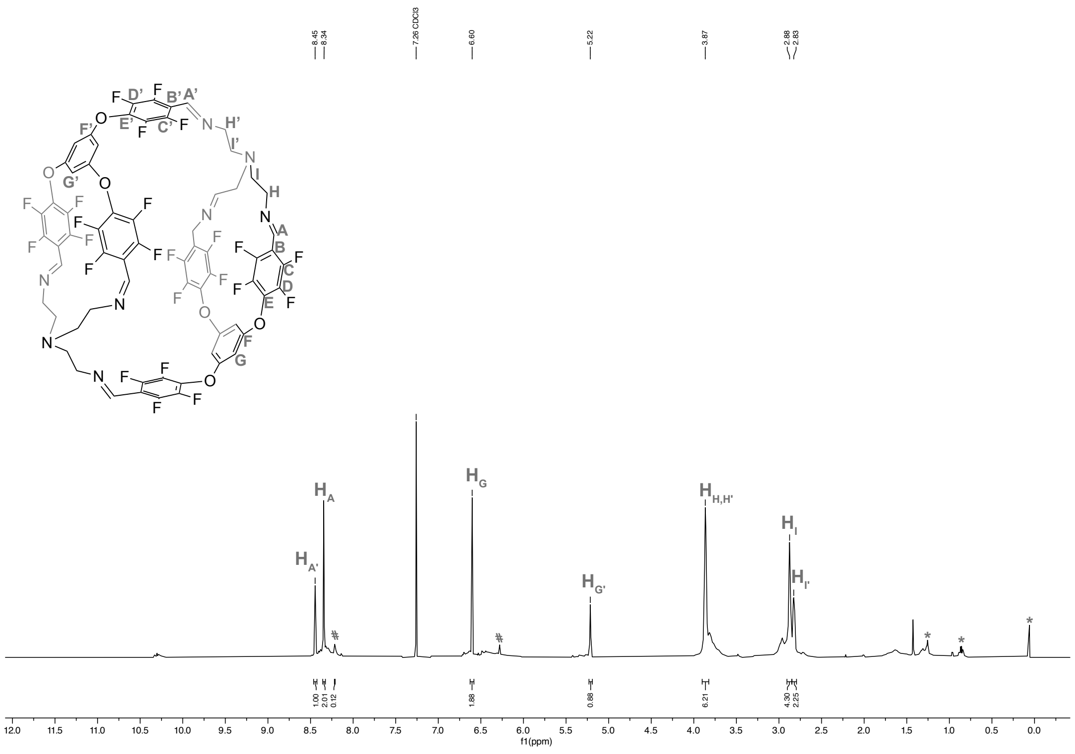

Figure S100: ^{1}H NMR (600 MHz, $CDCl_3$) of fluorinated imine cage **TREN^{2}F^2** the signals attributed to **TREN^{4}F^4** are marked with a hash (#). *H- and silicon grease

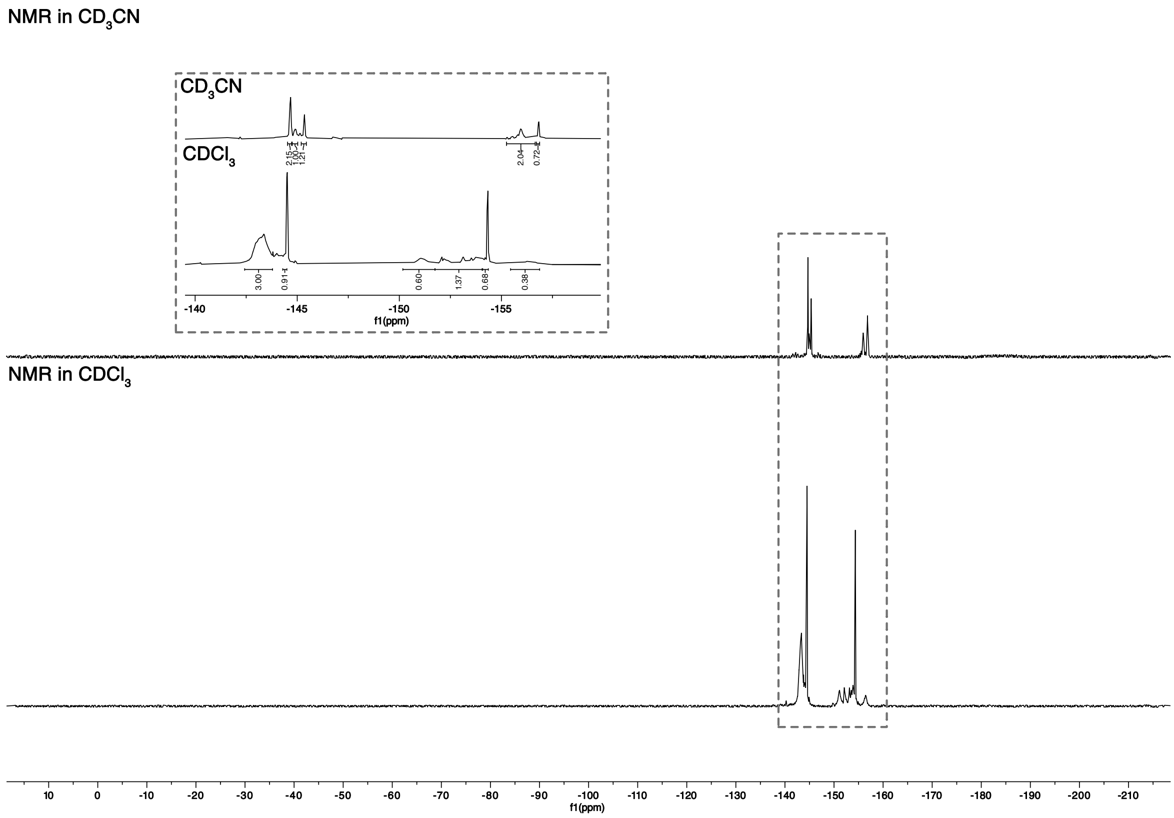

Figure S101: Stacked ^{19}F NMR (565 MHz) of fluorinated imine cage **TREN^{2}F^2** in CD3CN (top) and CDCl3 (bottom) the signals attributed to **TREN^{4}F^4** are marked with an asterisk.

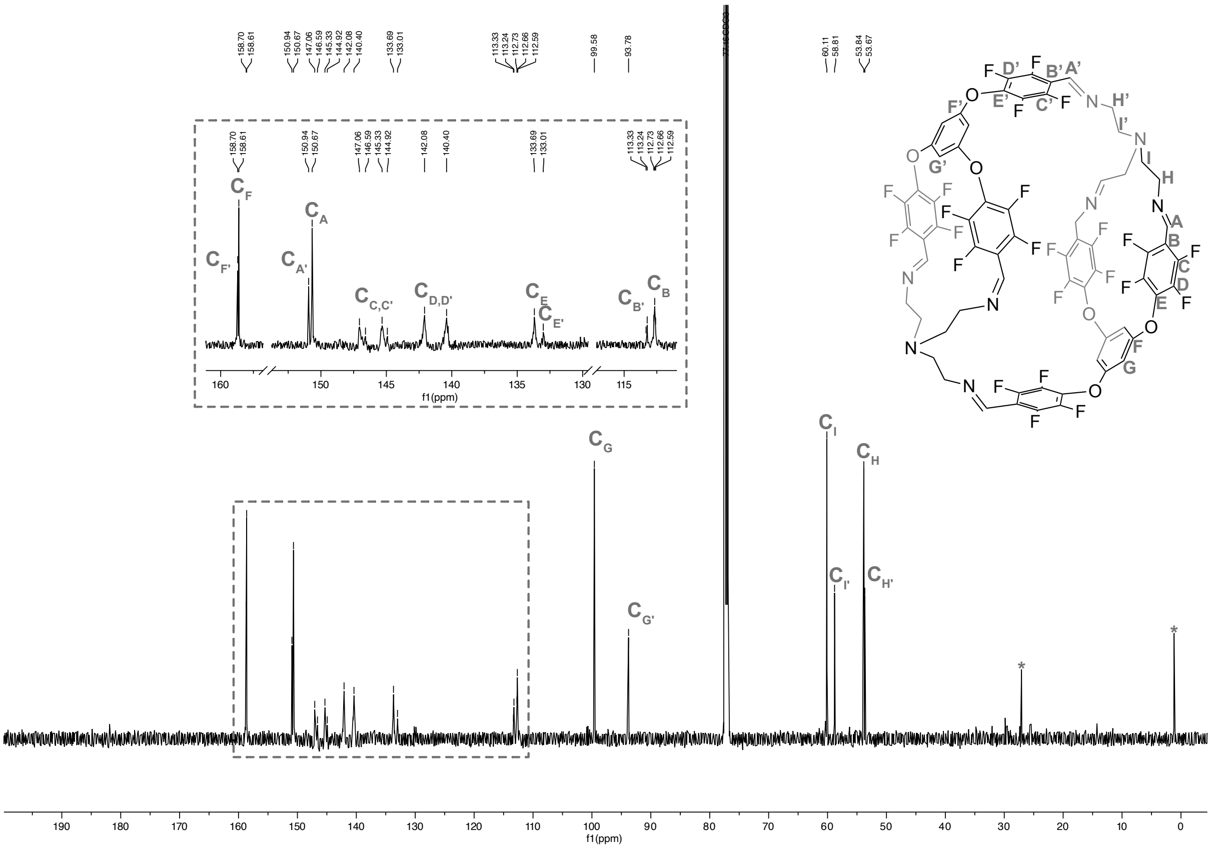

Figure S102: $^{13}C\{^{1}H\}$ NMR (151 MHz, $CDCl_3$) of fluorinated imine cage **TREN²F²**.

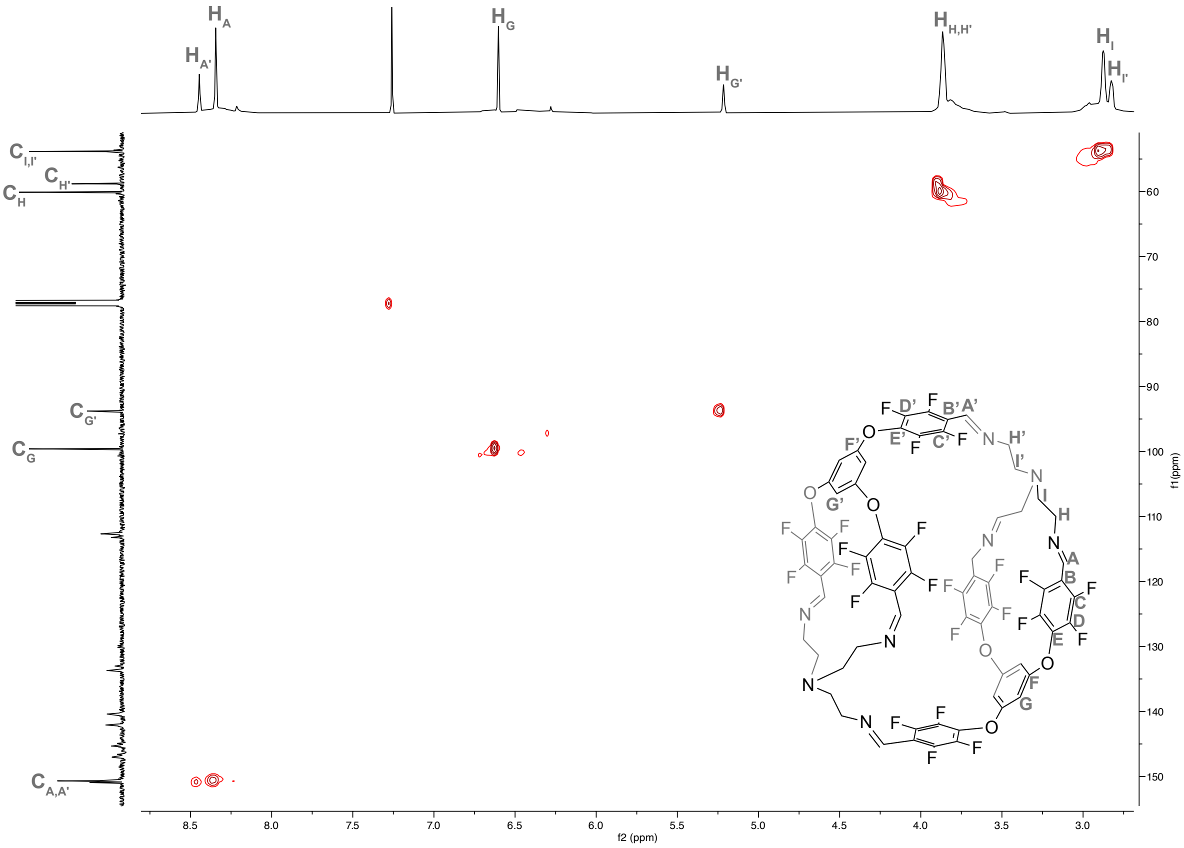

Figure S103: ^{1}H-^{13}C HSQC NMR (600 MHz, 151 MHz, $CDCl_3$) of fluorinated imine cage **TREN²F²**.

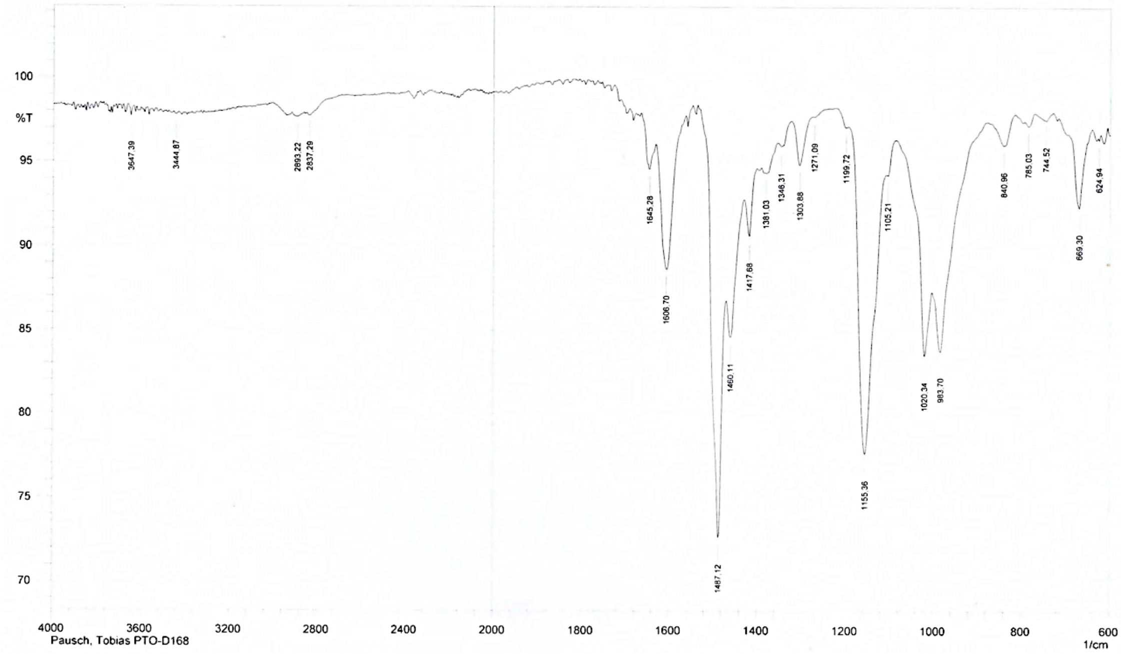

Figure S104: AT-IR spectrum of fluorinated imine cage **TREN^{2}F^2**.

Acquisition Parameter

Source Type	ESI	Ion Polarity	Positive	Set Nebulizer	0.3 Bar
Focus	Active	Set Capillary	4000 V	Set Dry Heater	180 °C
Scan Begin	300 m/z	Set End Plate Offset	-500 V	Set Dry Gas	4.0 l/min
Scan End	2800 m/z	Set Collision Cell RF	2500.0 Vpp	Set Divert Valve	Source

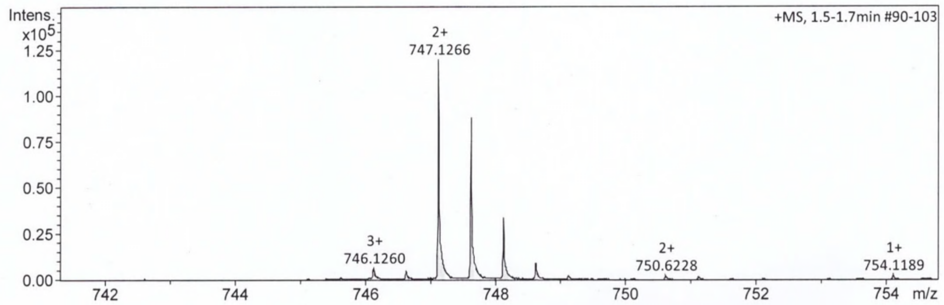

Meas. m/z	#	Ion Formula	m/z	err [ppm]	mSigma	# mSigma	Score	rdb	e⁻ Conf	N-Rule
747.1266	1	C66H38F24N8O6	747.1260	-0.8	5.7	1	94.16	40.0	even	ok
	2	C65H42F24N4O10	747.1253	-1.7	6.3	2	63.82	35.0	even	ok
	3	C63H30F24N18	747.1253	-1.7	8.3	3	61.41	46.0	even	ok
	4	C62H34F24N14O4	747.1247	-2.6	10.5	4	37.14	41.0	even	ok
	5	C67H34F24N12O2	747.1267	0.0	16.7	5	100.00	45.0	even	ok
	6	C64H46F24O14	747.1247	-2.6	17.2	6	32.64	30.0	even	ok
	7	C70H42F24N2O8	747.1273	1.0	19.3	7	69.53	39.0	even	ok

Figure S105: HR-MS (ESI) spectrum of fluorinated imine cage **TREN^{2}F^2**.

Imine Cage Et^2H^2

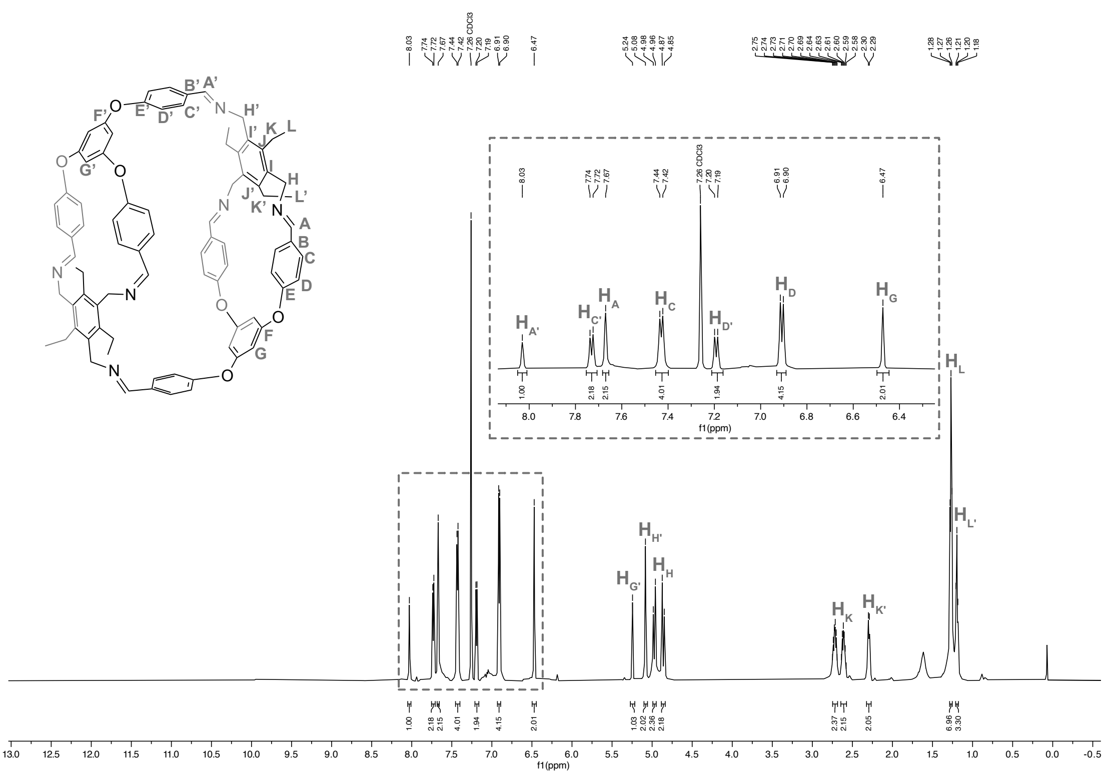

Figure S106: ^{1}H NMR (600 MHz, $CDCl_3$) of imine cage **Et^2H^2**.

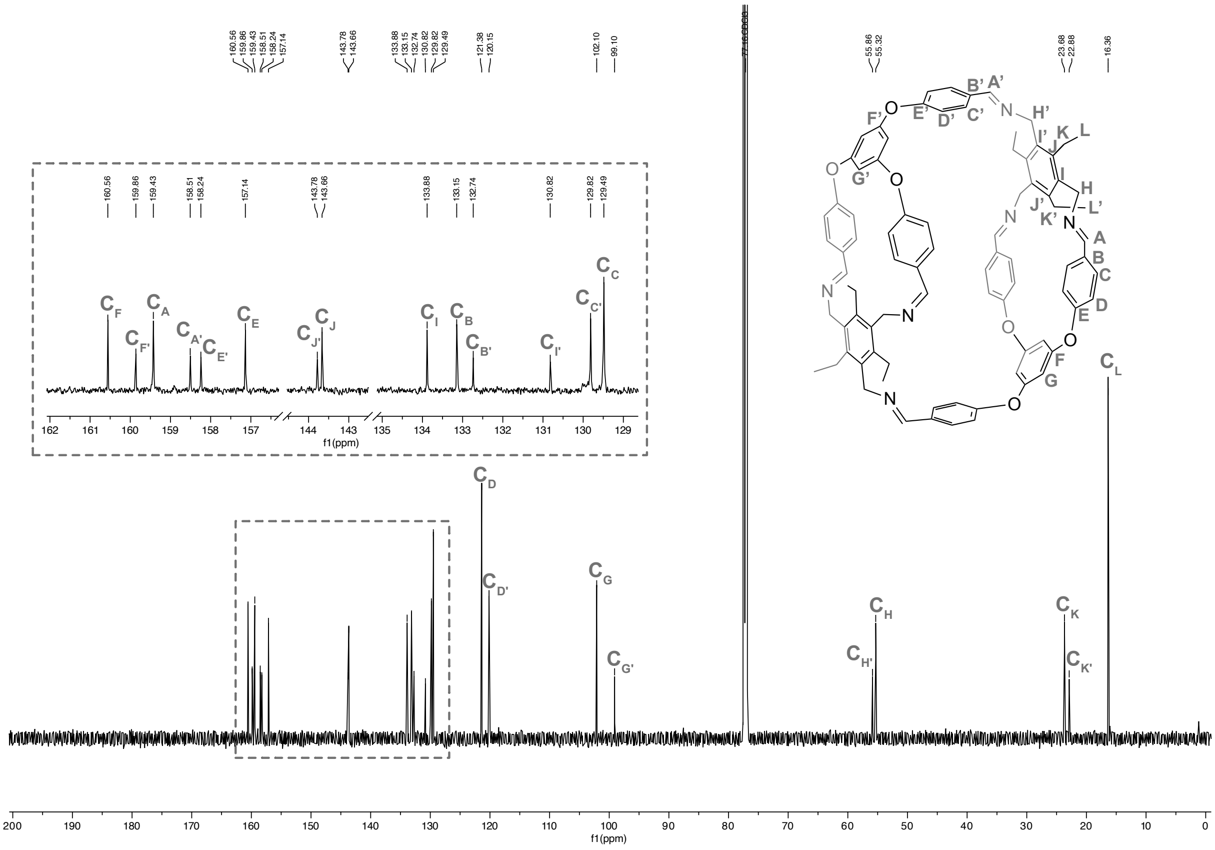

Figure S107: $^{13}C\{^1H\}$ NMR (151 MHz, $CDCl_3$) of imine cage **Et^2H^2**.

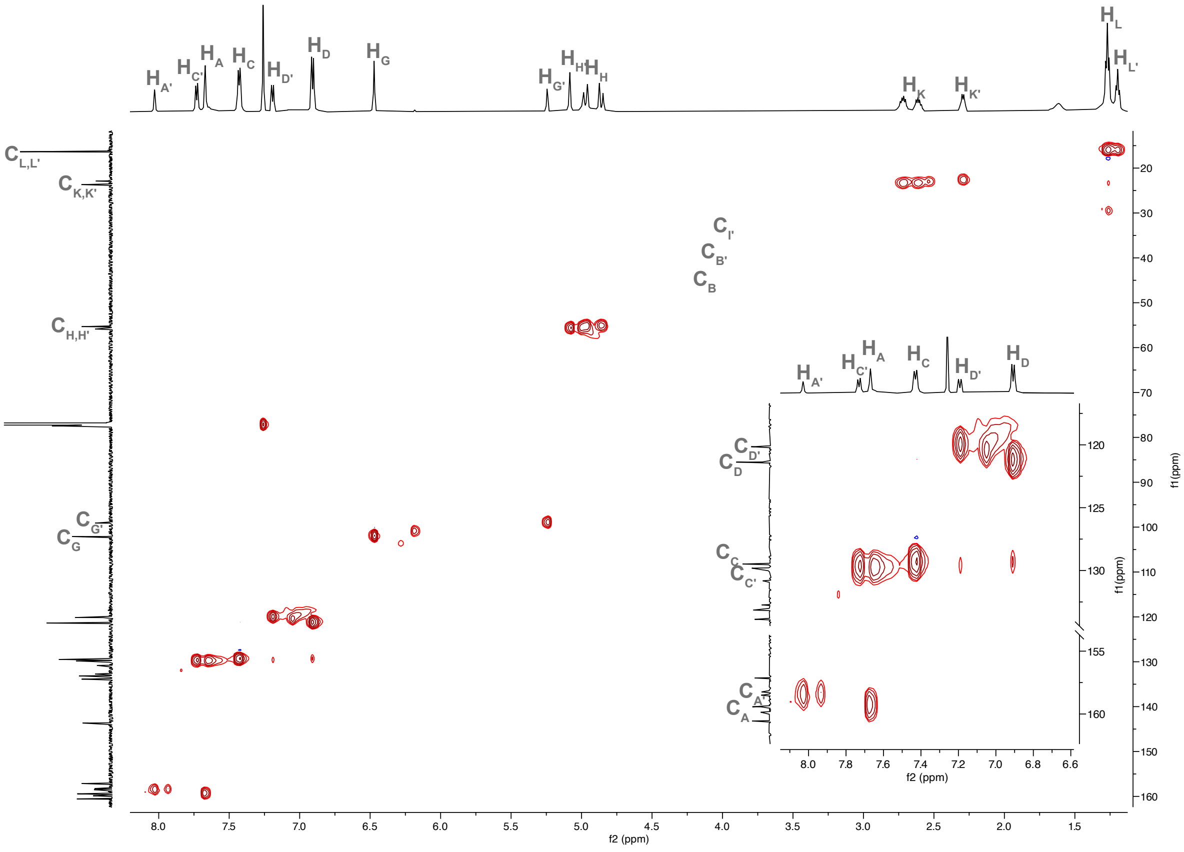

Figure S108: ^{1}H-^{13}C HSQC NMR (600 MHz, 151 MHz, $CDCl_3$) of imine cage **Et^{2}H^2**.

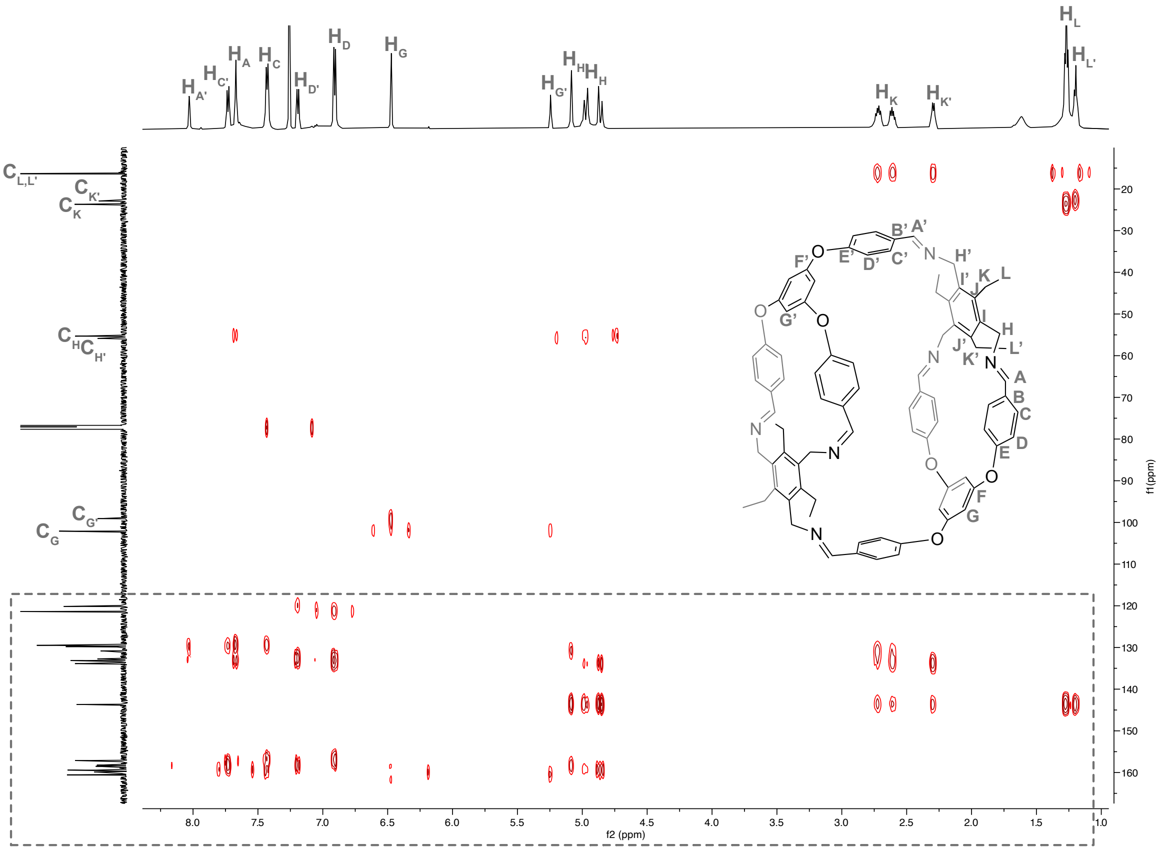

Figure S109: ^{1}H-^{13}C HMBC NMR (600 MHz, 151 MHz, $CDCl_3$) of imine cage **Et^{2}H^2**.

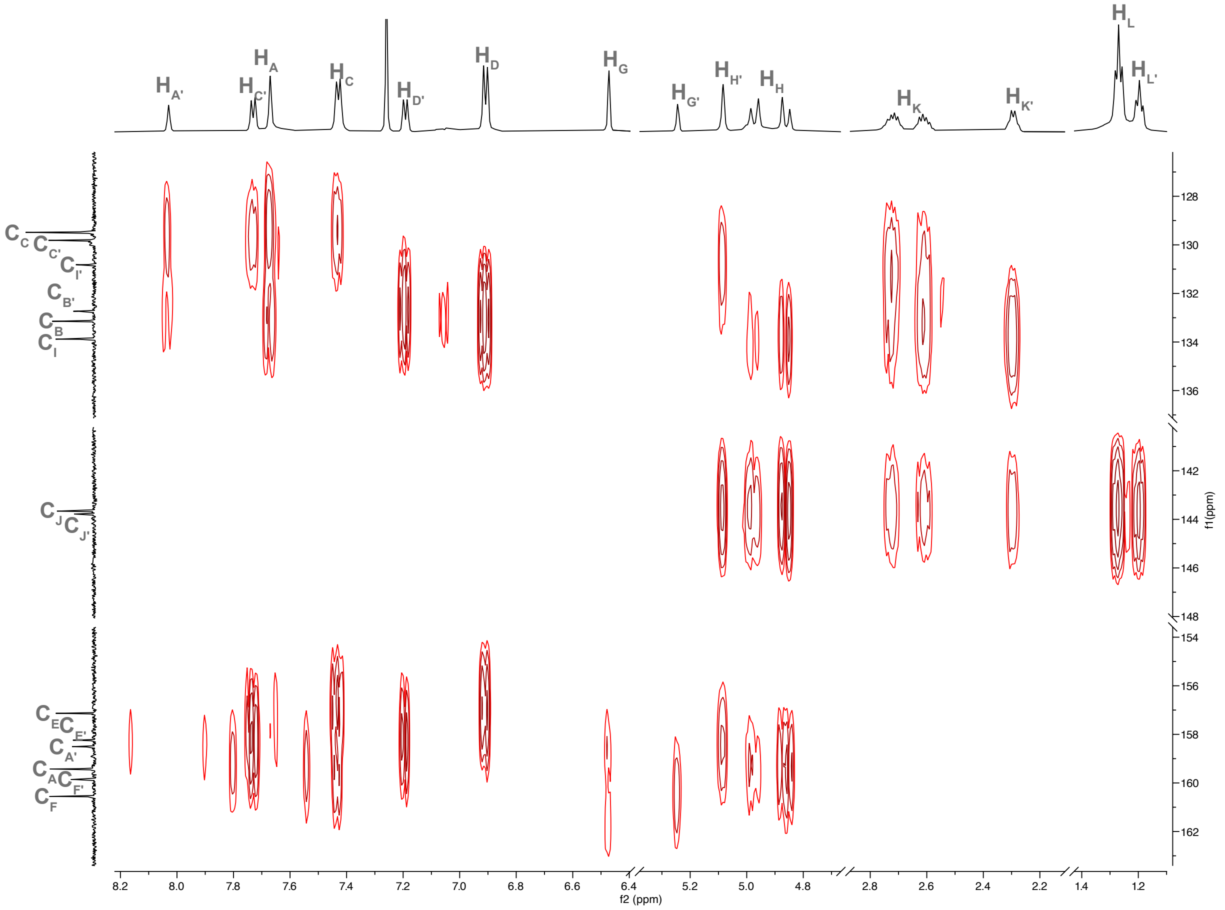

Figure S110: ^{1}H-^{13}C HMBC NMR (600 MHz, 151 MHz, $CDCl_3$) of imine cage **Et²H²**, excerpt of the aromatic region.

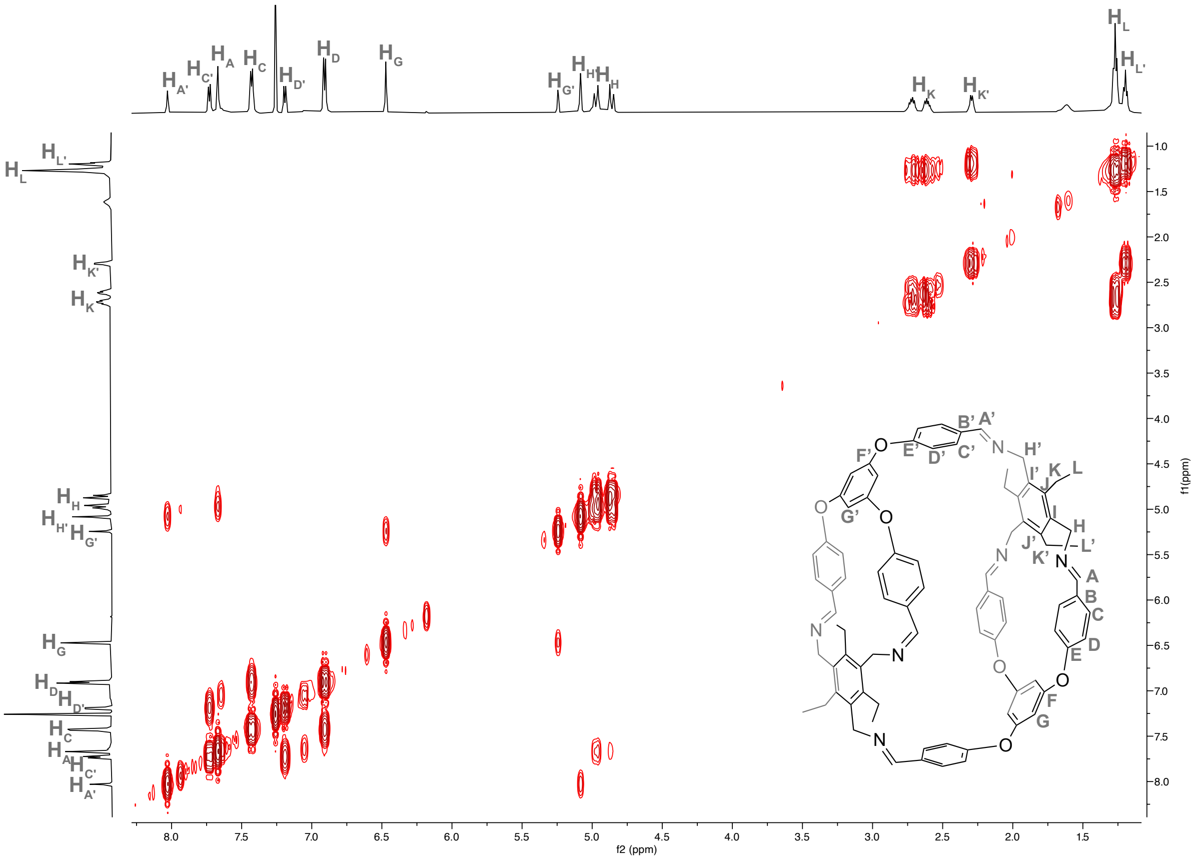

Figure S111: ^{1}H-COSY NMR (600 MHz, $CDCl_3$) of imine cage **Et^2H^2**.

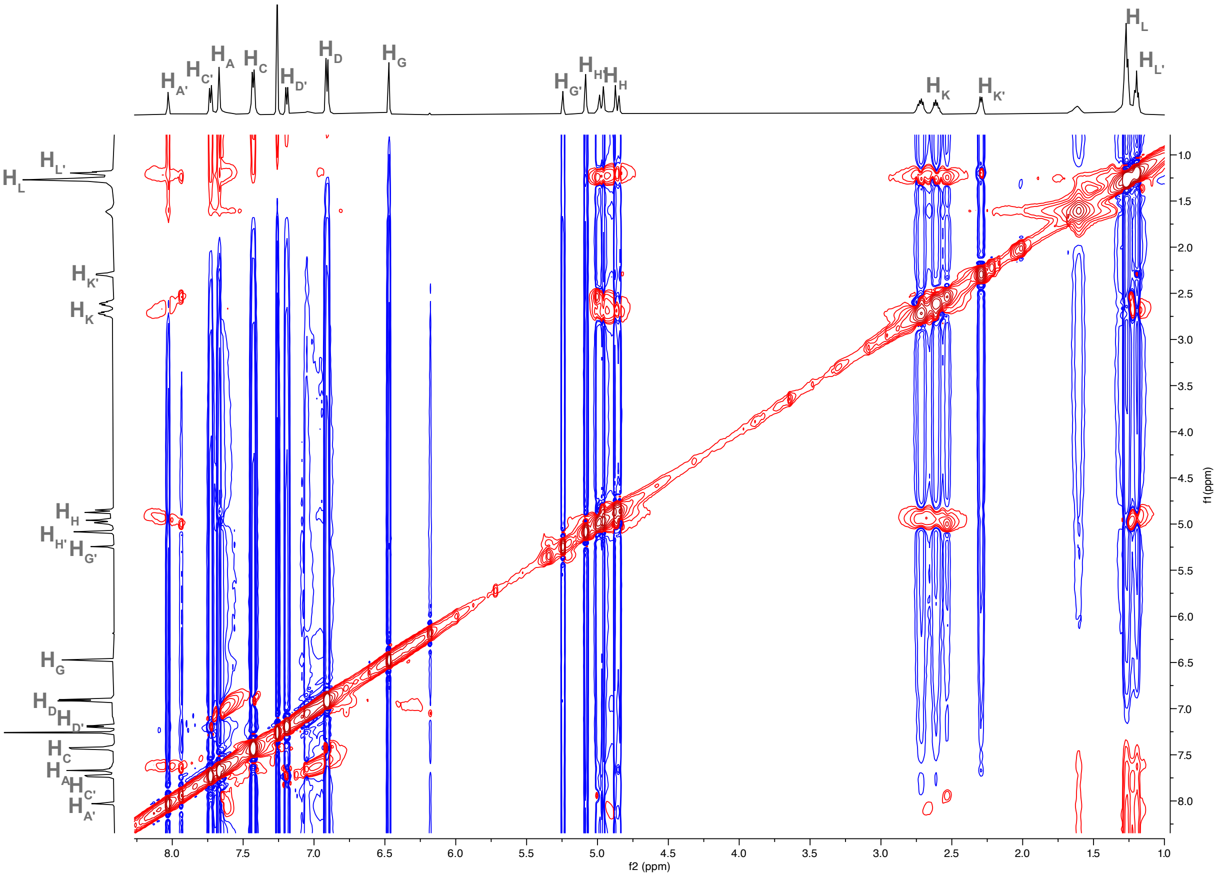

Figure S112: ^{1}H-NOESY NMR (600 MHz, $CDCl_3$) of imine cage **Et^2H^2**.

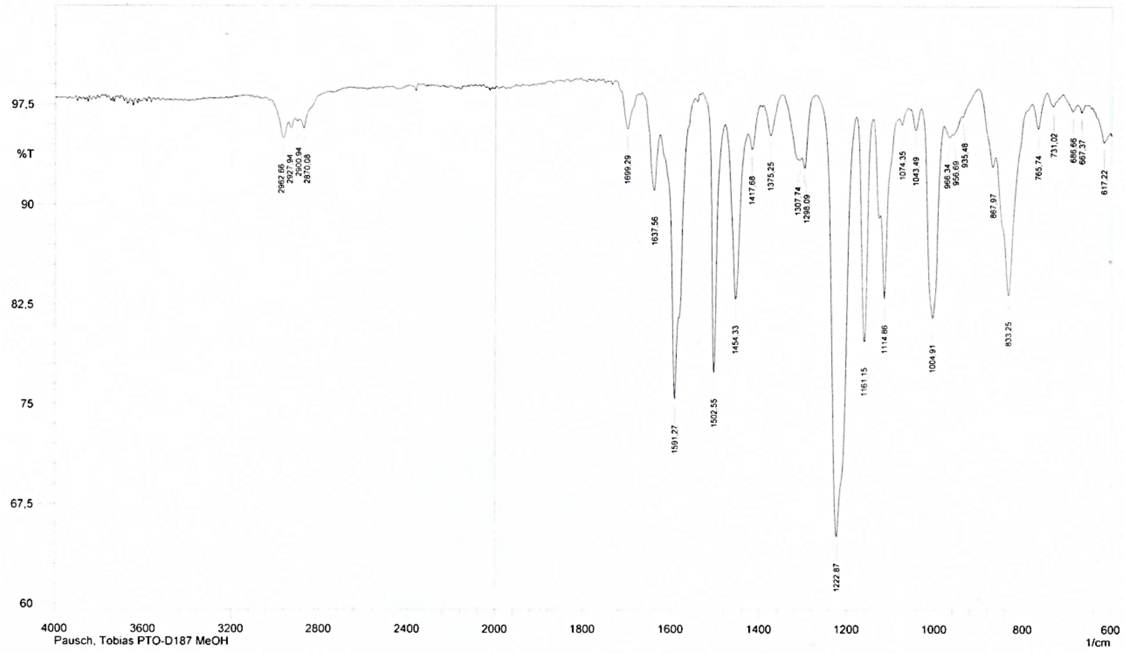

Figure S113: AT-IR spectrum of imine cage **Et²H²**.

Acquisition Parameter

Source Type	ESI	Ion Polarity	Positive	Set Nebulizer	0.3 Bar
Focus	Active	Set Capillary	4000 V	Set Dry Heater	180 °C
Scan Begin	300 m/z	Set End Plate Offset	-500 V	Set Dry Gas	4.0 l/min
Scan End	2800 m/z	Set Collision Cell RF	2500.0 Vpp	Set Divert Valve	Source

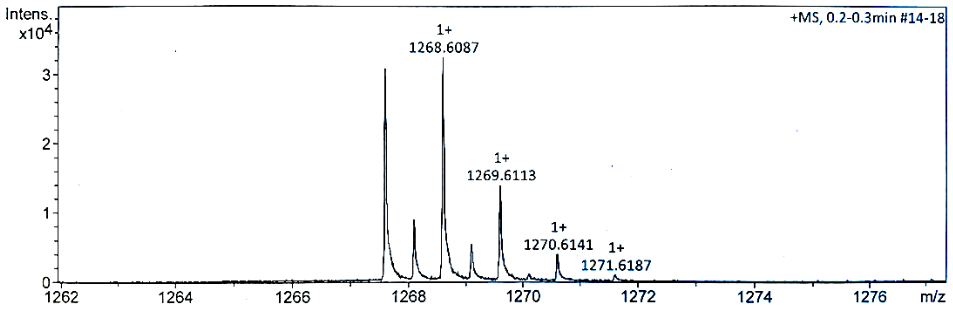

Meas. m/z	#	Ion Formula	m/z	err [ppm]	mSigma	# mSigma	Score	rdb	e⁻ Conf	N-Rule
634.3074	1	C84H80N6O6	634.3064	-1.5	20.4	1	94.90	48.0	even	ok
	2	C85H76N10O2	634.3071	-0.4	32.6	2	100.00	53.0	even	ok
	3	C88H84O8	634.3078	0.6	35.8	3	87.72	47.0	even	ok
	4	C89H80N4O4	634.3084	1.7	48.1	4	43.87	52.0	even	ok
1267.6049	1	C85H75N10O2	1267.6069	1.6	31.8	1	47.82	53.5	even	ok
	2	C84H79N6O6	1267.6056	0.5	35.8	2	100.00	48.5	even	ok
	3	C83H83N2O10	1267.6042	-0.6	43.1	3	79.26	43.5	even	ok

Figure S114: HR-MS (ESI) spectrum of imine cage **Et²H²**.

Imine Cage Et⁴H⁴

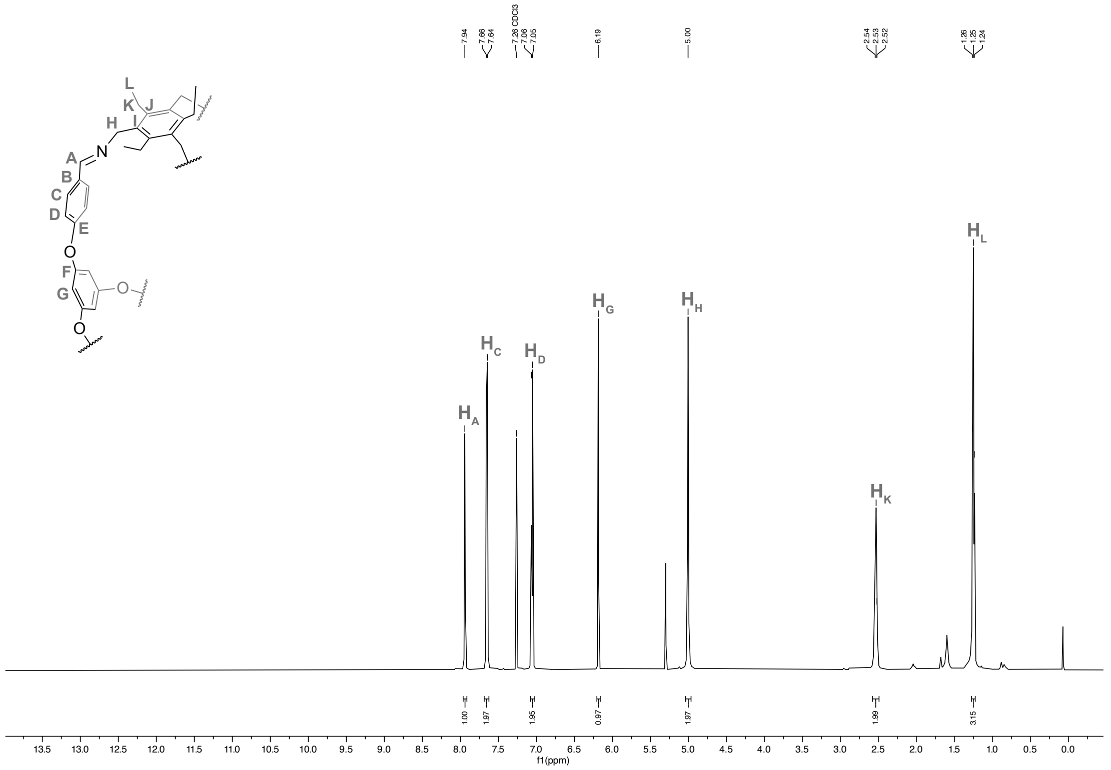

Figure S115: ^{1}H NMR (600 MHz, $CDCl_3$) of imine cage **Et⁴H⁴**.

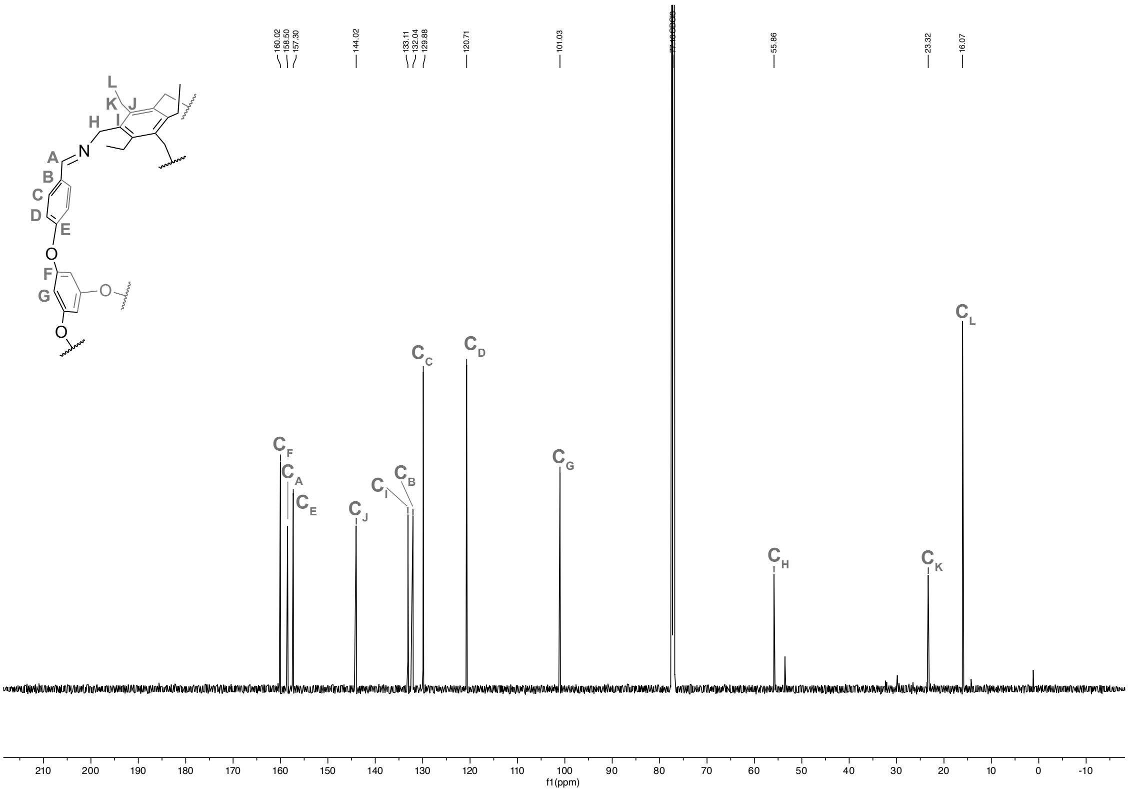

Figure S116: $^{13}C\{^{1}H\}$ NMR (151 MHz, $CDCl_3$) of imine cage **Et⁴H⁴**.

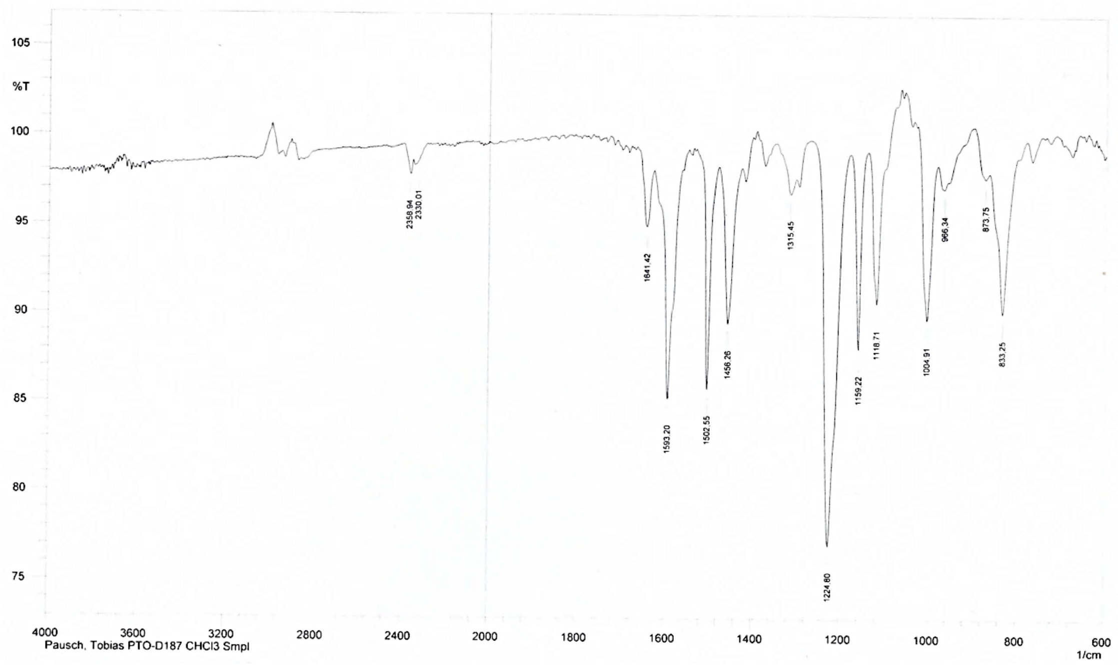

Figure S117: AT-IR spectrum of imine cage **Et^{4}H^4**.

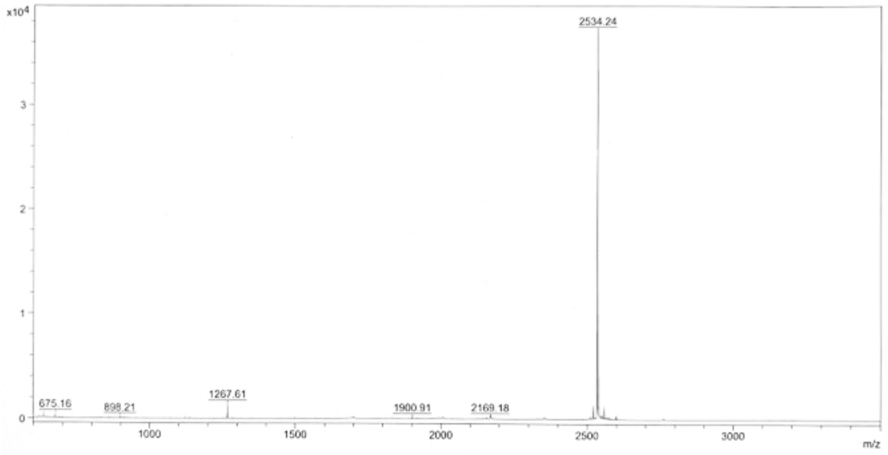

Figure S118: MS (MALDI) spectrum of imine cage **Et^{4}H^4**.

Imine Cage TREN⁴H⁴

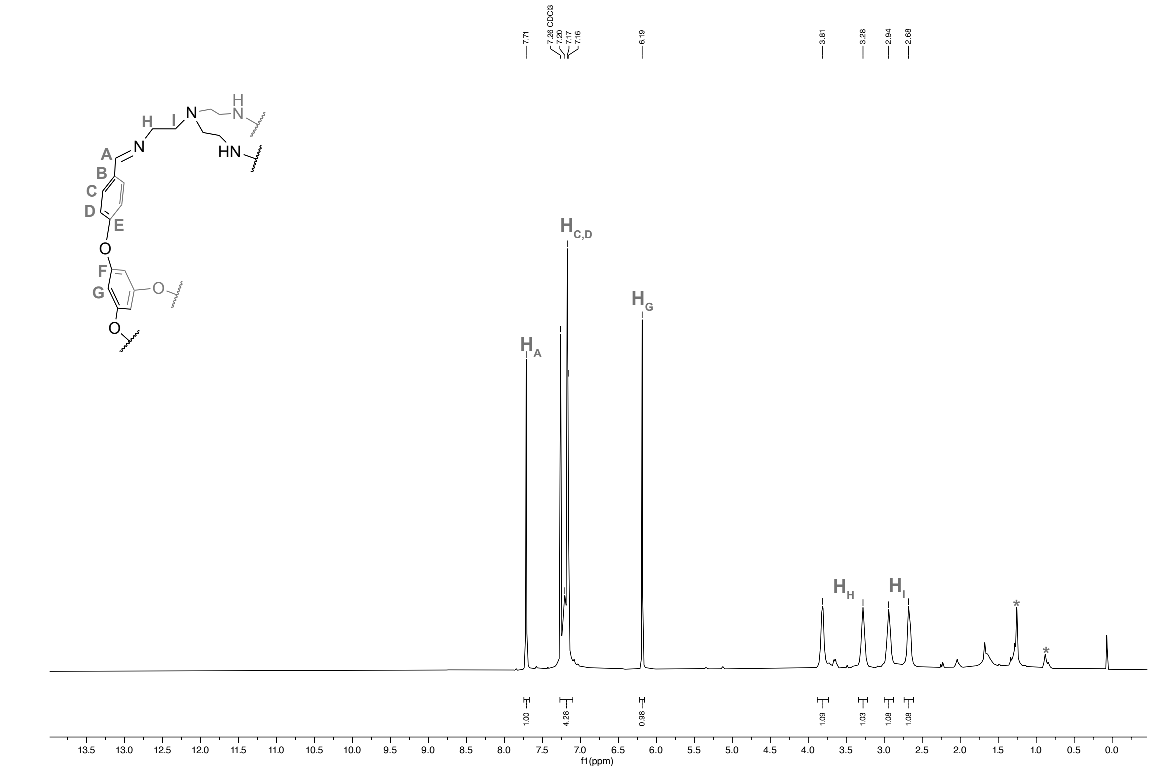

Figure S119: ^{1}H NMR (600 MHz, $CDCl_3$) of imine cage **TREN⁴H⁴**.

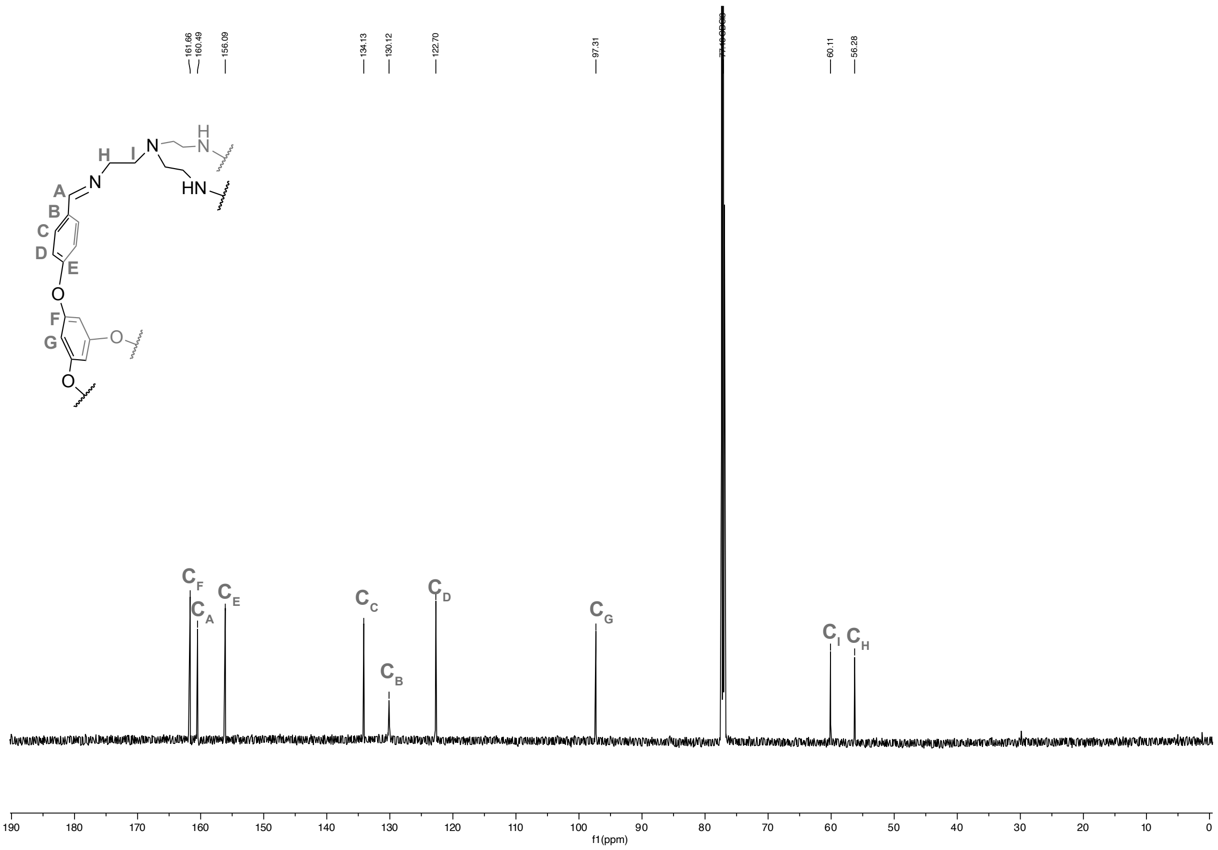

Figure S120: $^{13}C\{^{1}H\}$ NMR (151 MHz, $CDCl_3$) of imine cage **TREN⁴H⁴**.

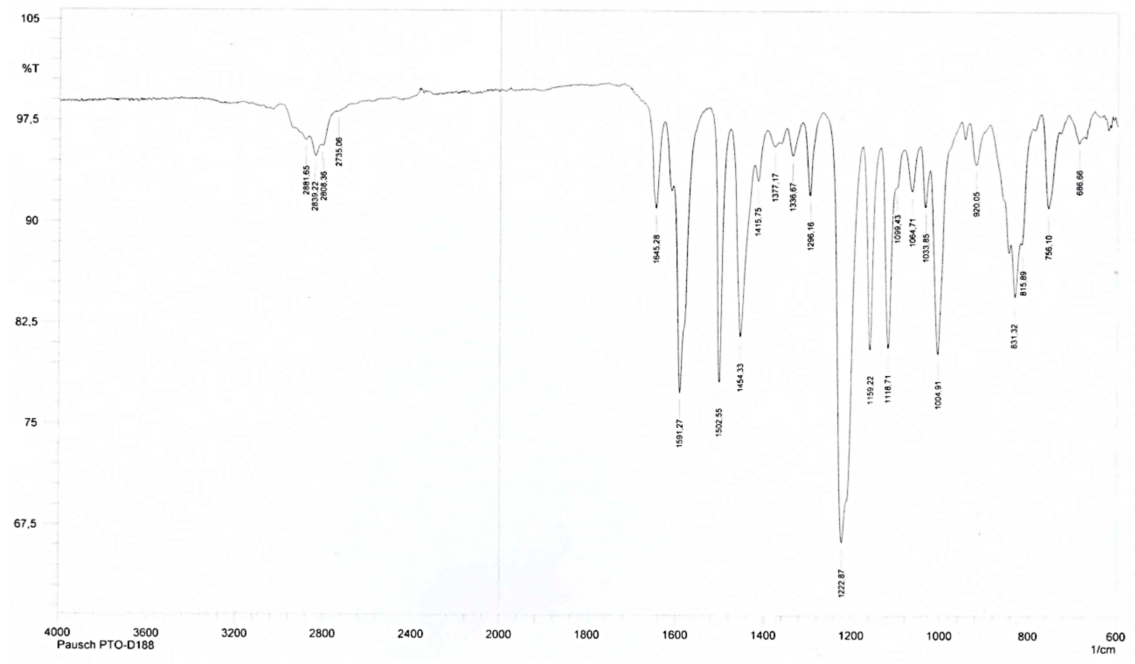

Figure S121: AT-IR spectrum of imine cage **TREN^{4}H^4**.

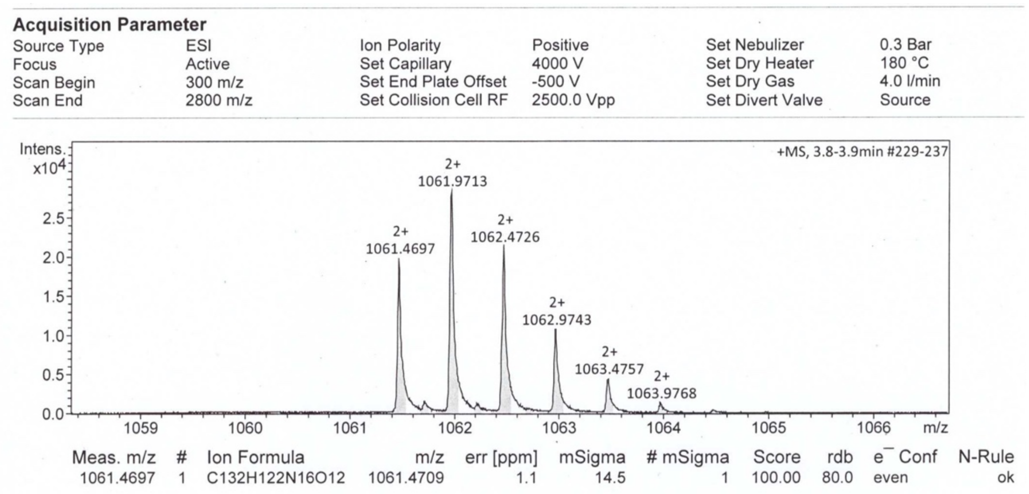

Figure S122: HR-MS (ESI) spectrum of imine cage **TREN^{4}H^4**.

Fluorinated Amin Cage $Et^2F^2_{red}$

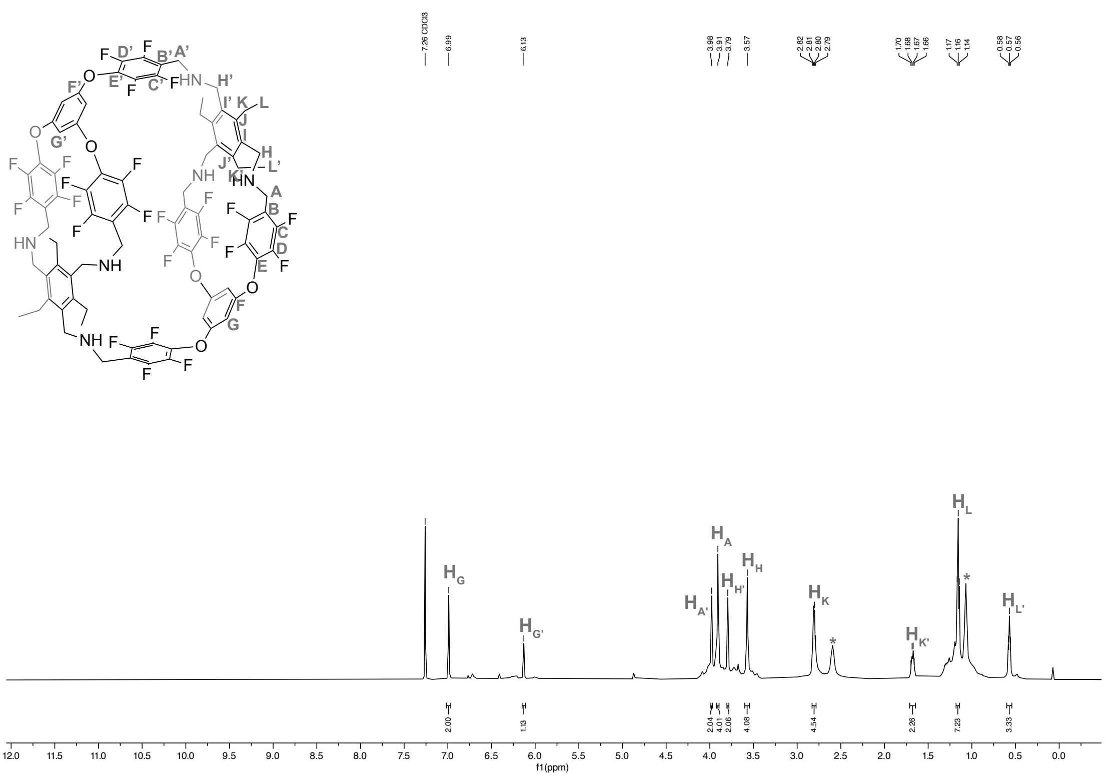

Figure S123: ^1H NMR (600 MHz, $CDCl_3$) of fluorinated amine cage $\mathbf{Et^2F^2_{red}}$. *triethylamine

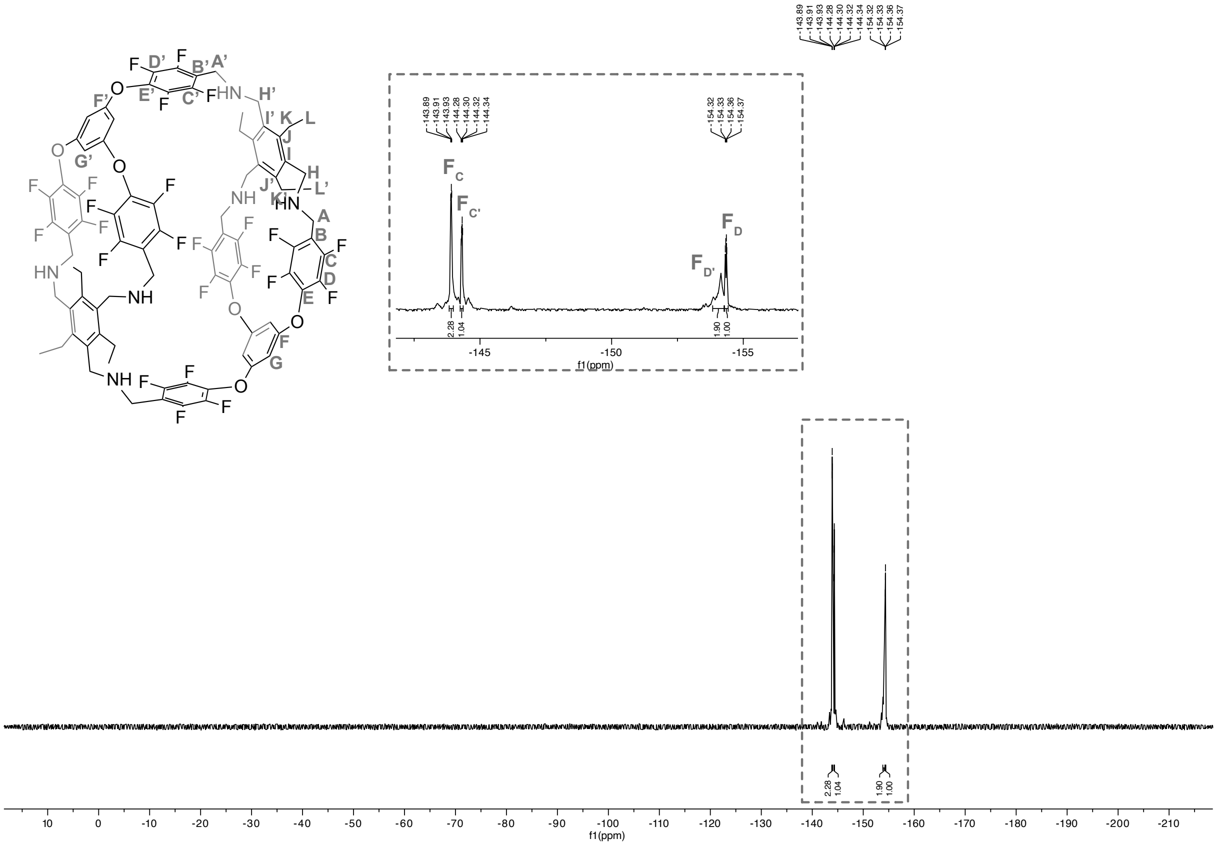

Figure S124: ^{19}F NMR (565 MHz, $CDCl_3$) of fluorinated amine cage $\mathbf{Et^2F^2_{red}}$.

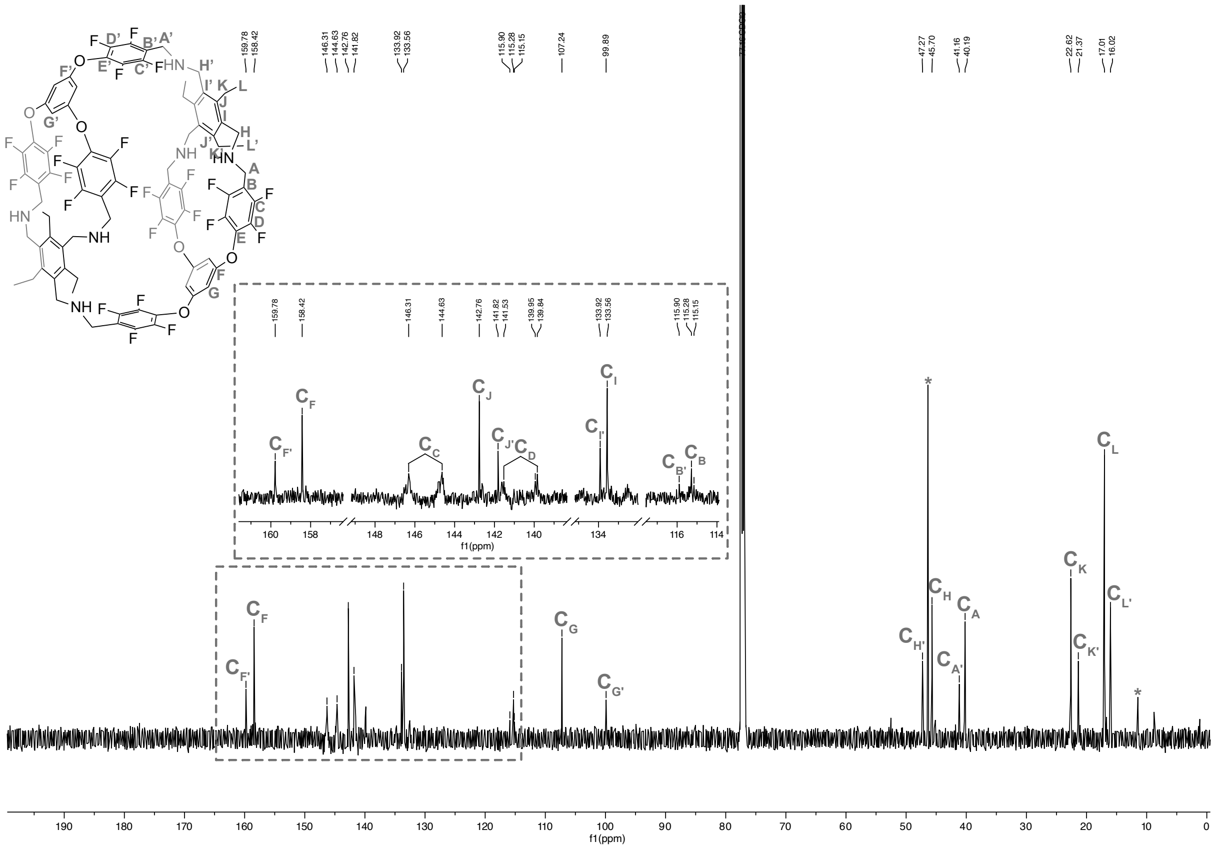

Figure S125: $^{13}C\{^{1}H\}$ NMR (151 MHz, $CDCl_3$) of fluorinated amine cage **$Et^2F^2{}_{red}$**. *triethylamine

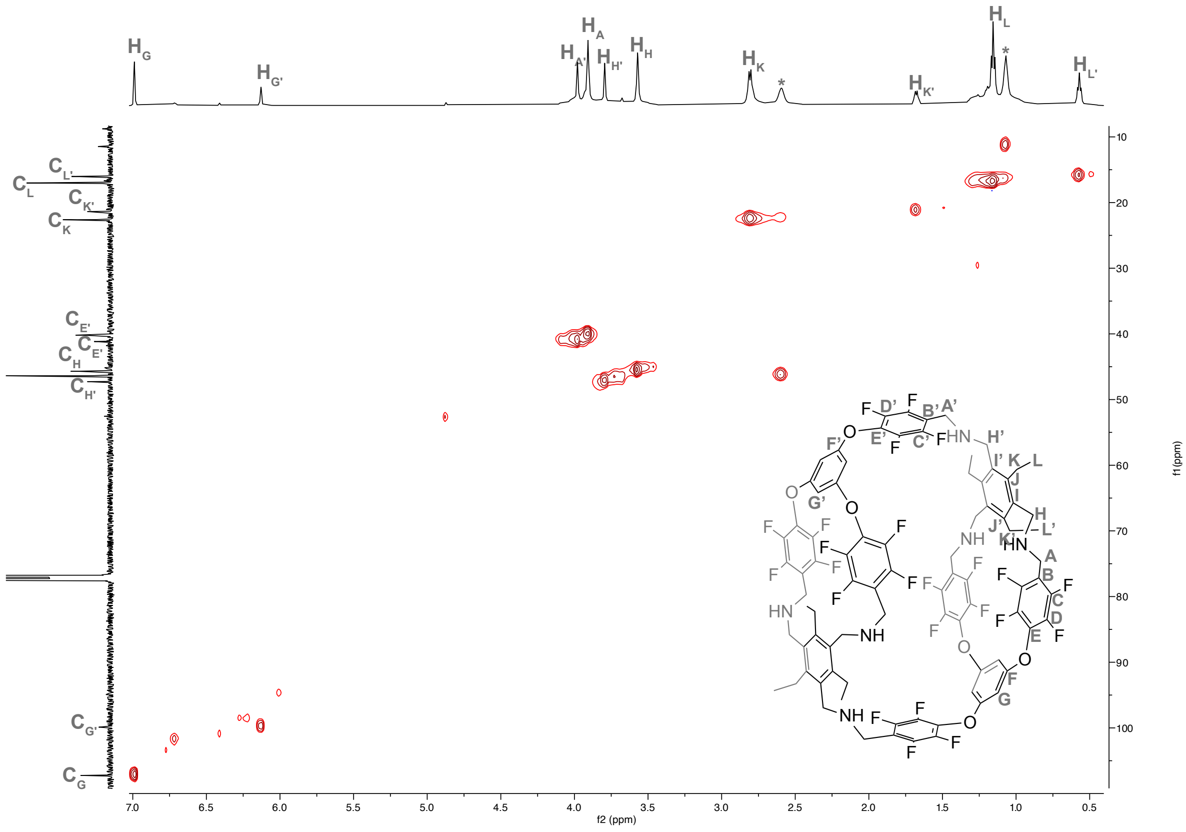

Figure S126: ^{1}H-^{13}C HSQC NMR (600 MHz, 151 MHz, $CDCl_3$) of fluorinated amine cage **$Et^2F^2{}_{red}$**.

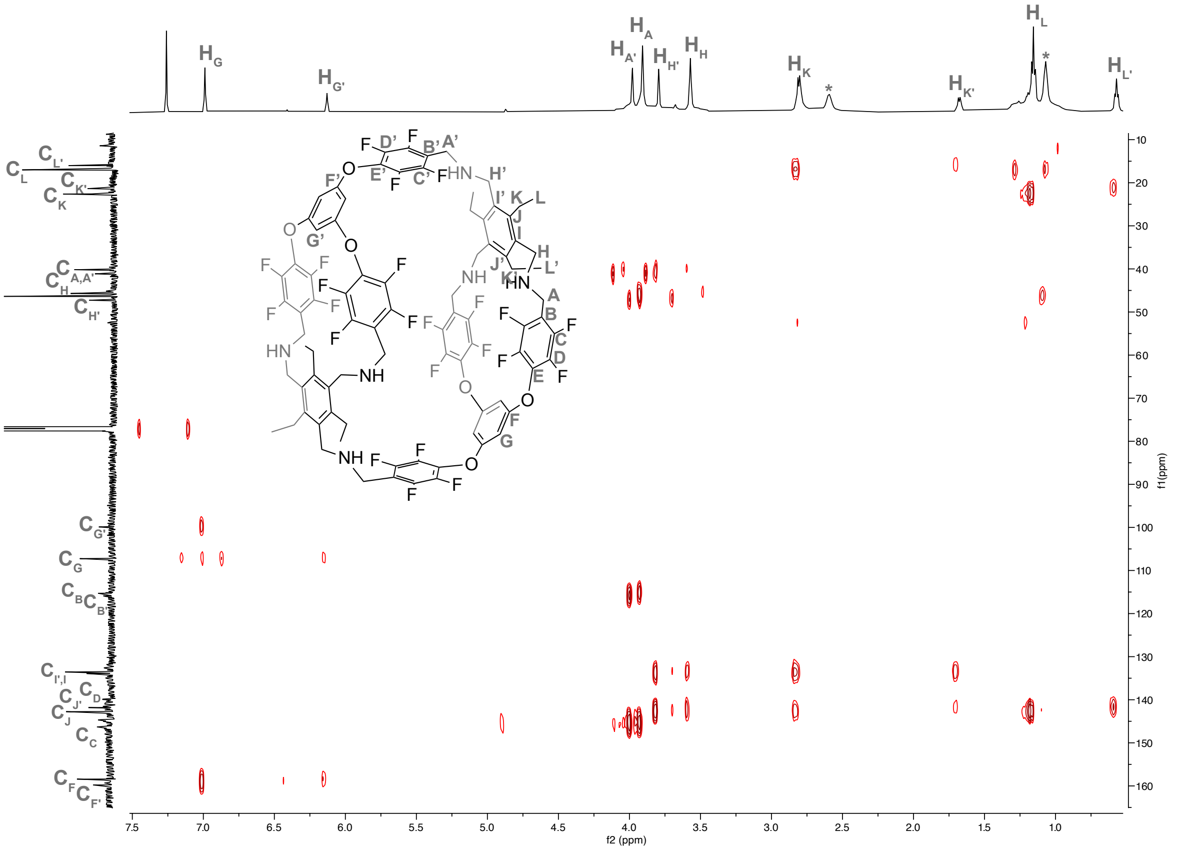

Figure S127: ^{1}H-^{13}C HMBC NMR (600 MHz, 151 MHz, $CDCl_3$) of fluorinated amine cage **$Et^2F^2_{red}$**.

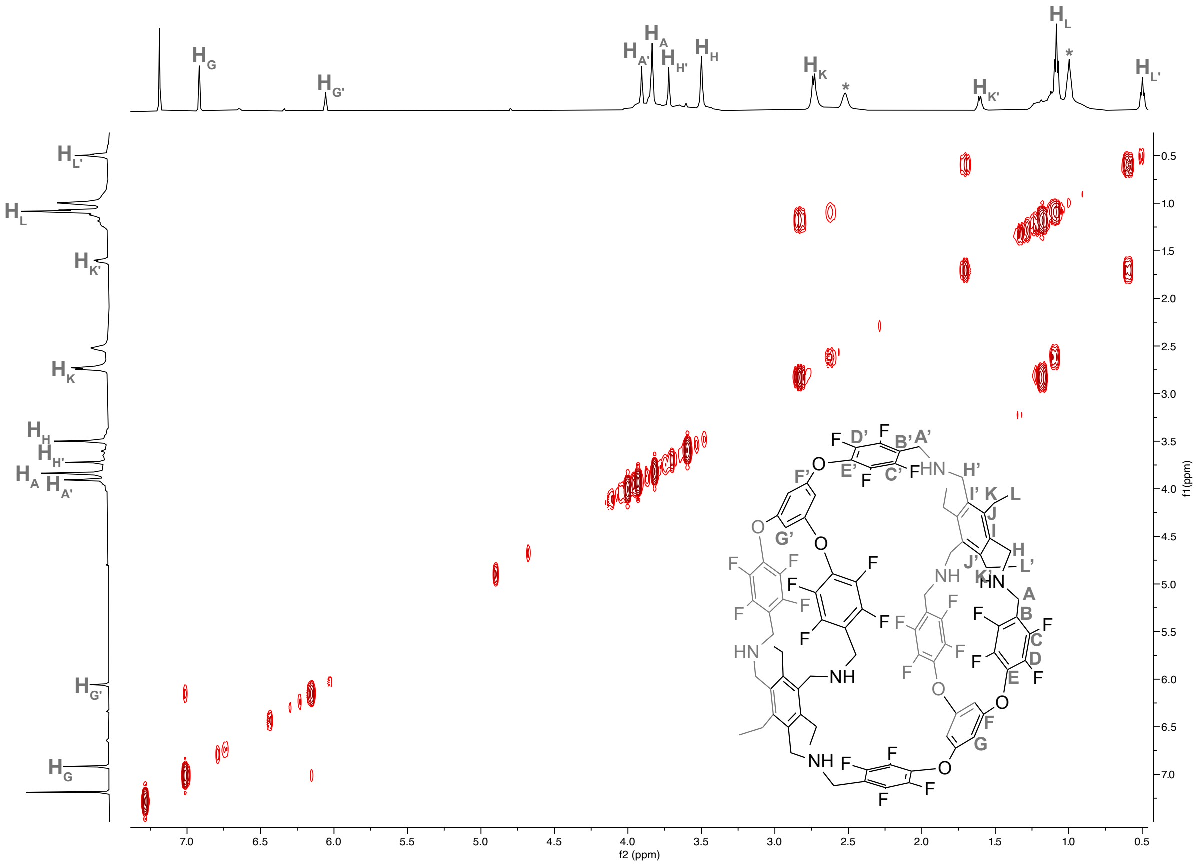

Figure S128: ^{1}H-COSY NMR (600 MHz, $CDCl_3$) of fluorinated amine cage **$Et^2F^2_{red}$**.

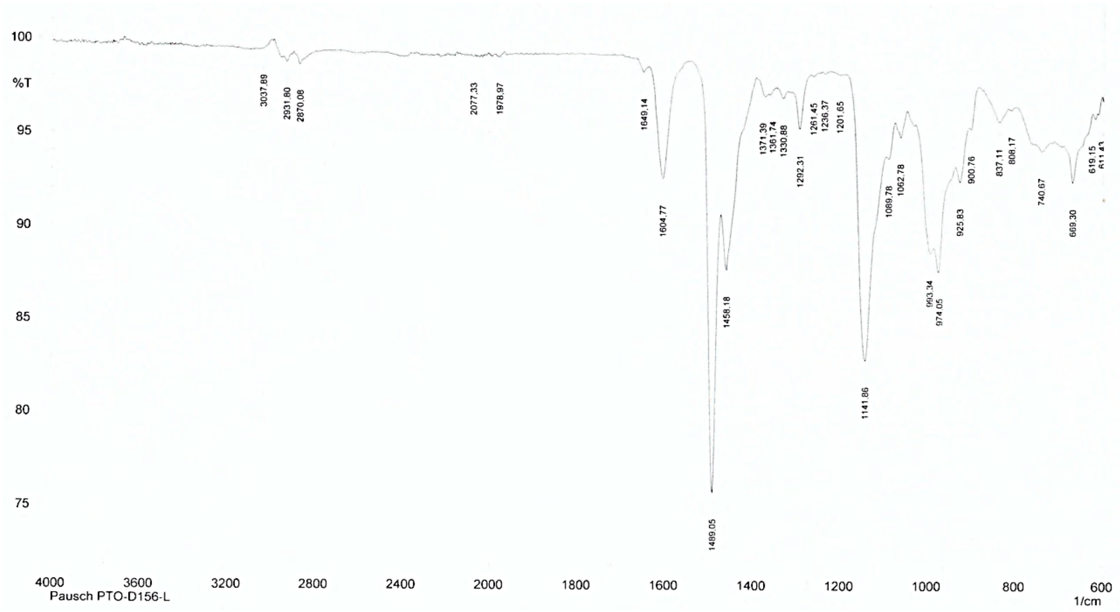

Figure S129: AT-IR spectrum of fluorinated amine cage $\mathbf{Et^2F^2_{red}}$.

Acquisition Parameter

Source Type	ESI	Ion Polarity	Positive	Set Nebulizer	0.3 Bar
Focus	Active	Set Capillary	4000 V	Set Dry Heater	180 °C
Scan Begin	300 m/z	Set End Plate Offset	-500 V	Set Dry Gas	4.0 l/min
Scan End	2800 m/z	Set Collision Cell RF	2500.0 Vpp	Set Divert Valve	Source

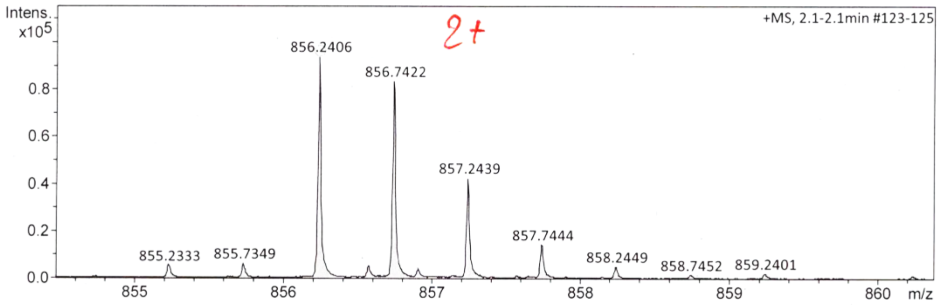

Meas. m/z	#	Ion Formula	m/z	err [ppm]	mSigma	# mSigma	Score	rdb	e⁻ Conf	N-Rule
856.2406	1	C83H72F24N2O10	856.2396	-1.2	18.0	1	83.42	37.0	even	ok
	2	C80H64F24N12O4	856.2390	-1.9	23.4	2	48.45	43.0	even	ok
	3	C84H68F24N6O6	856.2403	-0.4	25.2	3	100.00	42.0	even	ok
	4	C81H60F24N16	856.2396	-1.2	28.3	4	66.01	48.0	even	ok
	5	C85H64F24N10O2	856.2410	0.4	35.3	5	77.03	47.0	even	ok
	6	C88H72F24O8	856.2416	1.2	35.5	6	54.18	41.0	even	ok
	7	C89H68F24N4O4	856.2423	2.0	47.5	7	25.38	46.0	even	ok

Figure S130: HR-MS (ESI) spectrum of fluorinated amine cage $\mathbf{Et^2F^2_{red}}$.

Fluorinated Amin Cage TREN^{2}F$^2_{red}$

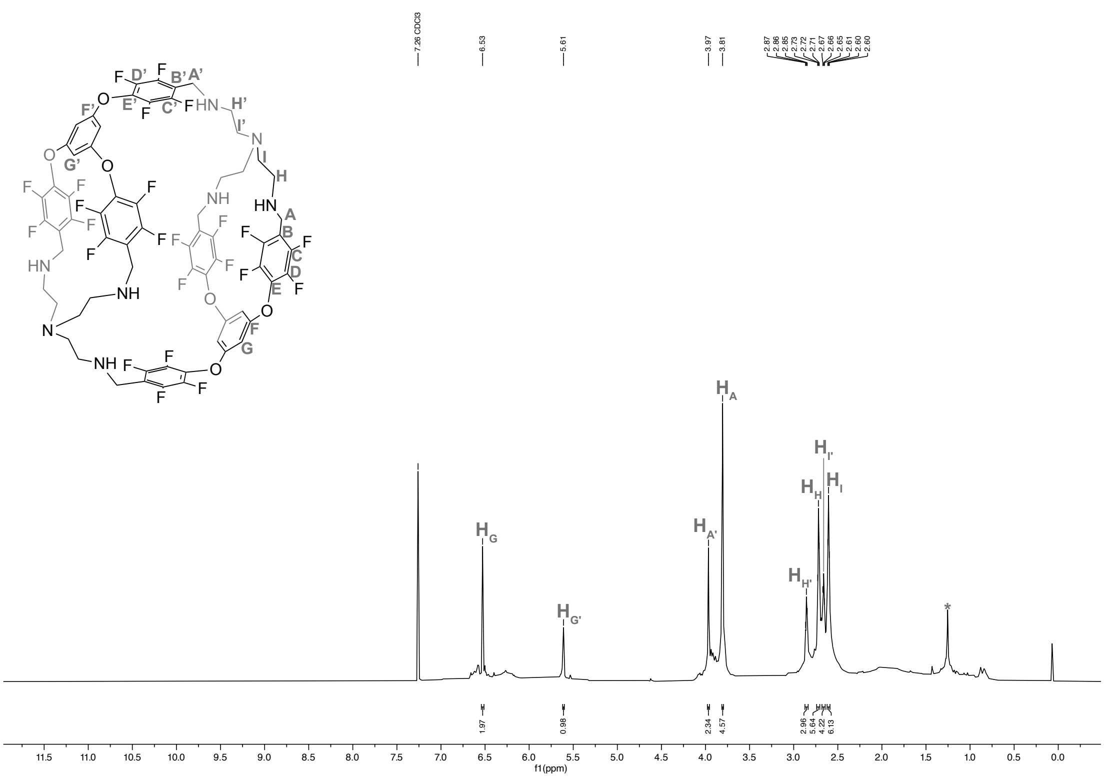

Figure S131: ^{1}H NMR (600 MHz, CDCl$_3$) of fluorinated amine cage **TREN^{2}F$^2_{red}$**.

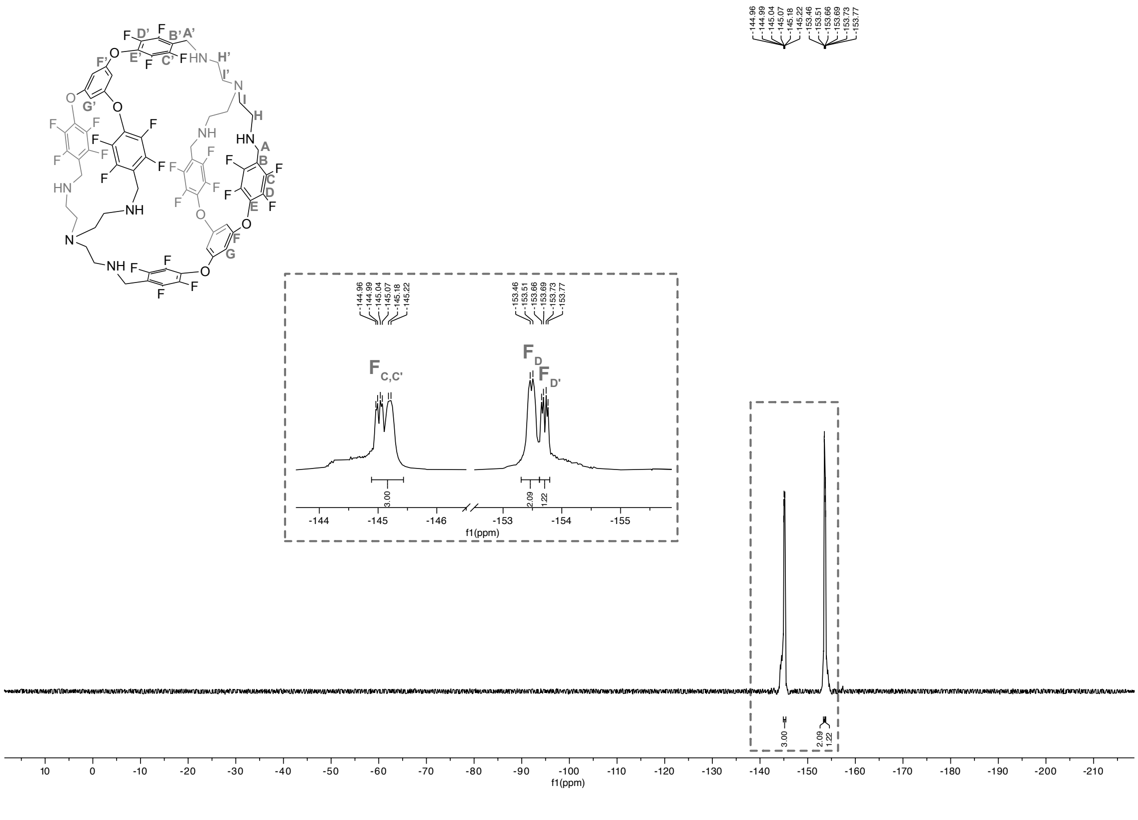

Figure S132: ^{19}F NMR (282 MHz, CDCl$_3$) of fluorinated amine cage **TREN^{2}F$^2_{red}$**.

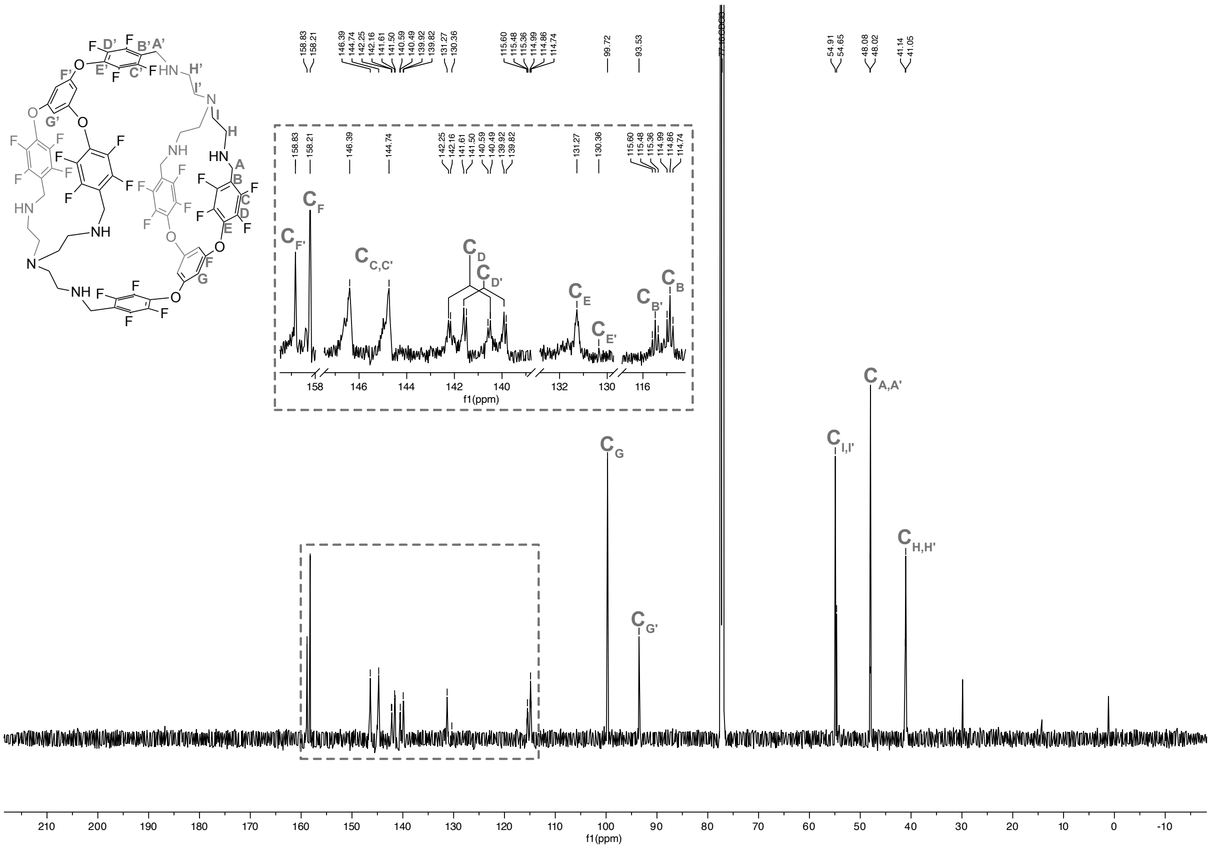

Figure S133: $^{13}C\{^{1}H\}$ NMR (151 MHz, $CDCl_3$) of fluorinated amine cage **TREN²F²red**.

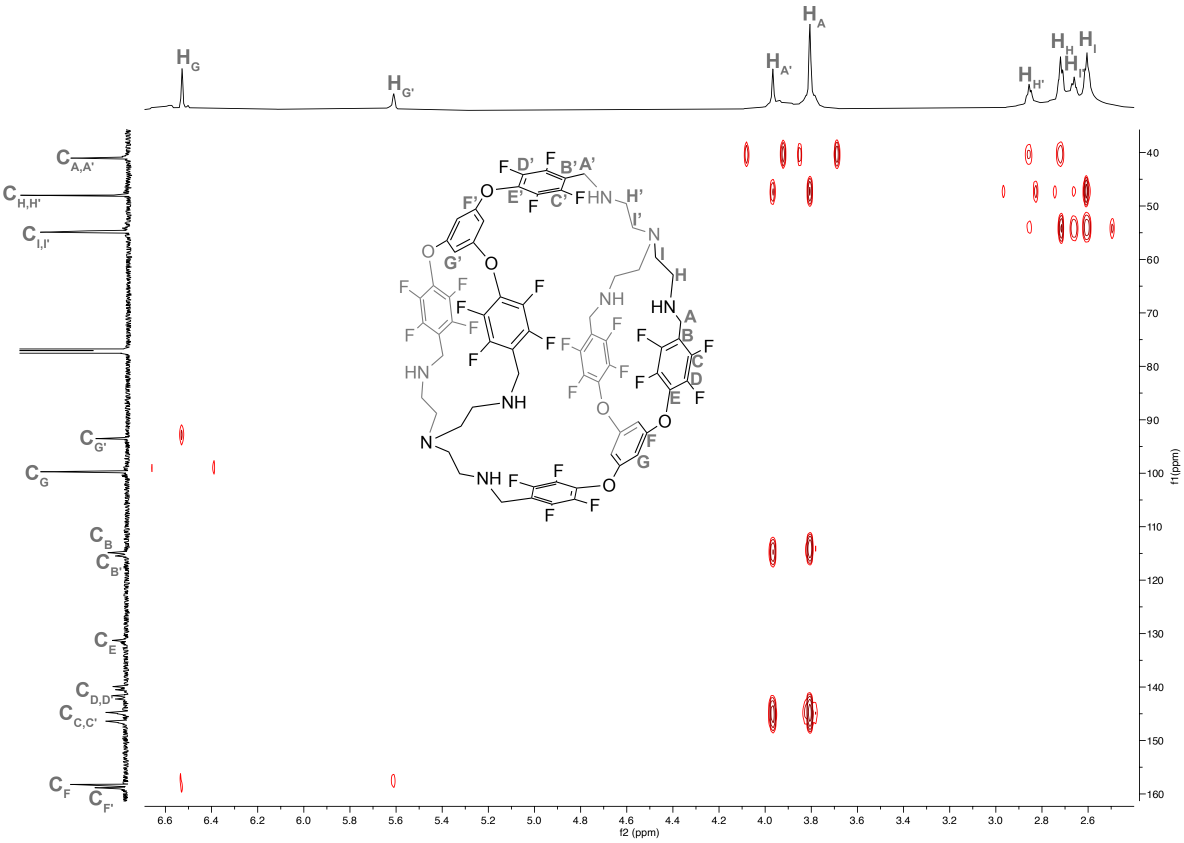

Figure S134: ^{1}H-^{13}C HSQC NMR (600 MHz, 151 MHz, $CDCl_3$) of fluorinated amine cage **TREN²F²red**.

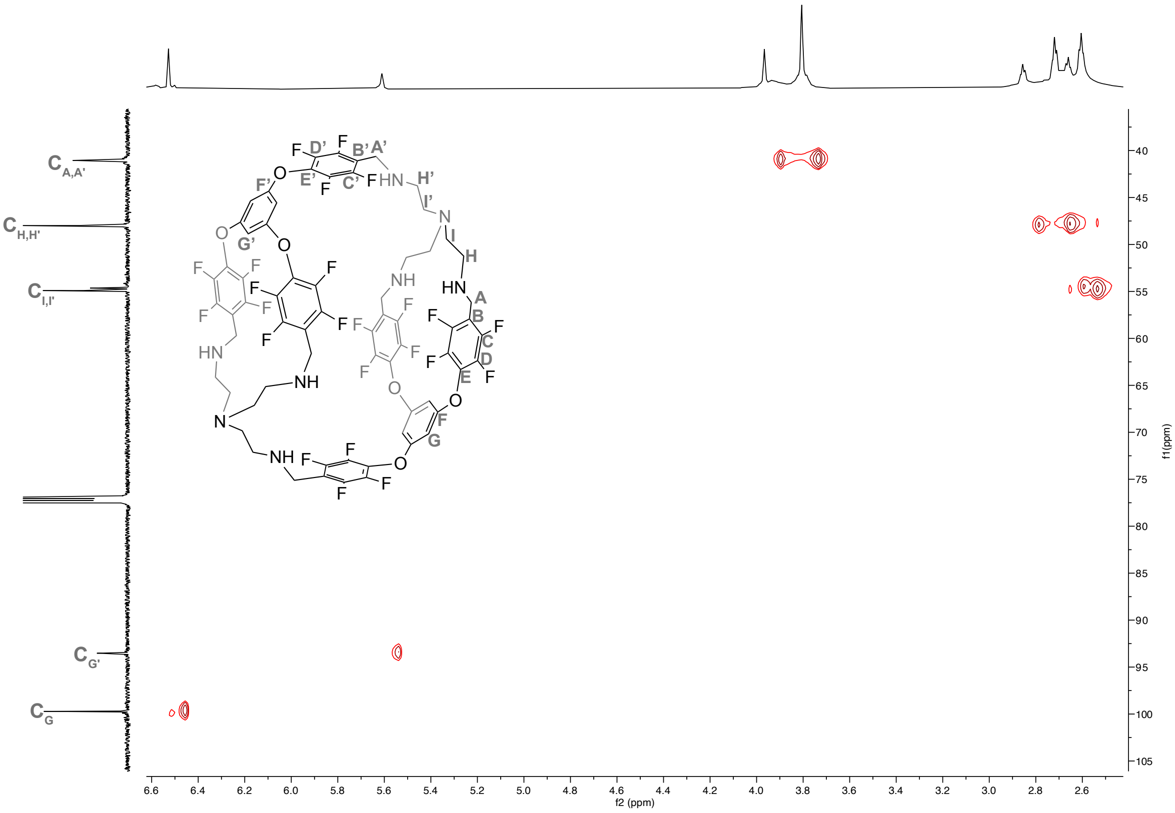

Figure S135: ^{1}H-^{13}C HMBC NMR (600 MHz, 151 MHz, $CDCl_3$) of fluorinated amine cage **TREN^{2}F^2$_{red}$**.

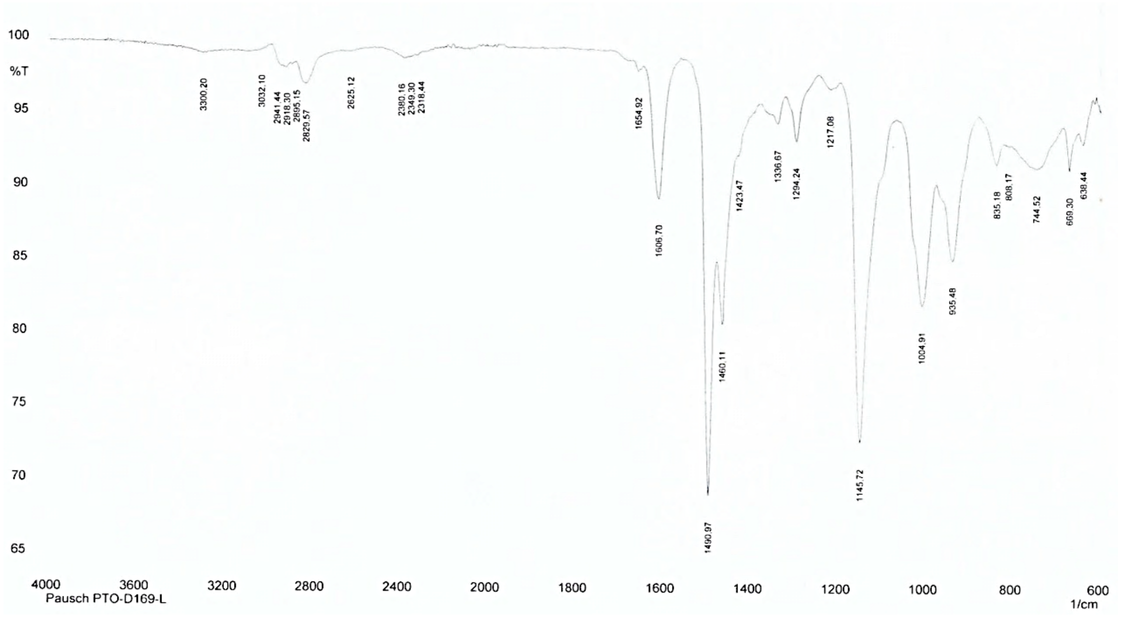

Figure S136: AT-IR spectrum of fluorinated amine cage **TREN^{2}F^2$_{red}$**.

Acquisition Parameter

Source Type	ESI	Ion Polarity	Positive	Set Nebulizer	0.3 Bar
Focus	Active	Set Capillary	4000 V	Set Dry Heater	180 °C
Scan Begin	300 m/z	Set End Plate Offset	-500 V	Set Dry Gas	4.0 l/min
Scan End	2800 m/z	Set Collision Cell RF	2500.0 Vpp	Set Divert Valve	Source

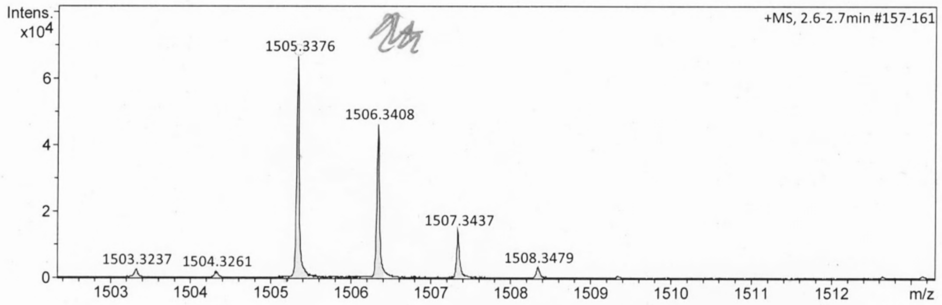

Meas. m/z	#	Ion Formula	m/z	err [ppm]	mSigma	# mSigma	Score	rdb	e⁻ Conf	N-Rule
1505.3376	1	C64H57F24O14	1505.3360	-1.1	27.0	1	55.43	24.5	even	ok
	2	C65H53F24N4O10	1505.3373	-0.2	33.5	2	100.00	29.5	even	ok
	3	C63H41F24N18	1505.3373	-0.2	37.7	3	89.37	40.5	even	ok
	4	C66H49F24N8O6	1505.3386	0.7	42.3	4	52.54	34.5	even	ok

Figure S137: HR-MS (ESI) spectrum of fluorinated amine cage **TREN^{2}F$^2_{red}$**.

Modelcompund $Bn^3F^1_{red}$

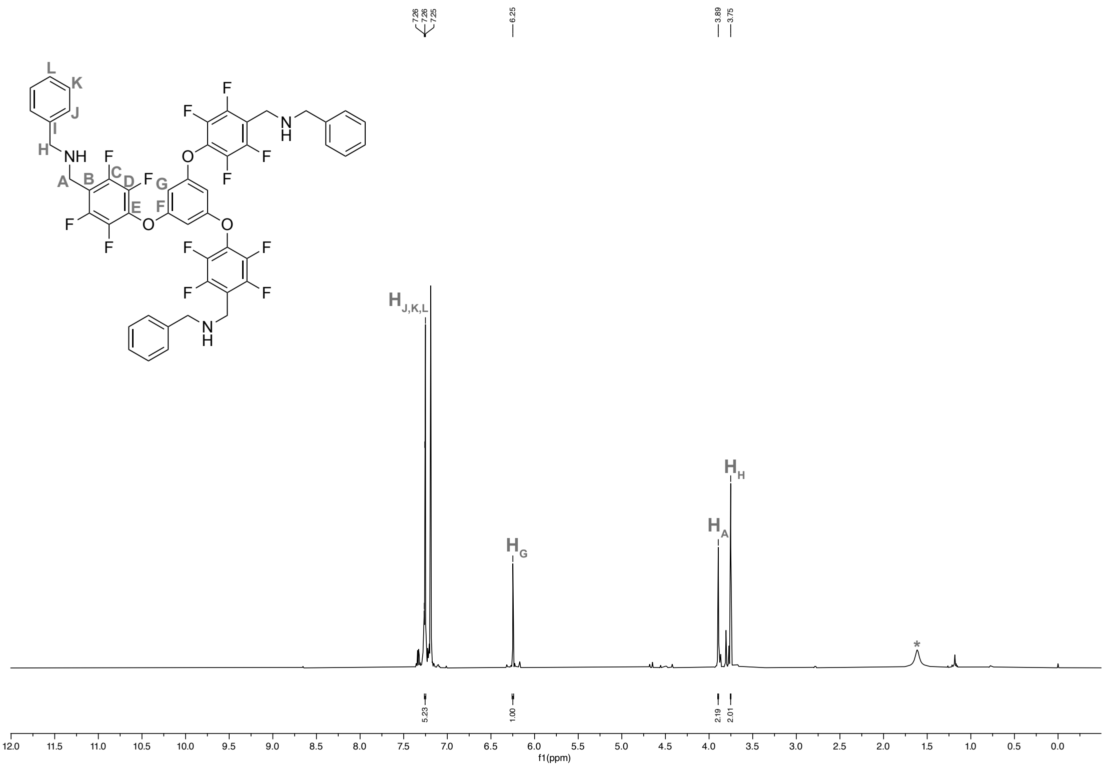

Figure S138: ^{1}H NMR (600 MHz, $CDCl_3$) of model compound $\mathbf{Bn^3F^1_{red}}$.

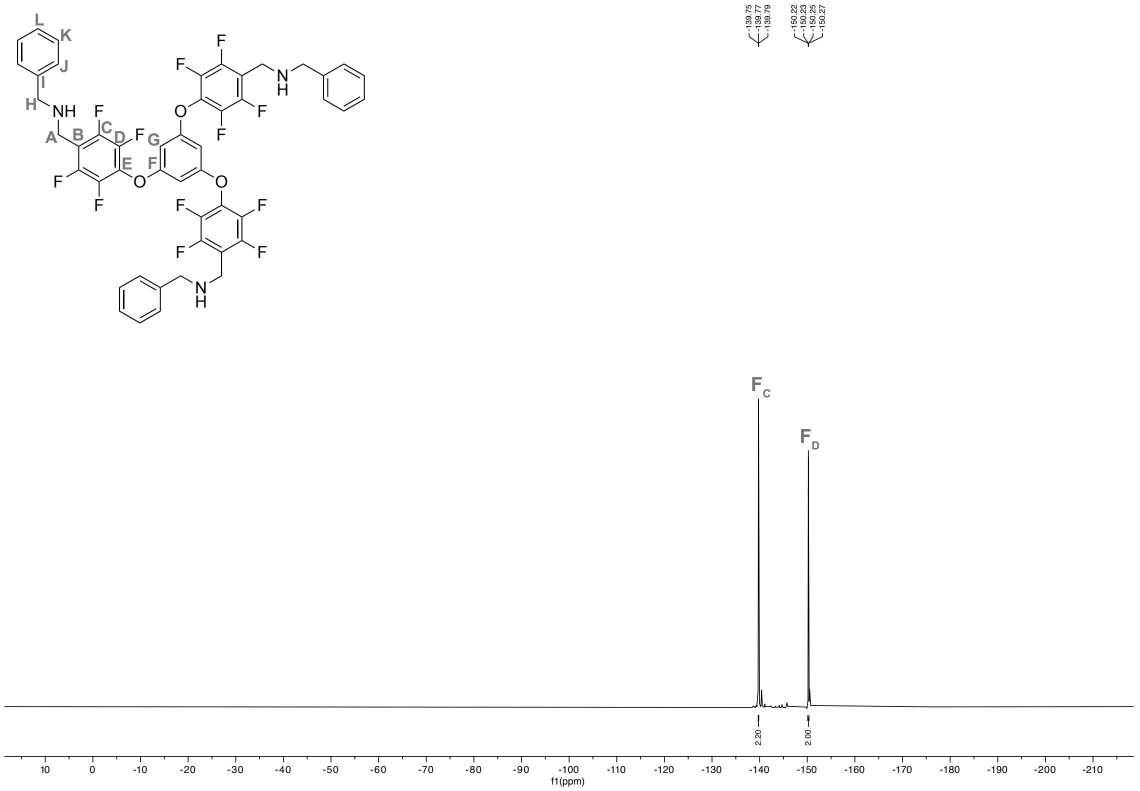

Figure S139: ^{19}F NMR (565 MHz, $CDCl_3$) of model compound $\mathbf{Bn^3F^1_{red}}$.

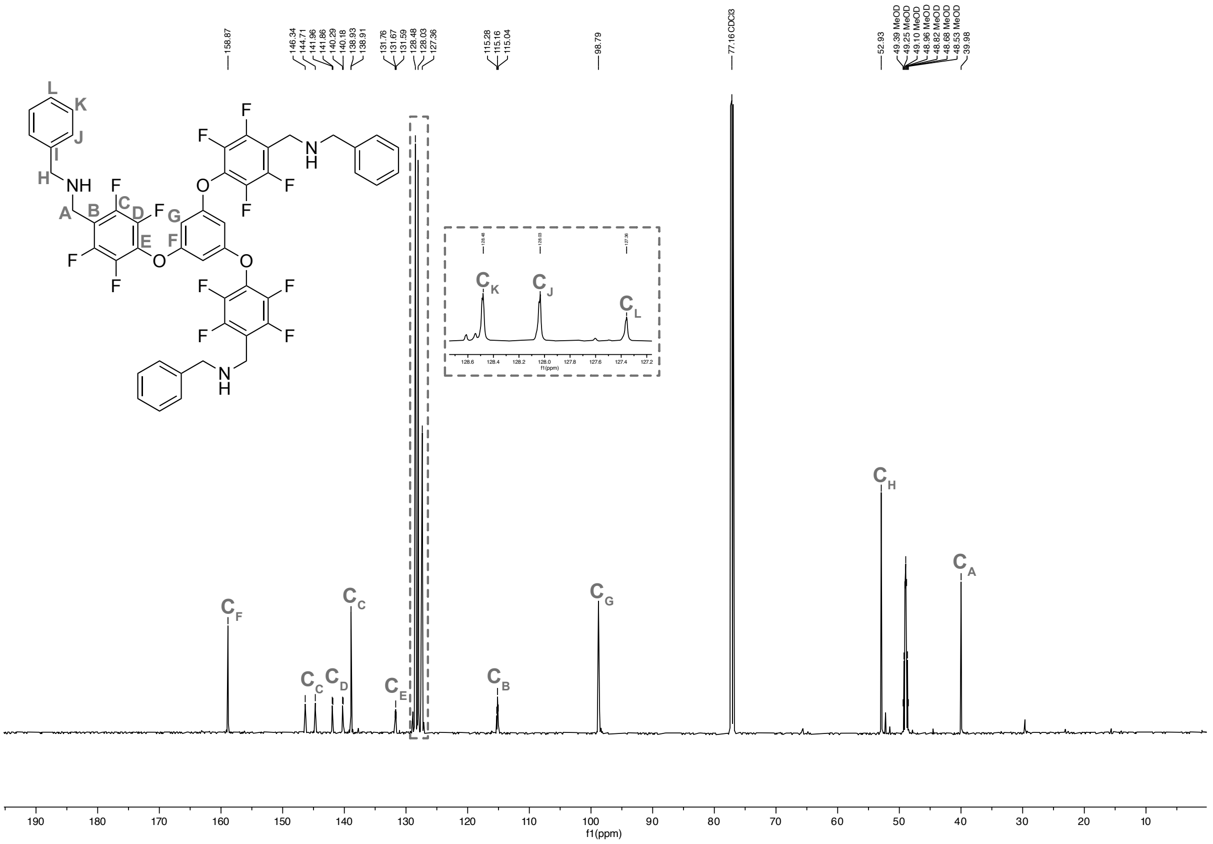

Figure S140: $^{13}C\{^1H\}$ NMR (151 MHz, $CDCl_3$) of model compound $\mathbf{Bn^3F^1_{red}}$.

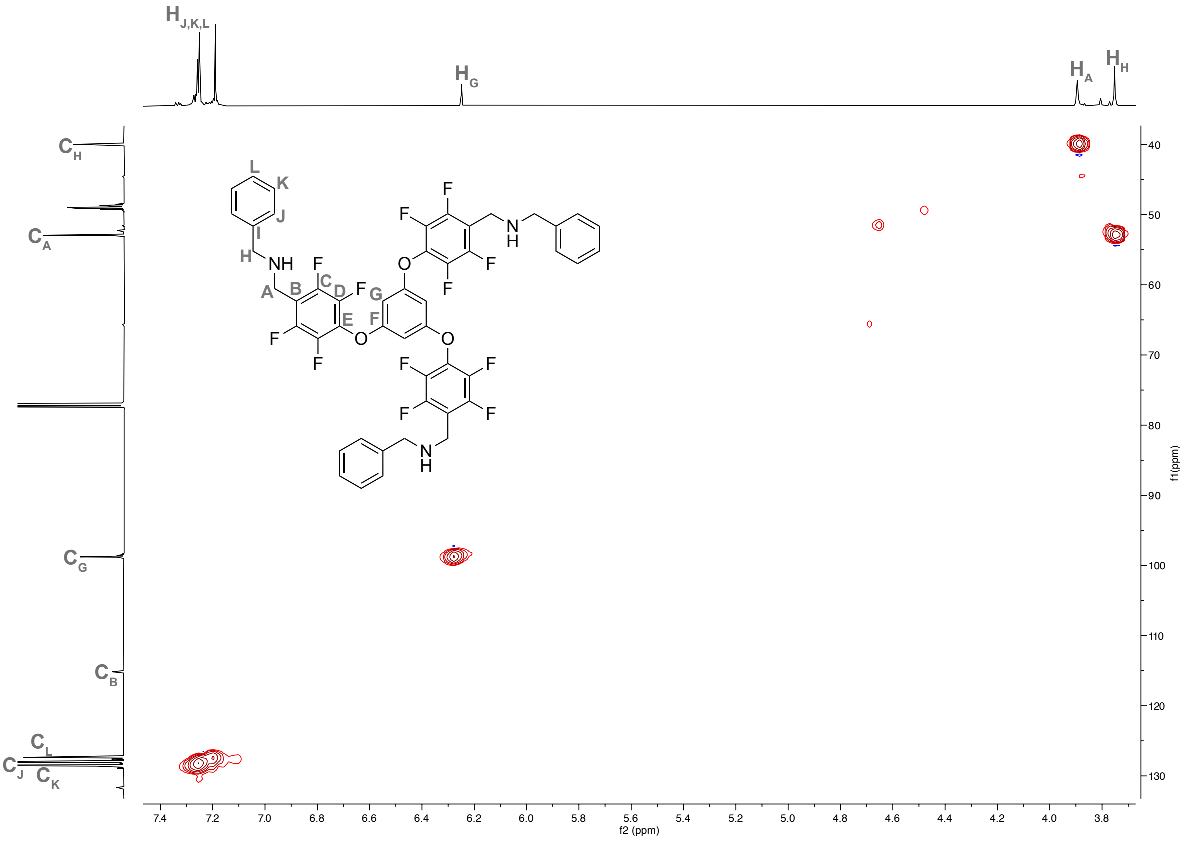

Figure S141: ^1H-^{13}C HSQC NMR (600 MHz, 151 MHz, $CDCl_3$) of model compound $\mathbf{Bn^3F^1_{red}}$.

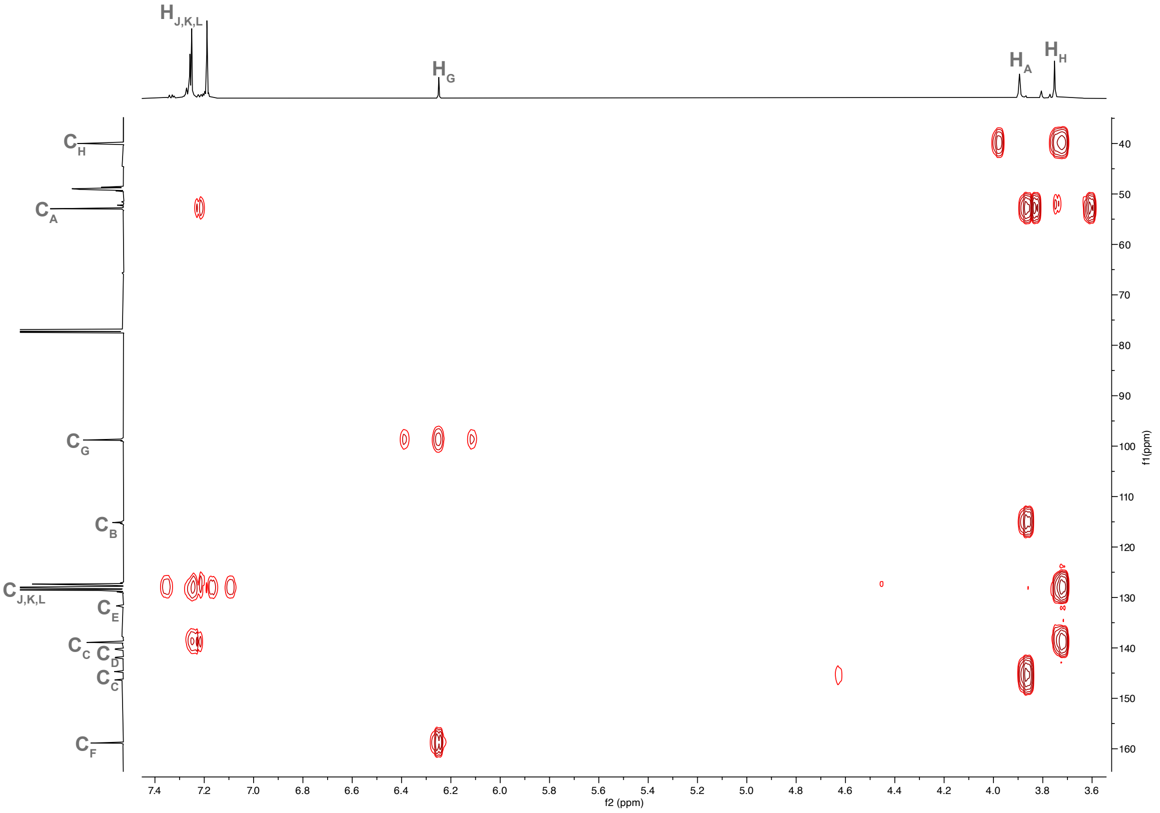

Figure S142: ^{1}H-^{13}C HMBC NMR (600 MHz, 151 MHz, $CDCl_3$) of fluorinated amine cage **$Bn^3F^1_{red}$**.

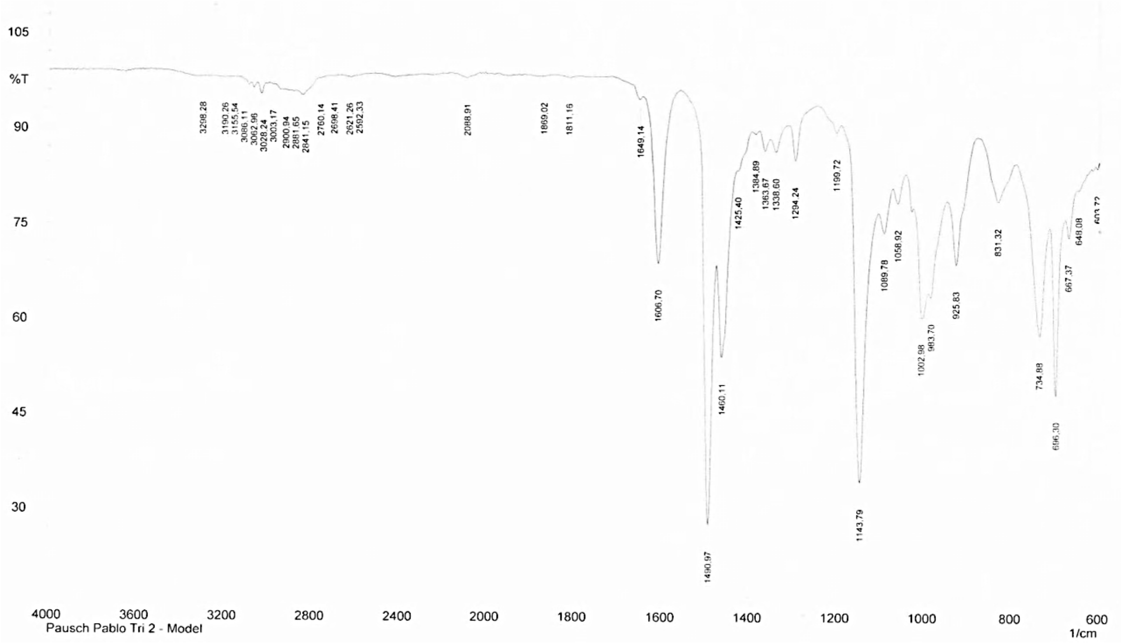

Figure S143: AT-IR spectrum of fluorinated amine cage **$Bn^3F^1_{red}$**.

Acquisition Parameter

Source Type	ESI	Ion Polarity	Positive	Set Nebulizer	0.3 Bar
Focus	Not active	Set Capillary	4000 V	Set Dry Heater	180 °C
Scan Begin	50 m/z	Set End Plate Offset	-500 V	Set Dry Gas	4.0 l/min
Scan End	1500 m/z	Set Collision Cell RF	600.0 Vpp	Set Divert Valve	Source

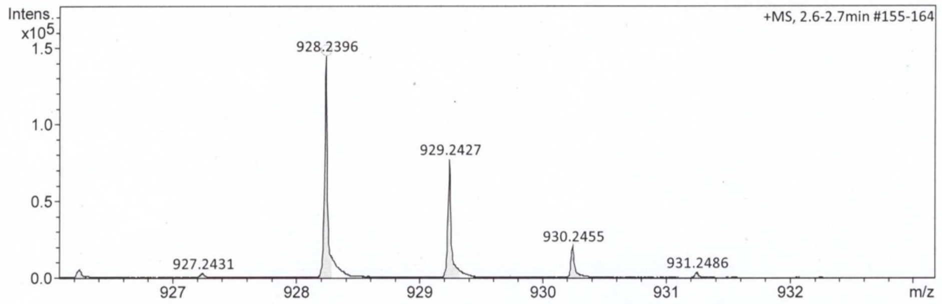

Meas. m/z	#	Ion Formula	m/z	err [ppm]	mSigma	# mSigma	Score	rdb	e⁻ Conf	N-Rule
928.2396	1	C48H34F12N3O3	928.2403	0.8	2.1	1	100.00	27.5	even	ok
	2	C44H30F12N9O	928.2376	-2.1	11.9	2	38.92	28.5	even	ok

Figure S144: HR-MS (ESI) spectrum of fluorinated amine cage **$Bn^3F^1_{red}$**.

XI. References

[S1] A. Karmakar, A. Kumar, A. K. Chaudhari, P. Samanta, A. V. Desai, R. Krishna, S. K. Ghosh, *Chem. Eur. J.* **2016**, *22*, 4931.

[S2] K. J. Wallace, R. Hanes, E. Anslyn, J. Morey, K. V. Kilway, J. Siegel, *Synthesis* **2005**, *12*, 2080–2083.

[S3] G. M. Sheldrick, *Acta Crystallogr., Sect. C: Struct. Chem.* **2015**, *71*, 3–8.

[S4] A. L. Spek, Acta Cryst. C **2015**, *71*, 9–18.

[S5] S. Viel, F. Ziarelli, G. Pages, C. Carrara, S. Caldarelli, *J. Magn. Reson.* **2008**, *190*, 113–123.

[S6] D. Li, G. Kagan, R. Hopson, P. G. Williard, *J. Am. Chem. Soc.* **2009**, *131*, 5627–5634.

[S7] L. J. Musegades, O. P. Curtin, J. D. Cyran, *J. Phys. Chem. C* **2024**, *128*, 1946–1951.

[S8] W. Ji, L. Xiao, Y.Ling. C. Ching, M. Matsumoto, R. P. Bisbey, D. E. Helbling, W. R. Dichtel, *J. Am. Chem. Soc.* **2018**, 140, 12677–12681.

[S9] M. E. Tighe, M. D. Thum, N. K. Weise, G. C. Daniels, *Chem. Eng. J.* **2024**, *499*, 156280.

[S10] W. Wang, Z. Zhou, H. Shao, S. Zhou, G. Yu, S. Deng, *Chem. Eng. J.* **2021**, *412*, 127509.

[S11] A. Chaix, C. Gomri, B. T. Benkhaled, M. Habib, R. Dupuis, E. Petit, J. Richard, A. Segala, L. Lichon, C. Nguyen, M. Gary-Bobo, S. Blanquer, M. Semsarilar, *Adv. Mater.* **2024**, *37*, 2410720.
